# Small-scale spatial variability in phylogenetic community structure during early plant succession depends on soil properties

**DOI:** 10.1007/s00442-014-2954-2

**Published:** 2014-05-09

**Authors:** Werner Ulrich, Marcin Piwczyński, Markus Klemens Zaplata, Susanne Winter, Wolfgang Schaaf, Anton Fischer

**Affiliations:** 1 Chair of Ecology and Biogeography, Nicolaus Copernicus University in Torun, Lwowska 1, 87-100 Toruń, Poland; 2 Geobotany, Center of Life and Food Sciences, Technische Universität München, Hans-Carl-von-Carlowitz-Platz 2, 85354 Freising, Germany; 3 Center of Landscape Development and Mining Landscapes (FZLB), Brandenburg University of Technology Cottbus-Senftenberg, Konrad-Wachsmann-Allee 6, 03046 Cottbus, Germany; 4 Chair of Land Improvement and Nature Conservation, Technische Universität Dresden, Pienner Str. 8, 01737 Tharandt, Germany; 5 Soil Protection and Recultivation, Brandenburg University of Technology Cottbus-Senftenberg, Konrad-Wachsmann-Allee 6, 03046 Cottbus, Germany

**Keywords:** Primary succession, Spatial analysis, Niche conservatism, Species co-occurrence, Null model

## Abstract

During early plant succession, the phylogenetic structure of a community changes in response to important environmental filters and emerging species interactions. We traced the development of temperate-zone plant communities during the first 7 years of primary succession on catchment soils to explore patterns of initial species assembly. We found pronounced small-scale differences in the phylogenetic composition of neighbouring plant assemblages and a large-scale trend towards phylogenetic evenness. This small-scale variability appears to be mediated by soil properties, particularly carbonate content. Therefore, abiotic environmental conditions might counteract or even supersede the effects of interspecific competition among closely related species, which are usually predicted to exhibit patterns of phylogenetic evenness. We conclude that theories on phylogenetic community composition need to incorporate effects of small-scale variability of environmental factors.

**Electronic supplementary material:**

The online version of this article (doi:10.1007/s00442-014-2954-2) contains supplementary material, which is available to authorized users.

## Introduction

Darwin (1859) noticed that closely related species often tend to have similar ecological traits. If local community assembly from a regional species pool is governed by environmental filters that select for particular traits, this niche conservatism (Lord et al. 1995; Ackerly 2004; Losos 2008) should lead to local communities that are phylogenetically similar and share a close evolutionary history (Webb et al. 2002; Wiens et al. 2010; Whitfeld et al. 2011). Conversely, competitive forces may tend to limit the co-occurrence of ecologically similar species (Elton 1946; Svenning et al. 2008; Allan et al. 2013), leading to a community that is phylogenetically over-dispersed. Unless these two forces precisely balance one another, plant species composition will not occur randomly with respect to phylogenetic relatedness, and will be either phylogenetically clustered (species being on average more closely related than expected) or phylogenetically even (species being less closely related than expected) (Elton 1946; Webb 2000; Webb et al. 2002).

In some cases, phylogenetic community structure (*PCS*) has been found to vary with environmental conditions. Dinnage (2009), Helmus et al. (2010), and Cavender-Bares and Reich (2012), for example, reported on a shift towards phylogenetic clustering after habitat disturbance. Different colonization conditions (Cadotte and Strauss 2011) or biotic and abiotic constraints (Machac et al. 2011) might force communities either towards phylogenetic evenness or towards clustering depending on the trade-offs among competitive ability, niche differentiation, and environmental conditions. Small-scale environmental variability might drive subsets of local communities towards phylogenetic clustering or evenness, generating a mosaic of varied phylogenetic patterns. However, there are few studies conducted at local scales to confirm these hypotheses. Particularly little is known about the influence of soil conditions on the phylogenetic community structure of plants, although Schreeg et al. (2010) found that phylogenetic structure in a tropical tree community varied along gradients of soil conditions and water availability.

Having its origin in the writings of Gleason (1926), the dispersal centred view of community assembly favours a random process of colonization from a regional species pool during succession. The neutral models popularized by Hubbell (2001) additionally assume ecological equivalence of species (identical trait space) within a given ecological guild. Neutrality predicts random patterns of species co-occurrence (Gotelli and McGill 2006; but see Ulrich 2004) and neither phylogenetic nor trait convergence or divergence (Götzenberger et al. 2011). Neutral community assembly was reported to be strongest in communities that are dominated by colonization (Zillio and Condit 2007).

**Fig. 1:**
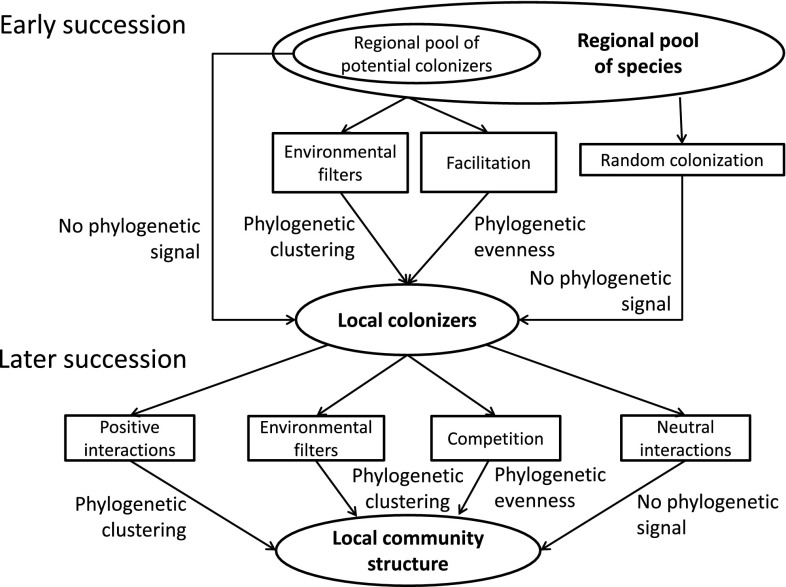
Flow chart showing major mechanisms leading to phylogenetic community structure

Early successional plant communities are predicted to be input-driven if random colonization and non-random habitat filtering dominate (Chazdon 2008). Later successional states are seen to be competition-driven, with biotic interactions generating segregated patterns of species occurrence (Baasch et al. 2009). A number of studies describe initially neutral community assembly followed by competitive or environmentally controlled assembly in late successional stages (e.g., Weiher and Keddy 1999; Zaplata et al. 2013; Ulrich et al. 2014). In both stages, phylogenetic community structure might be either clustered or even, depending on the species pool used for comparison and the type of biotic interactions considered (Fig. 1). In early succession, phylogenetic clustering is expected under strong environmental filtering, while facilitation (mainly among more distantly related species) should cause phylogenetic evenness (Fig. 1). In late succession, competitive effects might cause either phylogenetic evenness or phylogenetic clustering, while environmental filtering should lead to phylogenetic clustering only (Fig. 1). Neutral community assembly should not cause a significant *PCS*.

Competition might cause phylogenetic evenness in different successional states (Fig. 1), but the detection of evenness alone does not allow conclusions to be drawn about the underlying mechanisms. Especially, if phylogenetically distant species are involved in facilitation networks, a pattern of phylogenetic evenness might arise that is very similar to that generated by interspecific competition (Kunstler et al. 2012). With respect to phylogenetic clustering, Mayfield and Levine (2010) argue similarly and point out that interspecific competition might actually cause phylogenetic clustering if closely related taxa share similar traits (strong niche conservatism). Thus, any analysis of phylogenetic community structure should be accompanied by an analysis of trait distribution within the focal community and an assessment of the degree of niche conservatism.

Compared to the rich literature on changes in functional traits during succession, changes in phylogenetic structure have received little attention. Available studies mostly used chronosequence comparisons of different successional states. For instance, Whitfeld et al. (2011) reported a pattern of phylogenetic evenness in primary tropical forests of later succession stages, but phylogenetic clumping in early successional stages represented by secondary (regrowth) forests. Letcher (2010) and Letcher et al. (2012) found the strength of phylogenetic clustering and evenness changed with the age cohorts of tropical tree communities: evenness was strongest in early-succession plots of smaller trees and in late-succession plots of larger trees. Norden et al. (2012) showed a shift from phylogenetic clustering to evenness in a comparison of early and late successional lowland forest tree communities. However, no study to date has traced the change in *PCS* from the onset of natural succession within a true longitudinal study of a single plot through time.

Here, we use a unique data set on early plant succession (Zaplata et al. 2010, 2013) to assess the change in *PCS* during the first 7 years of community assembly. Previously, we used these data to detect a temporal gradient towards negative spatial species associations (Zaplata et al. 2013) and towards increased plant trait space (Ulrich et al. 2014). Assuming that the strength and direction of *PCS* depends on competitive ability, niche differentiation, and environmental conditions, we first evaluated the degree of niche conservatism among species during early plant succession. The positive phylogenetic signal obtained in important plant traits allowed us to ask whether the interplay of filtering and competitive exclusion drives later successional stages towards phylogenetic evenness, following the predictions of standard niche theory. We also assessed the degree to which the earliest successional stages are characterized by phylogenetically random community assembly, as predicted by standard neutral theory. Further, we investigated to what extent small-scale variability in soil conditions was able to overrule competition and dispersal effects and shift the community from phylogenetic evenness towards phylogenetic clustering. Hence, we aim to disentangle the influences of species richness, competition, soil conditions, and time on the phylogenetic structure during early succession.

## Materials and methods

### Study area and sampling

From 2005 to 2011, we studied the early vegetation succession in a 6 ha artificial water catchment called “Chicken Creek”, located in an open-cast lignite mine in NE Germany. Sand and loamy sand material originating from Pleistocene sediments was used for the construction of the 1–3.5 m top layer of the catchment (details in Gerwin et al. 2009).

To study the influence of initial soil conditions on plant community assembly, soil samples were taken from the upper 30 cm at the points of a regular grid (20 m × 20 m) immediately after completion of construction. Samples were analysed for pH, texture, and carbonate content (for more details and methods used, see Gerwin et al. 2011). Floristic sampling was based on a total of 426 plots of 1 m^2^, four of each being arranged around the points of these 20 m × 20 m grid points (Zaplata et al. 2013). Vegetation records started in 2005 with 360 plots; thereafter in all plots. A complete list of plant species is contained in the electronic supplementary material A. Annually (July–August), we carefully recorded all vascular plant species on the plots. For each species in each plot, we estimated the cover degree (abundance) according to a modified Londo scale (Londo 1976; 0.1: ≤0.1 %; 0.5: >0.1–0.5 %; 1: >0.5–1 %; 2: >1–2 %, in 1 % steps up to 10; 15: 13–17 %, then in 5 % steps up to 100 %). For each plot and each year, we measured the association of species occurrences and phylogenetic structure with carbonate content, fraction of sand, and pH of the soil samples from 2005. To infer the strength of niche conservatism, we used the Leda (Kleyer et al. 2008) and BioFlor (Klotz et al. 2002) databases and compiled a total of 33 plant functional, genetic, and particular morphological traits associated with assimilation, soil affinities, times of flowering and seed production, seed characteristics and output, and DNA content (Table 1, electronic supplementary material A). Ordinal variables were treated as continuous ones when they comprised at least five categories. We used the *K* (Blomberg et al. 2003) and Pagel’s *λ* (Pagel 1999) statistics to infer the degree of phylogenetic conservatism in 30 traits, while three remaining categorical traits were analysed by counting the number of parsimonious steps necessary to reconstruct the ancestral states on the internal nodes of the tree. The statistical significance of *K*, *λ*, and the number of parsimonious steps was analysed by tree randomization using the phytools R package for *K* and *λ* (Revell 2012) and Mesquite for parsimony analysis (Maddison and Maddison 2011). Because our trait matrix included a few missing values, we performed two sets of analyses: (1) missing values were replaced by arithmetic means of the respective traits, and (2) missing values were not considered. In order to check the robustness of both approaches against the assumed phylogenetic chronogram, we computed new branch lengths for the tree according to Grafen’s method (Grafen 1989) as implemented in the R package *APE* (Paradis et al. 2004). Three new trees were produced and used as a template in analysis of *K* and *λ*. Both methods returned nearly identical results, so we present the results of only the second approach, in which we did not consider missing values.

**Table 1:** Strength of phylogenetic signals (Blomberg’s *K* and Pagel’s *λ*) in 33 plant traits of all species found in the study catchment

Species	*K*	*P* (*K*)	*λ*	*P* (*λ*)	Species	*K*	*P* (*K*)	*λ*	*P* (*λ*)
*Morphological traits*					*Reproductive traits*				
Canopy height (m)	1.2	**0.001**	1	**<0.001**	Average month of seedling	0.111	**0.001**	0	1
Emergent attached to substrate	0.07	0.335	0.88	**0.001**	Duration of flowering	0.037	0.737	0.13	0.135
Leaf mass (mg)	0.11	0.147	1	**0.001**	Duration of seedling	0.042	0.517	0	1
Leaf size (mm^2^)	0.1	0.124	0.98	**0.003**	Earliest month flowering	0.082	0.018	0.8	**<0.001**
Life span	0.07	0.014	0.5	0.02	Earliest month seed shedding	0.049	0.227	0	1
Max releasing height (m)	2.11	**0.001**	1	**<0.001**	Latest month flowering	0.135	**0.001**	0.82	**<0.001**
Min releasing height (m)	0.78	**0.002**	1	**<0.001**	Latest month seed shedding	0.192	**0.001**	0.52	0.085
Specific leaf area mm^2^/mg	0.06	0.068	<0.01	1	ln (seeds per shoot)	0.063	0.059	0.63	**0.002**
Stem ascending to prostrate (%)	0.1	0.02	0.89	**<0.001**	Mean seed weight	0.132	0.078	1	**<0.001**
Stem erect (%)	0.11	**0.007**	0.96	**<0.001**	Seed bank longevity	0.06	0.075	0	1
Terminal velocity (m/s)	0.06	0.131	0.58	**<0.001**	Type of reproduction	57 steps	<0.050		
Woodiness stem	0.65	**0.001**	1	**<0.001**					
*Habitat requirements*					*Molecular traits*				
Light	0.04	0.502	0.22	0.063	Polyploidy	0.05	0.277	0.4	**0.001**
Soil fertility	0.06	0.154	<0.01	1	Chromosome number	8.62	**0.001**	1	**<0.001**
pH	0.05	0.269	<0.01	1	DNA content	0.39	**0.001**	0.75	**<0.001**
Nitrogen	0.05	0.126	<0.01	1					
Life strategy type	87 steps	*P* > 0.05							
Hemerobic level	79 steps	*P* > 0.05							

Significant signals (*P* < 0.01) are given in bold type. The number of steps in the categorical variables refer to the parsimony analysis of ancestral state reconstruction

### Data analysis

We constructed phylogenetic trees and the respective matrices of phylogenetic distances for all species using the *Phylomatic* phylogenetic database and toolkit (Webb et al. 2008), and the R package *APE* (Paradis et al. 2004). Trees generated by *Phylomatic* were based on the classification contained in *APG III* (Angiosperm Phylogeny Group 2009) and on recent sequences, which resolved the majority of polytomies. Because DNA sequence data were not available for all taxonomic levels of resolution, we assigned branch lengths to the tree with the Branch Length Adjustment (BLADJ) option in Phylocom (Webb et al. 2008), using minimum ages for genera and families and higher taxa from the molecular dating of Wikström et al. (2001). We spaced undated nodes evenly between dated ones. The complete tree is contained in the electronic supplementary material B.

To infer the change of phylogenetic species assembly during succession, we used four different approaches. First, for each 1-m^2^ study plot and each year, we calculated the nearest taxon index *NTI*, which is the standardized mean nearest neighbour phylogenetic distance (MNND) and focuses on the extent of terminal phylogenetic clustering in the co-occurrences of species (Webb et al. 2002). Second, we performed analogous analyses using another index, the net relatedness index NRI, which returns the standardized mean phylogenetic distance (MPD) (Webb et al. 2002). Both *NTI* and *NRI* increase with increasing phylogenetic clustering and decrease with increasing phylogenetic evenness. Third, we used linear phylogenetic eigenvector regression *PER* (Diniz-Filho et al. 1998, 2012). This technique allowed us to infer the part of variance in species abundance (quantified by the associated *r*_PER_^2^ statistics of goodness-of-fit of the multiple regression) of each plot explained by phylogenetic distances. We used the phylogenetic information contained in the first four eigenvectors of the phylogenetic distance matrix that explained 97 % of total variance (electronic supplementary material B). Species richness, abundance, *NTI*, *NRI*, and *r*_PER_^2^ showed weak spatial autocorrelation only (Moran’s *I* < 0.05). However, to account for the intermediate degree of spatial autocorrelation of soil variables, we used simultaneous autoregression (Rangel et al. 2010) to relate species richness, total abundance, *NTI*, *NRI*, and *r*_PER_^2^ to soil properties. Autoregression analysis was done with SAM 4.0 (Rangel et al. 2010) using default settings. During subsequent study years samples were taken on the same plots, as is typical for any real-time series. Consequently, temporal autocorrelation might influence our autoregression results by artificially inflating the degrees of freedom. To account for this type of temporal pseudo-replication, we restricted the degrees of freedom in the parametric *t* tests for all single predictors to the total number of plots (426) and did not use the total number of data points (2,271). This procedure should minimize the possible bias when estimating the respective significance levels, and thus should avoid negative effects of pseudo-replicated data.

Because common metrics of phylogenetic community structure do not account for multiple patterns of species co-occurrence (Gotelli and Ulrich 2012), we used the method proposed by Ulrich et al. (2012) that links phylogenetic community structure to pair-wise patterns of species co-occurrences in the underlying species × plot matrix. For this, the phylogenetic distances Δ*P* of pairs of species occurring at all pairs of plots are calculated with respect to three distinct patterns: (1) aggregated species co-occurrence in species × sites submatrices of the form $$\left( {\begin{array}{lll} 1 & \cdots & 1 \\ \vdots & \ddots & \vdots \\ 1 & \cdots & 1 \\ \end{array} } \right)$$, (2) segregated (checkerboard) 2 × 2 submatrices of the form $$\left( {\begin{array}{lll} 0 & \cdots & 1 \\ \vdots & \ddots & \vdots \\ 1 & \cdots & 0 \\ \end{array} } \right)$$ and (3) togetherness submatrices of the form $$\left( {\begin{array}{lll} 1 & \cdots & 0 \\ \vdots & \ddots & \vdots \\ 1 & \cdots & 0 \\ \end{array} } \right)$$ (cf. Ulrich and Gotelli 2013). Therefore, this co-occurrence (*CO*) method explicitly considers matrix-wide differences in the pattern of species co-occurrences (reviewed in Gotelli and Ulrich 2012) to infer the strength of the *PCS*. The aggregated and segregated spatial patterns are typically attributed to positive interactions and reciprocal competitive exclusion, respectively, whereas the togetherness pattern indicates filtering and similar habitat requirements (cf. Stone and Roberts 1992; Ulrich et al. 2012). Low scores of Δ*P* in combination with aggregated and togetherness patterns indicate phylogenetic clumping and spatial aggregation (Ulrich et al. 2012).

All four methods (*NTI*, *NRI*, *PER*, *CO*) to quantify phylogenetic community structure rely on a particular null model to which raw scores are compared. *NTI* and *NRI* are in fact the negative standardized effect sizes (*SES*) of *MPD* and *MNND* with respect to the predefined null model distribution. SES scores should have values below −1.96 and above +1.96 at the two-sided 5 % error level under the assumption that the respective null distribution is approximately normal. At higher species richness, the probability of including small and distantly related taxa in the community increases and consequently *MPD* and *MNND* decrease. Both metrics are also sensitive to phylogenetic autocorrelation of species abundances or numbers of occurrences (Hardy 2008). To eliminate this bias in statistical inference, null or neutral models have to be applied (Gotelli and Ulrich 2012).

The question of which null model is adequate in terms of the above consideration is crucial in any phylogenetic analysis. Results heavily depend on proper null model choice (Gotelli and Ulrich 2012; Ulrich and Gotelli 2013). In the present case, the equal size of our sample plots means that a suitable null hypothesis is the random appearance of individuals on single plots constrained only by differences in regional species abundance and plot quality. The first constraint might be approximated by the observed abundance distribution on all plots in a given year, and the second constraint approximated by the observed annual total number of individuals per plot. Therefore, we take advantage of our quantitative data structure and apply an abundance-based null model. Recently, Ulrich and Gotelli (2010) showed that such null models, which are based on the resampling of individuals instead of species, are indeed sensitive tools for inferring matrix patterns. In accordance with our null assumptions, we applied the *AA* null model of Ulrich and Gotelli (2010) that resamples the matrix proportional to both row (species abundances) and column (plot abundances) marginal distributions. Note that such a null model mimics a mechanistic neutral model (Hubbell 2001; Rosindell et al. 2012) without speciation and dispersal limitation, in which the probabilities of occurrence in single cells depend only on the relative abundance distribution of each species (Ulrich and Zalewski 2007; Zillio and Condit 2007). These assumptions seem appropriate in the present case. Note also that a neutral colonization process cannot be tested with this null model. Null expectations and standard deviations of the *AA* null distributions were in all cases based on 200 randomizations. All null model calculations were made with the Fortran software application *Niche* (Ulrich et al. 2012).

## Results

Any analysis of *PCS* relies on the assumption of niche conservatism. Blomberg’s *K* and Pagel’s *λ* identified the majority of morphological and molecular traits as being phylogenetically conserved (Table 1). In contrast, only some of the reproductive traits and none of the traits related to habitat requirements and life history showed a significant, albeit weak, phylogenetic signal (Table 1).

Species richness and total cover increased during succession (Table 2, Zaplata et al. 2013). There was a strong dependence on initial soil characteristics: total % cover decreased with increasing carbonate content and the fraction of sand (Table 2). pH is partly buffered by the carbonate content, and both variables were moderately correlated (*r* = 0.24, *P* < 0.01). Species richness correlated positively with pH (Table 2).

**Table 2:** The dependence of species richness, total plant cover, and four metrics of phylogenetic community structure on study year, species richness, cover, and soil properties

Variables	Species	Cover	*r*_PER_^2^	SES (*r*_PER_^2^)	NTI	NRI
Study year	0.61***	0.49***	0.12***	−0.46***	−0.35***	−0.36***
Species	–	0.30***	0.07**	0.08*	0.59***	0.39***
Cover	0.25***	–	0.53***	0.28***	−0.36***	−0.38***
CaCO_3_	0.01	−0.07***	−0.06**	−0.05*	0.09***	0.08***
Sand	−0.01	−0.05**	−0.07***	−0.07**	0.01	0.02
pH	0.04**	0.03	−0.01	0.01	−0.01	0.02
*r* ^2^ (predictors)	0.66	0.56	0.47	0.1	0.18	0.2
*P* (model)	<0.001	<0.001	<0.001	0.001	<0.001	<0.001

Given are standardized values of simultaneous autoregression. Parametric significances (*N* = 426): * *P* < 0.01, ** *P* < 0.001, *** *P* < 0.0001

**Fig. 2:**
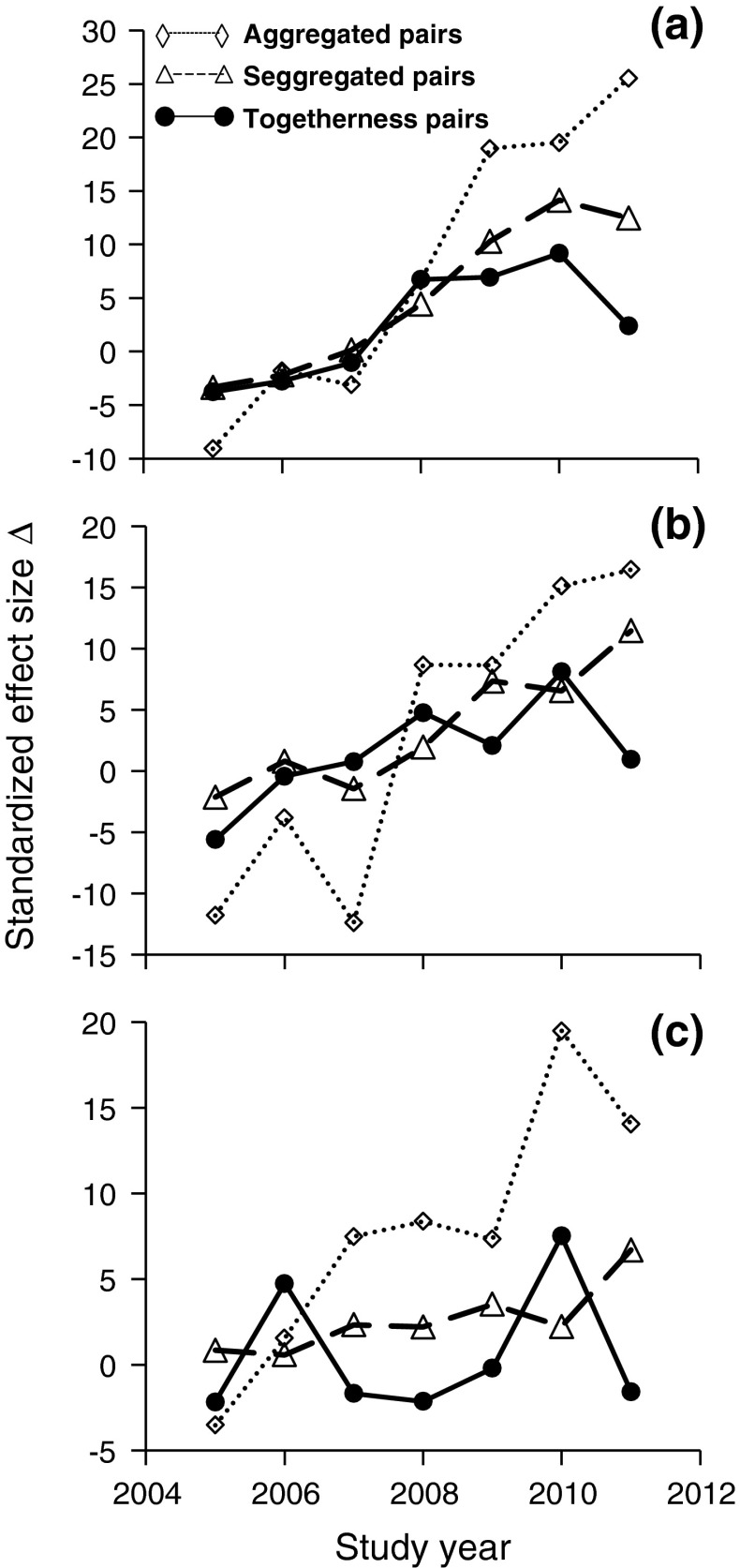
Temporal changes of standardized effect sizes (*AA* null model) of the differences Δ in **a** plot carbonate content, **b** fraction of sand, and **c** pH between plots where species pairs occurred aggregated, segregated, and in a togetherness pattern

The *CO* analysis revealed strong reactions in the pattern of species co-occurrences with respect to differences in soil quality (Fig. 2). In early succession, pairs of plots in which two species co-occurred (aggregation and togetherness) were, on average, less different in soil properties than expected by the null model. In later samples, this pattern reversed and plots of co-occurring species differed more in soil properties than expected (Fig. 2). This pattern was most pronounced for carbonate content and the fraction of sand (Fig. 2a, b). In the case of segregated spatial species co-occurrences (checkerboard), we found a random pattern in the first 3 years of succession and again a larger difference in soil properties for spatially segregated species than expected by chance (Fig. 2).

**Fig. 3:**
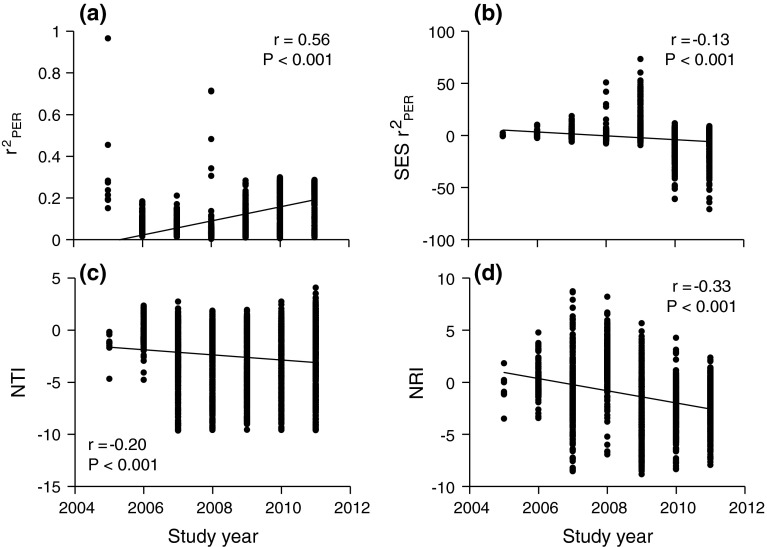
Temporal changes of **a** explained variance of the phylogenetic eigenvector regression *r*_PER_^2^, **b** respective standardized effect size *SES* *r*_PER_^2^, **c** *NTI*, and **d** *NRI*

The *r*_PER_^2^ of the *PCS* explained between 0.2 and 96 % of the variance in species abundances but, for most species, <20 % of the variance was explained (Fig. 3a). One-way ANOVA revealed significant differences between study years in the strength of the *PCS* (*F* = 153.0, *P* < 0.001): the year 2005 lacked a significant signal, 2006–2009 had a higher signal than expected by chance, and 2010, 2011 exhibited a lower signal than expected by chance (Fig. 3b). The raw *PCS* *r*_PER_^2^ increased significantly (*r* = 0.56, *P* < 0.001) during succession (Fig. 3a; Table 2), but it decreased (*r* = −0.2, *P* < 0.001) in comparison to the null model expectation (Fig. 3b; Table 2). *NTI* and *NRI* significantly decreased during succession, indicating an increase in phylogenetic evenness (Fig. 3c, d; *P* < 0.001). This trend held after accounting for richness and abundance differences (Table 2).

**Fig. 4:**
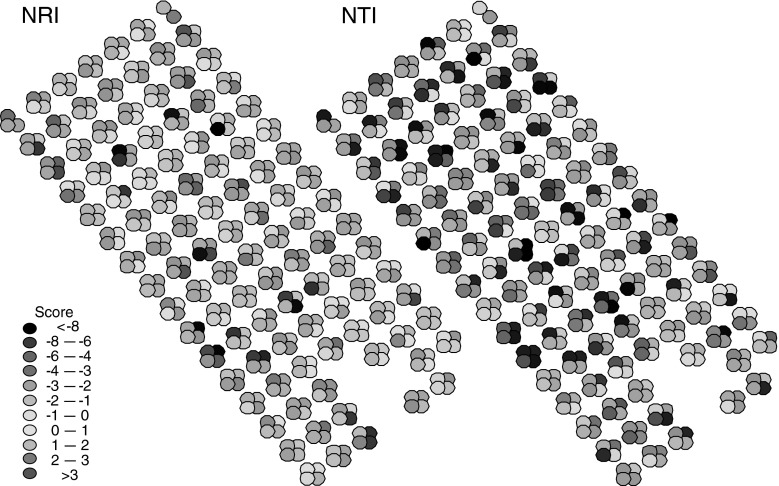
Small scale variability of *NRI* and *NTI* (426 1-m^2^ plots in 2011) in the study catchment. Colours reflect the tendencies towards phylogenetic evenness (*blue parts*) or clustering (*red parts*) (colour figure online)

**Fig. 5:**
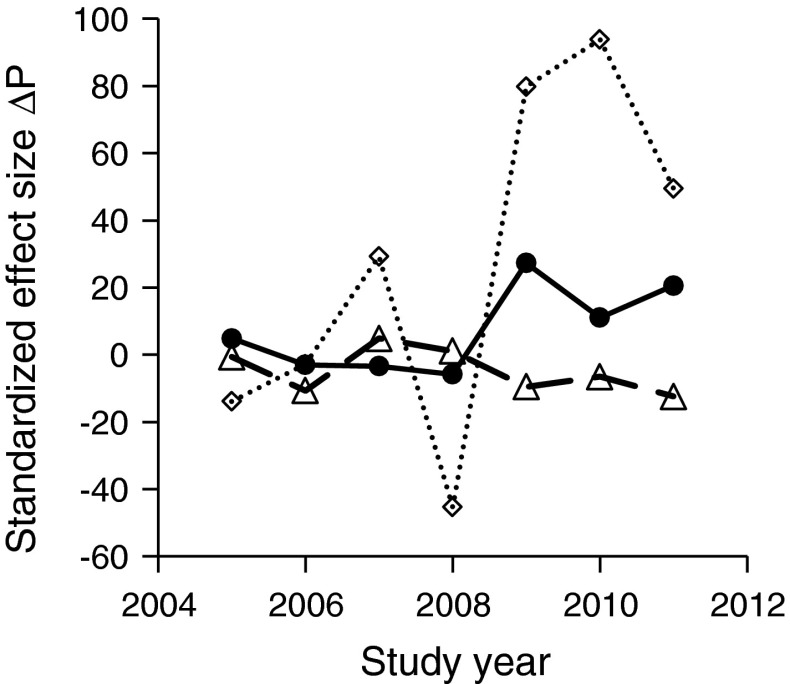
Temporal changes of standardized effect sizes of the phylogenetic differences Δ*P* between all species pairs of clumped occurrences (*dotted line*), togetherness (*full line*), and checkerboard occurrences (*broken line*)

Soil properties mediated the strength and direction of *PCS* (Table 2). *r*_PER_^2^ decreased significantly with increasing carbonate content and sand (Table 2). Spatially explicit maps of *NTI* and *NRI* revealed small-scale variation in phylogenetic evenness and clustering within the study area (Fig. 4), with moderate covariation in the scores of *NTI* and *NRI* (Pearson correlation *r* = 0.62, *P* < 0.001). Both *NTI* and *NRI* significantly increased with carbonate content (Table 2). The co-occurrence analyses (Fig. 5) revealed an initial random pattern up to 2008, and from 2009 to 2011 an increase in average phylogenetic distance with respect to species pairs that co-occur at two sites. Phylogenetic distance among checkerboard pairs decreased from 2007 to 2011 (Fig. 5). Consequently, starting from 2009, there was a strong phylogenetic evenness of co-occurring species and, respectively, a phylogenetic clustering of species that did not co-occur (all SES > |5.0|); Fig. 5).

## Discussion

Phylogeny does not affect community structure directly. Phylogeny is mediated by traits that determine whether species are able to colonize a site, and whether colonists will compete, positively interact, or be unaffected by each other (Webb et al. 2002). Thus, any phylogenetic analysis should be accompanied by a respective trait analysis (Freckleton et al. 2002). In the present case, we found the majority of morphological and molecular traits to contain a significant albeit often weak phylogenetic signal, i.e., to be non-randomly distributed across the phylogenetic tree. Significant scores of *K* and *λ* occurred also in seed number and weight as well as in those traits related to duration of flowering (Table 1). No trait related to life history and habitat requirements, particularly soil conditions, appeared to be phylogenetically conserved in this early successional plant community. This result contrasts with the finding of Prinzing et al. (2001), who reported on a strong phylogenetic signal of habitat, life history and niche factors in European plants, but is in line with the study of Silvertown et al. (2006), who did not find significant phylogenetic signals in meadow plant communities. These findings suggest that the strength of phylogenetic signals in plants increases with spatial scale, and therefore also with the size of the species pool considered (Silvertown et al. 2006). This scale dependence might be triggered by local environmental filters. The species of small local communities have already passed the abiotic, particularly soil-mediated, filters and are thus phylogenetically and ecologically more closely related than expected from the species pool. Therefore, tests for phylogenetic signals within local communities will often return a negative result.

A comprehensive understanding of the role of *PCS* for community composition should also include an understanding of which environmental factors control species occurrences and community composition. In the present case, we tested for three important soil variables (carbonate content, fraction of sandy material, and pH), and found particularly positive correlations of species richness with pH and negative correlations of cover with carbonate content and sand (Table 2). Our results did not change after accounting for spatial autocorrelation of the predictor variables (Table 2), indicating that the results are robust with respect to the observed spatial distribution of soil characteristics.

We found a significant temporal shift in species responses to the soil variables (Fig. 2). In the first 2 years of succession, plots of co-occurring species (clumped and togetherness pattern) were more similar in carbonate content (Fig. 2a) and fraction of sand (Fig 2b) than expected by chance, while in the later years of succession this pattern reversed. We explain this finding by the initial effect of small-scale habitat filtering. Habitats undergoing primary succession are often characterized by strong abiotic filters (e.g., mineral availability, drought) (Walker et al. 2006) and the initial spatial partitioning of colonizing plant species is thought to be controlled by physiological tolerances to abiotic stress (Chapin 1993). In the present case, abiotic mineral crusts occurred during the first years on the soil surface (Fischer et al. 2010) and might have acted as filters for seed germination and plant establishment, either through the exclusion of certain traits or through the temporarily restricted access of certain colonizers. Both mechanisms might cause a co-occurrence of phylogenetically related taxa with similar ecological features. However, the initial soil-mediated co-occurrences might also be an indication for a marginal contamination with diaspores during catchment construction (Zaplata et al. 2010, 2011). This seed bank effect is a neglected factor in studies of *PCS* and species co-occurrence and deserves further attention.

In the later successional states those species pairs that did not co-occur (≙ checkerboard pattern) were found significantly more often in plots that differed in soil properties (Fig. 2). This finding indicates a temporal trend towards the spatial segregation of species according to soil properties. Similarly, Biber et al. (2013) reported a significantly positive correlation between the species richness of N-fixing Fabaceae and the soil gravel content. Such findings contradict niche theories, which expect co-occurrences of functionally different species to increase synergistic effects (Silva and Batalha 2010) and to reduce possible interspecific competition (Götzenberger et al. 2011). Our results, however, indicate that functional differences related to substrate properties might dominate over competitive species interactions, leading to the spatial segregation of species with different microhabitat requirements. This finding is corroborated by the strong tendency towards decreasing demographic stochasticity and increasing degree of species spatial segregation (Zaplata et al. 2013) during the catchment succession.

Given the tendency towards niche conservatism (Table 1), increasing species spatial segregation (Zaplata et al. 2013), and increasing trait space (Ulrich et al. 2014), we expected to see directional shifts in phylogenetic community composition depending on species richness and soil properties. Indeed, the mean phylogenetic and nearest taxon indices decreased during succession and the phylogenetic community structure changed from an initially random pattern towards significant phylogenetic evenness (Fig. 3c, d). Importantly, this finding was stable after accounting for differences in species richness, cover, and soil properties (Table 2). Similar results have been obtained by Whitfeld et al. (2011) and Norden et al. (2012) in their comparisons of differently aged tropical tree communities. The present study is apparently the first to demonstrate this trend in early plant succession and for temperate regions.

According to Mayfield and Levine (2010), interspecific competition does not necessarily cause phylogenetic evenness (Fig. 1). The outcome depends on the interplay between niche conservatism and filtering processes and on the degree to which traits that are involved in interspecific competition are phylogenetically conserved. In turn, the detection of phylogenetic evenness does not necessarily indicate a major influence of competition. Other species interactions like facilitation (Fig. 1) might also cause phylogenetic evenness (Sargent and Ackerly 2008). A way to overcome these difficulties in interpretation and to infer causal connections is the study of multiple patterns of species co-occurrences with respect to *PCS* (Ulrich et al. 2012; Riedinger et al. 2013). This method enables the identification of different trends in *PCS* within the same community, depending on the spatial arrangement of species occurrences. We detected a significant temporal increase in the average phylogenetic distance of species, which jointly occurred and/or jointly were absent at a pair of sites (aggregated and togetherness co-occurrences) (Fig. 5). While joint occurrences do not point to competitive exclusion, the significant signal of phylogenetic evenness indicates synergistic effects of distantly related species. These species potentially differ in important traits (Table 1). In turn, spatially segregated species were significantly phylogenetically clustered after the first years of succession (Fig. 5). Closely related species hence had a tendency of spatial segregation. Taken together, both findings indicate that competitive effects shaped the observed spatial and phylogenetic distribution of species. Further, our co-occurrence analysis identified multiple types of species interactions that lead to phylogenetic evenness. These include interspecific competition among some of the species and synergistic interactions among others. In early plant succession, facilitation is of major importance (e.g., Connell and Slatyer 1977; Moeller 2004) and may well be the deciding factor in the successional series studied, although we did not particularly address this facilitation aspect here. Previously, Valiente-Banuet and Verdu (2007) detected a positive correlation of the degree of facilitation and phylogenetic evenness in plant communities.

A major finding of the present contribution is that the pattern of small-scale *PCS* depends on environmental factors, particularly on carbonate content. Despite the overall trend towards phylogenetic evenness (Table 2; Fig. 3c, d), we found a highly significant tendency towards phylogenetic clustering (positive *NTI* and *NRI*) on plots of higher carbonate content (Table 2; Fig. 4). Apparently, trends in the phylogenetic community composition at Chicken Creek were mediated by the initial soil conditions. In the present case, the whole study area was initially rather uniform concerning geomorphology, age, and abandonment. There were slight differences in soil chemistry that divided the catchment area into two parts, a southwestern and a north-eastern part, which differed in average fractions of silt and clay, and in nitrogen, organic, and inorganic carbon content (Zaplata et al. 2010, 2013). Our phylogenetic analysis partly recovered these catchment compartments and additionally revealed the small-scale variability in *PCS* (Fig. 4). Sargent and Ackerly (2008) reported similar environmental pressures towards either phylogenetic clustering or evenness in plant-pollinator communities. Therefore, within the biotic interaction-paradigm, successional communities can evolve towards both directions. Any prediction about the final outcome seems only possible after detailed modelling of species traits and accompanying abiotic factors. In any case, the strength of the *PCS* should increase during primary succession.

Our study does not point to neutral community assembly at later successional states. Under a neutral scenario of community development, we expected *NTI* and *NRI* scores not to differ from random expectation at all successional states and *PCS* not to show a directional trend (Fig. 1; Hardy et al. 2012; Münkemüller et al. 2012). This was not the case. However, a local *PCS* can even pertain under neutral colonization if the structure of the regional species pool is appropriately structured (Hardy et al. 2012) or if the initial seed bank was phylogenetically structured, for instance due to differential diaspore survival probabilities (Leishman 2001). Yet in this case, we would expect to see either a constant signal in time or a decrease in strength, contrary to our findings. Furthermore, the observed small-scale signal variability in *NTI* and *NRI* (Fig. 4) is not in accordance with simple ecological drift.

In the earliest states of succession, which we suggest to be the first 2 years, we cannot exclude a largely neutral community assembly. For these years, we did not find statistically significant *NRI* and *NTI* scores (Fig. 3c, d), and the strength of the *PCS* did not differ from random expectation (Fig. 3a, b). Previously, Zaplata et al. (2013) found a similar shift from random patterns of species co-occurrences (2005, 2006) towards distinct species spatial segregation (from 2007 on). We note that it is not possible to positively test for neutrality. Our null model already incorporates neutral features and recent theoretical work (Chisholm and Pacala 2010; Clark 2012) highlights that niche and neutral models might give similar predictions on community structure. Indeed, Gotelli and Ulrich (2012) and Rosindell et al. (2012) stress that the main function of neutral modelling is to provide appropriate null assumptions against which observed patterns can be tested. This makes neutrality an ecological interaction-free statistical standard rather than a predictive theory.

An important aspect of the present study is the use of small-scale plots within one locality. This approach enabled us not only to trace the development of the plant community in the whole catchment, but also to disentangle its internal fine-scale structure and to apply metrics of *PCS* and co-occurrence at the level of pair-wise species associations. Such species-pair analyses are rarely undertaken in phylogenetic research (Ulrich et al. 2012) but are potentially powerful tools to infer the precise mechanisms that lead to certain observed patterns. Phylogenetic analysis has to include comparisons of environmental needs, functional traits and patterns of co-occurrence at the species level. Furthermore, having shown the fine scale character of *PCS* and of functional traits, we need a precise modelling of the relationships between the distribution of functional traits and the degree of environmental filtering. This task demands data on the small-scale spatial distribution of important environmental filters (Wisz et al. 2012) and an appropriate spatially explicit high-resolution monitoring of plants, traits, and environmental correlates.

## Electronic supplementary material

Below is the link to the electronic supplementary material.

Electronic supplementary material A (PDF 853 kb)

Electronic supplementary material B (PDF 2635 kb)
