# Supplementary material for: Small-scale spatial variability in phylogenetic community structure during early plant succession depends on soil properties
Source: Oecologia. 2014 May 9;175(3):985–95. doi: 10.1007/s00442-014-2954-2 (PMC4059990; doi:10.1007/s00442-014-2954-2)
Supplement: Supplementary file 1 — Electronic supplementary material A (PDF 853 kb) [file 442_2014_2954_MOESM1_ESM.pdf]

Small scale spatial variability in phylogenetic signals during early plant succession depends on soil properties

Oecologia

Werner Ulrich[1], Marcin Piwczyński, Markus Klemens Zaplata, Susanne Winter, Wolfgang Schaaf, Anton Fischer
[1]Chair of Ecology and Biogeography, Nicolaus Copernicus University in Torun, Lwowska 1, PL 87-100 Toruń, Poland.
Phone: 0048 56 611 2649, e-mail: *ulrichw@umk.pl*

Database containing a total of 33 plant functional, genetic and morphological traits complied from the Leda (Kleyer et al. 2008) and BioFlor (Klotz et al. 2002) trait bases.

Species	Leaf mass [mg]	Leaf size [mm^2]	Life span	Min releasing height [m]	Max releasing height [m]
Vicia_hirsuta	NA	NA	1	0.15	0.6
Epilobium_hirsutum	NA	NA	5	0.8	1.5
Polygonum_persicaria	NA	NA	1	0.1	0.8
Sisymbrium_officinale	NA	NA	0.5	0.3	0.6
Erigeron_annuus	200.74	859	0.5	0.5	1
Epilobium_tetragonum	NA	NA	5	0.3	1
Poa_palustris	1.3	55	5	0.3	1
Tragopogon_dubius	12	321.5	1	0.3	0.8
Viola_canina_agg.	NA	NA	NA	0.05	0.15
Agrostis_vinealis	1.41	22.75	5	0.2	0.4
Myosotis	NA	NA	NA	NA	NA
Tanacetum_vulgare	NA	NA	5	0.6	1.2
Phalaris_arundinacea	NA	NA	5	0.8	2.5
Leucanthemum_vulgare_agg.	NA	NA	5	0.4	0.5
Daucus_carota	NA	NA	1	0.3	1
Echium_vulgare	NA	NA	2	0.25	1
Phragmites_australis	NA	NA	5	1	4
Jasione_montana	0.95	36.5	2	0.1	0.45
Viola_arvensis	NA	NA	1	0.05	0.2
Trifolium_arvense	NA	NA	1	0.08	0.3
Trifolium_pratense	39.91	NA	5	0.15	0.65
Hypericum_perforatum	NA	NA	5	0.3	0.9
Rumex_acetosa	11.2	360.43	5	0.1	1
Holcus_lanatus	NA	NA	5	0.2	1
Veronica_officinalis	NA	NA	5	0.05	0.4
Helictotrichon_pubescens	42.7	1004	5	0.3	1.05
Luzula_multiflora_subsp._multiflora	NA	NA	5	0.1	0.5
Potentilla_argentea	NA	NA	5	0.16	0.28
Medicago_lupulina	14.35	407	1	0.15	0.6
Lotus_corniculatus_agg.	NA	NA	5	0.05	1.7
Rumex_acetosella_var._tenuifolius	NA	NA	5	0.1	0.54
Juncus_articulatus	NA	NA	5	0.2	0.5
Tussilago_farfara	NA	NA	5	0.07	0.3
Picris_hieracioides	56.12	874	2	0.3	0.96
Festuca_ovina_agg.	1.59	23.8	2	0.14	0.65
Trifolium_campestre	NA	NA	1	0.15	0.3
Trifolium_dubium_agg.	NA	NA	1	0.1	0.3
Viola_tricolor_agg.	NA	NA	1	0.1	0.4
Crataegus_monogyna	NA	NA	5	2.5	10
Elymus_repens	128.24	3234	5	1.2	1.5
Agrostis_capillaris	NA	NA	5	0.2	0.83
Juncus_bufonius_agg.	NA	NA	1	0.1	0.25
Senecio_vulgaris	NA	NA	1	0.1	0.3
Rubus_fruticosus_agg.	NA	NA	5	0.1	1.8
Rubus_idaeus	118.07	NA	5	0.6	2
Moos_folios	NA	NA	NA	NA	NA
Moos_thallos	NA	NA	NA	NA	NA
Hieracium_umbellatum	51.9	1145.5	5	0.1	1.03
Salix_caprea	NA	NA	5	3	8
Epilobium_ciliatum	NA	NA	5	NA	NA
Robinia_pseudoacacia	NA	NA	5	15	25
Ulmus	126.93	4351	5	20	20
Cirsium_arvense	NA	NA	5	0.6	1.2
Torilis_japonica_agg.	NA	NA	0.5	0.3	1.2
Silene_alba_subsp._alba	NA	NA	NA	NA	NA
Carex_hirta	NA	NA	5	0.1	0.8
Rumex_thyrsiflorus	168.09	2871	NA	0.3	1.2

Calamagrostis_epigejos	NA	NA	2	0.6	1.5
Arctium_minus_agg.	NA	NA	2	0.5	1
Hippophae_rhamnoides	NA	NA	5	5	5
Lupinus_polyphyllus	1186.25	19778.5	5	0.74	1.5
Betula_pendula	NA	NA	5	10	25
Verbascum	NA	NA	2	0.5	1.2
Malus_domestica	NA	NA	5	5	10
Populus_tremula	178.47	2556.38	5	10	25
Artemisia_vulgaris_agg.	NA	NA	5	0.6	2.5
Dactylis_glomerata_agg.	0.01	167	5	0.3	1.62
Pinus_sylvestris	10.81	28.5	5	15	50
Sinapis_arvensis	NA	NA	1	0.3	0.6
Lupinus_luteus	188.65	4197.74	1	0.3	0.6
Echinochloa_crus-galli	61.54	1699.25	1	0.3	0.6
Convolvulus_arvensis	32.15	730	5	0.2	0.8
Vicia_villosa	NA	NA	0.5	0.3	1.2
Lactuca_serriola	NA	NA	1	0.6	1.2
Sonchus_asper	NA	NA	1	0.2	1.5
Chenopodium_album_agg.	NA	NA	1	0.02	3
Crepis_foetida	NA	NA	NA	0.15	0.3
Vicia_angustifolia	NA	NA	1	0.3	0.8
Papaver_rhoeas	NA	NA	1	0.3	0.9
Cirsium_vulgare	270.89	4421	2	0.3	2
Tripleurospermum_maritimum_agg.	8.63	198.75	1	0.35	0.35
Cerastium_holosteoides	0.5	22.5	1	0.05	0.5
Oenothera_parviflora_agg.	31.21	437.25	5	0.2	2
Sisymbrium_altissimum	212.42	4745.64	1	0.3	1.09
Chenopodium_polyspermum	15.23	NA	1	0.15	0.6
Conyza_canadensis	NA	NA	1	0.2	1
Sonchus_arvensis_agg.	NA	NA	5	0.5	1.5
Papaver_dubium	NA	NA	1	0.3	0.98
Hordeum_vulgare	156.41	3650.5	0.5	0.6	1.2
Apera_spica-venti	NA	NA	1	0.3	1
Bromus_hordeaceus_agg.	0	59	0.5	0.05	0.8
Salix_viminalis	32.88	349.75	NA	3	5
Corynephorus_canescens	1.29	33.5	5	0.15	0.3
Danthonia_decumbens	NA	NA	5	0.15	0.45
Chondrilla_juncea	23.62	301.25	5	0.3	1
Carex_spicata	12.04	299	5	0.2	0.6
Carex_arenaria_agg.	69.7	944	5	0.15	0.6
Melica_nutans_agg.	NA	NA	5	0.3	1.07
Typha	NA	NA	5	1	2
Artemisia_campestris_agg.	13	206.5	2	0.3	0.6
Festuca_gigantea	NA	NA	5	0.6	1.5
Achillea_pannonica	82.33	567.84	5	0.2	0.8
Hieracium_piloselloides	0.22	602.75	5	0.2	0.8
Crepis_capillaris	NA	NA	0.5	0.15	0.6
Carex_ericetorum	NA	NA	5	0.1	0.3
Helichrysum_arenarium	20.45	404.5	5	0.1	0.3
Geum_urbanum	NA	NA	2	0.3	1.2
Fragaria_vesca	NA	NA	2	0.05	0.3
Poa_compressa	NA	NA	5	0.2	0.8
Gnaphalium_sylvaticum	NA	NA	5	0.1	0.6
Berteroa_incana	6.52	141.25	0.5	0.3	0.65
Ajuga_genevensis	17.99	365.5	5	0.07	0.3
Centaurea_stoebe	35.13	538.91	NA	NA	NA
Moehringia_trinervia	NA	NA	1	0.05	0.3
Crepis_tectorum	NA	NA	0.5	0.1	0.6
Hieracium_bauhini	NA	NA	NA	NA	NA
Setaria_viridis	44.87	1354.5	1	0.05	1.2
Arenaria_serpyllifolia_agg.	0.24	3.55	0.5	0.03	0.3
Digitaria_sanguinalis	8.63	290.25	1	0.15	0.6
Senecio_vernalis	52.13	1464.85	1	0.15	0.45
Setaria_pumila	75.09	1890.75	1	0.1	0.69
Herniaria_glabra	0.25	7.55	5	0.05	0.3
Digitaria_ischaemum	0.65	109.88	1	0.03	0.45
Erodium_cicutarium_agg.	20.5	784	0.5	NA	NA
Scleranthus_annuus_agg.	0.89	9.94	0.5	0.02	0.2
Scleranthus_perennis	0.25	4.62	5	0.05	0.2
Bromus_tectorum	19.81	724	0.5	0.1	0.45
Vicia_tetrasperma_agg.	NA	NA	1	0.15	0.6
Matricaria_recutita	NA	NA	1	0.15	0.4
Centaurium_erythraea	5.7	NA	2	0.02	0.5
Ornithopus_perpusillus	NA	NA	0.5	0.05	0.39

Salsola_kali_subsp._ruthenica	6.84	62.5	0.5	0.25	0.6
Senecio_viscosus	NA	NA	0.5	0.15	0.5
Filago_arvensis	1.95	77	1	0.1	0.35
Filago_minima	0.2	6.64	1	0.05	0.2
Petrorhagia_prolifera	3.35	58.25	0.5	0.15	0.45
Cerastium_pumilum_agg.	0.76	21.25	0.5	NA	NA
Hordeum_jubatum	NA	NA	5	0.2	0.6
Brachypodium_sylvaticum	NA	NA	5	0.6	1.2
Genista_pilosa	1.62	20.6	5	0.15	0.3
Leontodon_taraxacoides	24.27	400.25	NA	NA	NA
Plantago_lanceolata	60.97	NA	5	0.05	1.3
Linaria_vulgaris	NA	NA	5	0.2	0.75
Veronica_chamaedrys_agg.	NA	NA	5	0.15	0.4
Solidago_canadensis	NA	NA	5	0.5	2.5
Eupatorium_cannabinum	NA	NA	5	0.5	1.5
Rumex_crispus	NA	NA	1	0.3	1.6
Festuca_rubra_agg.	NA	NA	2	0.4	0.72
Deschampsia_cespitosa_agg.	14.68	165.7	5	0.25	1.36
Equisetum_arvense	178.65	167	5	0.15	0.5
Hieracium_pilosella	NA	NA	5	0.05	0.35
Taraxacum_officinale_agg.	NA	NA	NA	0,05	0,3
Leontodon_autumnalis	3.95	148.58	5	0.07	0.45
Holcus_mollis	NA	NA	5	0.3	0.8
Lolium_perenne	NA	NA	1	0.1	0.86
Poa_pratensis_agg.	NA	NA	5	0.2	0.9
Prunella_vulgaris	7.03	NA	2	0.05	0.36
Hypochaeris_radicata	NA	NA	5	0.15	0.6
Trifolium_repens	6.66	NA	5	0.15	0.5
Solanum_nigrum	NA	NA	1	0.1	0.8
Plantago_major	NA	NA	1	0.06	0.4
Sagina_procumbens	NA	NA	1	0.02	0.15
Agrostis_stolonifera_agg.	NA	NA	5	0.1	0.7
Plantago_major_subsp._intermedia	23.83	388	1	0.05	0.4
Polygonum_aviculare_agg.	NA	NA	1	0.05	0.5
Poa_annua	NA	NA	1	0.02	0.3
Lepidium_ruderale	NA	NA	0.5	0.1	0.3
Spergularia_rubra	NA	NA	1	0.04	0.25

Species	Stem erect %	Stem ascending to prostrate %	Emergents attached to substrate	Terminal velocity m/s	Woodiness Stem	Light	Soil fertility	pH	Nitrogen
Vicia_hirsuta	0	0	0	4.72	0	7	4	0	4
Epilobium_hirsutum	100	0	0	0.2	0	7	8	8	8
Polygonum_persicaria	37	62	0	3.46	0	6	5	7	7
Sisymbrium_officinale	100	0	0	NA	0	8	4	0	7
Erigeron_annuus	100	0	0	NA	0	7	6	0	8
Epilobium_tetragonum	100	0	0	0.14	0	7	8	6	5
Poa_palustris	75	25	0	1.43	0	7	9	8	7
Tragopogon_dubius	100	0	0	0.55	0	8	4	8	4
Viola_canina_agg.	66	33	0	NA	0	7	4	3	2
Agrostis_vinealis	50	50	0	1.59	0	9	9	3	2
Myosotis	NA	NA	NA	NA	NA	6	5	0	6
Tanacetum_vulgare	100	0	0	NA	0	8	5	8	5
Phalaris_arundinacea	100	0	0	2.51	0	7	8	7	7
Leucanthemum_vulgare_agg.	100	0	0	2.42	0	7	4	0	3
Daucus_carota	100	0	0	2.21	0	8	4	0	4
Echium_vulgare	100	0	0	NA	0	9	4	8	4
Phragmites_australis	22	11	44	0.19	0	7	10	7	7
Jasione_montana	60	40	0	1.01	0	7	3	3	2
Viola_arvensis	60	40	0	NA	0	6	0	0	0
Trifolium_arvense	66	33	0	1.53	0	8	3	2	1
Trifolium_pratense	57	42	0	3.42	0	7	5	0	0
Hypericum_perforatum	100	0	0	1.92	0.21	7	4	6	4
Rumex_acetosa	100	0	0	1.75	0	8	0	0	6
Holcus_lanatus	75	25	0	1.54	0	7	6	0	5
Veronica_officinalis	20	80	0	2.08	0	6	4	3	4
Helictotrichon_pubescens	100	0	0	2.78	0	5	3	0	4
Luzula_multiflora_subsp._multiflora	100	0	0	2.2	0	7	5	5	3
Potentilla_argentea	0	100	0	2.43	0	9	2	3	1
Medicago_lupulina	75	42	0	3.12	0	7	4	8	0
Lotus_corniculatus_agg.	36	63	0	3.68	0.21	7	4	7	3
Rumex_acetosella_var._tenuifolius	100	0	0	2.96	NA	9	3	2	1

Juncus_articulatus	50	25	25	1.31	0	9	8	5	3
Tussilago_farfara	100	0	0	0.21	0	8	6	8	0
Picris_hieracioides	100	0	0	0.51	0	8	4	8	4
Festuca_ovina_agg.	100	0	0	2.86	0	7	0	3	1
Trifolium_campestre	60	40	0	2.98	0	8	4	6	3
Trifolium_dubium_agg.	66	33	0	2.45	0	6	4	6	4
Viola_tricolor_agg.	66	33	0	NA	0	7	4	0	0
Crataegus_monogyna	100	0	0	NA	1	7	4	8	4
Elymus_repens	100	0	0	2.72	0	7	0	0	7
Agrostis_capillaris	50	50	0	0.98	0	7	0	4	4
Juncus_bufonius_agg.	100	0	0	NA	0	7	7	3	4
Senecio_vulgaris	75	25	0	0.33	0	7	5	0	8
Rubus_fruticosus_agg.	66	33	0	NA	0.63	0	0	0	0
Rubus_idaeus	100	0	0	3.6	0.67	7	0	0	6
Moos_folios	NA	NA	NA	NA	NA	NA	NA	NA	NA
Moos_thallos	NA	NA	NA	NA	NA	NA	NA	NA	NA
Hieracium_umbellatum	100	0	0	0.63	0	6	3	4	3
Salix_caprea	100	0	0	0.13	1	7	6	7	7
Epilobium_ciliatum	100	0	0	0.19	0	7	5	7	8
Robinia_pseudoacacia	100	0	0	NA	1	5	4	0	8
Ulmus	100	0	0	NA	0	4	6	7	7
Cirsium_arvense	100	0	0	0.26	0	8	0	0	7
Torilis_japonica_agg.	100	0	0	NA	0	6	5	8	8
Silene_alba_subsp._alba	60	40	0	NA	0	8	4	0	7
Carex_hirta	75	25	0	3.47	0	7	6	0	5
Rumex_thyrsiflorus	100	0	0	1.15	0	8	3	7	4
Calamagrostis_epigejos	100	0	0	NA	0	7	0	0	6
Arctium_minus_agg.	100	0	0	NA	0	9	5	0	8
Hippophae_rhamnoides	100	0	0	3.73	1	9	4	8	3
Lupinus_polyphyllus	100	0	0	NA	0	7	5	4	0
Betula_pendula	100	0	0	0.38	1	8	0	0	0
Verbascum	100	0	0	NA	0	7	5	7	6
Malus_domestica	100	0	0	NA	1	0	0	0	0
Populus_tremula	100	0	0	NA	1	5	7	8	6
Artemisia_vulgaris_agg.	100	0	0	NA	0	7	6	0	8
Dactylis_glomerata_agg.	75	25	0	2.74	0	7	5	0	6
Pinus_sylvestris	100	0	0	NA	1	7	0	0	0
Sinapis_arvensis	100	0	0	3.85	0	7	0	8	6
Lupinus_luteus	100	0	0	NA	0	7	5	4	0
Echinochloa_crus-galli	50	50	0	3.15	0	6	5	0	8
Convolvulus_arvensis	0	36	0	4.03	0	7	4	7	0
Vicia_villosa	0	0	0	4.81	0	7	4	6	5
Lactuca_serriola	100	0	0	NA	0	9	4	0	4
Sonchus_asper	100	0	0	0.34	0	7	6	7	7
Chenopodium_album_agg.	83	16	0	NA	0	0	4	0	7
Crepis_foetida	100	0	0	NA	0	9	4	7	3
Vicia_angustifolia	33	0	0	NA	0	5	0	0	0
Papaver_rhoeas	75	25	0	NA	0	6	5	7	6
Cirsium_vulgare	100	0	0	0.3	0	8	5	7	8
Tripleurospermum_maritimum_agg.	0	99	0	2.33	0	9	6	7	8
Cerastium_holosteoides	100	0	0	1.99	0	6	5	0	5
Oenothera_parviflora_agg.	100	0	0	2.69	0	8	3	7	3
Sisymbrium_altissimum	100	0	0	2.29	0	8	4	7	4
Chenopodium_polyspermum	66	33	0	NA	0	6	6	0	8
Conyza_canadensis	100	0	0	0.26	0	8	4	0	5
Sonchus_arvensis_agg.	100	0	0	0.29	0	7	5	7	0
Papaver_dubium	100	0	0	NA	0	6	4	5	5
Hordeum_vulgare	100	0	0	5.06	0	9	4	7	7
Apera_spica-venti	100	0	0	1.31	0	6	6	5	0
Bromus_hordeaceus_agg.	60	40	0	2.72	0	7	0	0	3
Salix_viminalis	100	0	0	0.13	1	7	8	7	0
Corynephorus_canescens	100	0	0	NA	0	8	2	3	2
Danthonia_decumbens	33	66	0	2.59	0	8	0	3	2
Chondrilla_juncea	100	0	0	0.58	0	8	3	6	0
Carex_spicata	100	0	0	NA	0	7	4	6	4
Carex_arenaria_agg.	100	0	0	2.58	0	7	3	2	2
Melica_nutans_agg.	100	0	0	1.87	0	4	4	0	3
Typha	28	0	71	0.14	0	8	10	7	8
Artemisia_campestris_agg.	100	0	0	NA	0.38	9	2	5	2
Festuca_gigantea	100	0	0	4.09	0	4	7	6	6
Achillea_pannonica	100	0	0	NA	0	7	3	6	2
Hieracium_piloselloides	100	0	0	NA	NA	9	4	8	2
Crepis_capillaris	80	20	0	0.36	0	7	5	6	4
Carex_ericetorum	100	0	0	NA	0	5	4	0	2

Helichrysum_arenarium	50	50	0	0.2	0	8	2	5	1
Geum_urbanum	66	33	0	2.86	0	4	5	0	7
Fragaria_vesca	75	25	0	NA	0	7	5	0	6
Poa_compressa	100	0	0	1.52	0	9	3	9	3
Gnaphalium_sylvaticum	100	0	0	NA	0	8	5	4	6
Berteroa_incana	100	0	0	2.87	0	9	3	6	4
Ajuga_genevensis	75	25	0	NA	0	8	3	7	2
Centaurea_stoebe	NA	NA	NA	NA	NA	8	2	8	3
Moehringia_trinervia	25	75	0	NA	0	4	5	6	7
Crepis_tectorum	100	0	0	NA	0	8	4	0	6
Hieracium_bauhini	NA	NA	NA	NA	NA	9	3	7	1
Setaria_viridis	100	0	0	3.59	0	7	4	0	7
Arenaria_serpyllifolia_agg.	75	25	0	2.01	0	8	4	7	0
Digitaria_sanguinalis	66	33	0	NA	0	7	4	5	5
Senecio_vernalis	100	0	0	NA	0	7	4	7	6
Setaria_pumila	100	0	0	3.6	0	7	4	5	6
Herniaria_glabra	0	100	0	1.29	0.13	8	3	4	2
Digitaria_ischaemum	33	66	0	1.22	0	7	5	2	3
Erodium_cicutarium_agg.	100	0	0	2.5	0	8	4	0	0
Scleranthus_annuus_agg.	0	100	0	3.33	0	6	5	2	5
Scleranthus_perennis	25	75	0	NA	0.25	8	2	4	1
Bromus_tectorum	100	0	0	2.44	0	8	3	8	4
Vicia_tetrasperma_agg.	0	28	0	NA	0	6	5	5	5
Matricaria_recutita	100	0	0	NA	0	7	5	5	5
Centaurium_erythraea	100	0	0	NA	0	8	5	6	6
Ornithopus_perpusillus	0	100	0	NA	0	7	3	2	2
Salsola_kali_subsp._ruthenica	50	50	0	1.74	0	9	4	8	5
Senecio_viscosus	100	0	0	0.6	0	8	3	0	4
Filago_arvensis	100	0	0	NA	0	8	3	4	2
Filago_minima	100	0	0	0.19	0	9	2	4	1
Petrorhagia_prolifera	100	0	0	2.49	0	8	3	5	2
Cerastium_pumilum_agg.	100	0	0	NA	0	8	2	8	2
Hordeum_jubatum	100	0	0	1.92	0	9	6	7	6
Brachypodium_sylvaticum	75	25	0	3.09	0	3	5	6	6
Genista_pilosa	33	66	0	NA	0.83	7	0	2	1
Leontodon_taraxacoides	100	0	0	0.97	0	8	6	6	5
Plantago_lanceolata	100	0	0	3.31	0	6	0	0	0
Linaria_vulgaris	100	0	0	NA	0	8	4	7	5
Veronica_chamaedrys_agg.	42	56	0	2.17	0	6	4	7	6
Solidago_canadensis	100	0	0	0.31	0	8	0	0	6
Eupatorium_cannabinum	100	0	0	0.42	0	7	7	7	8
Rumex_crispus	100	0	0	2.27	0	7	7	0	6
Festuca_rubra_agg.	100	0	0	2.78	0	0	6	6	0
Deschampsia_cespitosa_agg.	100	0	0	2.21	0	6	7	0	3
Equisetum_arvense	100	0	0	NA	0	6	0	0	3
Hieracium_pilosella	100	0	0	0.41	0	7	4	0	2
Taraxacum_officinale_agg.	NA	NA	NA	NA	0	7	5	0	8
Leontodon_autumnalis	71	28	0	0.72	0	7	5	5	5
Holcus_mollis	100	0	0	1.66	0	5	5	2	3
Lolium_perenne	100	0	0	3.09	0	8	5	7	7
Poa_pratensis_agg.	75	25	0	2.12	0	6	5	0	6
Prunella_vulgaris	50	50	0	3.21	0	7	5	7	0
Hypochaeris_radicata	83	16	0	0.55	0	8	5	4	3
Trifolium_repens	33	66	0	2.91	0	8	5	6	6
Solanum_nigrum	83	16	0	NA	0	7	5	7	8
Plantago_major	80	20	0	2.52	0	8	5	0	6
Sagina_procumbens	0	100	0	0.88	0	7	5	7	6
Agrostis_stolonifera_agg.	50	25	25	1.11	0	8	7	0	5
Plantago_major_subsp._intermedia	NA	NA	NA	NA	0	7	7	5	4
Polygonum_aviculare_agg.	66	33	0	2.7	0	7	4	0	6
Poa_annua	55	44	0	1.94	0	7	6	0	8
Lepidium_ruderale	100	0	0	NA	0	9	4	0	6
Spergularia_rubra	0	99	0	1.13	0	7	5	3	4

Species	ln (Seeds per shoot)	Specific leaf area mm^2/mg	Early month seed shedding	Latest month seed shedding	Average month of seedling	Duration of seedling	Canopy height (m)
Vicia_hirsuta	5.298317367	NA	7	10	8.5	4	0.6
Epilobium_hirsutum	10.07154114	NA	8	11	9.5	4	1.5
Polygonum_persicaria	6.476972363	NA	6	10	8	5	0.75
Sisymbrium_officinale	7.901007052	NA	7	10	8.5	4	0.9
Erigeron_annuus	8.848365695	21.93	8	10	9	3	0.98
Epilobium_tetragonum	8.885994315	15.9	8	10	9	3	1

Poa_palustris	12.080318	29.28	7	9	8	3	1.1
Tragopogon_dubius	12.080318	27.03	6	9	7.5	4	0.55
Viola_canina_agg.	12.080318	NA	7	9	8	3	0.15
Agrostis_vinealis	4.317488114	15.64	9	11	10	3	0.15
Myosotis	12.080318	NA	NA	6	3	7	0
Tanacetum_vulgare	8.872487182	16.02	8	10	9	3	1.1
Phalaris_arundinacea	8.006367568	18.77	6	10	8	5	2.2
Leucanthemum_vulgare_agg.	6.946013991	19.78	6	10	8	5	0.6
Daucus_carota	8.29404964	21.7	8	11	9.5	4	1
Echium_vulgare	7.408530567	NA	7	11	9	5	0.9
Phragmites_australis	7.426549072	12.24	3	11	7	9	4
Jasione_montana	9.323669057	36.39	1	11	6	11	0.25
Viola_arvensis	7.824046011	NA	6	7	6.5	2	0.45
Trifolium_arvense	5.590986981	20.33	7	11	9	5	0.3
Trifolium_pratense	6.272877007	21.56	6	12	9	7	1
Hypericum_perforatum	10.05835226	28.52	1	12	6.5	12	1
Rumex_acetosa	5.487904134	23.08	5	11	8	7	1
Holcus_lanatus	12.08390501	25.03	6	11	8.5	6	0.5
Veronica_officinalis	5.382198851	25.38	1	12	6.5	12	0.4
Helictotrichon_pubescens	8.29404964	21.31	6	9	7.5	4	1.1
Luzula_multiflora_subsp._multiflora	6.041444479	NA	7	10	8.5	4	0.35
Potentilla_argentea	7.448333861	NA	7	8	7.5	2	0.5
Medicago_lupulina	7.368339686	25.54	6	12	9	7	0.6
Lotus_corniculatus_agg.	1.857859271	27.58	6	11	8.5	6	1.7
Rumex_acetosella_var._tenuifolius	6.907755279	38.02	6	10	8	5	0.2
Juncus_articulatus	12.080318	9.49	8	11	9.5	4	0.45
Tussilago_farfara	8.160518247	NA	3	7	5	5	0.3
Picris_hieracioides	6.85593553	24.82	7	10	8.5	4	0.93
Festuca_ovina_agg.	6.741700695	16.57	6	9	7.5	4	0.35
Trifolium_campestre	4.959342	NA	7	10	8.5	4	0.35
Trifolium_dubium_agg.	4.521788577	25.42	6	11	8.5	6	0.3
Viola_tricolor_agg.	7.162397497	NA	6	10	8	5	0.4
Crataegus_monogyna	12.080318	13.72	9	11	10	3	10
Elymus_repens	3.481240089	21.57	7	10	8.5	4	1.3
Agrostis_capillaris	5.260096154	26.76	1	12	6.5	12	0.4
Juncus_bufonius_agg.	9.76995616	22.18	6	10	8	5	0.25
Senecio_vulgaris	7.905994594	NA	3	12	7.5	10	0.3
Rubus_fruticosus_agg.	12.080318	16.1	9	11	10	3	4
Rubus_idaeus	6.40166841	23.88	6	9	7.5	4	2
Moos_folios	12.080318	NA	NA	6	3	7	0
Moos_thallos	12.080318	NA	NA	6	3	7	0
Hieracium_umbellatum	6.727671217	20.8	NA	6	3	7	0.8
Salix_caprea	16.00194946	NA	3	6	4.5	4	10
Epilobium_ciliatum	7.310550159	NA	6	11	8.5	6	1.5
Robinia_pseudoacacia	12.080318	NA	10	11	10.5	2	30
Ulmus	12.080318	35.24	3	7	5	5	40
Cirsium_arvense	8.517193191	10.07	7	12	9.5	6	1.5
Torilis_japonica_agg.	12.080318	NA	9	11	10	3	1.25
Silene_alba_subsp._alba	8.699514748	NA	6	11	8.5	6	0.9
Carex_hirta	5.991464547	17.53	7	9	8	3	0.8
Rumex_thyrsiflorus	7.233455419	18.46	NA	6	3	7	0.9
Calamagrostis_epigejos	12.89948167	6.1	7	10	8.5	4	1.45
Arctium_minus_agg.	9.367344121	NA	6	11	8.5	6	0.9
Hippophae_rhamnoides	12.080318	NA	8	10	9	3	5
Lupinus_polyphyllus	12.080318	18.95	6	10	8	5	1.5
Betula_pendula	12.080318	20.22	1	12	6.5	12	30
Verbascum	11.16424703	NA	NA	3	1.5	4	1.2
Malus_domestica	12.080318	NA	NA	6	3	7	10
Populus_tremula	12.080318	14.56	5	5	5	1	30
Artemisia_vulgaris_agg.	12.8346813	NA	7	12	9.5	6	2.25
Dactylis_glomerata_agg.	5.686975356	21.8	1	12	6.5	12	1.1
Pinus_sylvestris	10.01471582	4.97	3	5	4	3	46
Sinapis_arvensis	7.824046011	NA	6	10	8	5	0.8
Lupinus_luteus	5.703782475	22.74	NA	6	3	7	0.54
Echinochloa_crus-galli	6.26196867	27.56	9	10	9.5	2	0.85
Convolvulus_arvensis	6.309918278	25.8	7	10	8.5	4	0.8
Vicia_villosa	5.521460918	NA	NA	6	3	7	1.2
Lactuca_serriola	12.080318	18.07	6	11	8.5	6	0.9
Sonchus_asper	8.455317788	21.14	5	12	8.5	8	0.7
Chenopodium_album_agg.	9.350102314	34.84	6	11	8.5	6	1.4
Crepis_foetida	12.080318	NA	NA	6	3	7	0.2
Vicia_angustifolia	5.298317367	NA	NA	6	3	7	0.8
Papaver_rhoeas	9.903487553	NA	6	11	8.5	6	0.9
Cirsium_vulgare	8.1285852	10.6	8	11	9.5	4	1.2

Tripleurospermum_maritimum_agg.	10.4341158	19.74	1	12	6.5	12	0.4
Cerastium_holosteoides	5.872117789	39.38	5	10	7.5	6	0.25
Oenothera_parviflora_agg.	8.746334719	17.99	NA	6	3	7	1.9
Sisymbrium_altissimum	9.243097936	21.98	NA	6	3	7	1.01
Chenopodium_polyspermum	8.29404964	33.17	8	10	9	3	1
Conyza_canadensis	11.60823564	22.75	7	12	9.5	6	0.9
Sonchus_arvensis_agg.	8.764053269	NA	6	12	9	7	1.3
Papaver_dubium	9.798127037	NA	6	9	7.5	4	1
Hordeum_vulgare	5.442417711	21.75	NA	6	3	7	1.5
Apera_spica-venti	8.546751994	NA	6	10	8	5	0.85
Bromus_hordeaceus_agg.	5.65248918	28.2	3	8	5.5	6	0.8
Salix_viminalis	16.33401316	11.26	NA	6	3	7	6
Corynephorus_canescens	12.080318	13.09	1	12	6.5	12	0.1
Danthonia_decumbens	4.709530201	16.39	7	10	8.5	4	0.15
Chondrilla_juncea	6.424058323	7.53	7	10	8.5	4	0.75
Carex_spicata	12.080318	23.41	6	10	8	5	0.4
Carex_arenaria_agg.	12.080318	13.33	NA	6	3	7	0.6
Melica_nutans_agg.	2.32238772	NA	7	8	7.5	2	0.3
Typha	12.79385931	6.31	NA	6	3	7	2
Artemisia_campestris_agg.	10.11730778	15.99	8	12	10	5	0.6
Festuca_gigantea	5.164785974	NA	8	10	9	3	1.4
Achillea_pannonica	6.300785795	8.08	7	15	11	9	0.7
Hieracium_piloselloides	12.080318	23.7	6	10	8	5	0.15
Crepis_capillaris	8.987196821	NA	6	10	8	5	0.45
Carex_ericetorum	12.080318	NA	5	8	6.5	4	0.2
Helichrysum_arenarium	12.080318	18.07	8	9	8.5	2	0.3
Geum_urbanum	5.514436303	40.38	6	10	8	5	0.8
Fragaria_vesca	5.697093487	21.2	6	10	8	5	0.3
Poa_compressa	6.711740395	NA	7	12	9.5	6	0.6
Gnaphalium_sylvaticum	9.237079669	23.91	8	10	9	3	0.55
Berteroa_incana	8.874867636	20.62	NA	6	3	7	0.4
Ajuga_genevensis	12.080318	25.78	6	10	8	5	0.3
Centaurea_stoebe	12.080318	20.37	NA	6	3	7	0.8
Moehringia_trinervia	5.152713393	NA	6	10	8	5	0.4
Crepis_tectorum	7.521859252	NA	7	10	8.5	4	0.45
Hieracium_bauhini	12.080318	NA	NA	6	3	7	0.8
Setaria_viridis	8.777555453	27.74	8	10	9	3	1.1
Arenaria_serpyllifolia_agg.	6.003887067	16.05	6	9	7.5	4	0.3
Digitaria_sanguinalis	5.298317367	35.25	8	10	9	3	0.3
Senecio_vernalis	8.29404964	24.01	7	8	7.5	2	0.4
Setaria_pumila	7.436617265	25.46	8	12	10	5	0.57
Herniaria_glabra	6.912494033	30.76	6	11	8.5	6	0.3
Digitaria_ischaemum	5.749392986	40.62	9	10	9.5	2	0.4
Erodium_cicutarium_agg.	5.991464547	46.79	7	7	7	1	0.6
Scleranthus_annuus_agg.	6.946975992	18.92	6	12	9	7	0.2
Scleranthus_perennis	6.490723535	18.93	8	8	8	1	0.2
Bromus_tectorum	7.03284489	35.03	6	10	8	5	0.52
Vicia_tetrasperma_agg.	5.768320996	26.19	6	10	8	5	0.6
Matricaria_recutita	8.594154233	NA	6	8	7	3	0.35
Centaurium_erythraea	8.860641042	22.54	9	11	10	3	0.5
Ornithopus_perpusillus	12.080318	NA	NA	6	3	7	0.45
Salsola_kali_subsp._ruthenica	5.703782475	9.1	NA	6	3	7	0.6
Senecio_viscosus	8.160518247	NA	6	11	8.5	6	0.45
Filago_arvensis	5.298317367	39.06	9	12	10.5	4	0.35
Filago_minima	5.988961417	32.45	9	12	10.5	4	0.2
Petrorhagia_prolifera	6.923136381	17.63	6	11	8.5	6	0.5
Cerastium_pumilum_agg.	4.465908119	30.13	5	8	6.5	4	0.12
Hordeum_jubatum	12.080318	NA	7	7	7	1	0.4
Brachypodium_sylvaticum	4.49980967	NA	8	10	9	3	1.1
Genista_pilosa	12.080318	12.54	6	7	6.5	2	0.4
Leontodon_taraxacoides	12.080318	18.62	NA	6	3	7	0.1
Plantago_lanceolata	7.313220387	16.2	6	12	9	7	0.4
Linaria_vulgaris	9.913091289	NA	8	12	10	5	0.8
Veronica_chamaedrys_agg.	3.288401888	28.73	7	10	8.5	4	0.4
Solidago_canadensis	9.362202721	NA	9	12	10.5	4	2.3
Eupatorium_cannabinum	6.352629396	36.83	9	11	10	3	1.5
Rumex_crispus	8.219056661	13.28	6	11	8.5	6	1.5
Festuca_rubra_agg.	6.142037406	16.23	7	9	8	3	0.9
Deschampsia_cespitosa_agg.	8.411999329	13.56	8	11	9.5	4	2
Equisetum_arvense	12.080318	10.58	3	4	3.5	2	0.5
Hieracium_pilosella	3.877431561	22.7	6	10	8	5	0.1
Taraxacum_officinale_agg.	12.080318	NA	NA	6	3	7	0.4
Leontodon_autumnalis	6.711131937	24.7	6	11	8.5	6	0.15
Holcus_mollis	5.703782475	32.82	6	11	8.5	6	0.4

Lolium_perenne	12.080318	8.84	6	10	8	5	0.2
Poa_pratensis_agg.	5.33753808	24.57	6	9	7.5	4	0.5
Prunella_vulgaris	5.322522055	21.72	8	12	10	5	0.3
Hypochaeris_radicata	6.829793738	NA	6	11	8.5	6	0.1
Trifolium_repens	5.298317367	26.69	6	11	8.5	6	0.5
Solanum_nigrum	9.107144179	NA	6	11	8.5	6	0.7
Plantago_major	9.216223035	21.9	3	12	7.5	10	0.6
Sagina_procumbens	6.684611728	NA	5	10	7.5	6	0.2
Agrostis_stolonifera_agg.	12.080318	22.76	6	11	8.5	6	0.7
Plantago_major_subsp._intermedia	7.334166152	22.16	3	12	7.5	10	0.5
Polygonum_aviculare_agg.	5.090678002	16.53	6	12	9	7	2
Poa_annua	6.907755279	33.03	1	12	6.5	12	0.15
Lepidium_ruderale	7.170119543	NA	7	10	8.5	4	0.3
Spergularia_rubra	6.907755279	NA	6	10	8	5	0.25

Species	Seed bank longevity	Chromosome number	Polyploidy	DNA content	Early month flowering	Latest month flowering	Duration of flowering	Type reproduction
Vicia_hirsuta	0.25	7	2	8	6	7	2	1
Epilobium_hirsutum	0.64	18	2	0.6	6	9	4	3
Polygonum_persicaria	0.69	11	4	0.9	7	10	4	1
Sisymbrium_officinale	0.41	7	2	NA	5	10	6	1
Erigeron_annuus	0.41	9	3	NA	6	9	4	1
Epilobium_tetragonum	0.47	18	2	1.2	7	8	2	3
Poa_palustris	0.11	7	4	NA	6	7	2	3
Tragopogon_dubius	0.75	6	2	NA	5	7	3	1
Viola_canina_agg.	0.59	10	4	NA	5	6	2	2
Agrostis_vinealis	0.41	7	4	6.9	6	9	4	3
Myosotis	0	13	4	NA	4	9	6	1
Tanacetum_vulgare	0.16	9	2	7.8	7	9	3	2
Phalaris_arundinacea	0.6	7	4	8.2	6	7	2	3
Leucanthemum_vulgare_agg.	0.36	9	2	19	6	10	5	3
Daucus_carota	0.31	9	2	1.2	6	9	4	1
Echium_vulgare	0.29	8	4	NA	5	7	3	1
Phragmites_australis	0.2	12	2	2.4	7	9	3	4
Jasione_montana	0.06	6	2	NA	6	8	3	1
Viola_arvensis	0.53	17	2	NA	4	10	7	1
Trifolium_arvense	0.49	7	2	1.6	6	9	4	1
Trifolium_pratense	0.24	7	2	1	6	9	4	1
Hypericum_perforatum	0.64	9	2	1.2	7	8	2	3
Rumex_acetosa	0.23	7	2	3.3	5	7	3	3
Holcus_lanatus	0.51	7	2	3.8	6	8	3	3
Veronica_officinalis	0.63	9	2	NA	6	8	3	3
Helictotrichon_pubescens	0.08	7	2	12.6	5	6	2	3
Luzula_multiflora_subsp._multiflora	0.53	6	4	1.9	4	5	2	3
Potentilla_argentea	0.61	7	2	NA	6	10	5	1
Medicago_lupulina	0.35	8	2	1.8	5	10	6	3
Lotus_corniculatus_agg.	0.15	6	4	2.1	6	8	3	2
Rumex_acetosella_var._tenuifolius	0.7	7	4	3.4	5	7	3	3
Juncus_articulatus	0.91	20	4	3.7	7	9	3	3
Tussilago_farfara	0.03	30	2	4.6	3	4	2	3
Picris_hieracioides	0.18	5	2	5.7	7	10	4	1
Festuca_ovina_agg.	0.1	7	2	9.5	8	5	4	1
Trifolium_campestre	0.2	7	2	1	6	9	4	1
Trifolium_dubium_agg.	0.33	8	4	2.1	5	9	5	1
Viola_tricolor_agg.	0.53	13	2	NA	4	9	6	1
Crataegus_monogyna	0.03	17	2	23.8	5	6	2	1
Elymus_repens	0.07	7	6	26	6	8	3	4
Agrostis_capillaris	0.52	7	4	7.1	6	7	2	3
Juncus_bufonius_agg.	0.9	18	6	2.6	5	8	4	1
Senecio_vulgaris	0.55	10	4	3	2	11	10	1
Rubus_fruticosus_agg.	0.11	7	4	0.6	6	8	3	0
Rubus_idaeus	0.68	7	6	0.6	6	7	2	0
Moos_folios	0	NA	NA	NA	NA	NA	NA	0
Moos_thallos	0	NA	NA	NA	NA	NA	NA	0
Hieracium_umbellatum	0.24	9	2	7	5	8	4	0
Salix_caprea	0.02	19	2	1	3	4	2	1
Epilobium_ciliatum	0.97	18	1	1.1	6	9	4	3
Robinia_pseudoacacia	0.77	10	2	1.3	5	6	2	3
Ulmus	0	14	2	2.1	3	4	2	1
Cirsium_arvense	0.2	17	2	3.1	7	9	3	3
Torilis_japonica_agg.	0.47	8	2	4.6	6	8	3	1
Silene_alba_subsp._alba	0.44	12	2	5.7	6	9	4	2

Carex_hirta	0.19	5	11	NA	5	6	2	3
Rumex_thyrsiflorus	0	7	2	NA	7	8	2	1
Calamagrostis_epigejos	0.14	7	5	NA	7	8	2	3
Arctium_minus_agg.	0.67	8	4	NA	7	9	3	1
Hippophae_rhamnoides	0	12	2	NA	3	5	3	3
Lupinus_polyphyllus	1	12	4	NA	6	8	3	1
Betula_pendula	0.75	14	2	NA	4	5	2	1
Verbascum	0.6	15	2	NA	6	9	4	1
Malus_domestica	0	17	2	NA	4	5	2	2
Populus_tremula	0	19	2	NA	3	4	2	3
Artemisia_vulgaris_agg.	0.34	8	2	6	7	11	5	1
Dactylis_glomerata_agg.	0.15	7	4	9.8	5	7	3	2
Pinus_sylvestris	0	12	2	27.6	5	6	2	1
Sinapis_arvensis	0.83	9	2	0.8	6	10	5	1
Lupinus_luteus	0	13	4	1.9	6	9	4	1
Echinochloa_crus-galli	0.7	9	6	2.7	7	10	4	1
Convolvulus_arvensis	0.1	12	4	3.6	6	9	4	4
Vicia_villosa	0.18	7	2	3.7	6	9	4	1
Lactuca_serriola	0.21	9	2	3.7	7	9	3	1
Sonchus_asper	0.51	9	2	3.7	6	10	5	1
Chenopodium_album_agg.	0.83	9	3	3.8	7	10	4	1
Crepis_foetida	1	5	2	4.5	6	8	3	1
Vicia_angustifolia	0.22	6	2	5	5	7	3	1
Papaver_rhoeas	0.63	7	4	5.2	5	7	3	1
Cirsium_vulgare	0.2	17	4	5.2	6	9	4	1
Tripleurospermum_maritimum_agg.	1	9	2	5.3	6	10	5	1
Cerastium_holosteoides	0.21	18	8	5.9	3	6	4	3
Oenothera_parviflora_agg.	1	7	2	NA	7	9	3	1
Sisymbrium_altissimum	1	7	2	NA	5	7	3	1
Chenopodium_polyspermum	0.76	8	2	NA	7	9	3	1
Conyza_canadensis	0.5	9	3	NA	7	10	4	1
Sonchus_arvensis_agg.	0.78	9	4	NA	7	10	4	3
Papaver_dubium	0.43	7	4	7.4	5	7	3	1
Hordeum_vulgare	0	7	2	8.7	5	7	3	1
Apera_spica-venti	0.53	7	2	10.8	6	7	2	1
Bromus_hordeaceus_agg.	0.13	7	4	22.1	5	8	4	1
Salix_viminalis	0	19	2	1.6	3	4	2	3
Corynephorus_canescens	0.69	7	2	2.3	6	7	2	1
Danthonia_decumbens	0.29	6	4	5.9	6	7	2	3
Chondrilla_juncea	0	5	3	NA	7	9	3	2
Carex_spicata	0.1	5	6	NA	5	6	2	2
Carex_arenaria_agg.	0.22	5	6	NA	5	10	6	4
Melica_nutans_agg.	0.4	9	2	NA	5	6	2	3
Typha	1	15	2	NA	7	8	2	3
Artemisia_campestris_agg.	0.17	9	4	11	8	10	3	1
Festuca_gigantea	0.08	7	6	14.5	7	8	2	1
Achillea_pannonica	0	9	8	19.1	6	8	3	3
Hieracium_piloselloides	0	9	4	2	5	8	4	3
Crepis_capillaris	0.08	3	2	4.2	6	10	5	1
Carex_ericetorum	0	5	6	NA	4	5	2	3
Helichrysum_arenarium	0	7	4	NA	7	8	2	3
Geum_urbanum	0.05	7	6	NA	5	10	6	2
Fragaria_vesca	0.31	7	2	NA	5	6	2	3
Poa_compressa	0.5	7	6	NA	6	7	2	3
Gnaphalium_sylvaticum	0.71	7	8	NA	7	9	3	3
Berteroa_incana	0.25	8	2	NA	6	10	5	1
Ajuga_genevensis	0.56	8	4	NA	4	6	3	2
Centaurea_stoebe	0	9	2	NA	7	9	3	1
Moehringia_trinervia	0.59	12	2	NA	5	7	3	1
Crepis_tectorum	0.14	4	2	6.8	5	10	6	1
Hieracium_bauhini	0	9	4	8.9	5	7	3	3
Setaria_viridis	0.77	9	2	1	6	10	5	1
Arenaria_serpyllifolia_agg.	0.65	10	4	1.7	5	9	5	1
Digitaria_sanguinalis	0.95	3	4	2.4	7	10	4	1
Senecio_vernalis	0	10	2	2.5	5	11	7	1
Setaria_pumila	0.81	9	6	5.3	7	10	4	2
Herniaria_glabra	0	9	2	NA	6	10	5	1
Digitaria_ischaemum	0.6	9	4	NA	7	10	4	1
Erodium_cicutarium_agg.	0.13	10	2	NA	4	10	7	1
Scleranthus_annuus_agg.	0.13	11	4	NA	4	10	7	1
Scleranthus_perennis	0.5	11	2	NA	5	9	5	1
Bromus_tectorum	0.25	7	2	6.5	6	5	2	1
Vicia_tetrasperma_agg.	0.2	7	2	7.2	6	7	2	1
Matricaria_recutita	0.72	9	2	7.8	5	8	4	1

Centaurium_erythraea	0.79	10	4	2.5	7	9	3	1
Ornithopus_perpusillus	0.31	7	2	NA	5	6	2	1
Salsola_kali_subsp._ruthenica	0.25	9	4	NA	7	9	3	1
Senecio_viscosus	0.78	10	4	NA	6	10	5	1
Filago_arvensis	0	14	2	NA	7	9	3	1
Filago_minima	0.17	14	2	NA	7	9	3	1
Petrorhagia_prolifera	0	15	2	NA	6	10	5	1
Cerastium_pumilum_agg.	0.25	18	5	NA	3	5	3	1
Hordeum_jubatum	0.63	7	4	21.7	6	7	2	2
Brachypodium_sylvaticum	0.14	9	2	1	7	8	2	3
Genista_pilosa	0	11	2	NA	5	6	2	1
Leontodon_taraxacoides	0.12	4	2	1.7	7	8	2	3
Plantago_lanceolata	0.24	6	2	2.5	5	10	6	2
Linaria_vulgaris	0.25	6	2	NA	6	10	5	3
Veronica_chamaedrys_agg.	0.42	8	4	NA	5	7	3	3
Solidago_canadensis	0.19	9	3	3.1	8	10	3	3
Eupatorium_cannabinum	0.44	10	2	NA	7	9	3	3
Rumex_crispus	0.3	10	6	8.8	6	8	3	3
Festuca_rubra_agg.	0.14	7	6	9.5	6	7	2	3
Deschampsia_cespitosa_agg.	0.26	13	2	18	6	7	2	1
Equisetum_arvense	0	108	2	28.4	3	4	2	3
Hieracium_pilosella	0.15	9	2	2.1	5	10	6	3
Taraxacum_officinale_agg.	0	8	2	2.6	3	10	8	1
Leontodon_autumnalis	0.11	6	2	2.7	7	9	3	3
Holcus_mollis	0.03	7	4	5.6	6	8	3	4
Lolium_perenne	0.19	7	2	6.7	5	10	6	1
Poa_pratensis_agg.	0.38	7	6	10.8	5	6	2	3
Prunella_vulgaris	0.2	17	2	1.3	6	9	4	3
Hypochaeris_radicata	0.17	4	2	2.7	6	9	4	2
Trifolium_repens	0.38	8	4	3.1	5	9	5	3
Solanum_nigrum	0.74	12	6	6.2	6	10	5	1
Plantago_major	0.65	6	2	1.6	6	10	5	2
Sagina_procumbens	0.82	11	2	NA	5	9	5	1
Agrostis_stolonifera_agg.	0.38	7	4	7	6	7	2	3
Plantago_major_subsp._intermedia	0.65	6	2	1.6	6	10	5	1
Polygonum_aviculare_agg.	0.57	10	6	1.7	5	11	7	1
Poa_annua	0.83	7	4	5.2	1	12	12	2
Lepidium_ruderale	0	8	4	NA	5	10	6	1
Spergularia_rubra	1	9	4	NA	5	9	5	1

**Species**	**Life strategy type**	**Grazing tolerance**	**Hemerobic level**
Vicia_hirsuta	5	1	2
Epilobium_hirsutum	1	2	3
Polygonum_persicaria	2	2	3
Sisymbrium_officinale	2	2	3
Erigeron_annuus	2	2	2
Epilobium_tetragonum	3	2	4
Poa_palustris	3	2	3
Tragopogon_dubius	4	2	3
Viola_canina_agg.	4	2	2
Agrostis_vinealis	4	2	2
Myosotis	5	2	2
Tanacetum_vulgare	1	3	2
Phalaris_arundinacea	1	3	4
Leucanthemum_vulgare_agg.	1	3	2
Daucus_carota	2	3	3
Echium_vulgare	2	3	3
Phragmites_australis	3	3	3
Jasione_montana	4	3	2
Viola_arvensis	5	3	2
Trifolium_arvense	6	3	3
Trifolium_pratense	1	4	2
Hypericum_perforatum	1	4	5
Rumex_acetosa	1	4	2
Holcus_lanatus	1	4	2
Veronica_officinalis	1	4	2
Helictotrichon_pubescens	1	4	2
Luzula_multiflora_subsp._multiflora	3	4	2
Potentilla_argentea	3	4	3
Medicago_lupulina	4	4	3
Lotus_corniculatus_agg.	4	4	3
Rumex_acetosella_var._tenuifolius	4	4	4
Juncus_articulatus	4	4	4

Tussilago_farfara	4	4	4
Picris_hieracioides	4	4	4
Festuca_ovina_agg.	4	4	2
Trifolium_campestre	5	4	2
Trifolium_dubium_agg.	5	4	2
Viola_tricolor_agg.	5	4	3
Crataegus_monogyna	1	5	3
Elymus_repens	1	5	4
Agrostis_capillaris	4	5	3
Juncus_bufonius_agg.	5	5	4
Senecio_vulgaris	5	5	2
Rubus_fruticosus_agg.	NA	NA	NA
Rubus_idaeus	NA	NA	NA
Moos_folios	NA	NA	NA
Moos_thallos	NA	NA	NA
Hieracium_umbellatum	NA	NA	NA
Salix_caprea	1	NA	3
Epilobium_ciliatum	1	NA	4
Robinia_pseudoacacia	1	NA	3
Ulmus	1	NA	2
Cirsium_arvense	1	NA	4
Torilis_japonica_agg.	1	NA	2
Silene_alba_subsp._alba	1	NA	3
Carex_hirta	1	NA	4
Rumex_thyrsiflorus	1	NA	5
Calamagrostis_epigejos	1	NA	2
Arctium_minus_agg.	1	NA	2
Hippophae_rhamnoides	1	NA	2
Lupinus_polyphyllus	1	NA	2
Betula_pendula	1	NA	3
Verbascum	1	NA	2
Malus_domestica	1	NA	2
Populus_tremula	1	NA	2
Artemisia_vulgaris_agg.	1	NA	3
Dactylis_glomerata_agg.	1	NA	2
Pinus_sylvestris	1	NA	3
Sinapis_arvensis	2	NA	2
Lupinus_luteus	2	NA	1
Echinochloa_crus-galli	2	NA	4
Convolvulus_arvensis	2	NA	4
Vicia_villosa	2	NA	2
Lactuca_serriola	2	NA	3
Sonchus_asper	2	NA	2
Chenopodium_album_agg.	2	NA	3
Crepis_foetida	2	NA	3
Vicia_angustifolia	2	NA	3
Papaver_rhoeas	2	NA	3
Cirsium_vulgare	2	NA	2
Tripleurospermum_maritimum_agg.	2	NA	5
Cerastium_holosteoides	2	NA	3
Oenothera_parviflora_agg.	2	NA	0
Sisymbrium_altissimum	2	NA	2
Chenopodium_polyspermum	2	NA	2
Conyza_canadensis	2	NA	3
Sonchus_arvensis_agg.	2	NA	3
Papaver_dubium	2	NA	3
Hordeum_vulgare	2	NA	2
Apera_spica-venti	2	NA	3
Bromus_hordeaceus_agg.	2	NA	3
Salix_viminalis	3	NA	3
Corynephorus_canescens	3	NA	2
Danthonia_decumbens	3	NA	2
Chondrilla_juncea	3	NA	2
Carex_spicata	3	NA	2
Carex_arenaria_agg.	3	NA	3
Melica_nutans_agg.	3	NA	2
Typha	3	NA	3
Artemisia_campestris_agg.	3	NA	3
Festuca_gigantea	3	NA	2
Achillea_pannonica	3	NA	2
Hieracium_piloselloides	4	NA	2
Crepis_capillaris	4	NA	2
Carex_ericetorum	4	NA	2
Helichrysum_arenarium	4	NA	2

Geum_urbanum	4	NA	3
Fragaria_vesca	4	NA	2
Poa_compressa	4	NA	5
Gnaphalium_sylvaticum	4	NA	2
Berteroa_incana	4	NA	3
Ajuga_genevensis	4	NA	2
Centaurea_stoebe	4	NA	3
Moehringia_trinervia	4	NA	3
Crepis_tectorum	4	NA	2
Hieracium_bauhini	4	NA	3
Setaria_viridis	5	NA	2
Arenaria_serpyllifolia_agg.	5	NA	4
Digitaria_sanguinalis	5	NA	2
Senecio_vernalis	5	NA	3
Setaria_pumila	5	NA	2
Herniaria_glabra	5	NA	2
Digitaria_ischaemum	5	NA	2
Erodium_cicutarium_agg.	5	NA	2
Scleranthus_annuus_agg.	5	NA	4
Scleranthus_perennis	5	NA	2
Bromus_tectorum	5	NA	2
Vicia_tetrasperma_agg.	5	NA	3
Matricaria_recutita	5	NA	3
Centaurium_erythraea	6	NA	3
Ornithopus_perpusillus	6	NA	3
Salsola_kali_subsp._ruthenica	6	NA	2
Senecio_viscosus	6	NA	3
Filago_arvensis	6	NA	3
Filago_minima	6	NA	3
Petrorhagia_prolifera	6	NA	3
Cerastium_pumilum_agg.	6	NA	3
Hordeum_jubatum	6	NA	3
Brachypodium_sylvaticum	3	6	2
Genista_pilosa	3	6	2
Leontodon_taraxacoides	4	6	2
Plantago_lanceolata	4	6	3
Linaria_vulgaris	4	6	4
Veronica_chamaedrys_agg.	4	6	3
Solidago_canadensis	1	7	3
Eupatorium_cannabinum	1	7	2
Rumex_crispus	1	7	3
Festuca_rubra_agg.	1	7	1
Deschampsia_cespitosa_agg.	1	7	3
Equisetum_arvense	2	7	4
Hieracium_pilosella	4	7	3
Taraxacum_officinale_agg.	4	7	3
Leontodon_autumnalis	4	7	2
Holcus_mollis	4	7	3
Lolium_perenne	1	8	3
Poa_pratensis_agg.	1	8	3
Prunella_vulgaris	4	8	2
Hypochaeris_radicata	4	8	3
Trifolium_repens	4	8	3
Solanum_nigrum	5	8	3
Plantago_major	4	9	2
Sagina_procumbens	4	9	3
Agrostis_stolonifera_agg.	4	9	4
Plantago_major_subsp._intermedia	5	9	3
Polygonum_aviculare_agg.	5	9	3
Poa_annua	5	9	3
Lepidium_ruderale	5	9	3
Spergularia_rubra	5	9	2
