# Supplementary material for: Small-scale spatial variability in phylogenetic community structure during early plant succession depends on soil properties
Source: Oecologia. 2014 May 9;175(3):985–95. doi: 10.1007/s00442-014-2954-2 (PMC4059990; doi:10.1007/s00442-014-2954-2)
Supplement: Supplementary file 2 — Electronic supplementary material B (PDF 2635 kb) [file 442_2014_2954_MOESM2_ESM.pdf]

Small scale spatial variability in phylogenetic signals during early plant succession depends on soil properties
Oecologia

Werner Ulrich[1], Marcin Piwczyński, Markus Klemens Zaplata, Susanne Winter,
[1]Chair of Ecology and Biogeography, Nicolaus Copernicus University in Torun, Lwowska 1, PL 87-100 Toruń, Poland.
Phone: 0048 56 611 2649, e-mail: *ulrichw@umk.pl*

Principal components extracted from the phylogenetic distance matrix

S	PCA1	PCA2	PCA3	PCA4	PCA5
Achillea_pannonica	-468.85	195.67	312.73	31.158	13.725
Agrostis_capillaris	601.53	-808.36	42.733	5.1611	5.1178
Agrostis_stolonifera_agg.	601.53	-808.36	42.733	5.1611	5.1178
Agrostis_vinealis	601.53	-808.36	42.733	5.1611	5.1178
Ajuga_genevensis	-190.98	188.57	-72.698	-66.893	-231.37
Apera_spica_venti	601.67	-809.9	42.84	5.1872	5.1693
Arctium_minus_agg.	-453.06	195.68	295.15	27.761	10.307
Arenaria_serpyllifolia_agg.	-233.17	182.22	-163.4	-338.57	89.102
Artemisia_campestris_agg.	-467.18	195.69	311	30.851	13.477
Artemisia_vulgaris_agg.	-467.18	195.69	311	30.851	13.477
Berteroa_incana	-164.27	186.3	-187.86	39.499	-64.859
Betula_pendula	-190.15	184.73	-234.44	99.492	5.5541
Brachypodium_sylvaticum	600.51	-797.43	41.981	4.9797	4.763
Bromus_hordeaceus	601.22	-805.11	42.508	5.1066	5.0101
Bromus_tectorum	601.22	-805.11	42.508	5.1066	5.0101
Calamagrostis_epigejos	601.53	-808.36	42.733	5.1611	5.1178
Carex_arenaria_agg.	585.55	-646.07	31.115	2.2724	-0.86883
Carex_ericetorum	585.55	-646.07	31.115	2.2724	-0.86883
Carex_hirta	585.55	-646.07	31.115	2.2724	-0.86883
Carex_spicata	585.55	-646.07	31.115	2.2724	-0.86883
Centaurea_stoebe	-453.06	195.68	295.15	27.761	10.307
Centaurium_erythraea	-155.65	193.46	-59.465	-47.227	-98.727
Cerastium_holosteoides	-233.96	182.13	-163.98	-340.55	90.046
Cerastium_pumilum_agg.	-233.96	182.13	-163.98	-340.55	90.046
Chenopodium_album_agg.	-216.85	184.1	-151.31	-295.96	68.134
Chenopodium_polyspermum	-216.85	184.1	-151.31	-295.96	68.134
Chondrilla_juncea	-512.72	196.01	364.71	41.867	26.099
Cirsium_arvense	-453.06	195.68	295.15	27.761	10.307
Cirsium_vulgare	-453.06	195.68	295.15	27.761	10.307
Convolvulus_arvensis	-156.41	193.33	-59.711	-47.521	-99.863
Conyza_canadensis	-493.92	195.88	342.58	37.338	20.933
Corynephorus_canescens	601.53	-808.36	42.733	5.1611	5.1178
Crataegus_monogyna	-215.6	181.51	-264.79	123.83	8.6078
Crepis_capillaris	-514.37	196.02	366.64	42.26	26.543
Crepis_foetida	-514.37	196.02	366.64	42.26	26.543
Crepis_tectorum	-514.37	196.02	366.64	42.26	26.543
Dactylis_glomerata_agg.	601.63	-809.41	42.806	5.179	5.1531
Danthonia_decumbens	599.77	-789.71	41.441	4.8482	4.5009
Daucus_carota	-190.15	194.82	-6.3386	-32.312	-54.346
Deschampsia_cespitosa_agg.	601.65	-809.62	42.82	5.1825	5.16
Digitaria_ischaemum	599.94	-791.58	41.565	4.8772	4.5545
Digitaria_sanguinalis	599.94	-791.58	41.565	4.8772	4.5545
Echinochloa_crus_galli	599.9	-791.2	41.54	4.8714	4.5438
Echium_vulgare	-157.97	193.08	-60.22	-48.144	-102.35
Elymus_repens	601.23	-805.24	42.516	5.1086	5.0141
Epilobium_ciliatum	-161.78	186.64	-185.31	38.487	-60.709
Epilobium_hirsutum	-161.78	186.64	-185.31	38.487	-60.709
Epilobium_tetragonum	-161.78	186.64	-185.31	38.487	-60.709
Equisetum_arvense	2871.8	1898.9	161.13	19.635	13.623
Erigeron_annuus	-493.92	195.88	342.58	37.338	20.933
Erodium_cicutarium_agg.	-151.47	188.33	-176.29	35.439	-51.609
Eupatorium_cannabinum	-476.56	195.83	322.66	33.359	16.607
Festuca_gigantea	601.7	-810.18	42.859	5.1918	5.1783
Festuca_ovina_agg.	601.7	-810.18	42.859	5.1918	5.1783
Festuca_rubra_agg.	601.7	-810.18	42.859	5.1918	5.1783
Filago_arvensis	-489.29	195.84	337.04	36.188	19.586
Filago_minima	-489.29	195.84	337.04	36.188	19.586
Fragaria_vesca	-218.44	181.15	-268.21	126.6	8.9615
Galeopsis_segetum	-190.98	188.57	-72.698	-66.893	-231.37
Genista_pilosa	-230.1	180.62	-291.16	158.86	56.636
Geum_urbanum	-217.49	181.28	-267.09	125.71	8.8513
Gnaphalium_sylvaticum	-488.48	195.84	336.16	36.02	19.421
Helichrysum_arenarium	-486.86	195.85	334.38	35.682	19.09

Eigenvalues and explained variance bases on the variance-covariance matrix

Axes	Eigenvalue	% variance
PCA1	395034	52.216
PCA2	273116	36.1
PCA3	50175	6.6321
PCA4	16397.2	2.1674
PCA5	5139.37	0.67932

Helictotrichon_pubescens	601.67	-809.83	42.835	5.1859	5.1668
Herniaria_glabra	-216.59	184.19	-151.35	-296.94	69.348
Hieracium_bauhini	-506.45	195.96	357.26	40.332	24.324
Hieracium_pilosella	-506.45	195.96	357.26	40.332	24.324
Hieracium_piloselloides	-506.45	195.96	357.26	40.332	24.324
Hieracium_sabaudum	-506.45	195.96	357.26	40.332	24.324
Hieracium_umbellatum	-506.45	195.96	357.26	40.332	24.324
Hippophae_rhamnoides	-198.49	183.64	-244.13	107.05	6.4486
Holcus_lanatus	601.69	-810.04	42.849	5.1894	5.1737
Holcus_mollis	601.69	-810.04	42.849	5.1894	5.1737
Hordeum_jubatum	601.26	-805.49	42.533	5.1128	5.022
Hordeum_vulgare	601.26	-805.49	42.533	5.1128	5.022
Hypericum_perforatum	-185.95	184.85	-225.69	87.292	-9.8544
Hypochaeris_radicata	-512.39	196.01	364.32	41.787	26.007
Jasione_montana	-256.39	195.74	75.386	-14.894	-33.152
Juncus_articulatus	585.45	-643.86	31.023	2.2618	-0.84132
Juncus_bufonius_agg.	585.45	-643.86	31.023	2.2618	-0.84132
Lactuca_serriola	-505.48	195.96	356.22	40.138	24.144
Leontodon_autumnalis	-513.38	196.01	365.47	42.021	26.271
Leontodon_taraxacoides	-513.38	196.01	365.47	42.021	26.271
Lepidium_ruderale	-161.94	186.68	-185.81	38.801	-62.72
Leucanthemum_vulgare_agg.	-466.36	195.7	310.17	30.706	13.365
Linaria_vulgaris	-188.02	189.01	-71.65	-65.439	-222.78
Lolium_perenne	601.09	-803.73	42.413	5.084	4.9664
Lotus_corniculatus_agg.	-241.8	179.41	-307.66	175.93	70.698
Lupinus_luteus	-231.62	180.44	-293.1	160.67	57.821
Lupinus_polyphyllus	-231.62	180.44	-293.1	160.67	57.821
Luzula_multiflora	585.39	-642.39	30.964	2.2554	-0.82375
Malus_domestica	-215.6	181.51	-264.79	123.83	8.6078
Matricaria_recutita	-468.85	195.67	312.73	31.158	13.725
Medicago_lupulina	-246.46	178.96	-314.53	183.35	77.287
Melica_nutans_agg.	600.02	-792.18	41.619	4.8925	4.5926
Moehringia_trinervia	-233.17	182.22	-163.4	-338.57	89.102
Moos_folios	3410	2275.9	205.68	27.59	27.059
Moos_thallos	3947.4	2652.2	250.02	35.525	41.285
Myosotis	-157.97	193.08	-60.22	-48.144	-102.35
Oenothera_parviflora_agg.	-160.22	186.9	-183.96	38.036	-59.383
Ornithopus_perpusillus	-241.8	179.41	-307.66	175.93	70.698
Papaver_dubium	141.64	214.15	-71.12	-20.091	-40.319
Papaver_rhoeas	141.64	214.15	-71.12	-20.091	-40.319
Petrorhagia_prolifera	-227.9	182.84	-159.56	-325.21	82.708
Phalaris_arundinacea	601.5	-808.09	42.715	5.1568	5.1094
Phragmites_australis	599.77	-789.71	41.441	4.8482	4.5009
Picris_hieracioides	-513.05	196.01	365.09	41.943	26.183
Pinus_sylvestris	2065.5	1334.3	95.022	8.1552	-3.4798
Plantago_lanceolata	-194.1	188.14	-73.879	-68.669	-243.59
Plantago_major	-195.11	188	-74.248	-69.201	-246.98
Plantago_major_subsp_intermedia	-195.11	188	-74.248	-69.201	-246.98
Poa_annua	601.75	-810.74	42.898	5.2012	5.1968
Poa_compressa	601.75	-810.74	42.898	5.2012	5.1968
Poa_palustris	601.75	-810.74	42.898	5.2012	5.1968
Poa_pratensis_agg.	601.75	-810.74	42.898	5.2012	5.1968
Polygonum_aviculare_agg.	-197.24	186.05	-135.3	-233.04	28.527
Polygonum_persicaria	-197.24	186.05	-135.3	-233.04	28.527
Populus_tremula	-199.78	183	-241.25	98.29	-12.713
Populus_x_generosa	-199.78	183	-241.25	98.29	-12.713
Potentilla_argentea	-218.44	181.15	-268.21	126.6	8.9615
Prunella_vulgaris	-190.98	188.57	-72.698	-66.893	-231.37
Prunus	-214.34	181.67	-263.31	122.67	8.4658
Robinia_pseudoacacia	-240.58	179.55	-306.03	174.35	69.547
Rubus_fruticosus_agg.	-217.02	181.34	-266.52	125.25	8.792
Rubus_idaeus	-217.02	181.34	-266.52	125.25	8.792
Rumex_acetosa	-199.49	185.74	-136.73	-237.1	29.429
Rumex_acetosella	-199.49	185.74	-136.73	-237.1	29.429
Rumex_crispus	-199.49	185.74	-136.73	-237.1	29.429
Rumex_thyrsiflorus	-199.49	185.74	-136.73	-237.1	29.429
Sagina_procumbens	-231.68	182.4	-162.32	-334.8	87.302
Salix_caprea	-200.58	182.89	-242.11	98.883	-12.858
Salix_viminalis	-200.58	182.89	-242.11	98.883	-12.858
Salix_x_rubens	-200.58	182.89	-242.11	98.883	-12.858
Salsola_kali_subsp._ruthenica	-216.08	184.2	-150.78	-294.26	67.437
Scleranthus_annuus	-232.38	182.31	-162.82	-336.53	88.116
Scleranthus_perennis	-232.38	182.31	-162.82	-336.53	88.116
Senecio_vernalis	-474.78	195.75	319.99	32.703	15.619

Senecio_viscosus	-474.78	195.75	319.99	32.703	15.619
Senecio_vulgaris	-474.78	195.75	319.99	32.703	15.619
Setaria_pumila	599.89	-791.02	41.528	4.8685	4.5384
Setaria_viridis	599.89	-791.02	41.528	4.8685	4.5384
Silene_alba	-227.9	182.84	-159.56	-325.21	82.708
Sinapis_arvensis	-163.34	186.46	-187.04	39.22	-64.005
Sisymbrium_altissimum	-164.74	186.23	-188.27	39.638	-65.282
Sisymbrium_officinale	-164.74	186.23	-188.27	39.638	-65.282
Solanum_nigrum	-156.41	193.33	-59.711	-47.521	-99.863
Solidago_canadensis	-492.95	195.88	341.5	37.129	20.721
Sonchus_arvensis_agg.	-509.59	195.99	361.03	41.115	25.244
Sonchus_asper	-509.59	195.99	361.03	41.115	25.244
Spergularia_rubra	-221.86	183.56	-155.18	-310.14	75.611
Stellaria_media_agg.	-233.43	182.19	-163.6	-339.24	89.425
Tanacetum_vulgare	-468.02	195.68	311.88	31.008	13.606
Taraxacum_officinale_agg.	-513.71	196.01	365.87	42.103	26.365
Torilis_japonica_agg.	-190.15	194.82	-6.3386	-32.312	-54.346
Tragopogon_dubius	-496.59	195.91	345.84	38.03	21.773
Trifolium_arvense	-257.54	177.81	-330.15	199.51	90.583
Trifolium_campestre	-257.54	177.81	-330.15	199.51	90.583
Trifolium_dubium_agg.	-257.54	177.81	-330.15	199.51	90.583
Trifolium_pratense	-257.54	177.81	-330.15	199.51	90.583
Trifolium_repens	-257.54	177.81	-330.15	199.51	90.583
Tripleurospermum_maritimum_agg.	-466.08	195.7	309.87	30.652	13.32
Tussilago_farfara	-472.21	195.77	317.3	32.214	15.196
Typha	568.46	-444.03	18.196	-0.65225	-5.7862
Ulmus	-198.49	183.64	-244.13	107.05	6.4486
Verbascum_sp	-187.77	189.03	-71.538	-65.241	-221.07
Veronica_chamaedrys_agg.	-193.59	188.21	-73.69	-68.391	-241.76
Veronica_officinalis	-193.59	188.21	-73.69	-68.391	-241.76
Vicia_angustifolia	-256.62	177.91	-328.83	198.14	89.442
Vicia_hirsuta	-256.62	177.91	-328.83	198.14	89.442
Vicia_tetrasperma_agg.	-256.62	177.91	-328.83	198.14	89.442
Vicia_villosa	-256.62	177.91	-328.83	198.14	89.442
Viola_arvensis	-190.23	184.23	-230.19	90.254	-10.519
Viola_canina_agg.	-190.23	184.23	-230.19	90.254	-10.519
Viola_tricolor_agg.	-190.23	184.23	-230.19	90.254	-10.519

Phylogenetic distance matrix according to the classification contained in APG III (Angiosperm Phylogeny Group 2009)

S	Achillea_pa	Agrostis_ca	Agrostis_st	Agrostis_vi	Ajuga_gene	Apera_spic	Arctium_m	Arenaria_s	Artemisia_	Artemisia_
Achillea_pannonica	0.0	179.0	179.0	179.0	117.0	179.0	44.0	122.0	32.7	32.7
Agrostis_capillaris	179.0	0.0	2.5	2.5	179.0	6.7	179.0	179.0	179.0	179.0
Agrostis_stolonifera_agg.	179.0	2.5	0.0	2.5	179.0	6.7	179.0	179.0	179.0	179.0
Agrostis_vinealis	179.0	2.5	2.5	0.0	179.0	6.7	179.0	179.0	179.0	179.0
Ajuga_genevensis	117.0	179.0	179.0	179.0	0.0	179.0	117.0	122.0	117.0	117.0
Apera_spica_venti	179.0	6.7	6.7	6.7	179.0	0.0	179.0	179.0	179.0	179.0
Arctium_minus_agg.	44.0	179.0	179.0	179.0	117.0	179.0	0.0	122.0	44.0	44.0
Arenaria_serpyllifolia_agg.	122.0	179.0	179.0	179.0	122.0	179.0	122.0	0.0	122.0	122.0
Artemisia_campestris_agg.	32.7	179.0	179.0	179.0	117.0	179.0	44.0	122.0	0.0	10.9
Artemisia_vulgaris_agg.	32.7	179.0	179.0	179.0	117.0	179.0	44.0	122.0	10.9	0.0
Berteroa_incana	127.0	179.0	179.0	179.0	127.0	179.0	127.0	127.0	127.0	127.0
Betula_pendula	127.0	179.0	179.0	179.0	127.0	179.0	127.0	127.0	127.0	127.0
Brachypodium_sylvaticum	179.0	9.3	9.3	9.3	179.0	9.3	179.0	179.0	179.0	179.0
Bromus_hordeaceus	179.0	8.0	8.0	8.0	179.0	8.0	179.0	179.0	179.0	179.0
Bromus_tectorum	179.0	8.0	8.0	8.0	179.0	8.0	179.0	179.0	179.0	179.0
Calamagrostis_epigejos	179.0	5.0	5.0	5.0	179.0	6.7	179.0	179.0	179.0	179.0
Carex_arenaria_agg.	179.0	42.0	42.0	42.0	179.0	42.0	179.0	179.0	179.0	179.0
Carex_ericetorum	179.0	42.0	42.0	42.0	179.0	42.0	179.0	179.0	179.0	179.0
Carex_hirta	179.0	42.0	42.0	42.0	179.0	42.0	179.0	179.0	179.0	179.0
Carex_spicata	179.0	42.0	42.0	42.0	179.0	42.0	179.0	179.0	179.0	179.0
Centaurea_stoebe	44.0	179.0	179.0	179.0	117.0	179.0	14.7	122.0	44.0	44.0
Centaurium_erythraea	117.0	179.0	179.0	179.0	107.0	179.0	117.0	122.0	117.0	117.0
Cerastium_holosteoides	122.0	179.0	179.0	179.0	122.0	179.0	122.0	19.3	122.0	122.0
Cerastium_pumilum_agg.	122.0	179.0	179.0	179.0	122.0	179.0	122.0	19.3	122.0	122.0
Chenopodium_album_agg.	122.0	179.0	179.0	179.0	122.0	179.0	122.0	51.5	122.0	122.0
Chenopodium_polyspermum	122.0	179.0	179.0	179.0	122.0	179.0	122.0	51.5	122.0	122.0
Chondrilla_juncea	40.9	179.0	179.0	179.0	117.0	179.0	44.0	122.0	40.9	40.9
Cirsium_arvense	44.0	179.0	179.0	179.0	117.0	179.0	29.3	122.0	44.0	44.0
Cirsium_vulgare	44.0	179.0	179.0	179.0	117.0	179.0	29.3	122.0	44.0	44.0
Convolvulus_arvensis	117.0	179.0	179.0	179.0	107.0	179.0	117.0	122.0	117.0	117.0
Conyza_canadensis	40.9	179.0	179.0	179.0	117.0	179.0	44.0	122.0	40.9	40.9
Corynephorus_canescens	179.0	5.0	5.0	5.0	179.0	6.7	179.0	179.0	179.0	179.0
Crataegus_monogyna	127.0	179.0	179.0	179.0	127.0	179.0	127.0	127.0	127.0	127.0
Crepis_capillaris	40.9	179.0	179.0	179.0	117.0	179.0	44.0	122.0	40.9	40.9
Crepis_foetida	40.9	179.0	179.0	179.0	117.0	179.0	44.0	122.0	40.9	40.9
Crepis_tectorum	40.9	179.0	179.0	179.0	117.0	179.0	44.0	122.0	40.9	40.9
Dactylis_glomerata_agg.	179.0	6.7	6.7	6.7	179.0	5.3	179.0	179.0	179.0	179.0
Danthonia_decumbens	179.0	12.0	12.0	12.0	179.0	12.0	179.0	179.0	179.0	179.0
Daucus_carota	107.0	179.0	179.0	179.0	117.0	179.0	107.0	122.0	107.0	107.0
Deschampsia_cespitosa_agg.	179.0	6.7	6.7	6.7	179.0	5.3	179.0	179.0	179.0	179.0
Digitaria_ischaemum	179.0	12.0	12.0	12.0	179.0	12.0	179.0	179.0	179.0	179.0
Digitaria_sanguinalis	179.0	12.0	12.0	12.0	179.0	12.0	179.0	179.0	179.0	179.0
Echinochloa_crus_galli	179.0	12.0	12.0	12.0	179.0	12.0	179.0	179.0	179.0	179.0
Echium_vulgare	117.0	179.0	179.0	179.0	107.0	179.0	117.0	122.0	117.0	117.0
Elymus_repens	179.0	8.0	8.0	8.0	179.0	8.0	179.0	179.0	179.0	179.0
Epilobium_ciliatum	127.0	179.0	179.0	179.0	127.0	179.0	127.0	127.0	127.0	127.0
Epilobium_hirsutum	127.0	179.0	179.0	179.0	127.0	179.0	127.0	127.0	127.0	127.0
Epilobium_tetragonum	127.0	179.0	179.0	179.0	127.0	179.0	127.0	127.0	127.0	127.0
Equisetum_arvense	400.0	400.0	400.0	400.0	400.0	400.0	400.0	400.0	400.0	400.0
Erigeron_annuus	40.9	179.0	179.0	179.0	117.0	179.0	44.0	122.0	40.9	40.9
Erodium_cicutarium_agg.	127.0	179.0	179.0	179.0	127.0	179.0	127.0	127.0	127.0	127.0
Eupatorium_cannabinum	40.9	179.0	179.0	179.0	117.0	179.0	44.0	122.0	40.9	40.9
Festuca_gigantea	179.0	6.7	6.7	6.7	179.0	5.3	179.0	179.0	179.0	179.0
Festuca_ovina_agg.	179.0	6.7	6.7	6.7	179.0	5.3	179.0	179.0	179.0	179.0
Festuca_rubra_agg.	179.0	6.7	6.7	6.7	179.0	5.3	179.0	179.0	179.0	179.0
Filago_arvensis	40.9	179.0	179.0	179.0	117.0	179.0	44.0	122.0	40.9	40.9
Filago_minima	40.9	179.0	179.0	179.0	117.0	179.0	44.0	122.0	40.9	40.9
Fragaria_vesca	127.0	179.0	179.0	179.0	127.0	179.0	127.0	127.0	127.0	127.0
Galeopsis_segetum	117.0	179.0	179.0	179.0	23.0	179.0	117.0	122.0	117.0	117.0
Genista_pilosa	127.0	179.0	179.0	179.0	127.0	179.0	127.0	127.0	127.0	127.0
Geum_urbanum	127.0	179.0	179.0	179.0	127.0	179.0	127.0	127.0	127.0	127.0
Gnaphalium_sylvaticum	40.9	179.0	179.0	179.0	117.0	179.0	44.0	122.0	40.9	40.9
Helichrysum_arenarium	40.9	179.0	179.0	179.0	117.0	179.0	44.0	122.0	40.9	40.9

Helictotrichon_pubescens	179.0	6.7	6.7	6.7	179.0	5.3	179.0	179.0	179.0	179.0
Herniaria_glabra	122.0	179.0	179.0	179.0	122.0	179.0	122.0	45.1	122.0	122.0
Hieracium_bauhini	40.9	179.0	179.0	179.0	117.0	179.0	44.0	122.0	40.9	40.9
Hieracium_pilosella	40.9	179.0	179.0	179.0	117.0	179.0	44.0	122.0	40.9	40.9
Hieracium_piloselloides	40.9	179.0	179.0	179.0	117.0	179.0	44.0	122.0	40.9	40.9
Hieracium_sabaudum	40.9	179.0	179.0	179.0	117.0	179.0	44.0	122.0	40.9	40.9
Hieracium_umbellatum	40.9	179.0	179.0	179.0	117.0	179.0	44.0	122.0	40.9	40.9
Hippophae_rhamnoides	127.0	179.0	179.0	179.0	127.0	179.0	127.0	127.0	127.0	127.0
Holcus_lanatus	179.0	6.7	6.7	6.7	179.0	5.3	179.0	179.0	179.0	179.0
Holcus_mollis	179.0	6.7	6.7	6.7	179.0	5.3	179.0	179.0	179.0	179.0
Hordeum_jubatum	179.0	8.0	8.0	8.0	179.0	8.0	179.0	179.0	179.0	179.0
Hordeum_vulgare	179.0	8.0	8.0	8.0	179.0	8.0	179.0	179.0	179.0	179.0
Hypericum_perforatum	127.0	179.0	179.0	179.0	127.0	179.0	127.0	127.0	127.0	127.0
Hypochaeris_radicata	40.9	179.0	179.0	179.0	117.0	179.0	44.0	122.0	40.9	40.9
Jasione_montana	90.0	179.0	179.0	179.0	117.0	179.0	90.0	122.0	90.0	90.0
Juncus_articulatus	179.0	42.0	42.0	42.0	179.0	42.0	179.0	179.0	179.0	179.0
Juncus_bufonius_agg.	179.0	42.0	42.0	42.0	179.0	42.0	179.0	179.0	179.0	179.0
Lactuca_serriola	40.9	179.0	179.0	179.0	117.0	179.0	44.0	122.0	40.9	40.9
Leontodon_autumnalis	40.9	179.0	179.0	179.0	117.0	179.0	44.0	122.0	40.9	40.9
Leontodon_taraxacoides	40.9	179.0	179.0	179.0	117.0	179.0	44.0	122.0	40.9	40.9
Lepidium_ruderale	127.0	179.0	179.0	179.0	127.0	179.0	127.0	127.0	127.0	127.0
Leucanthemum_vulgare_agg.	24.5	179.0	179.0	179.0	117.0	179.0	44.0	122.0	32.7	32.7
Linaria_vulgaris	117.0	179.0	179.0	179.0	63.0	179.0	117.0	122.0	117.0	117.0
Lolium_perenne	179.0	8.0	8.0	8.0	179.0	8.0	179.0	179.0	179.0	179.0
Lotus_corniculatus_agg.	127.0	179.0	179.0	179.0	127.0	179.0	127.0	127.0	127.0	127.0
Lupinus_luteus	127.0	179.0	179.0	179.0	127.0	179.0	127.0	127.0	127.0	127.0
Lupinus_polyphyllus	127.0	179.0	179.0	179.0	127.0	179.0	127.0	127.0	127.0	127.0
Luzula_multiflora	179.0	42.0	42.0	42.0	179.0	42.0	179.0	179.0	179.0	179.0
Malus_domestica	127.0	179.0	179.0	179.0	127.0	179.0	127.0	127.0	127.0	127.0
Matricaria_recutita	8.2	179.0	179.0	179.0	117.0	179.0	44.0	122.0	32.7	32.7
Medicago_lupulina	127.0	179.0	179.0	179.0	127.0	179.0	127.0	127.0	127.0	127.0
Melica_nutans_agg.	179.0	10.7	10.7	10.7	179.0	10.7	179.0	179.0	179.0	179.0
Moehringia_trinervia	122.0	179.0	179.0	179.0	122.0	179.0	122.0	9.7	122.0	122.0
Moos_folios	450.0	450.0	450.0	450.0	450.0	450.0	450.0	450.0	450.0	450.0
Moos_thallos	500.0	500.0	500.0	500.0	500.0	500.0	500.0	500.0	500.0	500.0
Myosotis	117.0	179.0	179.0	179.0	107.0	179.0	117.0	122.0	117.0	117.0
Oenothera_parviflora_agg.	127.0	179.0	179.0	179.0	127.0	179.0	127.0	127.0	127.0	127.0
Ornithopus_perpusillus	127.0	179.0	179.0	179.0	127.0	179.0	127.0	127.0	127.0	127.0
Papaver_dubium	147.0	179.0	179.0	179.0	147.0	179.0	147.0	147.0	147.0	147.0
Papaver_rhoeas	147.0	179.0	179.0	179.0	147.0	179.0	147.0	147.0	147.0	147.0
Petrorhagia_prolifera	122.0	179.0	179.0	179.0	122.0	179.0	122.0	32.2	122.0	122.0
Phalaris_arundinacea	179.0	5.0	5.0	5.0	179.0	6.7	179.0	179.0	179.0	179.0
Phragmites_australis	179.0	12.0	12.0	12.0	179.0	12.0	179.0	179.0	179.0	179.0
Picris_hieracioides	40.9	179.0	179.0	179.0	117.0	179.0	44.0	122.0	40.9	40.9
Pinus_sylvestris	325.0	325.0	325.0	325.0	325.0	325.0	325.0	325.0	325.0	325.0
Plantago_lanceolata	117.0	179.0	179.0	179.0	63.0	179.0	117.0	122.0	117.0	117.0
Plantago_major	117.0	179.0	179.0	179.0	63.0	179.0	117.0	122.0	117.0	117.0
Plantago_major_subsp_intermedia	117.0	179.0	179.0	179.0	63.0	179.0	117.0	122.0	117.0	117.0
Poa_annua	179.0	6.7	6.7	6.7	179.0	3.6	179.0	179.0	179.0	179.0
Poa_compressa	179.0	6.7	6.7	6.7	179.0	3.6	179.0	179.0	179.0	179.0
Poa_palustris	179.0	6.7	6.7	6.7	179.0	3.6	179.0	179.0	179.0	179.0
Poa_pratensis_agg.	179.0	6.7	6.7	6.7	179.0	3.6	179.0	179.0	179.0	179.0
Polygonum_aviculare_agg.	122.0	179.0	179.0	179.0	122.0	179.0	122.0	84.0	122.0	122.0
Polygonum_persicaria	122.0	179.0	179.0	179.0	122.0	179.0	122.0	84.0	122.0	122.0
Populus_tremula	127.0	179.0	179.0	179.0	127.0	179.0	127.0	127.0	127.0	127.0
Populus_x_generosa	127.0	179.0	179.0	179.0	127.0	179.0	127.0	127.0	127.0	127.0
Potentilla_argentea	127.0	179.0	179.0	179.0	127.0	179.0	127.0	127.0	127.0	127.0
Prunella_vulgaris	117.0	179.0	179.0	179.0	23.0	179.0	117.0	122.0	117.0	117.0
Prunus	127.0	179.0	179.0	179.0	127.0	179.0	127.0	127.0	127.0	127.0
Robinia_pseudoacacia	127.0	179.0	179.0	179.0	127.0	179.0	127.0	127.0	127.0	127.0
Rubus_fruticosus_agg.	127.0	179.0	179.0	179.0	127.0	179.0	127.0	127.0	127.0	127.0
Rubus_idaeus	127.0	179.0	179.0	179.0	127.0	179.0	127.0	127.0	127.0	127.0
Rumex_acetosa	122.0	179.0	179.0	179.0	122.0	179.0	122.0	84.0	122.0	122.0
Rumex_acetosella	122.0	179.0	179.0	179.0	122.0	179.0	122.0	84.0	122.0	122.0
Rumex_crispus	122.0	179.0	179.0	179.0	122.0	179.0	122.0	84.0	122.0	122.0
Rumex_thyrsiflorus	122.0	179.0	179.0	179.0	122.0	179.0	122.0	84.0	122.0	122.0
Sagina_procumbens	122.0	179.0	179.0	179.0	122.0	179.0	122.0	25.8	122.0	122.0
Salix_caprea	127.0	179.0	179.0	179.0	127.0	179.0	127.0	127.0	127.0	127.0
Salix_viminalis	127.0	179.0	179.0	179.0	127.0	179.0	127.0	127.0	127.0	127.0
Salix_x_rubens	127.0	179.0	179.0	179.0	127.0	179.0	127.0	127.0	127.0	127.0
Salsola_kali_subsp._ruthenica	122.0	179.0	179.0	179.0	122.0	179.0	122.0	51.5	122.0	122.0
Scleranthus_annuus	122.0	179.0	179.0	179.0	122.0	179.0	122.0	25.8	122.0	122.0
Scleranthus_perennis	122.0	179.0	179.0	179.0	122.0	179.0	122.0	25.8	122.0	122.0
Senecio_vernalis	40.9	179.0	179.0	179.0	117.0	179.0	44.0	122.0	40.9	40.9

Senecio_viscosus	40.9	179.0	179.0	179.0	117.0	179.0	44.0	122.0	40.9	40.9
Senecio_vulgaris	40.9	179.0	179.0	179.0	117.0	179.0	44.0	122.0	40.9	40.9
Setaria_pumila	179.0	12.0	12.0	12.0	179.0	12.0	179.0	179.0	179.0	179.0
Setaria_viridis	179.0	12.0	12.0	12.0	179.0	12.0	179.0	179.0	179.0	179.0
Silene_alba	122.0	179.0	179.0	179.0	122.0	179.0	122.0	32.2	122.0	122.0
Sinapis_arvensis	127.0	179.0	179.0	179.0	127.0	179.0	127.0	127.0	127.0	127.0
Sisymbrium_altissimum	127.0	179.0	179.0	179.0	127.0	179.0	127.0	127.0	127.0	127.0
Sisymbrium_officinale	127.0	179.0	179.0	179.0	127.0	179.0	127.0	127.0	127.0	127.0
Solanum_nigrum	117.0	179.0	179.0	179.0	107.0	179.0	117.0	122.0	117.0	117.0
Solidago_canadensis	40.9	179.0	179.0	179.0	117.0	179.0	44.0	122.0	40.9	40.9
Sonchus_arvensis_agg.	40.9	179.0	179.0	179.0	117.0	179.0	44.0	122.0	40.9	40.9
Sonchus_asper	40.9	179.0	179.0	179.0	117.0	179.0	44.0	122.0	40.9	40.9
Spergularia_rubra	122.0	179.0	179.0	179.0	122.0	179.0	122.0	38.6	122.0	122.0
Stellaria_media_agg.	122.0	179.0	179.0	179.0	122.0	179.0	122.0	19.3	122.0	122.0
Tanacetum_vulgare	16.3	179.0	179.0	179.0	117.0	179.0	44.0	122.0	32.7	32.7
Taraxacum_officinale_agg.	40.9	179.0	179.0	179.0	117.0	179.0	44.0	122.0	40.9	40.9
Torilis_japonica_agg.	107.0	179.0	179.0	179.0	117.0	179.0	107.0	122.0	107.0	107.0
Tragopogon_dubius	40.9	179.0	179.0	179.0	117.0	179.0	44.0	122.0	40.9	40.9
Trifolium_arvense	127.0	179.0	179.0	179.0	127.0	179.0	127.0	127.0	127.0	127.0
Trifolium_campestre	127.0	179.0	179.0	179.0	127.0	179.0	127.0	127.0	127.0	127.0
Trifolium_dubium_agg.	127.0	179.0	179.0	179.0	127.0	179.0	127.0	127.0	127.0	127.0
Trifolium_pratense	127.0	179.0	179.0	179.0	127.0	179.0	127.0	127.0	127.0	127.0
Trifolium_repens	127.0	179.0	179.0	179.0	127.0	179.0	127.0	127.0	127.0	127.0
Tripleurospermum_maritimum_agg.	32.7	179.0	179.0	179.0	117.0	179.0	44.0	122.0	21.8	21.8
Tussilago_farfara	40.9	179.0	179.0	179.0	117.0	179.0	44.0	122.0	40.9	40.9
Typha	179.0	72.0	72.0	72.0	179.0	72.0	179.0	179.0	179.0	179.0
Ulmus	127.0	179.0	179.0	179.0	127.0	179.0	127.0	127.0	127.0	127.0
Verbascum_sp	117.0	179.0	179.0	179.0	43.0	179.0	117.0	122.0	117.0	117.0
Veronica_chamaedrys_agg.	117.0	179.0	179.0	179.0	63.0	179.0	117.0	122.0	117.0	117.0
Veronica_officinalis	117.0	179.0	179.0	179.0	63.0	179.0	117.0	122.0	117.0	117.0
Vicia_angustifolia	127.0	179.0	179.0	179.0	127.0	179.0	127.0	127.0	127.0	127.0
Vicia_hirsuta	127.0	179.0	179.0	179.0	127.0	179.0	127.0	127.0	127.0	127.0
Vicia_tetrasperma_agg.	127.0	179.0	179.0	179.0	127.0	179.0	127.0	127.0	127.0	127.0
Vicia_villosa	127.0	179.0	179.0	179.0	127.0	179.0	127.0	127.0	127.0	127.0
Viola_arvensis	127.0	179.0	179.0	179.0	127.0	179.0	127.0	127.0	127.0	127.0
Viola_canina_agg.	127.0	179.0	179.0	179.0	127.0	179.0	127.0	127.0	127.0	127.0
Viola_tricolor_agg.	127.0	179.0	179.0	179.0	127.0	179.0	127.0	127.0	127.0	127.0

Berteroa_i	Betula_per	Brachypodi	Bromus_hc	Bromus_te	Calamagros	Carex_arer	Carex_eric	Carex_hirta	Carex_spic	Centaurea_	Centauriun	Cerastium_	Cerastium_
127.0	127.0	179.0	179.0	179.0	179.0	179.0	179.0	179.0	179.0	44.0	117.0	122.0	122.0
179.0	179.0	9.3	8.0	8.0	5.0	42.0	42.0	42.0	42.0	179.0	179.0	179.0	179.0
179.0	179.0	9.3	8.0	8.0	5.0	42.0	42.0	42.0	42.0	179.0	179.0	179.0	179.0
179.0	179.0	9.3	8.0	8.0	5.0	42.0	42.0	42.0	42.0	179.0	179.0	179.0	179.0
127.0	127.0	179.0	179.0	179.0	179.0	179.0	179.0	179.0	179.0	117.0	107.0	122.0	122.0
179.0	179.0	9.3	8.0	8.0	6.7	42.0	42.0	42.0	42.0	179.0	179.0	179.0	179.0
127.0	127.0	179.0	179.0	179.0	179.0	179.0	179.0	179.0	179.0	14.7	117.0	122.0	122.0
127.0	127.0	179.0	179.0	179.0	179.0	179.0	179.0	179.0	179.0	122.0	122.0	19.3	19.3
127.0	127.0	179.0	179.0	179.0	179.0	179.0	179.0	179.0	179.0	44.0	117.0	122.0	122.0
127.0	127.0	179.0	179.0	179.0	179.0	179.0	179.0	179.0	179.0	44.0	117.0	122.0	122.0
0.0	112.5	179.0	179.0	179.0	179.0	179.0	179.0	179.0	179.0	127.0	127.0	127.0	127.0
112.5	0.0	179.0	179.0	179.0	179.0	179.0	179.0	179.0	179.0	127.0	127.0	127.0	127.0
179.0	179.0	0.0	9.3	9.3	9.3	42.0	42.0	42.0	42.0	179.0	179.0	179.0	179.0
179.0	179.0	9.3	0.0	2.4	8.0	42.0	42.0	42.0	42.0	179.0	179.0	179.0	179.0
179.0	179.0	9.3	2.4	0.0	8.0	42.0	42.0	42.0	42.0	179.0	179.0	179.0	179.0
179.0	179.0	9.3	8.0	8.0	0.0	42.0	42.0	42.0	42.0	179.0	179.0	179.0	179.0
179.0	179.0	42.0	42.0	42.0	42.0	0.0	15.8	15.8	15.8	179.0	179.0	179.0	179.0
179.0	179.0	42.0	42.0	42.0	42.0	15.8	0.0	15.8	15.8	179.0	179.0	179.0	179.0
179.0	179.0	42.0	42.0	42.0	42.0	15.8	15.8	0.0	15.8	179.0	179.0	179.0	179.0
179.0	179.0	42.0	42.0	42.0	42.0	15.8	15.8	15.8	0.0	179.0	179.0	179.0	179.0
127.0	127.0	179.0	179.0	179.0	179.0	179.0	179.0	179.0	179.0	0.0	117.0	122.0	122.0
127.0	127.0	179.0	179.0	179.0	179.0	179.0	179.0	179.0	179.0	117.0	0.0	122.0	122.0
127.0	127.0	179.0	179.0	179.0	179.0	179.0	179.0	179.0	179.0	122.0	122.0	0.0	6.4
127.0	127.0	179.0	179.0	179.0	179.0	179.0	179.0	179.0	179.0	122.0	122.0	6.4	0.0
127.0	127.0	179.0	179.0	179.0	179.0	179.0	179.0	179.0	179.0	122.0	122.0	51.5	51.5
127.0	127.0	179.0	179.0	179.0	179.0	179.0	179.0	179.0	179.0	122.0	122.0	51.5	51.5
127.0	127.0	179.0	179.0	179.0	179.0	179.0	179.0	179.0	179.0	44.0	117.0	122.0	122.0
127.0	127.0	179.0	179.0	179.0	179.0	179.0	179.0	179.0	179.0	29.3	117.0	122.0	122.0
127.0	127.0	179.0	179.0	179.0	179.0	179.0	179.0	179.0	179.0	29.3	117.0	122.0	122.0
127.0	127.0	179.0	179.0	179.0	179.0	179.0	179.0	179.0	179.0	117.0	87.7	122.0	122.0
127.0	127.0	179.0	179.0	179.0	179.0	179.0	179.0	179.0	179.0	44.0	117.0	122.0	122.0
179.0	179.0	9.3	8.0	8.0	1.7	42.0	42.0	42.0	42.0	179.0	179.0	179.0	179.0
112.5	83.3	179.0	179.0	179.0	179.0	179.0	179.0	179.0	179.0	127.0	127.0	127.0	127.0
127.0	127.0	179.0	179.0	179.0	179.0	179.0	179.0	179.0	179.0	44.0	117.0	122.0	122.0
127.0	127.0	179.0	179.0	179.0	179.0	179.0	179.0	179.0	179.0	44.0	117.0	122.0	122.0
127.0	127.0	179.0	179.0	179.0	179.0	179.0	179.0	179.0	179.0	44.0	117.0	122.0	122.0
179.0	179.0	9.3	8.0	8.0	6.7	42.0	42.0	42.0	42.0	179.0	179.0	179.0	179.0
179.0	179.0	12.0	12.0	12.0	12.0	42.0	42.0	42.0	42.0	179.0	179.0	179.0	179.0
127.0	127.0	179.0	179.0	179.0	179.0	179.0	179.0	179.0	179.0	107.0	117.0	122.0	122.0
179.0	179.0	9.3	8.0	8.0	6.7	42.0	42.0	42.0	42.0	179.0	179.0	179.0	179.0
179.0	179.0	12.0	12.0	12.0	12.0	42.0	42.0	42.0	42.0	179.0	179.0	179.0	179.0
179.0	179.0	12.0	12.0	12.0	12.0	42.0	42.0	42.0	42.0	179.0	179.0	179.0	179.0
179.0	179.0	12.0	12.0	12.0	12.0	42.0	42.0	42.0	42.0	179.0	179.0	179.0	179.0
127.0	127.0	179.0	179.0	179.0	179.0	179.0	179.0	179.0	179.0	117.0	97.3	122.0	122.0
179.0	179.0	9.3	4.8	4.8	8.0	42.0	42.0	42.0	42.0	179.0	179.0	179.0	179.0
95.0	112.5	179.0	179.0	179.0	179.0	179.0	179.0	179.0	179.0	127.0	127.0	127.0	127.0
95.0	112.5	179.0	179.0	179.0	179.0	179.0	179.0	179.0	179.0	127.0	127.0	127.0	127.0
95.0	112.5	179.0	179.0	179.0	179.0	179.0	179.0	179.0	179.0	127.0	127.0	127.0	127.0
400.0	400.0	400.0	400.0	400.0	400.0	400.0	400.0	400.0	400.0	400.0	400.0	400.0	400.0
127.0	127.0	179.0	179.0	179.0	179.0	179.0	179.0	179.0	179.0	44.0	117.0	122.0	122.0
95.0	112.5	179.0	179.0	179.0	179.0	179.0	179.0	179.0	179.0	127.0	127.0	127.0	127.0
127.0	127.0	179.0	179.0	179.0	179.0	179.0	179.0	179.0	179.0	44.0	117.0	122.0	122.0
179.0	179.0	9.3	8.0	8.0	6.7	42.0	42.0	42.0	42.0	179.0	179.0	179.0	179.0
179.0	179.0	9.3	8.0	8.0	6.7	42.0	42.0	42.0	42.0	179.0	179.0	179.0	179.0
179.0	179.0	9.3	8.0	8.0	6.7	42.0	42.0	42.0	42.0	179.0	179.0	179.0	179.0
127.0	127.0	179.0	179.0	179.0	179.0	179.0	179.0	179.0	179.0	44.0	117.0	122.0	122.0
127.0	127.0	179.0	179.0	179.0	179.0	179.0	179.0	179.0	179.0	44.0	117.0	122.0	122.0
112.5	83.3	179.0	179.0	179.0	179.0	179.0	179.0	179.0	179.0	127.0	127.0	127.0	127.0
127.0	127.0	179.0	179.0	179.0	179.0	179.0	179.0	179.0	179.0	117.0	107.0	122.0	122.0
112.5	90.7	179.0	179.0	179.0	179.0	179.0	179.0	179.0	179.0	127.0	127.0	127.0	127.0
112.5	83.3	179.0	179.0	179.0	179.0	179.0	179.0	179.0	179.0	127.0	127.0	127.0	127.0
127.0	127.0	179.0	179.0	179.0	179.0	179.0	179.0	179.0	179.0	44.0	117.0	122.0	122.0
127.0	127.0	179.0	179.0	179.0	179.0	179.0	179.0	179.0	179.0	44.0	117.0	122.0	122.0

179.0	179.0	9.3	8.0	8.0	6.7	42.0	42.0	42.0	42.0	179.0	179.0	179.0	179.0
127.0	127.0	179.0	179.0	179.0	179.0	179.0	179.0	179.0	179.0	122.0	122.0	45.1	45.1
127.0	127.0	179.0	179.0	179.0	179.0	179.0	179.0	179.0	179.0	44.0	117.0	122.0	122.0
127.0	127.0	179.0	179.0	179.0	179.0	179.0	179.0	179.0	179.0	44.0	117.0	122.0	122.0
127.0	127.0	179.0	179.0	179.0	179.0	179.0	179.0	179.0	179.0	44.0	117.0	122.0	122.0
127.0	127.0	179.0	179.0	179.0	179.0	179.0	179.0	179.0	179.0	44.0	117.0	122.0	122.0
127.0	127.0	179.0	179.0	179.0	179.0	179.0	179.0	179.0	179.0	44.0	117.0	122.0	122.0
112.5	83.3	179.0	179.0	179.0	179.0	179.0	179.0	179.0	179.0	127.0	127.0	127.0	127.0
179.0	179.0	9.3	8.0	8.0	6.7	42.0	42.0	42.0	42.0	179.0	179.0	179.0	179.0
179.0	179.0	9.3	8.0	8.0	6.7	42.0	42.0	42.0	42.0	179.0	179.0	179.0	179.0
179.0	179.0	9.3	4.8	4.8	8.0	42.0	42.0	42.0	42.0	179.0	179.0	179.0	179.0
179.0	179.0	9.3	4.8	4.8	8.0	42.0	42.0	42.0	42.0	179.0	179.0	179.0	179.0
112.5	98.0	179.0	179.0	179.0	179.0	179.0	179.0	179.0	179.0	127.0	127.0	127.0	127.0
127.0	127.0	179.0	179.0	179.0	179.0	179.0	179.0	179.0	179.0	44.0	117.0	122.0	122.0
127.0	127.0	179.0	179.0	179.0	179.0	179.0	179.0	179.0	179.0	90.0	117.0	122.0	122.0
179.0	179.0	42.0	42.0	42.0	42.0	31.5	31.5	31.5	31.5	179.0	179.0	179.0	179.0
179.0	179.0	42.0	42.0	42.0	42.0	31.5	31.5	31.5	31.5	179.0	179.0	179.0	179.0
127.0	127.0	179.0	179.0	179.0	179.0	179.0	179.0	179.0	179.0	44.0	117.0	122.0	122.0
127.0	127.0	179.0	179.0	179.0	179.0	179.0	179.0	179.0	179.0	44.0	117.0	122.0	122.0
127.0	127.0	179.0	179.0	179.0	179.0	179.0	179.0	179.0	179.0	44.0	117.0	122.0	122.0
24.0	112.5	179.0	179.0	179.0	179.0	179.0	179.0	179.0	179.0	127.0	127.0	127.0	127.0
127.0	127.0	179.0	179.0	179.0	179.0	179.0	179.0	179.0	179.0	44.0	117.0	122.0	122.0
127.0	127.0	179.0	179.0	179.0	179.0	179.0	179.0	179.0	179.0	117.0	107.0	122.0	122.0
179.0	179.0	9.3	6.4	6.4	8.0	42.0	42.0	42.0	42.0	179.0	179.0	179.0	179.0
112.5	90.7	179.0	179.0	179.0	179.0	179.0	179.0	179.0	179.0	127.0	127.0	127.0	127.0
112.5	90.7	179.0	179.0	179.0	179.0	179.0	179.0	179.0	179.0	127.0	127.0	127.0	127.0
112.5	90.7	179.0	179.0	179.0	179.0	179.0	179.0	179.0	179.0	127.0	127.0	127.0	127.0
179.0	179.0	42.0	42.0	42.0	42.0	31.5	31.5	31.5	31.5	179.0	179.0	179.0	179.0
112.5	83.3	179.0	179.0	179.0	179.0	179.0	179.0	179.0	179.0	127.0	127.0	127.0	127.0
127.0	127.0	179.0	179.0	179.0	179.0	179.0	179.0	179.0	179.0	44.0	117.0	122.0	122.0
112.5	90.7	179.0	179.0	179.0	179.0	179.0	179.0	179.0	179.0	127.0	127.0	127.0	127.0
179.0	179.0	10.7	10.7	10.7	10.7	42.0	42.0	42.0	42.0	179.0	179.0	179.0	179.0
127.0	127.0	179.0	179.0	179.0	179.0	179.0	179.0	179.0	179.0	122.0	122.0	19.3	19.3
450.0	450.0	450.0	450.0	450.0	450.0	450.0	450.0	450.0	450.0	450.0	450.0	450.0	450.0
500.0	500.0	500.0	500.0	500.0	500.0	500.0	500.0	500.0	500.0	500.0	500.0	500.0	500.0
127.0	127.0	179.0	179.0	179.0	179.0	179.0	179.0	179.0	179.0	117.0	97.3	122.0	122.0
95.0	112.5	179.0	179.0	179.0	179.0	179.0	179.0	179.0	179.0	127.0	127.0	127.0	127.0
112.5	90.7	179.0	179.0	179.0	179.0	179.0	179.0	179.0	179.0	127.0	127.0	127.0	127.0
147.0	147.0	179.0	179.0	179.0	179.0	179.0	179.0	179.0	179.0	147.0	147.0	147.0	147.0
147.0	147.0	179.0	179.0	179.0	179.0	179.0	179.0	179.0	179.0	147.0	147.0	147.0	147.0
127.0	127.0	179.0	179.0	179.0	179.0	179.0	179.0	179.0	179.0	122.0	122.0	32.2	32.2
179.0	179.0	9.3	8.0	8.0	3.3	42.0	42.0	42.0	42.0	179.0	179.0	179.0	179.0
179.0	179.0	12.0	12.0	12.0	12.0	42.0	42.0	42.0	42.0	179.0	179.0	179.0	179.0
127.0	127.0	179.0	179.0	179.0	179.0	179.0	179.0	179.0	179.0	44.0	117.0	122.0	122.0
325.0	325.0	325.0	325.0	325.0	325.0	325.0	325.0	325.0	325.0	325.0	325.0	325.0	325.0
127.0	127.0	179.0	179.0	179.0	179.0	179.0	179.0	179.0	179.0	117.0	107.0	122.0	122.0
127.0	127.0	179.0	179.0	179.0	179.0	179.0	179.0	179.0	179.0	117.0	107.0	122.0	122.0
127.0	127.0	179.0	179.0	179.0	179.0	179.0	179.0	179.0	179.0	117.0	107.0	122.0	122.0
179.0	179.0	9.3	8.0	8.0	6.7	42.0	42.0	42.0	42.0	179.0	179.0	179.0	179.0
179.0	179.0	9.3	8.0	8.0	6.7	42.0	42.0	42.0	42.0	179.0	179.0	179.0	179.0
179.0	179.0	9.3	8.0	8.0	6.7	42.0	42.0	42.0	42.0	179.0	179.0	179.0	179.0
179.0	179.0	9.3	8.0	8.0	6.7	42.0	42.0	42.0	42.0	179.0	179.0	179.0	179.0
127.0	127.0	179.0	179.0	179.0	179.0	179.0	179.0	179.0	179.0	122.0	122.0	84.0	84.0
127.0	127.0	179.0	179.0	179.0	179.0	179.0	179.0	179.0	179.0	122.0	122.0	84.0	84.0
112.5	98.0	179.0	179.0	179.0	179.0	179.0	179.0	179.0	179.0	127.0	127.0	127.0	127.0
112.5	98.0	179.0	179.0	179.0	179.0	179.0	179.0	179.0	179.0	127.0	127.0	127.0	127.0
112.5	83.3	179.0	179.0	179.0	179.0	179.0	179.0	179.0	179.0	127.0	127.0	127.0	127.0
127.0	127.0	179.0	179.0	179.0	179.0	179.0	179.0	179.0	179.0	117.0	107.0	122.0	122.0
112.5	83.3	179.0	179.0	179.0	179.0	179.0	179.0	179.0	179.0	127.0	127.0	127.0	127.0
112.5	90.7	179.0	179.0	179.0	179.0	179.0	179.0	179.0	179.0	127.0	127.0	127.0	127.0
112.5	83.3	179.0	179.0	179.0	179.0	179.0	179.0	179.0	179.0	127.0	127.0	127.0	127.0
112.5	83.3	179.0	179.0	179.0	179.0	179.0	179.0	179.0	179.0	127.0	127.0	127.0	127.0
127.0	127.0	179.0	179.0	179.0	179.0	179.0	179.0	179.0	179.0	122.0	122.0	84.0	84.0
127.0	127.0	179.0	179.0	179.0	179.0	179.0	179.0	179.0	179.0	122.0	122.0	84.0	84.0
127.0	127.0	179.0	179.0	179.0	179.0	179.0	179.0	179.0	179.0	122.0	122.0	84.0	84.0
127.0	127.0	179.0	179.0	179.0	179.0	179.0	179.0	179.0	179.0	122.0	122.0	84.0	84.0
127.0	127.0	179.0	179.0	179.0	179.0	179.0	179.0	179.0	179.0	122.0	122.0	25.8	25.8
112.5	98.0	179.0	179.0	179.0	179.0	179.0	179.0	179.0	179.0	127.0	127.0	127.0	127.0
112.5	98.0	179.0	179.0	179.0	179.0	179.0	179.0	179.0	179.0	127.0	127.0	127.0	127.0
112.5	98.0	179.0	179.0	179.0	179.0	179.0	179.0	179.0	179.0	127.0	127.0	127.0	127.0
127.0	127.0	179.0	179.0	179.0	179.0	179.0	179.0	179.0	179.0	122.0	122.0	51.5	51.5
127.0	127.0	179.0	179.0	179.0	179.0	179.0	179.0	179.0	179.0	122.0	122.0	25.8	25.8
127.0	127.0	179.0	179.0	179.0	179.0	179.0	179.0	179.0	179.0	122.0	122.0	25.8	25.8
127.0	127.0	179.0	179.0	179.0	179.0	179.0	179.0	179.0	179.0	44.0	117.0	122.0	122.0

127.0	127.0	179.0	179.0	179.0	179.0	179.0	179.0	179.0	179.0	44.0	117.0	122.0	122.0
127.0	127.0	179.0	179.0	179.0	179.0	179.0	179.0	179.0	179.0	44.0	117.0	122.0	122.0
179.0	179.0	12.0	12.0	12.0	12.0	42.0	42.0	42.0	42.0	179.0	179.0	179.0	179.0
179.0	179.0	12.0	12.0	12.0	12.0	42.0	42.0	42.0	42.0	179.0	179.0	179.0	179.0
127.0	127.0	179.0	179.0	179.0	179.0	179.0	179.0	179.0	179.0	122.0	122.0	32.2	32.2
18.0	112.5	179.0	179.0	179.0	179.0	179.0	179.0	179.0	179.0	127.0	127.0	127.0	127.0
12.0	112.5	179.0	179.0	179.0	179.0	179.0	179.0	179.0	179.0	127.0	127.0	127.0	127.0
12.0	112.5	179.0	179.0	179.0	179.0	179.0	179.0	179.0	179.0	127.0	127.0	127.0	127.0
127.0	127.0	179.0	179.0	179.0	179.0	179.0	179.0	179.0	179.0	117.0	87.7	122.0	122.0
127.0	127.0	179.0	179.0	179.0	179.0	179.0	179.0	179.0	179.0	44.0	117.0	122.0	122.0
127.0	127.0	179.0	179.0	179.0	179.0	179.0	179.0	179.0	179.0	44.0	117.0	122.0	122.0
127.0	127.0	179.0	179.0	179.0	179.0	179.0	179.0	179.0	179.0	44.0	117.0	122.0	122.0
127.0	127.0	179.0	179.0	179.0	179.0	179.0	179.0	179.0	179.0	122.0	122.0	38.6	38.6
127.0	127.0	179.0	179.0	179.0	179.0	179.0	179.0	179.0	179.0	122.0	122.0	12.9	12.9
127.0	127.0	179.0	179.0	179.0	179.0	179.0	179.0	179.0	179.0	44.0	117.0	122.0	122.0
127.0	127.0	179.0	179.0	179.0	179.0	179.0	179.0	179.0	179.0	44.0	117.0	122.0	122.0
127.0	127.0	179.0	179.0	179.0	179.0	179.0	179.0	179.0	179.0	107.0	117.0	122.0	122.0
127.0	127.0	179.0	179.0	179.0	179.0	179.0	179.0	179.0	179.0	44.0	117.0	122.0	122.0
112.5	90.7	179.0	179.0	179.0	179.0	179.0	179.0	179.0	179.0	127.0	127.0	127.0	127.0
112.5	90.7	179.0	179.0	179.0	179.0	179.0	179.0	179.0	179.0	127.0	127.0	127.0	127.0
112.5	90.7	179.0	179.0	179.0	179.0	179.0	179.0	179.0	179.0	127.0	127.0	127.0	127.0
112.5	90.7	179.0	179.0	179.0	179.0	179.0	179.0	179.0	179.0	127.0	127.0	127.0	127.0
112.5	90.7	179.0	179.0	179.0	179.0	179.0	179.0	179.0	179.0	127.0	127.0	127.0	127.0
127.0	127.0	179.0	179.0	179.0	179.0	179.0	179.0	179.0	179.0	44.0	117.0	122.0	122.0
127.0	127.0	179.0	179.0	179.0	179.0	179.0	179.0	179.0	179.0	44.0	117.0	122.0	122.0
179.0	179.0	72.0	72.0	72.0	72.0	72.0	72.0	72.0	72.0	179.0	179.0	179.0	179.0
112.5	83.3	179.0	179.0	179.0	179.0	179.0	179.0	179.0	179.0	127.0	127.0	127.0	127.0
127.0	127.0	179.0	179.0	179.0	179.0	179.0	179.0	179.0	179.0	117.0	107.0	122.0	122.0
127.0	127.0	179.0	179.0	179.0	179.0	179.0	179.0	179.0	179.0	117.0	107.0	122.0	122.0
127.0	127.0	179.0	179.0	179.0	179.0	179.0	179.0	179.0	179.0	117.0	107.0	122.0	122.0
112.5	90.7	179.0	179.0	179.0	179.0	179.0	179.0	179.0	179.0	127.0	127.0	127.0	127.0
112.5	90.7	179.0	179.0	179.0	179.0	179.0	179.0	179.0	179.0	127.0	127.0	127.0	127.0
112.5	90.7	179.0	179.0	179.0	179.0	179.0	179.0	179.0	179.0	127.0	127.0	127.0	127.0
112.5	90.7	179.0	179.0	179.0	179.0	179.0	179.0	179.0	179.0	127.0	127.0	127.0	127.0
112.5	98.0	179.0	179.0	179.0	179.0	179.0	179.0	179.0	179.0	127.0	127.0	127.0	127.0
112.5	98.0	179.0	179.0	179.0	179.0	179.0	179.0	179.0	179.0	127.0	127.0	127.0	127.0
112.5	98.0	179.0	179.0	179.0	179.0	179.0	179.0	179.0	179.0	127.0	127.0	127.0	127.0

Chenopodi	Chenopodi	Chondrilla_	Cirsium_ar	Cirsium_vu	Convolvulu	Conyza_ca	Corynepho	Crataegus_	Crepis_cap	Crepis_foe	Crepis_tect	Dactylis_gl	Danthonia_
122.0	122.0	40.9	44.0	44.0	117.0	40.9	179.0	127.0	40.9	40.9	40.9	179.0	179.0
179.0	179.0	179.0	179.0	179.0	179.0	179.0	5.0	179.0	179.0	179.0	179.0	6.7	12.0
179.0	179.0	179.0	179.0	179.0	179.0	179.0	5.0	179.0	179.0	179.0	179.0	6.7	12.0
179.0	179.0	179.0	179.0	179.0	179.0	179.0	5.0	179.0	179.0	179.0	179.0	6.7	12.0
122.0	122.0	117.0	117.0	117.0	107.0	117.0	179.0	127.0	117.0	117.0	117.0	179.0	179.0
179.0	179.0	179.0	179.0	179.0	179.0	179.0	6.7	179.0	179.0	179.0	179.0	5.3	12.0
122.0	122.0	44.0	29.3	29.3	117.0	44.0	179.0	127.0	44.0	44.0	44.0	179.0	179.0
51.5	51.5	122.0	122.0	122.0	122.0	122.0	179.0	127.0	122.0	122.0	122.0	179.0	179.0
122.0	122.0	40.9	44.0	44.0	117.0	40.9	179.0	127.0	40.9	40.9	40.9	179.0	179.0
122.0	122.0	40.9	44.0	44.0	117.0	40.9	179.0	127.0	40.9	40.9	40.9	179.0	179.0
127.0	127.0	127.0	127.0	127.0	127.0	127.0	179.0	112.5	127.0	127.0	127.0	179.0	179.0
127.0	127.0	127.0	127.0	127.0	127.0	127.0	179.0	83.3	127.0	127.0	127.0	179.0	179.0
179.0	179.0	179.0	179.0	179.0	179.0	179.0	9.3	179.0	179.0	179.0	179.0	9.3	12.0
179.0	179.0	179.0	179.0	179.0	179.0	179.0	8.0	179.0	179.0	179.0	179.0	8.0	12.0
179.0	179.0	179.0	179.0	179.0	179.0	179.0	8.0	179.0	179.0	179.0	179.0	8.0	12.0
179.0	179.0	179.0	179.0	179.0	179.0	179.0	1.7	179.0	179.0	179.0	179.0	6.7	12.0
179.0	179.0	179.0	179.0	179.0	179.0	179.0	42.0	179.0	179.0	179.0	179.0	42.0	42.0
179.0	179.0	179.0	179.0	179.0	179.0	179.0	42.0	179.0	179.0	179.0	179.0	42.0	42.0
179.0	179.0	179.0	179.0	179.0	179.0	179.0	42.0	179.0	179.0	179.0	179.0	42.0	42.0
179.0	179.0	179.0	179.0	179.0	179.0	179.0	42.0	179.0	179.0	179.0	179.0	42.0	42.0
122.0	122.0	44.0	29.3	29.3	117.0	44.0	179.0	127.0	44.0	44.0	44.0	179.0	179.0
122.0	122.0	117.0	117.0	117.0	87.7	117.0	179.0	127.0	117.0	117.0	117.0	179.0	179.0
51.5	51.5	122.0	122.0	122.0	122.0	122.0	179.0	127.0	122.0	122.0	122.0	179.0	179.0
51.5	51.5	122.0	122.0	122.0	122.0	122.0	179.0	127.0	122.0	122.0	122.0	179.0	179.0
0.0	9.5	122.0	122.0	122.0	122.0	122.0	179.0	127.0	122.0	122.0	122.0	179.0	179.0
9.5	0.0	122.0	122.0	122.0	122.0	122.0	179.0	127.0	122.0	122.0	122.0	179.0	179.0
122.0	122.0	0.0	44.0	44.0	117.0	28.3	179.0	127.0	9.4	9.4	9.4	179.0	179.0
122.0	122.0	44.0	0.0	14.7	117.0	44.0	179.0	127.0	44.0	44.0	44.0	179.0	179.0
122.0	122.0	44.0	14.7	0.0	117.0	44.0	179.0	127.0	44.0	44.0	44.0	179.0	179.0
122.0	122.0	117.0	117.0	117.0	0.0	117.0	179.0	127.0	117.0	117.0	117.0	179.0	179.0
122.0	122.0	28.3	44.0	44.0	117.0	0.0	179.0	127.0	28.3	28.3	28.3	179.0	179.0
179.0	179.0	179.0	179.0	179.0	179.0	179.0	0.0	179.0	179.0	179.0	179.0	6.7	12.0
127.0	127.0	127.0	127.0	127.0	127.0	127.0	179.0	0.0	127.0	127.0	127.0	179.0	179.0
122.0	122.0	9.4	44.0	44.0	117.0	28.3	179.0	127.0	0.0	3.1	3.1	179.0	179.0
122.0	122.0	9.4	44.0	44.0	117.0	28.3	179.0	127.0	3.1	0.0	3.1	179.0	179.0
122.0	122.0	9.4	44.0	44.0	117.0	28.3	179.0	127.0	3.1	3.1	0.0	179.0	179.0
179.0	179.0	179.0	179.0	179.0	179.0	179.0	6.7	179.0	179.0	179.0	179.0	0.0	12.0
179.0	179.0	179.0	179.0	179.0	179.0	179.0	12.0	179.0	179.0	179.0	179.0	12.0	0.0
122.0	122.0	107.0	107.0	107.0	117.0	107.0	179.0	127.0	107.0	107.0	107.0	179.0	179.0
179.0	179.0	179.0	179.0	179.0	179.0	179.0	6.7	179.0	179.0	179.0	179.0	5.3	12.0
179.0	179.0	179.0	179.0	179.0	179.0	179.0	12.0	179.0	179.0	179.0	179.0	12.0	9.6
179.0	179.0	179.0	179.0	179.0	179.0	179.0	12.0	179.0	179.0	179.0	179.0	12.0	9.6
179.0	179.0	179.0	179.0	179.0	179.0	179.0	12.0	179.0	179.0	179.0	179.0	12.0	9.6
122.0	122.0	117.0	117.0	117.0	97.3	117.0	179.0	127.0	117.0	117.0	117.0	179.0	179.0
179.0	179.0	179.0	179.0	179.0	179.0	179.0	8.0	179.0	179.0	179.0	179.0	8.0	12.0
127.0	127.0	127.0	127.0	127.0	127.0	127.0	179.0	112.5	127.0	127.0	127.0	179.0	179.0
127.0	127.0	127.0	127.0	127.0	127.0	127.0	179.0	112.5	127.0	127.0	127.0	179.0	179.0
127.0	127.0	127.0	127.0	127.0	127.0	127.0	179.0	112.5	127.0	127.0	127.0	179.0	179.0
400.0	400.0	400.0	400.0	400.0	400.0	400.0	400.0	400.0	400.0	400.0	400.0	400.0	400.0
122.0	122.0	28.3	44.0	44.0	117.0	9.4	179.0	127.0	28.3	28.3	28.3	179.0	179.0
127.0	127.0	127.0	127.0	127.0	127.0	127.0	179.0	112.5	127.0	127.0	127.0	179.0	179.0
122.0	122.0	34.6	44.0	44.0	117.0	34.6	179.0	127.0	34.6	34.6	34.6	179.0	179.0
179.0	179.0	179.0	179.0	179.0	179.0	179.0	6.7	179.0	179.0	179.0	179.0	5.3	12.0
179.0	179.0	179.0	179.0	179.0	179.0	179.0	6.7	179.0	179.0	179.0	179.0	5.3	12.0
179.0	179.0	179.0	179.0	179.0	179.0	179.0	6.7	179.0	179.0	179.0	179.0	5.3	12.0
122.0	122.0	31.4	44.0	44.0	117.0	31.4	179.0	127.0	31.4	31.4	31.4	179.0	179.0
122.0	122.0	31.4	44.0	44.0	117.0	31.4	179.0	127.0	31.4	31.4	31.4	179.0	179.0
127.0	127.0	127.0	127.0	127.0	127.0	127.0	179.0	47.0	127.0	127.0	127.0	179.0	179.0
122.0	122.0	117.0	117.0	117.0	107.0	117.0	179.0	127.0	117.0	117.0	117.0	179.0	179.0
127.0	127.0	127.0	127.0	127.0	127.0	127.0	179.0	90.7	127.0	127.0	127.0	179.0	179.0
127.0	127.0	127.0	127.0	127.0	127.0	127.0	179.0	47.0	127.0	127.0	127.0	179.0	179.0
122.0	122.0	31.4	44.0	44.0	117.0	31.4	179.0	127.0	31.4	31.4	31.4	179.0	179.0
122.0	122.0	31.4	44.0	44.0	117.0	31.4	179.0	127.0	31.4	31.4	31.4	179.0	179.0

179.0	179.0	179.0	179.0	179.0	179.0	179.0	6.7	179.0	179.0	179.0	179.0	4.0	12.0
51.5	51.5	122.0	122.0	122.0	122.0	122.0	179.0	127.0	122.0	122.0	122.0	179.0	179.0
122.0	122.0	22.0	44.0	44.0	117.0	28.3	179.0	127.0	22.0	22.0	22.0	179.0	179.0
122.0	122.0	22.0	44.0	44.0	117.0	28.3	179.0	127.0	22.0	22.0	22.0	179.0	179.0
122.0	122.0	22.0	44.0	44.0	117.0	28.3	179.0	127.0	22.0	22.0	22.0	179.0	179.0
122.0	122.0	22.0	44.0	44.0	117.0	28.3	179.0	127.0	22.0	22.0	22.0	179.0	179.0
122.0	122.0	22.0	44.0	44.0	117.0	28.3	179.0	127.0	22.0	22.0	22.0	179.0	179.0
127.0	127.0	127.0	127.0	127.0	127.0	127.0	179.0	76.0	127.0	127.0	127.0	179.0	179.0
179.0	179.0	179.0	179.0	179.0	179.0	179.0	6.7	179.0	179.0	179.0	179.0	4.0	12.0
179.0	179.0	179.0	179.0	179.0	179.0	179.0	6.7	179.0	179.0	179.0	179.0	4.0	12.0
179.0	179.0	179.0	179.0	179.0	179.0	179.0	8.0	179.0	179.0	179.0	179.0	8.0	12.0
179.0	179.0	179.0	179.0	179.0	179.0	179.0	8.0	179.0	179.0	179.0	179.0	8.0	12.0
127.0	127.0	127.0	127.0	127.0	127.0	127.0	179.0	98.0	127.0	127.0	127.0	179.0	179.0
122.0	122.0	12.6	44.0	44.0	117.0	28.3	179.0	127.0	12.6	12.6	12.6	179.0	179.0
122.0	122.0	90.0	90.0	90.0	117.0	90.0	179.0	127.0	90.0	90.0	90.0	179.0	179.0
179.0	179.0	179.0	179.0	179.0	179.0	179.0	42.0	179.0	179.0	179.0	179.0	42.0	42.0
179.0	179.0	179.0	179.0	179.0	179.0	179.0	42.0	179.0	179.0	179.0	179.0	42.0	42.0
122.0	122.0	18.9	44.0	44.0	117.0	28.3	179.0	127.0	18.9	18.9	18.9	179.0	179.0
122.0	122.0	12.6	44.0	44.0	117.0	28.3	179.0	127.0	12.6	12.6	12.6	179.0	179.0
122.0	122.0	12.6	44.0	44.0	117.0	28.3	179.0	127.0	12.6	12.6	12.6	179.0	179.0
127.0	127.0	127.0	127.0	127.0	127.0	127.0	179.0	112.5	127.0	127.0	127.0	179.0	179.0
122.0	122.0	40.9	44.0	44.0	117.0	40.9	179.0	127.0	40.9	40.9	40.9	179.0	179.0
122.0	122.0	117.0	117.0	117.0	107.0	117.0	179.0	127.0	117.0	117.0	117.0	179.0	179.0
179.0	179.0	179.0	179.0	179.0	179.0	179.0	8.0	179.0	179.0	179.0	179.0	8.0	12.0
127.0	127.0	127.0	127.0	127.0	127.0	127.0	179.0	90.7	127.0	127.0	127.0	179.0	179.0
127.0	127.0	127.0	127.0	127.0	127.0	127.0	179.0	90.7	127.0	127.0	127.0	179.0	179.0
127.0	127.0	127.0	127.0	127.0	127.0	127.0	179.0	90.7	127.0	127.0	127.0	179.0	179.0
179.0	179.0	179.0	179.0	179.0	179.0	179.0	42.0	179.0	179.0	179.0	179.0	42.0	42.0
127.0	127.0	127.0	127.0	127.0	127.0	127.0	179.0	15.7	127.0	127.0	127.0	179.0	179.0
122.0	122.0	40.9	44.0	44.0	117.0	40.9	179.0	127.0	40.9	40.9	40.9	179.0	179.0
127.0	127.0	127.0	127.0	127.0	127.0	127.0	179.0	90.7	127.0	127.0	127.0	179.0	179.0
179.0	179.0	179.0	179.0	179.0	179.0	179.0	10.7	179.0	179.0	179.0	179.0	10.7	12.0
51.5	51.5	122.0	122.0	122.0	122.0	122.0	179.0	127.0	122.0	122.0	122.0	179.0	179.0
450.0	450.0	450.0	450.0	450.0	450.0	450.0	450.0	450.0	450.0	450.0	450.0	450.0	450.0
500.0	500.0	500.0	500.0	500.0	500.0	500.0	500.0	500.0	500.0	500.0	500.0	500.0	500.0
122.0	122.0	117.0	117.0	117.0	97.3	117.0	179.0	127.0	117.0	117.0	117.0	179.0	179.0
127.0	127.0	127.0	127.0	127.0	127.0	127.0	179.0	112.5	127.0	127.0	127.0	179.0	179.0
127.0	127.0	127.0	127.0	127.0	127.0	127.0	179.0	90.7	127.0	127.0	127.0	179.0	179.0
147.0	147.0	147.0	147.0	147.0	147.0	147.0	179.0	147.0	147.0	147.0	147.0	179.0	179.0
147.0	147.0	147.0	147.0	147.0	147.0	147.0	179.0	147.0	147.0	147.0	147.0	179.0	179.0
51.5	51.5	122.0	122.0	122.0	122.0	122.0	179.0	127.0	122.0	122.0	122.0	179.0	179.0
179.0	179.0	179.0	179.0	179.0	179.0	179.0	3.3	179.0	179.0	179.0	179.0	6.7	12.0
179.0	179.0	179.0	179.0	179.0	179.0	179.0	12.0	179.0	179.0	179.0	179.0	12.0	4.8
122.0	122.0	12.6	44.0	44.0	117.0	28.3	179.0	127.0	12.6	12.6	12.6	179.0	179.0
325.0	325.0	325.0	325.0	325.0	325.0	325.0	325.0	325.0	325.0	325.0	325.0	325.0	325.0
122.0	122.0	117.0	117.0	117.0	107.0	117.0	179.0	127.0	117.0	117.0	117.0	179.0	179.0
122.0	122.0	117.0	117.0	117.0	107.0	117.0	179.0	127.0	117.0	117.0	117.0	179.0	179.0
122.0	122.0	117.0	117.0	117.0	107.0	117.0	179.0	127.0	117.0	117.0	117.0	179.0	179.0
179.0	179.0	179.0	179.0	179.0	179.0	179.0	6.7	179.0	179.0	179.0	179.0	5.3	12.0
179.0	179.0	179.0	179.0	179.0	179.0	179.0	6.7	179.0	179.0	179.0	179.0	5.3	12.0
179.0	179.0	179.0	179.0	179.0	179.0	179.0	6.7	179.0	179.0	179.0	179.0	5.3	12.0
179.0	179.0	179.0	179.0	179.0	179.0	179.0	6.7	179.0	179.0	179.0	179.0	5.3	12.0
84.0	84.0	122.0	122.0	122.0	122.0	122.0	179.0	127.0	122.0	122.0	122.0	179.0	179.0
84.0	84.0	122.0	122.0	122.0	122.0	122.0	179.0	127.0	122.0	122.0	122.0	179.0	179.0
127.0	127.0	127.0	127.0	127.0	127.0	127.0	179.0	98.0	127.0	127.0	127.0	179.0	179.0
127.0	127.0	127.0	127.0	127.0	127.0	127.0	179.0	98.0	127.0	127.0	127.0	179.0	179.0
127.0	127.0	127.0	127.0	127.0	127.0	127.0	179.0	47.0	127.0	127.0	127.0	179.0	179.0
122.0	122.0	117.0	117.0	117.0	107.0	117.0	179.0	127.0	117.0	117.0	117.0	179.0	179.0
127.0	127.0	127.0	127.0	127.0	127.0	127.0	179.0	31.3	127.0	127.0	127.0	179.0	179.0
127.0	127.0	127.0	127.0	127.0	127.0	127.0	179.0	90.7	127.0	127.0	127.0	179.0	179.0
127.0	127.0	127.0	127.0	127.0	127.0	127.0	179.0	47.0	127.0	127.0	127.0	179.0	179.0
127.0	127.0	127.0	127.0	127.0	127.0	127.0	179.0	47.0	127.0	127.0	127.0	179.0	179.0
84.0	84.0	122.0	122.0	122.0	122.0	122.0	179.0	127.0	122.0	122.0	122.0	179.0	179.0
84.0	84.0	122.0	122.0	122.0	122.0	122.0	179.0	127.0	122.0	122.0	122.0	179.0	179.0
84.0	84.0	122.0	122.0	122.0	122.0	122.0	179.0	127.0	122.0	122.0	122.0	179.0	179.0
84.0	84.0	122.0	122.0	122.0	122.0	122.0	179.0	127.0	122.0	122.0	122.0	179.0	179.0
51.5	51.5	122.0	122.0	122.0	122.0	122.0	179.0	127.0	122.0	122.0	122.0	179.0	179.0
127.0	127.0	127.0	127.0	127.0	127.0	127.0	179.0	98.0	127.0	127.0	127.0	179.0	179.0
127.0	127.0	127.0	127.0	127.0	127.0	127.0	179.0	98.0	127.0	127.0	127.0	179.0	179.0
127.0	127.0	127.0	127.0	127.0	127.0	127.0	179.0	98.0	127.0	127.0	127.0	179.0	179.0
19.0	19.0	122.0	122.0	122.0	122.0	122.0	179.0	127.0	122.0	122.0	122.0	179.0	179.0
51.5	51.5	122.0	122.0	122.0	122.0	122.0	179.0	127.0	122.0	122.0	122.0	179.0	179.0
51.5	51.5	122.0	122.0	122.0	122.0	122.0	179.0	127.0	122.0	122.0	122.0	179.0	179.0
122.0	122.0	37.7	44.0	44.0	117.0	37.7	179.0	127.0	37.7	37.7	37.7	179.0	179.0

122.0	122.0	37.7	44.0	44.0	117.0	37.7	179.0	127.0	37.7	37.7	37.7	179.0	179.0
122.0	122.0	37.7	44.0	44.0	117.0	37.7	179.0	127.0	37.7	37.7	37.7	179.0	179.0
179.0	179.0	179.0	179.0	179.0	179.0	179.0	12.0	179.0	179.0	179.0	179.0	12.0	9.6
179.0	179.0	179.0	179.0	179.0	179.0	179.0	12.0	179.0	179.0	179.0	179.0	12.0	9.6
51.5	51.5	122.0	122.0	122.0	122.0	122.0	179.0	127.0	122.0	122.0	122.0	179.0	179.0
127.0	127.0	127.0	127.0	127.0	127.0	127.0	179.0	112.5	127.0	127.0	127.0	179.0	179.0
127.0	127.0	127.0	127.0	127.0	127.0	127.0	179.0	112.5	127.0	127.0	127.0	179.0	179.0
127.0	127.0	127.0	127.0	127.0	127.0	127.0	179.0	112.5	127.0	127.0	127.0	179.0	179.0
122.0	122.0	117.0	117.0	117.0	78.0	117.0	179.0	127.0	117.0	117.0	117.0	179.0	179.0
122.0	122.0	28.3	44.0	44.0	117.0	18.9	179.0	127.0	28.3	28.3	28.3	179.0	179.0
122.0	122.0	15.7	44.0	44.0	117.0	28.3	179.0	127.0	15.7	15.7	15.7	179.0	179.0
122.0	122.0	15.7	44.0	44.0	117.0	28.3	179.0	127.0	15.7	15.7	15.7	179.0	179.0
51.5	51.5	122.0	122.0	122.0	122.0	122.0	179.0	127.0	122.0	122.0	122.0	179.0	179.0
51.5	51.5	122.0	122.0	122.0	122.0	122.0	179.0	127.0	122.0	122.0	122.0	179.0	179.0
122.0	122.0	40.9	44.0	44.0	117.0	40.9	179.0	127.0	40.9	40.9	40.9	179.0	179.0
122.0	122.0	9.4	44.0	44.0	117.0	28.3	179.0	127.0	6.3	6.3	6.3	179.0	179.0
122.0	122.0	107.0	107.0	107.0	117.0	107.0	179.0	127.0	107.0	107.0	107.0	179.0	179.0
122.0	122.0	25.1	44.0	44.0	117.0	28.3	179.0	127.0	25.1	25.1	25.1	179.0	179.0
127.0	127.0	127.0	127.0	127.0	127.0	127.0	179.0	90.7	127.0	127.0	127.0	179.0	179.0
127.0	127.0	127.0	127.0	127.0	127.0	127.0	179.0	90.7	127.0	127.0	127.0	179.0	179.0
127.0	127.0	127.0	127.0	127.0	127.0	127.0	179.0	90.7	127.0	127.0	127.0	179.0	179.0
127.0	127.0	127.0	127.0	127.0	127.0	127.0	179.0	90.7	127.0	127.0	127.0	179.0	179.0
127.0	127.0	127.0	127.0	127.0	127.0	127.0	179.0	90.7	127.0	127.0	127.0	179.0	179.0
122.0	122.0	40.9	44.0	44.0	117.0	40.9	179.0	127.0	40.9	40.9	40.9	179.0	179.0
122.0	122.0	37.7	44.0	44.0	117.0	37.7	179.0	127.0	37.7	37.7	37.7	179.0	179.0
179.0	179.0	179.0	179.0	179.0	179.0	179.0	72.0	179.0	179.0	179.0	179.0	72.0	72.0
127.0	127.0	127.0	127.0	127.0	127.0	127.0	179.0	76.0	127.0	127.0	127.0	179.0	179.0
122.0	122.0	117.0	117.0	117.0	107.0	117.0	179.0	127.0	117.0	117.0	117.0	179.0	179.0
122.0	122.0	117.0	117.0	117.0	107.0	117.0	179.0	127.0	117.0	117.0	117.0	179.0	179.0
122.0	122.0	117.0	117.0	117.0	107.0	117.0	179.0	127.0	117.0	117.0	117.0	179.0	179.0
127.0	127.0	127.0	127.0	127.0	127.0	127.0	179.0	90.7	127.0	127.0	127.0	179.0	179.0
127.0	127.0	127.0	127.0	127.0	127.0	127.0	179.0	90.7	127.0	127.0	127.0	179.0	179.0
127.0	127.0	127.0	127.0	127.0	127.0	127.0	179.0	90.7	127.0	127.0	127.0	179.0	179.0
127.0	127.0	127.0	127.0	127.0	127.0	127.0	179.0	90.7	127.0	127.0	127.0	179.0	179.0
127.0	127.0	127.0	127.0	127.0	127.0	127.0	179.0	98.0	127.0	127.0	127.0	179.0	179.0
127.0	127.0	127.0	127.0	127.0	127.0	127.0	179.0	98.0	127.0	127.0	127.0	179.0	179.0
127.0	127.0	127.0	127.0	127.0	127.0	127.0	179.0	98.0	127.0	127.0	127.0	179.0	179.0

Daucus_ca	Deschamps	Digitaria_is	Digitaria_sa	Echinochlo	Echium_vu	Elymus_rep	Epilobium_	Epilobium_	Epilobium_	Equisetum_	Erigeron_a	Erodium_ci	Eupatorium
107.0	179.0	179.0	179.0	179.0	117.0	179.0	127.0	127.0	127.0	400.0	40.9	127.0	40.9
179.0	6.7	12.0	12.0	12.0	179.0	8.0	179.0	179.0	179.0	400.0	179.0	179.0	179.0
179.0	6.7	12.0	12.0	12.0	179.0	8.0	179.0	179.0	179.0	400.0	179.0	179.0	179.0
179.0	6.7	12.0	12.0	12.0	179.0	8.0	179.0	179.0	179.0	400.0	179.0	179.0	179.0
117.0	179.0	179.0	179.0	179.0	107.0	179.0	127.0	127.0	127.0	400.0	117.0	127.0	117.0
179.0	5.3	12.0	12.0	12.0	179.0	8.0	179.0	179.0	179.0	400.0	179.0	179.0	179.0
107.0	179.0	179.0	179.0	179.0	117.0	179.0	127.0	127.0	127.0	400.0	44.0	127.0	44.0
122.0	179.0	179.0	179.0	179.0	122.0	179.0	127.0	127.0	127.0	400.0	122.0	127.0	122.0
107.0	179.0	179.0	179.0	179.0	117.0	179.0	127.0	127.0	127.0	400.0	40.9	127.0	40.9
107.0	179.0	179.0	179.0	179.0	117.0	179.0	127.0	127.0	127.0	400.0	40.9	127.0	40.9
127.0	179.0	179.0	179.0	179.0	127.0	179.0	95.0	95.0	95.0	400.0	127.0	95.0	127.0
127.0	179.0	179.0	179.0	179.0	127.0	179.0	112.5	112.5	112.5	400.0	127.0	112.5	127.0
179.0	9.3	12.0	12.0	12.0	179.0	9.3	179.0	179.0	179.0	400.0	179.0	179.0	179.0
179.0	8.0	12.0	12.0	12.0	179.0	4.8	179.0	179.0	179.0	400.0	179.0	179.0	179.0
179.0	8.0	12.0	12.0	12.0	179.0	4.8	179.0	179.0	179.0	400.0	179.0	179.0	179.0
179.0	6.7	12.0	12.0	12.0	179.0	8.0	179.0	179.0	179.0	400.0	179.0	179.0	179.0
179.0	42.0	42.0	42.0	42.0	179.0	42.0	179.0	179.0	179.0	400.0	179.0	179.0	179.0
179.0	42.0	42.0	42.0	42.0	179.0	42.0	179.0	179.0	179.0	400.0	179.0	179.0	179.0
179.0	42.0	42.0	42.0	42.0	179.0	42.0	179.0	179.0	179.0	400.0	179.0	179.0	179.0
179.0	42.0	42.0	42.0	42.0	179.0	42.0	179.0	179.0	179.0	400.0	179.0	179.0	179.0
107.0	179.0	179.0	179.0	179.0	117.0	179.0	127.0	127.0	127.0	400.0	44.0	127.0	44.0
117.0	179.0	179.0	179.0	179.0	97.3	179.0	127.0	127.0	127.0	400.0	117.0	127.0	117.0
122.0	179.0	179.0	179.0	179.0	122.0	179.0	127.0	127.0	127.0	400.0	122.0	127.0	122.0
122.0	179.0	179.0	179.0	179.0	122.0	179.0	127.0	127.0	127.0	400.0	122.0	127.0	122.0
122.0	179.0	179.0	179.0	179.0	122.0	179.0	127.0	127.0	127.0	400.0	122.0	127.0	122.0
122.0	179.0	179.0	179.0	179.0	122.0	179.0	127.0	127.0	127.0	400.0	122.0	127.0	122.0
107.0	179.0	179.0	179.0	179.0	117.0	179.0	127.0	127.0	127.0	400.0	28.3	127.0	34.6
107.0	179.0	179.0	179.0	179.0	117.0	179.0	127.0	127.0	127.0	400.0	44.0	127.0	44.0
107.0	179.0	179.0	179.0	179.0	117.0	179.0	127.0	127.0	127.0	400.0	44.0	127.0	44.0
117.0	179.0	179.0	179.0	179.0	97.3	179.0	127.0	127.0	127.0	400.0	117.0	127.0	117.0
107.0	179.0	179.0	179.0	179.0	117.0	179.0	127.0	127.0	127.0	400.0	9.4	127.0	34.6
179.0	6.7	12.0	12.0	12.0	179.0	8.0	179.0	179.0	179.0	400.0	179.0	179.0	179.0
127.0	179.0	179.0	179.0	179.0	127.0	179.0	112.5	112.5	112.5	400.0	127.0	112.5	127.0
107.0	179.0	179.0	179.0	179.0	117.0	179.0	127.0	127.0	127.0	400.0	28.3	127.0	34.6
107.0	179.0	179.0	179.0	179.0	117.0	179.0	127.0	127.0	127.0	400.0	28.3	127.0	34.6
107.0	179.0	179.0	179.0	179.0	117.0	179.0	127.0	127.0	127.0	400.0	28.3	127.0	34.6
179.0	5.3	12.0	12.0	12.0	179.0	8.0	179.0	179.0	179.0	400.0	179.0	179.0	179.0
179.0	12.0	9.6	9.6	9.6	179.0	12.0	179.0	179.0	179.0	400.0	179.0	179.0	179.0
0.0	179.0	179.0	179.0	179.0	117.0	179.0	127.0	127.0	127.0	400.0	107.0	127.0	107.0
179.0	0.0	12.0	12.0	12.0	179.0	8.0	179.0	179.0	179.0	400.0	179.0	179.0	179.0
179.0	12.0	0.0	2.4	4.8	179.0	12.0	179.0	179.0	179.0	400.0	179.0	179.0	179.0
179.0	12.0	2.4	0.0	4.8	179.0	12.0	179.0	179.0	179.0	400.0	179.0	179.0	179.0
179.0	12.0	4.8	4.8	0.0	179.0	12.0	179.0	179.0	179.0	400.0	179.0	179.0	179.0
117.0	179.0	179.0	179.0	179.0	0.0	179.0	127.0	127.0	127.0	400.0	117.0	127.0	117.0
179.0	8.0	12.0	12.0	12.0	179.0	0.0	179.0	179.0	179.0	400.0	179.0	179.0	179.0
127.0	179.0	179.0	179.0	179.0	127.0	179.0	0.0	10.0	10.0	400.0	127.0	57.5	127.0
127.0	179.0	179.0	179.0	179.0	127.0	179.0	10.0	0.0	10.0	400.0	127.0	57.5	127.0
127.0	179.0	179.0	179.0	179.0	127.0	179.0	10.0	10.0	0.0	400.0	127.0	57.5	127.0
400.0	400.0	400.0	400.0	400.0	400.0	400.0	400.0	400.0	400.0	0.0	400.0	400.0	400.0
107.0	179.0	179.0	179.0	179.0	117.0	179.0	127.0	127.0	127.0	400.0	0.0	127.0	34.6
127.0	179.0	179.0	179.0	179.0	127.0	179.0	57.5	57.5	57.5	400.0	127.0	0.0	127.0
107.0	179.0	179.0	179.0	179.0	117.0	179.0	127.0	127.0	127.0	400.0	34.6	127.0	0.0
179.0	3.6	12.0	12.0	12.0	179.0	8.0	179.0	179.0	179.0	400.0	179.0	179.0	179.0
179.0	3.6	12.0	12.0	12.0	179.0	8.0	179.0	179.0	179.0	400.0	179.0	179.0	179.0
179.0	3.6	12.0	12.0	12.0	179.0	8.0	179.0	179.0	179.0	400.0	179.0	179.0	179.0
107.0	179.0	179.0	179.0	179.0	117.0	179.0	127.0	127.0	127.0	400.0	31.4	127.0	34.6
107.0	179.0	179.0	179.0	179.0	117.0	179.0	127.0	127.0	127.0	400.0	31.4	127.0	34.6
127.0	179.0	179.0	179.0	179.0	127.0	179.0	112.5	112.5	112.5	400.0	127.0	112.5	127.0
117.0	179.0	179.0	179.0	179.0	107.0	179.0	127.0	127.0	127.0	400.0	117.0	127.0	117.0
127.0	179.0	179.0	179.0	179.0	127.0	179.0	112.5	112.5	112.5	400.0	127.0	112.5	127.0
127.0	179.0	179.0	179.0	179.0	127.0	179.0	112.5	112.5	112.5	400.0	127.0	112.5	127.0
107.0	179.0	179.0	179.0	179.0	117.0	179.0	127.0	127.0	127.0	400.0	31.4	127.0	34.6
107.0	179.0	179.0	179.0	179.0	117.0	179.0	127.0	127.0	127.0	400.0	31.4	127.0	34.6

179.0	5.3	12.0	12.0	12.0	179.0	8.0	179.0	179.0	179.0	400.0	179.0	179.0	179.0
122.0	179.0	179.0	179.0	179.0	122.0	179.0	127.0	127.0	127.0	400.0	122.0	127.0	122.0
107.0	179.0	179.0	179.0	179.0	117.0	179.0	127.0	127.0	127.0	400.0	28.3	127.0	34.6
107.0	179.0	179.0	179.0	179.0	117.0	179.0	127.0	127.0	127.0	400.0	28.3	127.0	34.6
107.0	179.0	179.0	179.0	179.0	117.0	179.0	127.0	127.0	127.0	400.0	28.3	127.0	34.6
107.0	179.0	179.0	179.0	179.0	117.0	179.0	127.0	127.0	127.0	400.0	28.3	127.0	34.6
107.0	179.0	179.0	179.0	179.0	117.0	179.0	127.0	127.0	127.0	400.0	28.3	127.0	34.6
127.0	179.0	179.0	179.0	179.0	127.0	179.0	112.5	112.5	112.5	400.0	127.0	112.5	127.0
179.0	5.3	12.0	12.0	12.0	179.0	8.0	179.0	179.0	179.0	400.0	179.0	179.0	179.0
179.0	5.3	12.0	12.0	12.0	179.0	8.0	179.0	179.0	179.0	400.0	179.0	179.0	179.0
179.0	8.0	12.0	12.0	12.0	179.0	3.2	179.0	179.0	179.0	400.0	179.0	179.0	179.0
179.0	8.0	12.0	12.0	12.0	179.0	3.2	179.0	179.0	179.0	400.0	179.0	179.0	179.0
127.0	179.0	179.0	179.0	179.0	127.0	179.0	112.5	112.5	112.5	400.0	127.0	112.5	127.0
107.0	179.0	179.0	179.0	179.0	117.0	179.0	127.0	127.0	127.0	400.0	28.3	127.0	34.6
107.0	179.0	179.0	179.0	179.0	117.0	179.0	127.0	127.0	127.0	400.0	90.0	127.0	90.0
179.0	42.0	42.0	42.0	42.0	179.0	42.0	179.0	179.0	179.0	400.0	179.0	179.0	179.0
179.0	42.0	42.0	42.0	42.0	179.0	42.0	179.0	179.0	179.0	400.0	179.0	179.0	179.0
107.0	179.0	179.0	179.0	179.0	117.0	179.0	127.0	127.0	127.0	400.0	28.3	127.0	34.6
107.0	179.0	179.0	179.0	179.0	117.0	179.0	127.0	127.0	127.0	400.0	28.3	127.0	34.6
107.0	179.0	179.0	179.0	179.0	117.0	179.0	127.0	127.0	127.0	400.0	28.3	127.0	34.6
127.0	179.0	179.0	179.0	179.0	127.0	179.0	95.0	95.0	95.0	400.0	127.0	95.0	127.0
107.0	179.0	179.0	179.0	179.0	117.0	179.0	127.0	127.0	127.0	400.0	40.9	127.0	40.9
117.0	179.0	179.0	179.0	179.0	107.0	179.0	127.0	127.0	127.0	400.0	117.0	127.0	117.0
179.0	8.0	12.0	12.0	12.0	179.0	6.4	179.0	179.0	179.0	400.0	179.0	179.0	179.0
127.0	179.0	179.0	179.0	179.0	127.0	179.0	112.5	112.5	112.5	400.0	127.0	112.5	127.0
127.0	179.0	179.0	179.0	179.0	127.0	179.0	112.5	112.5	112.5	400.0	127.0	112.5	127.0
127.0	179.0	179.0	179.0	179.0	127.0	179.0	112.5	112.5	112.5	400.0	127.0	112.5	127.0
179.0	42.0	42.0	42.0	42.0	179.0	42.0	179.0	179.0	179.0	400.0	179.0	179.0	179.0
127.0	179.0	179.0	179.0	179.0	127.0	179.0	112.5	112.5	112.5	400.0	127.0	112.5	127.0
107.0	179.0	179.0	179.0	179.0	117.0	179.0	127.0	127.0	127.0	400.0	40.9	127.0	40.9
127.0	179.0	179.0	179.0	179.0	127.0	179.0	112.5	112.5	112.5	400.0	127.0	112.5	127.0
179.0	10.7	12.0	12.0	12.0	179.0	10.7	179.0	179.0	179.0	400.0	179.0	179.0	179.0
122.0	179.0	179.0	179.0	179.0	122.0	179.0	127.0	127.0	127.0	400.0	122.0	127.0	122.0
450.0	450.0	450.0	450.0	450.0	450.0	450.0	450.0	450.0	450.0	450.0	450.0	450.0	450.0
500.0	500.0	500.0	500.0	500.0	500.0	500.0	500.0	500.0	500.0	500.0	500.0	500.0	500.0
117.0	179.0	179.0	179.0	179.0	48.7	179.0	127.0	127.0	127.0	400.0	117.0	127.0	117.0
127.0	179.0	179.0	179.0	179.0	127.0	179.0	20.0	20.0	20.0	400.0	127.0	57.5	127.0
127.0	179.0	179.0	179.0	179.0	127.0	179.0	112.5	112.5	112.5	400.0	127.0	112.5	127.0
147.0	179.0	179.0	179.0	179.0	147.0	179.0	147.0	147.0	147.0	400.0	147.0	147.0	147.0
147.0	179.0	179.0	179.0	179.0	147.0	179.0	147.0	147.0	147.0	400.0	147.0	147.0	147.0
122.0	179.0	179.0	179.0	179.0	122.0	179.0	127.0	127.0	127.0	400.0	122.0	127.0	122.0
179.0	6.7	12.0	12.0	12.0	179.0	8.0	179.0	179.0	179.0	400.0	179.0	179.0	179.0
179.0	12.0	9.6	9.6	9.6	179.0	12.0	179.0	179.0	179.0	400.0	179.0	179.0	179.0
107.0	179.0	179.0	179.0	179.0	117.0	179.0	127.0	127.0	127.0	400.0	28.3	127.0	34.6
325.0	325.0	325.0	325.0	325.0	325.0	325.0	325.0	325.0	325.0	400.0	325.0	325.0	325.0
117.0	179.0	179.0	179.0	179.0	107.0	179.0	127.0	127.0	127.0	400.0	117.0	127.0	117.0
117.0	179.0	179.0	179.0	179.0	107.0	179.0	127.0	127.0	127.0	400.0	117.0	127.0	117.0
117.0	179.0	179.0	179.0	179.0	107.0	179.0	127.0	127.0	127.0	400.0	117.0	127.0	117.0
179.0	5.3	12.0	12.0	12.0	179.0	8.0	179.0	179.0	179.0	400.0	179.0	179.0	179.0
179.0	5.3	12.0	12.0	12.0	179.0	8.0	179.0	179.0	179.0	400.0	179.0	179.0	179.0
179.0	5.3	12.0	12.0	12.0	179.0	8.0	179.0	179.0	179.0	400.0	179.0	179.0	179.0
179.0	5.3	12.0	12.0	12.0	179.0	8.0	179.0	179.0	179.0	400.0	179.0	179.0	179.0
122.0	179.0	179.0	179.0	179.0	122.0	179.0	127.0	127.0	127.0	400.0	122.0	127.0	122.0
122.0	179.0	179.0	179.0	179.0	122.0	179.0	127.0	127.0	127.0	400.0	122.0	127.0	122.0
127.0	179.0	179.0	179.0	179.0	127.0	179.0	112.5	112.5	112.5	400.0	127.0	112.5	127.0
127.0	179.0	179.0	179.0	179.0	127.0	179.0	112.5	112.5	112.5	400.0	127.0	112.5	127.0
127.0	179.0	179.0	179.0	179.0	127.0	179.0	112.5	112.5	112.5	400.0	127.0	112.5	127.0
117.0	179.0	179.0	179.0	179.0	107.0	179.0	127.0	127.0	127.0	400.0	117.0	127.0	117.0
127.0	179.0	179.0	179.0	179.0	127.0	179.0	112.5	112.5	112.5	400.0	127.0	112.5	127.0
127.0	179.0	179.0	179.0	179.0	127.0	179.0	112.5	112.5	112.5	400.0	127.0	112.5	127.0
127.0	179.0	179.0	179.0	179.0	127.0	179.0	112.5	112.5	112.5	400.0	127.0	112.5	127.0
127.0	179.0	179.0	179.0	179.0	127.0	179.0	112.5	112.5	112.5	400.0	127.0	112.5	127.0
122.0	179.0	179.0	179.0	179.0	122.0	179.0	127.0	127.0	127.0	400.0	122.0	127.0	122.0
122.0	179.0	179.0	179.0	179.0	122.0	179.0	127.0	127.0	127.0	400.0	122.0	127.0	122.0
122.0	179.0	179.0	179.0	179.0	122.0	179.0	127.0	127.0	127.0	400.0	122.0	127.0	122.0
122.0	179.0	179.0	179.0	179.0	122.0	179.0	127.0	127.0	127.0	400.0	122.0	127.0	122.0
122.0	179.0	179.0	179.0	179.0	122.0	179.0	127.0	127.0	127.0	400.0	122.0	127.0	122.0
127.0	179.0	179.0	179.0	179.0	127.0	179.0	112.5	112.5	112.5	400.0	127.0	112.5	127.0
127.0	179.0	179.0	179.0	179.0	127.0	179.0	112.5	112.5	112.5	400.0	127.0	112.5	127.0
127.0	179.0	179.0	179.0	179.0	127.0	179.0	112.5	112.5	112.5	400.0	127.0	112.5	127.0
122.0	179.0	179.0	179.0	179.0	122.0	179.0	127.0	127.0	127.0	400.0	122.0	127.0	122.0
122.0	179.0	179.0	179.0	179.0	122.0	179.0	127.0	127.0	127.0	400.0	122.0	127.0	122.0
122.0	179.0	179.0	179.0	179.0	122.0	179.0	127.0	127.0	127.0	400.0	122.0	127.0	122.0
107.0	179.0	179.0	179.0	179.0	117.0	179.0	127.0	127.0	127.0	400.0	37.7	127.0	37.7

107.0	179.0	179.0	179.0	179.0	117.0	179.0	127.0	127.0	127.0	400.0	37.7	127.0	37.7
107.0	179.0	179.0	179.0	179.0	117.0	179.0	127.0	127.0	127.0	400.0	37.7	127.0	37.7
179.0	12.0	7.2	7.2	7.2	179.0	12.0	179.0	179.0	179.0	400.0	179.0	179.0	179.0
179.0	12.0	7.2	7.2	7.2	179.0	12.0	179.0	179.0	179.0	400.0	179.0	179.0	179.0
122.0	179.0	179.0	179.0	179.0	122.0	179.0	127.0	127.0	127.0	400.0	122.0	127.0	122.0
127.0	179.0	179.0	179.0	179.0	127.0	179.0	95.0	95.0	95.0	400.0	127.0	95.0	127.0
127.0	179.0	179.0	179.0	179.0	127.0	179.0	95.0	95.0	95.0	400.0	127.0	95.0	127.0
127.0	179.0	179.0	179.0	179.0	127.0	179.0	95.0	95.0	95.0	400.0	127.0	95.0	127.0
117.0	179.0	179.0	179.0	179.0	97.3	179.0	127.0	127.0	127.0	400.0	117.0	127.0	117.0
107.0	179.0	179.0	179.0	179.0	117.0	179.0	127.0	127.0	127.0	400.0	18.9	127.0	34.6
107.0	179.0	179.0	179.0	179.0	117.0	179.0	127.0	127.0	127.0	400.0	28.3	127.0	34.6
107.0	179.0	179.0	179.0	179.0	117.0	179.0	127.0	127.0	127.0	400.0	28.3	127.0	34.6
122.0	179.0	179.0	179.0	179.0	122.0	179.0	127.0	127.0	127.0	400.0	122.0	127.0	122.0
122.0	179.0	179.0	179.0	179.0	122.0	179.0	127.0	127.0	127.0	400.0	122.0	127.0	122.0
107.0	179.0	179.0	179.0	179.0	117.0	179.0	127.0	127.0	127.0	400.0	40.9	127.0	40.9
107.0	179.0	179.0	179.0	179.0	117.0	179.0	127.0	127.0	127.0	400.0	28.3	127.0	34.6
33.0	179.0	179.0	179.0	179.0	117.0	179.0	127.0	127.0	127.0	400.0	107.0	127.0	107.0
107.0	179.0	179.0	179.0	179.0	117.0	179.0	127.0	127.0	127.0	400.0	28.3	127.0	34.6
127.0	179.0	179.0	179.0	179.0	127.0	179.0	112.5	112.5	112.5	400.0	127.0	112.5	127.0
127.0	179.0	179.0	179.0	179.0	127.0	179.0	112.5	112.5	112.5	400.0	127.0	112.5	127.0
127.0	179.0	179.0	179.0	179.0	127.0	179.0	112.5	112.5	112.5	400.0	127.0	112.5	127.0
127.0	179.0	179.0	179.0	179.0	127.0	179.0	112.5	112.5	112.5	400.0	127.0	112.5	127.0
127.0	179.0	179.0	179.0	179.0	127.0	179.0	112.5	112.5	112.5	400.0	127.0	112.5	127.0
107.0	179.0	179.0	179.0	179.0	117.0	179.0	127.0	127.0	127.0	400.0	40.9	127.0	40.9
107.0	179.0	179.0	179.0	179.0	117.0	179.0	127.0	127.0	127.0	400.0	37.7	127.0	37.7
179.0	72.0	72.0	72.0	72.0	179.0	72.0	179.0	179.0	179.0	400.0	179.0	179.0	179.0
127.0	179.0	179.0	179.0	179.0	127.0	179.0	112.5	112.5	112.5	400.0	127.0	112.5	127.0
117.0	179.0	179.0	179.0	179.0	107.0	179.0	127.0	127.0	127.0	400.0	117.0	127.0	117.0
117.0	179.0	179.0	179.0	179.0	107.0	179.0	127.0	127.0	127.0	400.0	117.0	127.0	117.0
117.0	179.0	179.0	179.0	179.0	107.0	179.0	127.0	127.0	127.0	400.0	117.0	127.0	117.0
127.0	179.0	179.0	179.0	179.0	127.0	179.0	112.5	112.5	112.5	400.0	127.0	112.5	127.0
127.0	179.0	179.0	179.0	179.0	127.0	179.0	112.5	112.5	112.5	400.0	127.0	112.5	127.0
127.0	179.0	179.0	179.0	179.0	127.0	179.0	112.5	112.5	112.5	400.0	127.0	112.5	127.0
127.0	179.0	179.0	179.0	179.0	127.0	179.0	112.5	112.5	112.5	400.0	127.0	112.5	127.0
127.0	179.0	179.0	179.0	179.0	127.0	179.0	112.5	112.5	112.5	400.0	127.0	112.5	127.0
127.0	179.0	179.0	179.0	179.0	127.0	179.0	112.5	112.5	112.5	400.0	127.0	112.5	127.0
127.0	179.0	179.0	179.0	179.0	127.0	179.0	112.5	112.5	112.5	400.0	127.0	112.5	127.0

Festuca_gi	Festuca_ov	Festuca_ru	Filago_arve	Filago_min	Fragaria_ve	Galeopsis_	Genista_pil	Geum_urb	Gnaphaliur	Helichrysur	Helictotrich	Herniaria_	Hieracium_
179.0	179.0	179.0	40.9	40.9	127.0	117.0	127.0	127.0	40.9	40.9	179.0	122.0	40.9
6.7	6.7	6.7	179.0	179.0	179.0	179.0	179.0	179.0	179.0	179.0	6.7	179.0	179.0
6.7	6.7	6.7	179.0	179.0	179.0	179.0	179.0	179.0	179.0	179.0	6.7	179.0	179.0
6.7	6.7	6.7	179.0	179.0	179.0	179.0	179.0	179.0	179.0	179.0	6.7	179.0	179.0
179.0	179.0	179.0	117.0	117.0	127.0	23.0	127.0	127.0	117.0	117.0	179.0	122.0	117.0
5.3	5.3	5.3	179.0	179.0	179.0	179.0	179.0	179.0	179.0	179.0	5.3	179.0	179.0
179.0	179.0	179.0	44.0	44.0	127.0	117.0	127.0	127.0	44.0	44.0	179.0	122.0	44.0
179.0	179.0	179.0	122.0	122.0	127.0	122.0	127.0	127.0	122.0	122.0	179.0	45.1	122.0
179.0	179.0	179.0	40.9	40.9	127.0	117.0	127.0	127.0	40.9	40.9	179.0	122.0	40.9
179.0	179.0	179.0	40.9	40.9	127.0	117.0	127.0	127.0	40.9	40.9	179.0	122.0	40.9
179.0	179.0	179.0	127.0	127.0	112.5	127.0	112.5	112.5	127.0	127.0	179.0	127.0	127.0
179.0	179.0	179.0	127.0	127.0	83.3	127.0	90.7	83.3	127.0	127.0	179.0	127.0	127.0
9.3	9.3	9.3	179.0	179.0	179.0	179.0	179.0	179.0	179.0	179.0	9.3	179.0	179.0
8.0	8.0	8.0	179.0	179.0	179.0	179.0	179.0	179.0	179.0	179.0	8.0	179.0	179.0
8.0	8.0	8.0	179.0	179.0	179.0	179.0	179.0	179.0	179.0	179.0	8.0	179.0	179.0
6.7	6.7	6.7	179.0	179.0	179.0	179.0	179.0	179.0	179.0	179.0	6.7	179.0	179.0
42.0	42.0	42.0	179.0	179.0	179.0	179.0	179.0	179.0	179.0	179.0	42.0	179.0	179.0
42.0	42.0	42.0	179.0	179.0	179.0	179.0	179.0	179.0	179.0	179.0	42.0	179.0	179.0
42.0	42.0	42.0	179.0	179.0	179.0	179.0	179.0	179.0	179.0	179.0	42.0	179.0	179.0
42.0	42.0	42.0	179.0	179.0	179.0	179.0	179.0	179.0	179.0	179.0	42.0	179.0	179.0
179.0	179.0	179.0	44.0	44.0	127.0	117.0	127.0	127.0	44.0	44.0	179.0	122.0	44.0
179.0	179.0	179.0	117.0	117.0	127.0	107.0	127.0	127.0	117.0	117.0	179.0	122.0	117.0
179.0	179.0	179.0	122.0	122.0	127.0	122.0	127.0	127.0	122.0	122.0	179.0	45.1	122.0
179.0	179.0	179.0	122.0	122.0	127.0	122.0	127.0	127.0	122.0	122.0	179.0	45.1	122.0
179.0	179.0	179.0	122.0	122.0	127.0	122.0	127.0	127.0	122.0	122.0	179.0	51.5	122.0
179.0	179.0	179.0	122.0	122.0	127.0	122.0	127.0	127.0	122.0	122.0	179.0	51.5	122.0
179.0	179.0	179.0	31.4	31.4	127.0	117.0	127.0	127.0	31.4	31.4	179.0	122.0	22.0
179.0	179.0	179.0	44.0	44.0	127.0	117.0	127.0	127.0	44.0	44.0	179.0	122.0	44.0
179.0	179.0	179.0	44.0	44.0	127.0	117.0	127.0	127.0	44.0	44.0	179.0	122.0	44.0
179.0	179.0	179.0	117.0	117.0	127.0	107.0	127.0	127.0	117.0	117.0	179.0	122.0	117.0
179.0	179.0	179.0	31.4	31.4	127.0	117.0	127.0	127.0	31.4	31.4	179.0	122.0	28.3
6.7	6.7	6.7	179.0	179.0	179.0	179.0	179.0	179.0	179.0	179.0	6.7	179.0	179.0
179.0	179.0	179.0	127.0	127.0	47.0	127.0	90.7	47.0	127.0	127.0	179.0	127.0	127.0
179.0	179.0	179.0	31.4	31.4	127.0	117.0	127.0	127.0	31.4	31.4	179.0	122.0	22.0
179.0	179.0	179.0	31.4	31.4	127.0	117.0	127.0	127.0	31.4	31.4	179.0	122.0	22.0
179.0	179.0	179.0	31.4	31.4	127.0	117.0	127.0	127.0	31.4	31.4	179.0	122.0	22.0
5.3	5.3	5.3	179.0	179.0	179.0	179.0	179.0	179.0	179.0	179.0	4.0	179.0	179.0
12.0	12.0	12.0	179.0	179.0	179.0	179.0	179.0	179.0	179.0	179.0	12.0	179.0	179.0
179.0	179.0	179.0	107.0	107.0	127.0	117.0	127.0	127.0	107.0	107.0	179.0	122.0	107.0
3.6	3.6	3.6	179.0	179.0	179.0	179.0	179.0	179.0	179.0	179.0	5.3	179.0	179.0
12.0	12.0	12.0	179.0	179.0	179.0	179.0	179.0	179.0	179.0	179.0	12.0	179.0	179.0
12.0	12.0	12.0	179.0	179.0	179.0	179.0	179.0	179.0	179.0	179.0	12.0	179.0	179.0
12.0	12.0	12.0	179.0	179.0	179.0	179.0	179.0	179.0	179.0	179.0	12.0	179.0	179.0
179.0	179.0	179.0	117.0	117.0	127.0	107.0	127.0	127.0	117.0	117.0	179.0	122.0	117.0
8.0	8.0	8.0	179.0	179.0	179.0	179.0	179.0	179.0	179.0	179.0	8.0	179.0	179.0
179.0	179.0	179.0	127.0	127.0	112.5	127.0	112.5	112.5	127.0	127.0	179.0	127.0	127.0
179.0	179.0	179.0	127.0	127.0	112.5	127.0	112.5	112.5	127.0	127.0	179.0	127.0	127.0
179.0	179.0	179.0	127.0	127.0	112.5	127.0	112.5	112.5	127.0	127.0	179.0	127.0	127.0
400.0	400.0	400.0	400.0	400.0	400.0	400.0	400.0	400.0	400.0	400.0	400.0	400.0	400.0
179.0	179.0	179.0	31.4	31.4	127.0	117.0	127.0	127.0	31.4	31.4	179.0	122.0	28.3
179.0	179.0	179.0	127.0	127.0	112.5	127.0	112.5	112.5	127.0	127.0	179.0	127.0	127.0
179.0	179.0	179.0	34.6	34.6	127.0	117.0	127.0	127.0	34.6	34.6	179.0	122.0	34.6
0.0	1.8	1.8	179.0	179.0	179.0	179.0	179.0	179.0	179.0	179.0	5.3	179.0	179.0
1.8	0.0	1.8	179.0	179.0	179.0	179.0	179.0	179.0	179.0	179.0	5.3	179.0	179.0
1.8	1.8	0.0	179.0	179.0	179.0	179.0	179.0	179.0	179.0	179.0	5.3	179.0	179.0
179.0	179.0	179.0	0.0	7.9	127.0	117.0	127.0	127.0	15.7	23.6	179.0	122.0	31.4
179.0	179.0	179.0	7.9	0.0	127.0	117.0	127.0	127.0	15.7	23.6	179.0	122.0	31.4
179.0	179.0	179.0	127.0	127.0	0.0	127.0	90.7	23.5	127.0	127.0	179.0	127.0	127.0
179.0	179.0	179.0	117.0	117.0	127.0	0.0	127.0	127.0	117.0	117.0	179.0	122.0	117.0
179.0	179.0	179.0	127.0	127.0	90.7	127.0	0.0	90.7	127.0	127.0	179.0	127.0	127.0
179.0	179.0	179.0	127.0	127.0	23.5	127.0	90.7	0.0	127.0	127.0	179.0	127.0	127.0
179.0	179.0	179.0	15.7	15.7	127.0	117.0	127.0	127.0	0.0	23.6	179.0	122.0	31.4
179.0	179.0	179.0	23.6	23.6	127.0	117.0	127.0	127.0	23.6	0.0	179.0	122.0	31.4

5.3	5.3	5.3	179.0	179.0	179.0	179.0	179.0	179.0	179.0	179.0	0.0	179.0	179.0
179.0	179.0	179.0	122.0	122.0	127.0	122.0	127.0	127.0	122.0	122.0	179.0	0.0	122.0
179.0	179.0	179.0	31.4	31.4	127.0	117.0	127.0	127.0	31.4	31.4	179.0	122.0	0.0
179.0	179.0	179.0	31.4	31.4	127.0	117.0	127.0	127.0	31.4	31.4	179.0	122.0	11.0
179.0	179.0	179.0	31.4	31.4	127.0	117.0	127.0	127.0	31.4	31.4	179.0	122.0	11.0
179.0	179.0	179.0	31.4	31.4	127.0	117.0	127.0	127.0	31.4	31.4	179.0	122.0	11.0
179.0	179.0	179.0	31.4	31.4	127.0	117.0	127.0	127.0	31.4	31.4	179.0	122.0	11.0
179.0	179.0	179.0	127.0	127.0	76.0	127.0	90.7	76.0	127.0	127.0	179.0	127.0	127.0
5.3	5.3	5.3	179.0	179.0	179.0	179.0	179.0	179.0	179.0	179.0	2.7	179.0	179.0
5.3	5.3	5.3	179.0	179.0	179.0	179.0	179.0	179.0	179.0	179.0	2.7	179.0	179.0
8.0	8.0	8.0	179.0	179.0	179.0	179.0	179.0	179.0	179.0	179.0	8.0	179.0	179.0
8.0	8.0	8.0	179.0	179.0	179.0	179.0	179.0	179.0	179.0	179.0	8.0	179.0	179.0
179.0	179.0	179.0	127.0	127.0	98.0	127.0	98.0	98.0	127.0	127.0	179.0	127.0	127.0
179.0	179.0	179.0	31.4	31.4	127.0	117.0	127.0	127.0	31.4	31.4	179.0	122.0	22.0
179.0	179.0	179.0	90.0	90.0	127.0	117.0	127.0	127.0	90.0	90.0	179.0	122.0	90.0
42.0	42.0	42.0	179.0	179.0	179.0	179.0	179.0	179.0	179.0	179.0	42.0	179.0	179.0
42.0	42.0	42.0	179.0	179.0	179.0	179.0	179.0	179.0	179.0	179.0	42.0	179.0	179.0
179.0	179.0	179.0	31.4	31.4	127.0	117.0	127.0	127.0	31.4	31.4	179.0	122.0	22.0
179.0	179.0	179.0	31.4	31.4	127.0	117.0	127.0	127.0	31.4	31.4	179.0	122.0	22.0
179.0	179.0	179.0	31.4	31.4	127.0	117.0	127.0	127.0	31.4	31.4	179.0	122.0	22.0
179.0	179.0	179.0	127.0	127.0	112.5	127.0	112.5	112.5	127.0	127.0	179.0	127.0	127.0
179.0	179.0	179.0	40.9	40.9	127.0	117.0	127.0	127.0	40.9	40.9	179.0	122.0	40.9
179.0	179.0	179.0	117.0	117.0	127.0	63.0	127.0	127.0	117.0	117.0	179.0	122.0	117.0
8.0	8.0	8.0	179.0	179.0	179.0	179.0	179.0	179.0	179.0	179.0	8.0	179.0	179.0
179.0	179.0	179.0	127.0	127.0	90.7	127.0	56.0	90.7	127.0	127.0	179.0	127.0	127.0
179.0	179.0	179.0	127.0	127.0	90.7	127.0	37.3	90.7	127.0	127.0	179.0	127.0	127.0
179.0	179.0	179.0	127.0	127.0	90.7	127.0	37.3	90.7	127.0	127.0	179.0	127.0	127.0
42.0	42.0	42.0	179.0	179.0	179.0	179.0	179.0	179.0	179.0	179.0	42.0	179.0	179.0
179.0	179.0	179.0	127.0	127.0	47.0	127.0	90.7	47.0	127.0	127.0	179.0	127.0	127.0
179.0	179.0	179.0	40.9	40.9	127.0	117.0	127.0	127.0	40.9	40.9	179.0	122.0	40.9
179.0	179.0	179.0	127.0	127.0	90.7	127.0	56.0	90.7	127.0	127.0	179.0	127.0	127.0
10.7	10.7	10.7	179.0	179.0	179.0	179.0	179.0	179.0	179.0	179.0	10.7	179.0	179.0
179.0	179.0	179.0	122.0	122.0	127.0	122.0	127.0	127.0	122.0	122.0	179.0	45.1	122.0
450.0	450.0	450.0	450.0	450.0	450.0	450.0	450.0	450.0	450.0	450.0	450.0	450.0	450.0
500.0	500.0	500.0	500.0	500.0	500.0	500.0	500.0	500.0	500.0	500.0	500.0	500.0	500.0
179.0	179.0	179.0	117.0	117.0	127.0	107.0	127.0	127.0	117.0	117.0	179.0	122.0	117.0
179.0	179.0	179.0	127.0	127.0	112.5	127.0	112.5	112.5	127.0	127.0	179.0	127.0	127.0
179.0	179.0	179.0	127.0	127.0	90.7	127.0	56.0	90.7	127.0	127.0	179.0	127.0	127.0
179.0	179.0	179.0	147.0	147.0	147.0	147.0	147.0	147.0	147.0	147.0	179.0	147.0	147.0
179.0	179.0	179.0	147.0	147.0	147.0	147.0	147.0	147.0	147.0	147.0	179.0	147.0	147.0
179.0	179.0	179.0	122.0	122.0	127.0	122.0	127.0	127.0	122.0	122.0	179.0	45.1	122.0
6.7	6.7	6.7	179.0	179.0	179.0	179.0	179.0	179.0	179.0	179.0	6.7	179.0	179.0
12.0	12.0	12.0	179.0	179.0	179.0	179.0	179.0	179.0	179.0	179.0	12.0	179.0	179.0
179.0	179.0	179.0	31.4	31.4	127.0	117.0	127.0	127.0	31.4	31.4	179.0	122.0	22.0
325.0	325.0	325.0	325.0	325.0	325.0	325.0	325.0	325.0	325.0	325.0	325.0	325.0	325.0
179.0	179.0	179.0	117.0	117.0	127.0	63.0	127.0	127.0	117.0	117.0	179.0	122.0	117.0
179.0	179.0	179.0	117.0	117.0	127.0	63.0	127.0	127.0	117.0	117.0	179.0	122.0	117.0
179.0	179.0	179.0	117.0	117.0	127.0	63.0	127.0	127.0	117.0	117.0	179.0	122.0	117.0
5.3	5.3	5.3	179.0	179.0	179.0	179.0	179.0	179.0	179.0	179.0	5.3	179.0	179.0
5.3	5.3	5.3	179.0	179.0	179.0	179.0	179.0	179.0	179.0	179.0	5.3	179.0	179.0
5.3	5.3	5.3	179.0	179.0	179.0	179.0	179.0	179.0	179.0	179.0	5.3	179.0	179.0
5.3	5.3	5.3	179.0	179.0	179.0	179.0	179.0	179.0	179.0	179.0	5.3	179.0	179.0
179.0	179.0	179.0	122.0	122.0	127.0	122.0	127.0	127.0	122.0	122.0	179.0	84.0	122.0
179.0	179.0	179.0	122.0	122.0	127.0	122.0	127.0	127.0	122.0	122.0	179.0	84.0	122.0
179.0	179.0	179.0	127.0	127.0	98.0	127.0	98.0	98.0	127.0	127.0	179.0	127.0	127.0
179.0	179.0	179.0	127.0	127.0	98.0	127.0	98.0	98.0	127.0	127.0	179.0	127.0	127.0
179.0	179.0	179.0	127.0	127.0	11.8	127.0	90.7	23.5	127.0	127.0	179.0	127.0	127.0
179.0	179.0	179.0	117.0	117.0	127.0	23.0	127.0	127.0	117.0	117.0	179.0	122.0	117.0
179.0	179.0	179.0	127.0	127.0	47.0	127.0	90.7	47.0	127.0	127.0	179.0	127.0	127.0
179.0	179.0	179.0	127.0	127.0	90.7	127.0	56.0	90.7	127.0	127.0	179.0	127.0	127.0
179.0	179.0	179.0	127.0	127.0	35.3	127.0	90.7	35.3	127.0	127.0	179.0	127.0	127.0
179.0	179.0	179.0	127.0	127.0	35.3	127.0	90.7	35.3	127.0	127.0	179.0	127.0	127.0
179.0	179.0	179.0	122.0	122.0	127.0	122.0	127.0	127.0	122.0	122.0	179.0	84.0	122.0
179.0	179.0	179.0	122.0	122.0	127.0	122.0	127.0	127.0	122.0	122.0	179.0	84.0	122.0
179.0	179.0	179.0	122.0	122.0	127.0	122.0	127.0	127.0	122.0	122.0	179.0	84.0	122.0
179.0	179.0	179.0	122.0	122.0	127.0	122.0	127.0	127.0	122.0	122.0	179.0	84.0	122.0
179.0	179.0	179.0	122.0	122.0	127.0	122.0	127.0	127.0	122.0	122.0	179.0	45.1	122.0
179.0	179.0	179.0	127.0	127.0	98.0	127.0	98.0	98.0	127.0	127.0	179.0	127.0	127.0
179.0	179.0	179.0	127.0	127.0	98.0	127.0	98.0	98.0	127.0	127.0	179.0	127.0	127.0
179.0	179.0	179.0	127.0	127.0	98.0	127.0	98.0	98.0	127.0	127.0	179.0	127.0	127.0
179.0	179.0	179.0	122.0	122.0	127.0	122.0	127.0	127.0	122.0	122.0	179.0	51.5	122.0
179.0	179.0	179.0	122.0	122.0	127.0	122.0	127.0	127.0	122.0	122.0	179.0	45.1	122.0
179.0	179.0	179.0	122.0	122.0	127.0	122.0	127.0	127.0	122.0	122.0	179.0	45.1	122.0
179.0	179.0	179.0	37.7	37.7	127.0	117.0	127.0	127.0	37.7	37.7	179.0	122.0	37.7

179.0	179.0	179.0	37.7	37.7	127.0	117.0	127.0	127.0	37.7	37.7	179.0	122.0	37.7
179.0	179.0	179.0	37.7	37.7	127.0	117.0	127.0	127.0	37.7	37.7	179.0	122.0	37.7
12.0	12.0	12.0	179.0	179.0	179.0	179.0	179.0	179.0	179.0	179.0	12.0	179.0	179.0
12.0	12.0	12.0	179.0	179.0	179.0	179.0	179.0	179.0	179.0	179.0	12.0	179.0	179.0
179.0	179.0	179.0	122.0	122.0	127.0	122.0	127.0	127.0	122.0	122.0	179.0	45.1	122.0
179.0	179.0	179.0	127.0	127.0	112.5	127.0	112.5	112.5	127.0	127.0	179.0	127.0	127.0
179.0	179.0	179.0	127.0	127.0	112.5	127.0	112.5	112.5	127.0	127.0	179.0	127.0	127.0
179.0	179.0	179.0	127.0	127.0	112.5	127.0	112.5	112.5	127.0	127.0	179.0	127.0	127.0
179.0	179.0	179.0	117.0	117.0	127.0	107.0	127.0	127.0	117.0	117.0	179.0	122.0	117.0
179.0	179.0	179.0	31.4	31.4	127.0	117.0	127.0	127.0	31.4	31.4	179.0	122.0	28.3
179.0	179.0	179.0	31.4	31.4	127.0	117.0	127.0	127.0	31.4	31.4	179.0	122.0	22.0
179.0	179.0	179.0	31.4	31.4	127.0	117.0	127.0	127.0	31.4	31.4	179.0	122.0	22.0
179.0	179.0	179.0	122.0	122.0	127.0	122.0	127.0	127.0	122.0	122.0	179.0	45.1	122.0
179.0	179.0	179.0	122.0	122.0	127.0	122.0	127.0	127.0	122.0	122.0	179.0	45.1	122.0
179.0	179.0	179.0	40.9	40.9	127.0	117.0	127.0	127.0	40.9	40.9	179.0	122.0	40.9
179.0	179.0	179.0	31.4	31.4	127.0	117.0	127.0	127.0	31.4	31.4	179.0	122.0	22.0
179.0	179.0	179.0	107.0	107.0	127.0	117.0	127.0	127.0	107.0	107.0	179.0	122.0	107.0
179.0	179.0	179.0	31.4	31.4	127.0	117.0	127.0	127.0	31.4	31.4	179.0	122.0	25.1
179.0	179.0	179.0	127.0	127.0	90.7	127.0	56.0	90.7	127.0	127.0	179.0	127.0	127.0
179.0	179.0	179.0	127.0	127.0	90.7	127.0	56.0	90.7	127.0	127.0	179.0	127.0	127.0
179.0	179.0	179.0	127.0	127.0	90.7	127.0	56.0	90.7	127.0	127.0	179.0	127.0	127.0
179.0	179.0	179.0	127.0	127.0	90.7	127.0	56.0	90.7	127.0	127.0	179.0	127.0	127.0
179.0	179.0	179.0	127.0	127.0	90.7	127.0	56.0	90.7	127.0	127.0	179.0	127.0	127.0
179.0	179.0	179.0	40.9	40.9	127.0	117.0	127.0	127.0	40.9	40.9	179.0	122.0	40.9
179.0	179.0	179.0	37.7	37.7	127.0	117.0	127.0	127.0	37.7	37.7	179.0	122.0	37.7
72.0	72.0	72.0	179.0	179.0	179.0	179.0	179.0	179.0	179.0	179.0	72.0	179.0	179.0
179.0	179.0	179.0	127.0	127.0	76.0	127.0	90.7	76.0	127.0	127.0	179.0	127.0	127.0
179.0	179.0	179.0	117.0	117.0	127.0	43.0	127.0	127.0	117.0	117.0	179.0	122.0	117.0
179.0	179.0	179.0	117.0	117.0	127.0	63.0	127.0	127.0	117.0	117.0	179.0	122.0	117.0
179.0	179.0	179.0	117.0	117.0	127.0	63.0	127.0	127.0	117.0	117.0	179.0	122.0	117.0
179.0	179.0	179.0	127.0	127.0	90.7	127.0	56.0	90.7	127.0	127.0	179.0	127.0	127.0
179.0	179.0	179.0	127.0	127.0	90.7	127.0	56.0	90.7	127.0	127.0	179.0	127.0	127.0
179.0	179.0	179.0	127.0	127.0	90.7	127.0	56.0	90.7	127.0	127.0	179.0	127.0	127.0
179.0	179.0	179.0	127.0	127.0	90.7	127.0	56.0	90.7	127.0	127.0	179.0	127.0	127.0
179.0	179.0	179.0	127.0	127.0	98.0	127.0	98.0	98.0	127.0	127.0	179.0	127.0	127.0
179.0	179.0	179.0	127.0	127.0	98.0	127.0	98.0	98.0	127.0	127.0	179.0	127.0	127.0
179.0	179.0	179.0	127.0	127.0	98.0	127.0	98.0	98.0	127.0	127.0	179.0	127.0	127.0

Hieracium_	Hieracium_	Hieracium_	Hieracium_	Hippophae	Holcus_lan	Holcus_mo	Hordeum_j	Hordeum_	Hypericum	Hypochaer	Jasione_mo	Juncus_arti	Juncus_buf
40.9	40.9	40.9	40.9	127.0	179.0	179.0	179.0	179.0	127.0	40.9	90.0	179.0	179.0
179.0	179.0	179.0	179.0	179.0	6.7	6.7	8.0	8.0	179.0	179.0	179.0	42.0	42.0
179.0	179.0	179.0	179.0	179.0	6.7	6.7	8.0	8.0	179.0	179.0	179.0	42.0	42.0
179.0	179.0	179.0	179.0	179.0	6.7	6.7	8.0	8.0	179.0	179.0	179.0	42.0	42.0
117.0	117.0	117.0	117.0	127.0	179.0	179.0	179.0	179.0	127.0	117.0	117.0	179.0	179.0
179.0	179.0	179.0	179.0	179.0	5.3	5.3	8.0	8.0	179.0	179.0	179.0	42.0	42.0
44.0	44.0	44.0	44.0	127.0	179.0	179.0	179.0	179.0	127.0	44.0	90.0	179.0	179.0
122.0	122.0	122.0	122.0	127.0	179.0	179.0	179.0	179.0	127.0	122.0	122.0	179.0	179.0
40.9	40.9	40.9	40.9	127.0	179.0	179.0	179.0	179.0	127.0	40.9	90.0	179.0	179.0
40.9	40.9	40.9	40.9	127.0	179.0	179.0	179.0	179.0	127.0	40.9	90.0	179.0	179.0
127.0	127.0	127.0	127.0	112.5	179.0	179.0	179.0	179.0	112.5	127.0	127.0	179.0	179.0
127.0	127.0	127.0	127.0	83.3	179.0	179.0	179.0	179.0	98.0	127.0	127.0	179.0	179.0
179.0	179.0	179.0	179.0	179.0	9.3	9.3	9.3	9.3	179.0	179.0	179.0	42.0	42.0
179.0	179.0	179.0	179.0	179.0	8.0	8.0	4.8	4.8	179.0	179.0	179.0	42.0	42.0
179.0	179.0	179.0	179.0	179.0	8.0	8.0	4.8	4.8	179.0	179.0	179.0	42.0	42.0
179.0	179.0	179.0	179.0	179.0	6.7	6.7	8.0	8.0	179.0	179.0	179.0	42.0	42.0
179.0	179.0	179.0	179.0	179.0	42.0	42.0	42.0	42.0	179.0	179.0	179.0	31.5	31.5
179.0	179.0	179.0	179.0	179.0	42.0	42.0	42.0	42.0	179.0	179.0	179.0	31.5	31.5
179.0	179.0	179.0	179.0	179.0	42.0	42.0	42.0	42.0	179.0	179.0	179.0	31.5	31.5
179.0	179.0	179.0	179.0	179.0	42.0	42.0	42.0	42.0	179.0	179.0	179.0	31.5	31.5
44.0	44.0	44.0	44.0	127.0	179.0	179.0	179.0	179.0	127.0	44.0	90.0	179.0	179.0
117.0	117.0	117.0	117.0	127.0	179.0	179.0	179.0	179.0	127.0	117.0	117.0	179.0	179.0
122.0	122.0	122.0	122.0	127.0	179.0	179.0	179.0	179.0	127.0	122.0	122.0	179.0	179.0
122.0	122.0	122.0	122.0	127.0	179.0	179.0	179.0	179.0	127.0	122.0	122.0	179.0	179.0
122.0	122.0	122.0	122.0	127.0	179.0	179.0	179.0	179.0	127.0	122.0	122.0	179.0	179.0
122.0	122.0	122.0	122.0	127.0	179.0	179.0	179.0	179.0	127.0	122.0	122.0	179.0	179.0
22.0	22.0	22.0	22.0	127.0	179.0	179.0	179.0	179.0	127.0	12.6	90.0	179.0	179.0
44.0	44.0	44.0	44.0	127.0	179.0	179.0	179.0	179.0	127.0	44.0	90.0	179.0	179.0
44.0	44.0	44.0	44.0	127.0	179.0	179.0	179.0	179.0	127.0	44.0	90.0	179.0	179.0
117.0	117.0	117.0	117.0	127.0	179.0	179.0	179.0	179.0	127.0	117.0	117.0	179.0	179.0
28.3	28.3	28.3	28.3	127.0	179.0	179.0	179.0	179.0	127.0	28.3	90.0	179.0	179.0
179.0	179.0	179.0	179.0	179.0	6.7	6.7	8.0	8.0	179.0	179.0	179.0	42.0	42.0
127.0	127.0	127.0	127.0	76.0	179.0	179.0	179.0	179.0	98.0	127.0	127.0	179.0	179.0
22.0	22.0	22.0	22.0	127.0	179.0	179.0	179.0	179.0	127.0	12.6	90.0	179.0	179.0
22.0	22.0	22.0	22.0	127.0	179.0	179.0	179.0	179.0	127.0	12.6	90.0	179.0	179.0
22.0	22.0	22.0	22.0	127.0	179.0	179.0	179.0	179.0	127.0	12.6	90.0	179.0	179.0
179.0	179.0	179.0	179.0	179.0	4.0	4.0	8.0	8.0	179.0	179.0	179.0	42.0	42.0
179.0	179.0	179.0	179.0	179.0	12.0	12.0	12.0	12.0	179.0	179.0	179.0	42.0	42.0
107.0	107.0	107.0	107.0	127.0	179.0	179.0	179.0	179.0	127.0	107.0	107.0	179.0	179.0
179.0	179.0	179.0	179.0	179.0	5.3	5.3	8.0	8.0	179.0	179.0	179.0	42.0	42.0
179.0	179.0	179.0	179.0	179.0	12.0	12.0	12.0	12.0	179.0	179.0	179.0	42.0	42.0
179.0	179.0	179.0	179.0	179.0	12.0	12.0	12.0	12.0	179.0	179.0	179.0	42.0	42.0
179.0	179.0	179.0	179.0	179.0	12.0	12.0	12.0	12.0	179.0	179.0	179.0	42.0	42.0
117.0	117.0	117.0	117.0	127.0	179.0	179.0	179.0	179.0	127.0	117.0	117.0	179.0	179.0
179.0	179.0	179.0	179.0	179.0	8.0	8.0	3.2	3.2	179.0	179.0	179.0	42.0	42.0
127.0	127.0	127.0	127.0	112.5	179.0	179.0	179.0	179.0	112.5	127.0	127.0	179.0	179.0
127.0	127.0	127.0	127.0	112.5	179.0	179.0	179.0	179.0	112.5	127.0	127.0	179.0	179.0
127.0	127.0	127.0	127.0	112.5	179.0	179.0	179.0	179.0	112.5	127.0	127.0	179.0	179.0
400.0	400.0	400.0	400.0	400.0	400.0	400.0	400.0	400.0	400.0	400.0	400.0	400.0	400.0
28.3	28.3	28.3	28.3	127.0	179.0	179.0	179.0	179.0	127.0	28.3	90.0	179.0	179.0
127.0	127.0	127.0	127.0	112.5	179.0	179.0	179.0	179.0	112.5	127.0	127.0	179.0	179.0
34.6	34.6	34.6	34.6	127.0	179.0	179.0	179.0	179.0	127.0	34.6	90.0	179.0	179.0
179.0	179.0	179.0	179.0	179.0	5.3	5.3	8.0	8.0	179.0	179.0	179.0	42.0	42.0
179.0	179.0	179.0	179.0	179.0	5.3	5.3	8.0	8.0	179.0	179.0	179.0	42.0	42.0
179.0	179.0	179.0	179.0	179.0	5.3	5.3	8.0	8.0	179.0	179.0	179.0	42.0	42.0
31.4	31.4	31.4	31.4	127.0	179.0	179.0	179.0	179.0	127.0	31.4	90.0	179.0	179.0
31.4	31.4	31.4	31.4	127.0	179.0	179.0	179.0	179.0	127.0	31.4	90.0	179.0	179.0
127.0	127.0	127.0	127.0	76.0	179.0	179.0	179.0	179.0	98.0	127.0	127.0	179.0	179.0
117.0	117.0	117.0	117.0	127.0	179.0	179.0	179.0	179.0	127.0	117.0	117.0	179.0	179.0
127.0	127.0	127.0	127.0	90.7	179.0	179.0	179.0	179.0	98.0	127.0	127.0	179.0	179.0
127.0	127.0	127.0	127.0	76.0	179.0	179.0	179.0	179.0	98.0	127.0	127.0	179.0	179.0
31.4	31.4	31.4	31.4	127.0	179.0	179.0	179.0	179.0	127.0	31.4	90.0	179.0	179.0
31.4	31.4	31.4	31.4	127.0	179.0	179.0	179.0	179.0	127.0	31.4	90.0	179.0	179.0

179.0	179.0	179.0	179.0	179.0	2.7	2.7	8.0	8.0	179.0	179.0	179.0	42.0	42.0
122.0	122.0	122.0	122.0	127.0	179.0	179.0	179.0	179.0	127.0	122.0	122.0	179.0	179.0
11.0	11.0	11.0	11.0	127.0	179.0	179.0	179.0	179.0	127.0	22.0	90.0	179.0	179.0
0.0	11.0	11.0	11.0	127.0	179.0	179.0	179.0	179.0	127.0	22.0	90.0	179.0	179.0
11.0	0.0	11.0	11.0	127.0	179.0	179.0	179.0	179.0	127.0	22.0	90.0	179.0	179.0
11.0	11.0	0.0	11.0	127.0	179.0	179.0	179.0	179.0	127.0	22.0	90.0	179.0	179.0
11.0	11.0	11.0	0.0	127.0	179.0	179.0	179.0	179.0	127.0	22.0	90.0	179.0	179.0
127.0	127.0	127.0	127.0	0.0	179.0	179.0	179.0	179.0	98.0	127.0	127.0	179.0	179.0
179.0	179.0	179.0	179.0	179.0	0.0	1.3	8.0	8.0	179.0	179.0	179.0	42.0	42.0
179.0	179.0	179.0	179.0	179.0	1.3	0.0	8.0	8.0	179.0	179.0	179.0	42.0	42.0
179.0	179.0	179.0	179.0	179.0	8.0	8.0	0.0	1.6	179.0	179.0	179.0	42.0	42.0
179.0	179.0	179.0	179.0	179.0	8.0	8.0	1.6	0.0	179.0	179.0	179.0	42.0	42.0
127.0	127.0	127.0	127.0	98.0	179.0	179.0	179.0	179.0	0.0	127.0	127.0	179.0	179.0
22.0	22.0	22.0	22.0	127.0	179.0	179.0	179.0	179.0	127.0	0.0	90.0	179.0	179.0
90.0	90.0	90.0	90.0	127.0	179.0	179.0	179.0	179.0	127.0	90.0	0.0	179.0	179.0
179.0	179.0	179.0	179.0	179.0	42.0	42.0	42.0	42.0	179.0	179.0	179.0	0.0	10.5
179.0	179.0	179.0	179.0	179.0	42.0	42.0	42.0	42.0	179.0	179.0	179.0	10.5	0.0
22.0	22.0	22.0	22.0	127.0	179.0	179.0	179.0	179.0	127.0	18.9	90.0	179.0	179.0
22.0	22.0	22.0	22.0	127.0	179.0	179.0	179.0	179.0	127.0	9.4	90.0	179.0	179.0
22.0	22.0	22.0	22.0	127.0	179.0	179.0	179.0	179.0	127.0	9.4	90.0	179.0	179.0
127.0	127.0	127.0	127.0	112.5	179.0	179.0	179.0	179.0	112.5	127.0	127.0	179.0	179.0
40.9	40.9	40.9	40.9	127.0	179.0	179.0	179.0	179.0	127.0	40.9	90.0	179.0	179.0
117.0	117.0	117.0	117.0	127.0	179.0	179.0	179.0	179.0	127.0	117.0	117.0	179.0	179.0
179.0	179.0	179.0	179.0	179.0	8.0	8.0	6.4	6.4	179.0	179.0	179.0	42.0	42.0
127.0	127.0	127.0	127.0	90.7	179.0	179.0	179.0	179.0	98.0	127.0	127.0	179.0	179.0
127.0	127.0	127.0	127.0	90.7	179.0	179.0	179.0	179.0	98.0	127.0	127.0	179.0	179.0
127.0	127.0	127.0	127.0	90.7	179.0	179.0	179.0	179.0	98.0	127.0	127.0	179.0	179.0
179.0	179.0	179.0	179.0	179.0	42.0	42.0	42.0	42.0	179.0	179.0	179.0	21.0	21.0
127.0	127.0	127.0	127.0	76.0	179.0	179.0	179.0	179.0	98.0	127.0	127.0	179.0	179.0
40.9	40.9	40.9	40.9	127.0	179.0	179.0	179.0	179.0	127.0	40.9	90.0	179.0	179.0
127.0	127.0	127.0	127.0	90.7	179.0	179.0	179.0	179.0	98.0	127.0	127.0	179.0	179.0
179.0	179.0	179.0	179.0	179.0	10.7	10.7	10.7	10.7	179.0	179.0	179.0	42.0	42.0
122.0	122.0	122.0	122.0	127.0	179.0	179.0	179.0	179.0	127.0	122.0	122.0	179.0	179.0
450.0	450.0	450.0	450.0	450.0	450.0	450.0	450.0	450.0	450.0	450.0	450.0	450.0	450.0
500.0	500.0	500.0	500.0	500.0	500.0	500.0	500.0	500.0	500.0	500.0	500.0	500.0	500.0
117.0	117.0	117.0	117.0	127.0	179.0	179.0	179.0	179.0	127.0	117.0	117.0	179.0	179.0
127.0	127.0	127.0	127.0	112.5	179.0	179.0	179.0	179.0	112.5	127.0	127.0	179.0	179.0
127.0	127.0	127.0	127.0	90.7	179.0	179.0	179.0	179.0	98.0	127.0	127.0	179.0	179.0
147.0	147.0	147.0	147.0	147.0	179.0	179.0	179.0	179.0	147.0	147.0	147.0	179.0	179.0
147.0	147.0	147.0	147.0	147.0	179.0	179.0	179.0	179.0	147.0	147.0	147.0	179.0	179.0
122.0	122.0	122.0	122.0	127.0	179.0	179.0	179.0	179.0	127.0	122.0	122.0	179.0	179.0
179.0	179.0	179.0	179.0	179.0	6.7	6.7	8.0	8.0	179.0	179.0	179.0	42.0	42.0
179.0	179.0	179.0	179.0	179.0	12.0	12.0	12.0	12.0	179.0	179.0	179.0	42.0	42.0
22.0	22.0	22.0	22.0	127.0	179.0	179.0	179.0	179.0	127.0	9.4	90.0	179.0	179.0
325.0	325.0	325.0	325.0	325.0	325.0	325.0	325.0	325.0	325.0	325.0	325.0	325.0	325.0
117.0	117.0	117.0	117.0	127.0	179.0	179.0	179.0	179.0	127.0	117.0	117.0	179.0	179.0
117.0	117.0	117.0	117.0	127.0	179.0	179.0	179.0	179.0	127.0	117.0	117.0	179.0	179.0
117.0	117.0	117.0	117.0	127.0	179.0	179.0	179.0	179.0	127.0	117.0	117.0	179.0	179.0
179.0	179.0	179.0	179.0	179.0	5.3	5.3	8.0	8.0	179.0	179.0	179.0	42.0	42.0
179.0	179.0	179.0	179.0	179.0	5.3	5.3	8.0	8.0	179.0	179.0	179.0	42.0	42.0
179.0	179.0	179.0	179.0	179.0	5.3	5.3	8.0	8.0	179.0	179.0	179.0	42.0	42.0
179.0	179.0	179.0	179.0	179.0	5.3	5.3	8.0	8.0	179.0	179.0	179.0	42.0	42.0
122.0	122.0	122.0	122.0	127.0	179.0	179.0	179.0	179.0	127.0	122.0	122.0	179.0	179.0
122.0	122.0	122.0	122.0	127.0	179.0	179.0	179.0	179.0	127.0	122.0	122.0	179.0	179.0
127.0	127.0	127.0	127.0	98.0	179.0	179.0	179.0	179.0	81.0	127.0	127.0	179.0	179.0
127.0	127.0	127.0	127.0	98.0	179.0	179.0	179.0	179.0	81.0	127.0	127.0	179.0	179.0
127.0	127.0	127.0	127.0	76.0	179.0	179.0	179.0	179.0	98.0	127.0	127.0	179.0	179.0
117.0	117.0	117.0	117.0	127.0	179.0	179.0	179.0	179.0	127.0	117.0	117.0	179.0	179.0
127.0	127.0	127.0	127.0	76.0	179.0	179.0	179.0	179.0	98.0	127.0	127.0	179.0	179.0
127.0	127.0	127.0	127.0	90.7	179.0	179.0	179.0	179.0	98.0	127.0	127.0	179.0	179.0
127.0	127.0	127.0	127.0	76.0	179.0	179.0	179.0	179.0	98.0	127.0	127.0	179.0	179.0
127.0	127.0	127.0	127.0	76.0	179.0	179.0	179.0	179.0	98.0	127.0	127.0	179.0	179.0
122.0	122.0	122.0	122.0	127.0	179.0	179.0	179.0	179.0	127.0	122.0	122.0	179.0	179.0
122.0	122.0	122.0	122.0	127.0	179.0	179.0	179.0	179.0	127.0	122.0	122.0	179.0	179.0
122.0	122.0	122.0	122.0	127.0	179.0	179.0	179.0	179.0	127.0	122.0	122.0	179.0	179.0
122.0	122.0	122.0	122.0	127.0	179.0	179.0	179.0	179.0	127.0	122.0	122.0	179.0	179.0
122.0	122.0	122.0	122.0	127.0	179.0	179.0	179.0	179.0	127.0	122.0	122.0	179.0	179.0
127.0	127.0	127.0	127.0	98.0	179.0	179.0	179.0	179.0	81.0	127.0	127.0	179.0	179.0
127.0	127.0	127.0	127.0	98.0	179.0	179.0	179.0	179.0	81.0	127.0	127.0	179.0	179.0
127.0	127.0	127.0	127.0	98.0	179.0	179.0	179.0	179.0	81.0	127.0	127.0	179.0	179.0
122.0	122.0	122.0	122.0	127.0	179.0	179.0	179.0	179.0	127.0	122.0	122.0	179.0	179.0
122.0	122.0	122.0	122.0	127.0	179.0	179.0	179.0	179.0	127.0	122.0	122.0	179.0	179.0
122.0	122.0	122.0	122.0	127.0	179.0	179.0	179.0	179.0	127.0	122.0	122.0	179.0	179.0
37.7	37.7	37.7	37.7	127.0	179.0	179.0	179.0	179.0	127.0	37.7	90.0	179.0	179.0

37.7	37.7	37.7	37.7	127.0	179.0	179.0	179.0	179.0	127.0	37.7	90.0	179.0	179.0
37.7	37.7	37.7	37.7	127.0	179.0	179.0	179.0	179.0	127.0	37.7	90.0	179.0	179.0
179.0	179.0	179.0	179.0	179.0	12.0	12.0	12.0	12.0	179.0	179.0	179.0	42.0	42.0
179.0	179.0	179.0	179.0	179.0	12.0	12.0	12.0	12.0	179.0	179.0	179.0	42.0	42.0
122.0	122.0	122.0	122.0	127.0	179.0	179.0	179.0	179.0	127.0	122.0	122.0	179.0	179.0
127.0	127.0	127.0	127.0	112.5	179.0	179.0	179.0	179.0	112.5	127.0	127.0	179.0	179.0
127.0	127.0	127.0	127.0	112.5	179.0	179.0	179.0	179.0	112.5	127.0	127.0	179.0	179.0
127.0	127.0	127.0	127.0	112.5	179.0	179.0	179.0	179.0	112.5	127.0	127.0	179.0	179.0
117.0	117.0	117.0	117.0	127.0	179.0	179.0	179.0	179.0	127.0	117.0	117.0	179.0	179.0
28.3	28.3	28.3	28.3	127.0	179.0	179.0	179.0	179.0	127.0	28.3	90.0	179.0	179.0
22.0	22.0	22.0	22.0	127.0	179.0	179.0	179.0	179.0	127.0	15.7	90.0	179.0	179.0
22.0	22.0	22.0	22.0	127.0	179.0	179.0	179.0	179.0	127.0	15.7	90.0	179.0	179.0
122.0	122.0	122.0	122.0	127.0	179.0	179.0	179.0	179.0	127.0	122.0	122.0	179.0	179.0
122.0	122.0	122.0	122.0	127.0	179.0	179.0	179.0	179.0	127.0	122.0	122.0	179.0	179.0
40.9	40.9	40.9	40.9	127.0	179.0	179.0	179.0	179.0	127.0	40.9	90.0	179.0	179.0
22.0	22.0	22.0	22.0	127.0	179.0	179.0	179.0	179.0	127.0	12.6	90.0	179.0	179.0
107.0	107.0	107.0	107.0	127.0	179.0	179.0	179.0	179.0	127.0	107.0	107.0	179.0	179.0
25.1	25.1	25.1	25.1	127.0	179.0	179.0	179.0	179.0	127.0	25.1	90.0	179.0	179.0
127.0	127.0	127.0	127.0	90.7	179.0	179.0	179.0	179.0	98.0	127.0	127.0	179.0	179.0
127.0	127.0	127.0	127.0	90.7	179.0	179.0	179.0	179.0	98.0	127.0	127.0	179.0	179.0
127.0	127.0	127.0	127.0	90.7	179.0	179.0	179.0	179.0	98.0	127.0	127.0	179.0	179.0
127.0	127.0	127.0	127.0	90.7	179.0	179.0	179.0	179.0	98.0	127.0	127.0	179.0	179.0
127.0	127.0	127.0	127.0	90.7	179.0	179.0	179.0	179.0	98.0	127.0	127.0	179.0	179.0
40.9	40.9	40.9	40.9	127.0	179.0	179.0	179.0	179.0	127.0	40.9	90.0	179.0	179.0
37.7	37.7	37.7	37.7	127.0	179.0	179.0	179.0	179.0	127.0	37.7	90.0	179.0	179.0
179.0	179.0	179.0	179.0	179.0	72.0	72.0	72.0	72.0	179.0	179.0	179.0	72.0	72.0
127.0	127.0	127.0	127.0	38.0	179.0	179.0	179.0	179.0	98.0	127.0	127.0	179.0	179.0
117.0	117.0	117.0	117.0	127.0	179.0	179.0	179.0	179.0	127.0	117.0	117.0	179.0	179.0
117.0	117.0	117.0	117.0	127.0	179.0	179.0	179.0	179.0	127.0	117.0	117.0	179.0	179.0
117.0	117.0	117.0	117.0	127.0	179.0	179.0	179.0	179.0	127.0	117.0	117.0	179.0	179.0
127.0	127.0	127.0	127.0	90.7	179.0	179.0	179.0	179.0	98.0	127.0	127.0	179.0	179.0
127.0	127.0	127.0	127.0	90.7	179.0	179.0	179.0	179.0	98.0	127.0	127.0	179.0	179.0
127.0	127.0	127.0	127.0	90.7	179.0	179.0	179.0	179.0	98.0	127.0	127.0	179.0	179.0
127.0	127.0	127.0	127.0	90.7	179.0	179.0	179.0	179.0	98.0	127.0	127.0	179.0	179.0
127.0	127.0	127.0	127.0	98.0	179.0	179.0	179.0	179.0	54.0	127.0	127.0	179.0	179.0
127.0	127.0	127.0	127.0	98.0	179.0	179.0	179.0	179.0	54.0	127.0	127.0	179.0	179.0
127.0	127.0	127.0	127.0	98.0	179.0	179.0	179.0	179.0	54.0	127.0	127.0	179.0	179.0

Lactuca_se	Leontodon_	Leontodon_	Lepidium_r	Leucanther	Linaria_vul	Lolium_per	Lotus_corn	Lupinus_lu	Lupinus_pc	Luzula_mu	Malus_don	Matricaria_	Medicago_
40.9	40.9	40.9	127.0	24.5	117.0	179.0	127.0	127.0	127.0	179.0	127.0	8.2	127.0
179.0	179.0	179.0	179.0	179.0	179.0	8.0	179.0	179.0	179.0	42.0	179.0	179.0	179.0
179.0	179.0	179.0	179.0	179.0	179.0	8.0	179.0	179.0	179.0	42.0	179.0	179.0	179.0
179.0	179.0	179.0	179.0	179.0	179.0	8.0	179.0	179.0	179.0	42.0	179.0	179.0	179.0
117.0	117.0	117.0	127.0	117.0	63.0	179.0	127.0	127.0	127.0	179.0	127.0	117.0	127.0
179.0	179.0	179.0	179.0	179.0	179.0	8.0	179.0	179.0	179.0	42.0	179.0	179.0	179.0
44.0	44.0	44.0	127.0	44.0	117.0	179.0	127.0	127.0	127.0	179.0	127.0	44.0	127.0
122.0	122.0	122.0	127.0	122.0	122.0	179.0	127.0	127.0	127.0	179.0	127.0	122.0	127.0
40.9	40.9	40.9	127.0	32.7	117.0	179.0	127.0	127.0	127.0	179.0	127.0	32.7	127.0
40.9	40.9	40.9	127.0	32.7	117.0	179.0	127.0	127.0	127.0	179.0	127.0	32.7	127.0
127.0	127.0	127.0	24.0	127.0	127.0	179.0	112.5	112.5	112.5	179.0	112.5	127.0	112.5
127.0	127.0	127.0	112.5	127.0	127.0	179.0	90.7	90.7	90.7	179.0	83.3	127.0	90.7
179.0	179.0	179.0	179.0	179.0	179.0	9.3	179.0	179.0	179.0	42.0	179.0	179.0	179.0
179.0	179.0	179.0	179.0	179.0	179.0	6.4	179.0	179.0	179.0	42.0	179.0	179.0	179.0
179.0	179.0	179.0	179.0	179.0	179.0	6.4	179.0	179.0	179.0	42.0	179.0	179.0	179.0
179.0	179.0	179.0	179.0	179.0	179.0	8.0	179.0	179.0	179.0	42.0	179.0	179.0	179.0
179.0	179.0	179.0	179.0	179.0	179.0	42.0	179.0	179.0	179.0	31.5	179.0	179.0	179.0
179.0	179.0	179.0	179.0	179.0	179.0	42.0	179.0	179.0	179.0	31.5	179.0	179.0	179.0
179.0	179.0	179.0	179.0	179.0	179.0	42.0	179.0	179.0	179.0	31.5	179.0	179.0	179.0
179.0	179.0	179.0	179.0	179.0	179.0	42.0	179.0	179.0	179.0	31.5	179.0	179.0	179.0
44.0	44.0	44.0	127.0	44.0	117.0	179.0	127.0	127.0	127.0	179.0	127.0	44.0	127.0
117.0	117.0	117.0	127.0	117.0	107.0	179.0	127.0	127.0	127.0	179.0	127.0	117.0	127.0
122.0	122.0	122.0	127.0	122.0	122.0	179.0	127.0	127.0	127.0	179.0	127.0	122.0	127.0
122.0	122.0	122.0	127.0	122.0	122.0	179.0	127.0	127.0	127.0	179.0	127.0	122.0	127.0
122.0	122.0	122.0	127.0	122.0	122.0	179.0	127.0	127.0	127.0	179.0	127.0	122.0	127.0
122.0	122.0	122.0	127.0	122.0	122.0	179.0	127.0	127.0	127.0	179.0	127.0	122.0	127.0
18.9	12.6	12.6	127.0	40.9	117.0	179.0	127.0	127.0	127.0	179.0	127.0	40.9	127.0
44.0	44.0	44.0	127.0	44.0	117.0	179.0	127.0	127.0	127.0	179.0	127.0	44.0	127.0
44.0	44.0	44.0	127.0	44.0	117.0	179.0	127.0	127.0	127.0	179.0	127.0	44.0	127.0
117.0	117.0	117.0	127.0	117.0	107.0	179.0	127.0	127.0	127.0	179.0	127.0	117.0	127.0
28.3	28.3	28.3	127.0	40.9	117.0	179.0	127.0	127.0	127.0	179.0	127.0	40.9	127.0
179.0	179.0	179.0	179.0	179.0	179.0	8.0	179.0	179.0	179.0	42.0	179.0	179.0	179.0
127.0	127.0	127.0	112.5	127.0	127.0	179.0	90.7	90.7	90.7	179.0	15.7	127.0	90.7
18.9	12.6	12.6	127.0	40.9	117.0	179.0	127.0	127.0	127.0	179.0	127.0	40.9	127.0
18.9	12.6	12.6	127.0	40.9	117.0	179.0	127.0	127.0	127.0	179.0	127.0	40.9	127.0
18.9	12.6	12.6	127.0	40.9	117.0	179.0	127.0	127.0	127.0	179.0	127.0	40.9	127.0
179.0	179.0	179.0	179.0	179.0	179.0	8.0	179.0	179.0	179.0	42.0	179.0	179.0	179.0
179.0	179.0	179.0	179.0	179.0	179.0	12.0	179.0	179.0	179.0	42.0	179.0	179.0	179.0
107.0	107.0	107.0	127.0	107.0	117.0	179.0	127.0	127.0	127.0	179.0	127.0	107.0	127.0
179.0	179.0	179.0	179.0	179.0	179.0	8.0	179.0	179.0	179.0	42.0	179.0	179.0	179.0
179.0	179.0	179.0	179.0	179.0	179.0	12.0	179.0	179.0	179.0	42.0	179.0	179.0	179.0
179.0	179.0	179.0	179.0	179.0	179.0	12.0	179.0	179.0	179.0	42.0	179.0	179.0	179.0
179.0	179.0	179.0	179.0	179.0	179.0	12.0	179.0	179.0	179.0	42.0	179.0	179.0	179.0
117.0	117.0	117.0	127.0	117.0	107.0	179.0	127.0	127.0	127.0	179.0	127.0	117.0	127.0
179.0	179.0	179.0	179.0	179.0	179.0	6.4	179.0	179.0	179.0	42.0	179.0	179.0	179.0
127.0	127.0	127.0	95.0	127.0	127.0	179.0	112.5	112.5	112.5	179.0	112.5	127.0	112.5
127.0	127.0	127.0	95.0	127.0	127.0	179.0	112.5	112.5	112.5	179.0	112.5	127.0	112.5
127.0	127.0	127.0	95.0	127.0	127.0	179.0	112.5	112.5	112.5	179.0	112.5	127.0	112.5
400.0	400.0	400.0	400.0	400.0	400.0	400.0	400.0	400.0	400.0	400.0	400.0	400.0	400.0
28.3	28.3	28.3	127.0	40.9	117.0	179.0	127.0	127.0	127.0	179.0	127.0	40.9	127.0
127.0	127.0	127.0	95.0	127.0	127.0	179.0	112.5	112.5	112.5	179.0	112.5	127.0	112.5
34.6	34.6	34.6	127.0	40.9	117.0	179.0	127.0	127.0	127.0	179.0	127.0	40.9	127.0
179.0	179.0	179.0	179.0	179.0	179.0	8.0	179.0	179.0	179.0	42.0	179.0	179.0	179.0
179.0	179.0	179.0	179.0	179.0	179.0	8.0	179.0	179.0	179.0	42.0	179.0	179.0	179.0
179.0	179.0	179.0	179.0	179.0	179.0	8.0	179.0	179.0	179.0	42.0	179.0	179.0	179.0
31.4	31.4	31.4	127.0	40.9	117.0	179.0	127.0	127.0	127.0	179.0	127.0	40.9	127.0
31.4	31.4	31.4	127.0	40.9	117.0	179.0	127.0	127.0	127.0	179.0	127.0	40.9	127.0
127.0	127.0	127.0	112.5	127.0	127.0	179.0	90.7	90.7	90.7	179.0	47.0	127.0	90.7
117.0	117.0	117.0	127.0	117.0	63.0	179.0	127.0	127.0	127.0	179.0	127.0	117.0	127.0
127.0	127.0	127.0	112.5	127.0	127.0	179.0	56.0	37.3	37.3	179.0	90.7	127.0	56.0
127.0	127.0	127.0	112.5	127.0	127.0	179.0	90.7	90.7	90.7	179.0	47.0	127.0	90.7
31.4	31.4	31.4	127.0	40.9	117.0	179.0	127.0	127.0	127.0	179.0	127.0	40.9	127.0
31.4	31.4	31.4	127.0	40.9	117.0	179.0	127.0	127.0	127.0	179.0	127.0	40.9	127.0

179.0	179.0	179.0	179.0	179.0	179.0	8.0	179.0	179.0	179.0	42.0	179.0	179.0	179.0
122.0	122.0	122.0	127.0	122.0	122.0	179.0	127.0	127.0	127.0	179.0	127.0	122.0	127.0
22.0	22.0	22.0	127.0	40.9	117.0	179.0	127.0	127.0	127.0	179.0	127.0	40.9	127.0
22.0	22.0	22.0	127.0	40.9	117.0	179.0	127.0	127.0	127.0	179.0	127.0	40.9	127.0
22.0	22.0	22.0	127.0	40.9	117.0	179.0	127.0	127.0	127.0	179.0	127.0	40.9	127.0
22.0	22.0	22.0	127.0	40.9	117.0	179.0	127.0	127.0	127.0	179.0	127.0	40.9	127.0
22.0	22.0	22.0	127.0	40.9	117.0	179.0	127.0	127.0	127.0	179.0	127.0	40.9	127.0
127.0	127.0	127.0	112.5	127.0	127.0	179.0	90.7	90.7	90.7	179.0	76.0	127.0	90.7
179.0	179.0	179.0	179.0	179.0	179.0	8.0	179.0	179.0	179.0	42.0	179.0	179.0	179.0
179.0	179.0	179.0	179.0	179.0	179.0	8.0	179.0	179.0	179.0	42.0	179.0	179.0	179.0
179.0	179.0	179.0	179.0	179.0	179.0	6.4	179.0	179.0	179.0	42.0	179.0	179.0	179.0
179.0	179.0	179.0	179.0	179.0	179.0	6.4	179.0	179.0	179.0	42.0	179.0	179.0	179.0
127.0	127.0	127.0	112.5	127.0	127.0	179.0	98.0	98.0	98.0	179.0	98.0	127.0	98.0
18.9	9.4	9.4	127.0	40.9	117.0	179.0	127.0	127.0	127.0	179.0	127.0	40.9	127.0
90.0	90.0	90.0	127.0	90.0	117.0	179.0	127.0	127.0	127.0	179.0	127.0	90.0	127.0
179.0	179.0	179.0	179.0	179.0	179.0	42.0	179.0	179.0	179.0	21.0	179.0	179.0	179.0
179.0	179.0	179.0	179.0	179.0	179.0	42.0	179.0	179.0	179.0	21.0	179.0	179.0	179.0
0.0	18.9	18.9	127.0	40.9	117.0	179.0	127.0	127.0	127.0	179.0	127.0	40.9	127.0
18.9	0.0	3.1	127.0	40.9	117.0	179.0	127.0	127.0	127.0	179.0	127.0	40.9	127.0
18.9	3.1	0.0	127.0	40.9	117.0	179.0	127.0	127.0	127.0	179.0	127.0	40.9	127.0
127.0	127.0	127.0	0.0	127.0	127.0	179.0	112.5	112.5	112.5	179.0	112.5	127.0	112.5
40.9	40.9	40.9	127.0	0.0	117.0	179.0	127.0	127.0	127.0	179.0	127.0	24.5	127.0
117.0	117.0	117.0	127.0	117.0	0.0	179.0	127.0	127.0	127.0	179.0	127.0	117.0	127.0
179.0	179.0	179.0	179.0	179.0	179.0	0.0	179.0	179.0	179.0	42.0	179.0	179.0	179.0
127.0	127.0	127.0	112.5	127.0	127.0	179.0	0.0	56.0	56.0	179.0	90.7	127.0	44.8
127.0	127.0	127.0	112.5	127.0	127.0	179.0	56.0	0.0	18.7	179.0	90.7	127.0	56.0
127.0	127.0	127.0	112.5	127.0	127.0	179.0	56.0	18.7	0.0	179.0	90.7	127.0	56.0
179.0	179.0	179.0	179.0	179.0	179.0	42.0	179.0	179.0	179.0	0.0	179.0	179.0	179.0
127.0	127.0	127.0	112.5	127.0	127.0	179.0	90.7	90.7	90.7	179.0	0.0	127.0	90.7
40.9	40.9	40.9	127.0	24.5	117.0	179.0	127.0	127.0	127.0	179.0	127.0	0.0	127.0
127.0	127.0	127.0	112.5	127.0	127.0	179.0	44.8	56.0	56.0	179.0	90.7	127.0	0.0
179.0	179.0	179.0	179.0	179.0	179.0	10.7	179.0	179.0	179.0	42.0	179.0	179.0	179.0
122.0	122.0	122.0	127.0	122.0	122.0	179.0	127.0	127.0	127.0	179.0	127.0	122.0	127.0
450.0	450.0	450.0	450.0	450.0	450.0	450.0	450.0	450.0	450.0	450.0	450.0	450.0	450.0
500.0	500.0	500.0	500.0	500.0	500.0	500.0	500.0	500.0	500.0	500.0	500.0	500.0	500.0
117.0	117.0	117.0	127.0	117.0	107.0	179.0	127.0	127.0	127.0	179.0	127.0	117.0	127.0
127.0	127.0	127.0	95.0	127.0	127.0	179.0	112.5	112.5	112.5	179.0	112.5	127.0	112.5
127.0	127.0	127.0	112.5	127.0	127.0	179.0	14.9	56.0	56.0	179.0	90.7	127.0	44.8
147.0	147.0	147.0	147.0	147.0	147.0	179.0	147.0	147.0	147.0	179.0	147.0	147.0	147.0
147.0	147.0	147.0	147.0	147.0	147.0	179.0	147.0	147.0	147.0	179.0	147.0	147.0	147.0
122.0	122.0	122.0	127.0	122.0	122.0	179.0	127.0	127.0	127.0	179.0	127.0	122.0	127.0
179.0	179.0	179.0	179.0	179.0	179.0	8.0	179.0	179.0	179.0	42.0	179.0	179.0	179.0
179.0	179.0	179.0	179.0	179.0	179.0	12.0	179.0	179.0	179.0	42.0	179.0	179.0	179.0
18.9	6.3	6.3	127.0	40.9	117.0	179.0	127.0	127.0	127.0	179.0	127.0	40.9	127.0
325.0	325.0	325.0	325.0	325.0	325.0	325.0	325.0	325.0	325.0	325.0	325.0	325.0	325.0
117.0	117.0	117.0	127.0	117.0	50.4	179.0	127.0	127.0	127.0	179.0	127.0	117.0	127.0
117.0	117.0	117.0	127.0	117.0	50.4	179.0	127.0	127.0	127.0	179.0	127.0	117.0	127.0
117.0	117.0	117.0	127.0	117.0	50.4	179.0	127.0	127.0	127.0	179.0	127.0	117.0	127.0
179.0	179.0	179.0	179.0	179.0	179.0	8.0	179.0	179.0	179.0	42.0	179.0	179.0	179.0
179.0	179.0	179.0	179.0	179.0	179.0	8.0	179.0	179.0	179.0	42.0	179.0	179.0	179.0
179.0	179.0	179.0	179.0	179.0	179.0	8.0	179.0	179.0	179.0	42.0	179.0	179.0	179.0
179.0	179.0	179.0	179.0	179.0	179.0	8.0	179.0	179.0	179.0	42.0	179.0	179.0	179.0
122.0	122.0	122.0	127.0	122.0	122.0	179.0	127.0	127.0	127.0	179.0	127.0	122.0	127.0
122.0	122.0	122.0	127.0	122.0	122.0	179.0	127.0	127.0	127.0	179.0	127.0	122.0	127.0
127.0	127.0	127.0	112.5	127.0	127.0	179.0	98.0	98.0	98.0	179.0	98.0	127.0	98.0
127.0	127.0	127.0	112.5	127.0	127.0	179.0	98.0	98.0	98.0	179.0	98.0	127.0	98.0
127.0	127.0	127.0	112.5	127.0	127.0	179.0	90.7	90.7	90.7	179.0	47.0	127.0	90.7
117.0	117.0	117.0	127.0	117.0	63.0	179.0	127.0	127.0	127.0	179.0	127.0	117.0	127.0
127.0	127.0	127.0	112.5	127.0	127.0	179.0	90.7	90.7	90.7	179.0	31.3	127.0	90.7
127.0	127.0	127.0	112.5	127.0	127.0	179.0	29.9	56.0	56.0	179.0	90.7	127.0	44.8
127.0	127.0	127.0	112.5	127.0	127.0	179.0	90.7	90.7	90.7	179.0	47.0	127.0	90.7
127.0	127.0	127.0	112.5	127.0	127.0	179.0	90.7	90.7	90.7	179.0	47.0	127.0	90.7
122.0	122.0	122.0	127.0	122.0	122.0	179.0	127.0	127.0	127.0	179.0	127.0	122.0	127.0
122.0	122.0	122.0	127.0	122.0	122.0	179.0	127.0	127.0	127.0	179.0	127.0	122.0	127.0
122.0	122.0	122.0	127.0	122.0	122.0	179.0	127.0	127.0	127.0	179.0	127.0	122.0	127.0
122.0	122.0	122.0	127.0	122.0	122.0	179.0	127.0	127.0	127.0	179.0	127.0	122.0	127.0
122.0	122.0	122.0	127.0	122.0	122.0	179.0	127.0	127.0	127.0	179.0	127.0	122.0	127.0
127.0	127.0	127.0	112.5	127.0	127.0	179.0	98.0	98.0	98.0	179.0	98.0	127.0	98.0
127.0	127.0	127.0	112.5	127.0	127.0	179.0	98.0	98.0	98.0	179.0	98.0	127.0	98.0
127.0	127.0	127.0	112.5	127.0	127.0	179.0	98.0	98.0	98.0	179.0	98.0	127.0	98.0
122.0	122.0	122.0	127.0	122.0	122.0	179.0	127.0	127.0	127.0	179.0	127.0	122.0	127.0
122.0	122.0	122.0	127.0	122.0	122.0	179.0	127.0	127.0	127.0	179.0	127.0	122.0	127.0
122.0	122.0	122.0	127.0	122.0	122.0	179.0	127.0	127.0	127.0	179.0	127.0	122.0	127.0
37.7	37.7	37.7	127.0	40.9	117.0	179.0	127.0	127.0	127.0	179.0	127.0	40.9	127.0

37.7	37.7	37.7	127.0	40.9	117.0	179.0	127.0	127.0	127.0	179.0	127.0	40.9	127.0
37.7	37.7	37.7	127.0	40.9	117.0	179.0	127.0	127.0	127.0	179.0	127.0	40.9	127.0
179.0	179.0	179.0	179.0	179.0	179.0	12.0	179.0	179.0	179.0	42.0	179.0	179.0	179.0
179.0	179.0	179.0	179.0	179.0	179.0	12.0	179.0	179.0	179.0	42.0	179.0	179.0	179.0
122.0	122.0	122.0	127.0	122.0	122.0	179.0	127.0	127.0	127.0	179.0	127.0	122.0	127.0
127.0	127.0	127.0	24.0	127.0	127.0	179.0	112.5	112.5	112.5	179.0	112.5	127.0	112.5
127.0	127.0	127.0	24.0	127.0	127.0	179.0	112.5	112.5	112.5	179.0	112.5	127.0	112.5
127.0	127.0	127.0	24.0	127.0	127.0	179.0	112.5	112.5	112.5	179.0	112.5	127.0	112.5
117.0	117.0	117.0	127.0	117.0	107.0	179.0	127.0	127.0	127.0	179.0	127.0	117.0	127.0
28.3	28.3	28.3	127.0	40.9	117.0	179.0	127.0	127.0	127.0	179.0	127.0	40.9	127.0
18.9	15.7	15.7	127.0	40.9	117.0	179.0	127.0	127.0	127.0	179.0	127.0	40.9	127.0
18.9	15.7	15.7	127.0	40.9	117.0	179.0	127.0	127.0	127.0	179.0	127.0	40.9	127.0
122.0	122.0	122.0	127.0	122.0	122.0	179.0	127.0	127.0	127.0	179.0	127.0	122.0	127.0
122.0	122.0	122.0	127.0	122.0	122.0	179.0	127.0	127.0	127.0	179.0	127.0	122.0	127.0
40.9	40.9	40.9	127.0	24.5	117.0	179.0	127.0	127.0	127.0	179.0	127.0	16.3	127.0
18.9	12.6	12.6	127.0	40.9	117.0	179.0	127.0	127.0	127.0	179.0	127.0	40.9	127.0
107.0	107.0	107.0	127.0	107.0	117.0	179.0	127.0	127.0	127.0	179.0	127.0	107.0	127.0
25.1	25.1	25.1	127.0	40.9	117.0	179.0	127.0	127.0	127.0	179.0	127.0	40.9	127.0
127.0	127.0	127.0	112.5	127.0	127.0	179.0	44.8	56.0	56.0	179.0	90.7	127.0	33.6
127.0	127.0	127.0	112.5	127.0	127.0	179.0	44.8	56.0	56.0	179.0	90.7	127.0	33.6
127.0	127.0	127.0	112.5	127.0	127.0	179.0	44.8	56.0	56.0	179.0	90.7	127.0	33.6
127.0	127.0	127.0	112.5	127.0	127.0	179.0	44.8	56.0	56.0	179.0	90.7	127.0	33.6
127.0	127.0	127.0	112.5	127.0	127.0	179.0	44.8	56.0	56.0	179.0	90.7	127.0	33.6
40.9	40.9	40.9	127.0	32.7	117.0	179.0	127.0	127.0	127.0	179.0	127.0	32.7	127.0
37.7	37.7	37.7	127.0	40.9	117.0	179.0	127.0	127.0	127.0	179.0	127.0	40.9	127.0
179.0	179.0	179.0	179.0	179.0	179.0	72.0	179.0	179.0	179.0	72.0	179.0	179.0	179.0
127.0	127.0	127.0	112.5	127.0	127.0	179.0	90.7	90.7	90.7	179.0	76.0	127.0	90.7
117.0	117.0	117.0	127.0	117.0	63.0	179.0	127.0	127.0	127.0	179.0	127.0	117.0	127.0
117.0	117.0	117.0	127.0	117.0	50.4	179.0	127.0	127.0	127.0	179.0	127.0	117.0	127.0
117.0	117.0	117.0	127.0	117.0	50.4	179.0	127.0	127.0	127.0	179.0	127.0	117.0	127.0
127.0	127.0	127.0	112.5	127.0	127.0	179.0	44.8	56.0	56.0	179.0	90.7	127.0	33.6
127.0	127.0	127.0	112.5	127.0	127.0	179.0	44.8	56.0	56.0	179.0	90.7	127.0	33.6
127.0	127.0	127.0	112.5	127.0	127.0	179.0	44.8	56.0	56.0	179.0	90.7	127.0	33.6
127.0	127.0	127.0	112.5	127.0	127.0	179.0	44.8	56.0	56.0	179.0	90.7	127.0	33.6
127.0	127.0	127.0	112.5	127.0	127.0	179.0	98.0	98.0	98.0	179.0	98.0	127.0	98.0
127.0	127.0	127.0	112.5	127.0	127.0	179.0	98.0	98.0	98.0	179.0	98.0	127.0	98.0
127.0	127.0	127.0	112.5	127.0	127.0	179.0	98.0	98.0	98.0	179.0	98.0	127.0	98.0

Melica_nut	Moehringia	Moos_folic	Moos_thal	Myosotis	Oenothera	Ornithopus	Papaver_du	Papaver_rh	Petrorhagia	Phalaris_ar	Phragmites	Picris_hiera	Pinus_sylve
179.0	122.0	450.0	500.0	117.0	127.0	127.0	147.0	147.0	122.0	179.0	179.0	40.9	325.0
10.7	179.0	450.0	500.0	179.0	179.0	179.0	179.0	179.0	179.0	5.0	12.0	179.0	325.0
10.7	179.0	450.0	500.0	179.0	179.0	179.0	179.0	179.0	179.0	5.0	12.0	179.0	325.0
10.7	179.0	450.0	500.0	179.0	179.0	179.0	179.0	179.0	179.0	5.0	12.0	179.0	325.0
179.0	122.0	450.0	500.0	107.0	127.0	127.0	147.0	147.0	122.0	179.0	179.0	117.0	325.0
10.7	179.0	450.0	500.0	179.0	179.0	179.0	179.0	179.0	179.0	6.7	12.0	179.0	325.0
179.0	122.0	450.0	500.0	117.0	127.0	127.0	147.0	147.0	122.0	179.0	179.0	44.0	325.0
179.0	9.7	450.0	500.0	122.0	127.0	127.0	147.0	147.0	32.2	179.0	179.0	122.0	325.0
179.0	122.0	450.0	500.0	117.0	127.0	127.0	147.0	147.0	122.0	179.0	179.0	40.9	325.0
179.0	122.0	450.0	500.0	117.0	127.0	127.0	147.0	147.0	122.0	179.0	179.0	40.9	325.0
179.0	127.0	450.0	500.0	127.0	95.0	112.5	147.0	147.0	127.0	179.0	179.0	127.0	325.0
179.0	127.0	450.0	500.0	127.0	112.5	90.7	147.0	147.0	127.0	179.0	179.0	127.0	325.0
10.7	179.0	450.0	500.0	179.0	179.0	179.0	179.0	179.0	179.0	9.3	12.0	179.0	325.0
10.7	179.0	450.0	500.0	179.0	179.0	179.0	179.0	179.0	179.0	8.0	12.0	179.0	325.0
10.7	179.0	450.0	500.0	179.0	179.0	179.0	179.0	179.0	179.0	8.0	12.0	179.0	325.0
10.7	179.0	450.0	500.0	179.0	179.0	179.0	179.0	179.0	179.0	3.3	12.0	179.0	325.0
42.0	179.0	450.0	500.0	179.0	179.0	179.0	179.0	179.0	179.0	42.0	42.0	179.0	325.0
42.0	179.0	450.0	500.0	179.0	179.0	179.0	179.0	179.0	179.0	42.0	42.0	179.0	325.0
42.0	179.0	450.0	500.0	179.0	179.0	179.0	179.0	179.0	179.0	42.0	42.0	179.0	325.0
42.0	179.0	450.0	500.0	179.0	179.0	179.0	179.0	179.0	179.0	42.0	42.0	179.0	325.0
179.0	122.0	450.0	500.0	117.0	127.0	127.0	147.0	147.0	122.0	179.0	179.0	44.0	325.0
179.0	122.0	450.0	500.0	97.3	127.0	127.0	147.0	147.0	122.0	179.0	179.0	117.0	325.0
179.0	19.3	450.0	500.0	122.0	127.0	127.0	147.0	147.0	32.2	179.0	179.0	122.0	325.0
179.0	19.3	450.0	500.0	122.0	127.0	127.0	147.0	147.0	32.2	179.0	179.0	122.0	325.0
179.0	51.5	450.0	500.0	122.0	127.0	127.0	147.0	147.0	51.5	179.0	179.0	122.0	325.0
179.0	51.5	450.0	500.0	122.0	127.0	127.0	147.0	147.0	51.5	179.0	179.0	122.0	325.0
179.0	122.0	450.0	500.0	117.0	127.0	127.0	147.0	147.0	122.0	179.0	179.0	12.6	325.0
179.0	122.0	450.0	500.0	117.0	127.0	127.0	147.0	147.0	122.0	179.0	179.0	44.0	325.0
179.0	122.0	450.0	500.0	117.0	127.0	127.0	147.0	147.0	122.0	179.0	179.0	44.0	325.0
179.0	122.0	450.0	500.0	97.3	127.0	127.0	147.0	147.0	122.0	179.0	179.0	117.0	325.0
179.0	122.0	450.0	500.0	117.0	127.0	127.0	147.0	147.0	122.0	179.0	179.0	28.3	325.0
10.7	179.0	450.0	500.0	179.0	179.0	179.0	179.0	179.0	179.0	3.3	12.0	179.0	325.0
179.0	127.0	450.0	500.0	127.0	112.5	90.7	147.0	147.0	127.0	179.0	179.0	127.0	325.0
179.0	122.0	450.0	500.0	117.0	127.0	127.0	147.0	147.0	122.0	179.0	179.0	12.6	325.0
179.0	122.0	450.0	500.0	117.0	127.0	127.0	147.0	147.0	122.0	179.0	179.0	12.6	325.0
179.0	122.0	450.0	500.0	117.0	127.0	127.0	147.0	147.0	122.0	179.0	179.0	12.6	325.0
10.7	179.0	450.0	500.0	179.0	179.0	179.0	179.0	179.0	179.0	6.7	12.0	179.0	325.0
12.0	179.0	450.0	500.0	179.0	179.0	179.0	179.0	179.0	179.0	12.0	4.8	179.0	325.0
179.0	122.0	450.0	500.0	117.0	127.0	127.0	147.0	147.0	122.0	179.0	179.0	107.0	325.0
10.7	179.0	450.0	500.0	179.0	179.0	179.0	179.0	179.0	179.0	6.7	12.0	179.0	325.0
12.0	179.0	450.0	500.0	179.0	179.0	179.0	179.0	179.0	179.0	12.0	9.6	179.0	325.0
12.0	179.0	450.0	500.0	179.0	179.0	179.0	179.0	179.0	179.0	12.0	9.6	179.0	325.0
12.0	179.0	450.0	500.0	179.0	179.0	179.0	179.0	179.0	179.0	12.0	9.6	179.0	325.0
179.0	122.0	450.0	500.0	48.7	127.0	127.0	147.0	147.0	122.0	179.0	179.0	117.0	325.0
10.7	179.0	450.0	500.0	179.0	179.0	179.0	179.0	179.0	179.0	8.0	12.0	179.0	325.0
179.0	127.0	450.0	500.0	127.0	20.0	112.5	147.0	147.0	127.0	179.0	179.0	127.0	325.0
179.0	127.0	450.0	500.0	127.0	20.0	112.5	147.0	147.0	127.0	179.0	179.0	127.0	325.0
179.0	127.0	450.0	500.0	127.0	20.0	112.5	147.0	147.0	127.0	179.0	179.0	127.0	325.0
400.0	400.0	450.0	500.0	400.0	400.0	400.0	400.0	400.0	400.0	400.0	400.0	400.0	400.0
179.0	122.0	450.0	500.0	117.0	127.0	127.0	147.0	147.0	122.0	179.0	179.0	28.3	325.0
179.0	127.0	450.0	500.0	127.0	57.5	112.5	147.0	147.0	127.0	179.0	179.0	127.0	325.0
179.0	122.0	450.0	500.0	117.0	127.0	127.0	147.0	147.0	122.0	179.0	179.0	34.6	325.0
10.7	179.0	450.0	500.0	179.0	179.0	179.0	179.0	179.0	179.0	6.7	12.0	179.0	325.0
10.7	179.0	450.0	500.0	179.0	179.0	179.0	179.0	179.0	179.0	6.7	12.0	179.0	325.0
10.7	179.0	450.0	500.0	179.0	179.0	179.0	179.0	179.0	179.0	6.7	12.0	179.0	325.0
179.0	122.0	450.0	500.0	117.0	127.0	127.0	147.0	147.0	122.0	179.0	179.0	31.4	325.0
179.0	122.0	450.0	500.0	117.0	127.0	127.0	147.0	147.0	122.0	179.0	179.0	31.4	325.0
179.0	127.0	450.0	500.0	127.0	112.5	90.7	147.0	147.0	127.0	179.0	179.0	127.0	325.0
179.0	122.0	450.0	500.0	107.0	127.0	127.0	147.0	147.0	122.0	179.0	179.0	117.0	325.0
179.0	127.0	450.0	500.0	127.0	112.5	56.0	147.0	147.0	127.0	179.0	179.0	127.0	325.0
179.0	127.0	450.0	500.0	127.0	112.5	90.7	147.0	147.0	127.0	179.0	179.0	127.0	325.0
179.0	122.0	450.0	500.0	117.0	127.0	127.0	147.0	147.0	122.0	179.0	179.0	31.4	325.0
179.0	122.0	450.0	500.0	117.0	127.0	127.0	147.0	147.0	122.0	179.0	179.0	31.4	325.0

10.7	179.0	450.0	500.0	179.0	179.0	179.0	179.0	179.0	179.0	6.7	12.0	179.0	325.0
179.0	45.1	450.0	500.0	122.0	127.0	127.0	147.0	147.0	45.1	179.0	179.0	122.0	325.0
179.0	122.0	450.0	500.0	117.0	127.0	127.0	147.0	147.0	122.0	179.0	179.0	22.0	325.0
179.0	122.0	450.0	500.0	117.0	127.0	127.0	147.0	147.0	122.0	179.0	179.0	22.0	325.0
179.0	122.0	450.0	500.0	117.0	127.0	127.0	147.0	147.0	122.0	179.0	179.0	22.0	325.0
179.0	122.0	450.0	500.0	117.0	127.0	127.0	147.0	147.0	122.0	179.0	179.0	22.0	325.0
179.0	122.0	450.0	500.0	117.0	127.0	127.0	147.0	147.0	122.0	179.0	179.0	22.0	325.0
179.0	127.0	450.0	500.0	127.0	112.5	90.7	147.0	147.0	127.0	179.0	179.0	127.0	325.0
10.7	179.0	450.0	500.0	179.0	179.0	179.0	179.0	179.0	179.0	6.7	12.0	179.0	325.0
10.7	179.0	450.0	500.0	179.0	179.0	179.0	179.0	179.0	179.0	6.7	12.0	179.0	325.0
10.7	179.0	450.0	500.0	179.0	179.0	179.0	179.0	179.0	179.0	8.0	12.0	179.0	325.0
10.7	179.0	450.0	500.0	179.0	179.0	179.0	179.0	179.0	179.0	8.0	12.0	179.0	325.0
179.0	127.0	450.0	500.0	127.0	112.5	98.0	147.0	147.0	127.0	179.0	179.0	127.0	325.0
179.0	122.0	450.0	500.0	117.0	127.0	127.0	147.0	147.0	122.0	179.0	179.0	9.4	325.0
179.0	122.0	450.0	500.0	117.0	127.0	127.0	147.0	147.0	122.0	179.0	179.0	90.0	325.0
42.0	179.0	450.0	500.0	179.0	179.0	179.0	179.0	179.0	179.0	42.0	42.0	179.0	325.0
42.0	179.0	450.0	500.0	179.0	179.0	179.0	179.0	179.0	179.0	42.0	42.0	179.0	325.0
179.0	122.0	450.0	500.0	117.0	127.0	127.0	147.0	147.0	122.0	179.0	179.0	18.9	325.0
179.0	122.0	450.0	500.0	117.0	127.0	127.0	147.0	147.0	122.0	179.0	179.0	6.3	325.0
179.0	122.0	450.0	500.0	117.0	127.0	127.0	147.0	147.0	122.0	179.0	179.0	6.3	325.0
179.0	127.0	450.0	500.0	127.0	95.0	112.5	147.0	147.0	127.0	179.0	179.0	127.0	325.0
179.0	122.0	450.0	500.0	117.0	127.0	127.0	147.0	147.0	122.0	179.0	179.0	40.9	325.0
179.0	122.0	450.0	500.0	107.0	127.0	127.0	147.0	147.0	122.0	179.0	179.0	117.0	325.0
10.7	179.0	450.0	500.0	179.0	179.0	179.0	179.0	179.0	179.0	8.0	12.0	179.0	325.0
179.0	127.0	450.0	500.0	127.0	112.5	14.9	147.0	147.0	127.0	179.0	179.0	127.0	325.0
179.0	127.0	450.0	500.0	127.0	112.5	56.0	147.0	147.0	127.0	179.0	179.0	127.0	325.0
179.0	127.0	450.0	500.0	127.0	112.5	56.0	147.0	147.0	127.0	179.0	179.0	127.0	325.0
42.0	179.0	450.0	500.0	179.0	179.0	179.0	179.0	179.0	179.0	42.0	42.0	179.0	325.0
179.0	127.0	450.0	500.0	127.0	112.5	90.7	147.0	147.0	127.0	179.0	179.0	127.0	325.0
179.0	122.0	450.0	500.0	117.0	127.0	127.0	147.0	147.0	122.0	179.0	179.0	40.9	325.0
179.0	127.0	450.0	500.0	127.0	112.5	44.8	147.0	147.0	127.0	179.0	179.0	127.0	325.0
0.0	179.0	450.0	500.0	179.0	179.0	179.0	179.0	179.0	179.0	10.7	12.0	179.0	325.0
179.0	0.0	450.0	500.0	122.0	127.0	127.0	147.0	147.0	32.2	179.0	179.0	122.0	325.0
450.0	450.0	0.0	500.0	450.0	450.0	450.0	450.0	450.0	450.0	450.0	450.0	450.0	450.0
500.0	500.0	500.0	0.0	500.0	500.0	500.0	500.0	500.0	500.0	500.0	500.0	500.0	500.0
179.0	122.0	450.0	500.0	0.0	127.0	127.0	147.0	147.0	122.0	179.0	179.0	117.0	325.0
179.0	127.0	450.0	500.0	127.0	0.0	112.5	147.0	147.0	127.0	179.0	179.0	127.0	325.0
179.0	127.0	450.0	500.0	127.0	112.5	0.0	147.0	147.0	127.0	179.0	179.0	127.0	325.0
179.0	147.0	450.0	500.0	147.0	147.0	147.0	0.0	73.5	147.0	179.0	179.0	147.0	325.0
179.0	147.0	450.0	500.0	147.0	147.0	147.0	73.5	0.0	147.0	179.0	179.0	147.0	325.0
179.0	32.2	450.0	500.0	122.0	127.0	127.0	147.0	147.0	0.0	179.0	179.0	122.0	325.0
10.7	179.0	450.0	500.0	179.0	179.0	179.0	179.0	179.0	179.0	0.0	12.0	179.0	325.0
12.0	179.0	450.0	500.0	179.0	179.0	179.0	179.0	179.0	179.0	12.0	0.0	179.0	325.0
179.0	122.0	450.0	500.0	117.0	127.0	127.0	147.0	147.0	122.0	179.0	179.0	0.0	325.0
325.0	325.0	450.0	500.0	325.0	325.0	325.0	325.0	325.0	325.0	325.0	325.0	325.0	0.0
179.0	122.0	450.0	500.0	107.0	127.0	127.0	147.0	147.0	122.0	179.0	179.0	117.0	325.0
179.0	122.0	450.0	500.0	107.0	127.0	127.0	147.0	147.0	122.0	179.0	179.0	117.0	325.0
179.0	122.0	450.0	500.0	107.0	127.0	127.0	147.0	147.0	122.0	179.0	179.0	117.0	325.0
10.7	179.0	450.0	500.0	179.0	179.0	179.0	179.0	179.0	179.0	6.7	12.0	179.0	325.0
10.7	179.0	450.0	500.0	179.0	179.0	179.0	179.0	179.0	179.0	6.7	12.0	179.0	325.0
10.7	179.0	450.0	500.0	179.0	179.0	179.0	179.0	179.0	179.0	6.7	12.0	179.0	325.0
10.7	179.0	450.0	500.0	179.0	179.0	179.0	179.0	179.0	179.0	6.7	12.0	179.0	325.0
179.0	84.0	450.0	500.0	122.0	127.0	127.0	147.0	147.0	84.0	179.0	179.0	122.0	325.0
179.0	84.0	450.0	500.0	122.0	127.0	127.0	147.0	147.0	84.0	179.0	179.0	122.0	325.0
179.0	127.0	450.0	500.0	127.0	112.5	98.0	147.0	147.0	127.0	179.0	179.0	127.0	325.0
179.0	127.0	450.0	500.0	127.0	112.5	98.0	147.0	147.0	127.0	179.0	179.0	127.0	325.0
179.0	127.0	450.0	500.0	127.0	112.5	90.7	147.0	147.0	127.0	179.0	179.0	127.0	325.0
179.0	122.0	450.0	500.0	107.0	127.0	127.0	147.0	147.0	122.0	179.0	179.0	117.0	325.0
179.0	127.0	450.0	500.0	127.0	112.5	90.7	147.0	147.0	127.0	179.0	179.0	127.0	325.0
179.0	127.0	450.0	500.0	127.0	112.5	29.9	147.0	147.0	127.0	179.0	179.0	127.0	325.0
179.0	127.0	450.0	500.0	127.0	112.5	90.7	147.0	147.0	127.0	179.0	179.0	127.0	325.0
179.0	127.0	450.0	500.0	127.0	112.5	90.7	147.0	147.0	127.0	179.0	179.0	127.0	325.0
179.0	84.0	450.0	500.0	122.0	127.0	127.0	147.0	147.0	84.0	179.0	179.0	122.0	325.0
179.0	84.0	450.0	500.0	122.0	127.0	127.0	147.0	147.0	84.0	179.0	179.0	122.0	325.0
179.0	84.0	450.0	500.0	122.0	127.0	127.0	147.0	147.0	84.0	179.0	179.0	122.0	325.0
179.0	84.0	450.0	500.0	122.0	127.0	127.0	147.0	147.0	84.0	179.0	179.0	122.0	325.0
179.0	25.8	450.0	500.0	122.0	127.0	127.0	147.0	147.0	32.2	179.0	179.0	122.0	325.0
179.0	127.0	450.0	500.0	127.0	112.5	98.0	147.0	147.0	127.0	179.0	179.0	127.0	325.0
179.0	127.0	450.0	500.0	127.0	112.5	98.0	147.0	147.0	127.0	179.0	179.0	127.0	325.0
179.0	127.0	450.0	500.0	127.0	112.5	98.0	147.0	147.0	127.0	179.0	179.0	127.0	325.0
179.0	51.5	450.0	500.0	122.0	127.0	127.0	147.0	147.0	51.5	179.0	179.0	122.0	325.0
179.0	25.8	450.0	500.0	122.0	127.0	127.0	147.0	147.0	32.2	179.0	179.0	122.0	325.0
179.0	25.8	450.0	500.0	122.0	127.0	127.0	147.0	147.0	32.2	179.0	179.0	122.0	325.0
179.0	122.0	450.0	500.0	117.0	127.0	127.0	147.0	147.0	122.0	179.0	179.0	37.7	325.0

179.0	122.0	450.0	500.0	117.0	127.0	127.0	147.0	147.0	122.0	179.0	179.0	37.7	325.0
179.0	122.0	450.0	500.0	117.0	127.0	127.0	147.0	147.0	122.0	179.0	179.0	37.7	325.0
12.0	179.0	450.0	500.0	179.0	179.0	179.0	179.0	179.0	179.0	12.0	9.6	179.0	325.0
12.0	179.0	450.0	500.0	179.0	179.0	179.0	179.0	179.0	179.0	12.0	9.6	179.0	325.0
179.0	32.2	450.0	500.0	122.0	127.0	127.0	147.0	147.0	16.1	179.0	179.0	122.0	325.0
179.0	127.0	450.0	500.0	127.0	95.0	112.5	147.0	147.0	127.0	179.0	179.0	127.0	325.0
179.0	127.0	450.0	500.0	127.0	95.0	112.5	147.0	147.0	127.0	179.0	179.0	127.0	325.0
179.0	127.0	450.0	500.0	127.0	95.0	112.5	147.0	147.0	127.0	179.0	179.0	127.0	325.0
179.0	122.0	450.0	500.0	97.3	127.0	127.0	147.0	147.0	122.0	179.0	179.0	117.0	325.0
179.0	122.0	450.0	500.0	117.0	127.0	127.0	147.0	147.0	122.0	179.0	179.0	28.3	325.0
179.0	122.0	450.0	500.0	117.0	127.0	127.0	147.0	147.0	122.0	179.0	179.0	15.7	325.0
179.0	122.0	450.0	500.0	117.0	127.0	127.0	147.0	147.0	122.0	179.0	179.0	15.7	325.0
179.0	38.6	450.0	500.0	122.0	127.0	127.0	147.0	147.0	38.6	179.0	179.0	122.0	325.0
179.0	19.3	450.0	500.0	122.0	127.0	127.0	147.0	147.0	32.2	179.0	179.0	122.0	325.0
179.0	122.0	450.0	500.0	117.0	127.0	127.0	147.0	147.0	122.0	179.0	179.0	40.9	325.0
179.0	122.0	450.0	500.0	117.0	127.0	127.0	147.0	147.0	122.0	179.0	179.0	12.6	325.0
179.0	122.0	450.0	500.0	117.0	127.0	127.0	147.0	147.0	122.0	179.0	179.0	107.0	325.0
179.0	122.0	450.0	500.0	117.0	127.0	127.0	147.0	147.0	122.0	179.0	179.0	25.1	325.0
179.0	127.0	450.0	500.0	127.0	112.5	44.8	147.0	147.0	127.0	179.0	179.0	127.0	325.0
179.0	127.0	450.0	500.0	127.0	112.5	44.8	147.0	147.0	127.0	179.0	179.0	127.0	325.0
179.0	127.0	450.0	500.0	127.0	112.5	44.8	147.0	147.0	127.0	179.0	179.0	127.0	325.0
179.0	127.0	450.0	500.0	127.0	112.5	44.8	147.0	147.0	127.0	179.0	179.0	127.0	325.0
179.0	127.0	450.0	500.0	127.0	112.5	44.8	147.0	147.0	127.0	179.0	179.0	127.0	325.0
179.0	122.0	450.0	500.0	117.0	127.0	127.0	147.0	147.0	122.0	179.0	179.0	40.9	325.0
179.0	122.0	450.0	500.0	117.0	127.0	127.0	147.0	147.0	122.0	179.0	179.0	37.7	325.0
72.0	179.0	450.0	500.0	179.0	179.0	179.0	179.0	179.0	179.0	72.0	72.0	179.0	325.0
179.0	127.0	450.0	500.0	127.0	112.5	90.7	147.0	147.0	127.0	179.0	179.0	127.0	325.0
179.0	122.0	450.0	500.0	107.0	127.0	127.0	147.0	147.0	122.0	179.0	179.0	117.0	325.0
179.0	122.0	450.0	500.0	107.0	127.0	127.0	147.0	147.0	122.0	179.0	179.0	117.0	325.0
179.0	122.0	450.0	500.0	107.0	127.0	127.0	147.0	147.0	122.0	179.0	179.0	117.0	325.0
179.0	127.0	450.0	500.0	127.0	112.5	44.8	147.0	147.0	127.0	179.0	179.0	127.0	325.0
179.0	127.0	450.0	500.0	127.0	112.5	44.8	147.0	147.0	127.0	179.0	179.0	127.0	325.0
179.0	127.0	450.0	500.0	127.0	112.5	44.8	147.0	147.0	127.0	179.0	179.0	127.0	325.0
179.0	127.0	450.0	500.0	127.0	112.5	44.8	147.0	147.0	127.0	179.0	179.0	127.0	325.0
179.0	127.0	450.0	500.0	127.0	112.5	98.0	147.0	147.0	127.0	179.0	179.0	127.0	325.0
179.0	127.0	450.0	500.0	127.0	112.5	98.0	147.0	147.0	127.0	179.0	179.0	127.0	325.0
179.0	127.0	450.0	500.0	127.0	112.5	98.0	147.0	147.0	127.0	179.0	179.0	127.0	325.0

Plantago_l	Plantago_n	Plantago_n	Poa_annua	Poa_comp	Poa_palust	Poa_prater	Polygonum	Polygonum	Populus_tr	Populus_x_	Potentilla_	Prunella_v	Prunus
117.0	117.0	117.0	179.0	179.0	179.0	179.0	122.0	122.0	127.0	127.0	127.0	117.0	127.0
179.0	179.0	179.0	6.7	6.7	6.7	6.7	179.0	179.0	179.0	179.0	179.0	179.0	179.0
179.0	179.0	179.0	6.7	6.7	6.7	6.7	179.0	179.0	179.0	179.0	179.0	179.0	179.0
179.0	179.0	179.0	6.7	6.7	6.7	6.7	179.0	179.0	179.0	179.0	179.0	179.0	179.0
63.0	63.0	63.0	179.0	179.0	179.0	179.0	122.0	122.0	127.0	127.0	127.0	23.0	127.0
179.0	179.0	179.0	3.6	3.6	3.6	3.6	179.0	179.0	179.0	179.0	179.0	179.0	179.0
117.0	117.0	117.0	179.0	179.0	179.0	179.0	122.0	122.0	127.0	127.0	127.0	117.0	127.0
122.0	122.0	122.0	179.0	179.0	179.0	179.0	84.0	84.0	127.0	127.0	127.0	122.0	127.0
117.0	117.0	117.0	179.0	179.0	179.0	179.0	122.0	122.0	127.0	127.0	127.0	117.0	127.0
117.0	117.0	117.0	179.0	179.0	179.0	179.0	122.0	122.0	127.0	127.0	127.0	117.0	127.0
127.0	127.0	127.0	179.0	179.0	179.0	179.0	127.0	127.0	112.5	112.5	112.5	127.0	112.5
127.0	127.0	127.0	179.0	179.0	179.0	179.0	127.0	127.0	98.0	98.0	83.3	127.0	83.3
179.0	179.0	179.0	9.3	9.3	9.3	9.3	179.0	179.0	179.0	179.0	179.0	179.0	179.0
179.0	179.0	179.0	8.0	8.0	8.0	8.0	179.0	179.0	179.0	179.0	179.0	179.0	179.0
179.0	179.0	179.0	8.0	8.0	8.0	8.0	179.0	179.0	179.0	179.0	179.0	179.0	179.0
179.0	179.0	179.0	6.7	6.7	6.7	6.7	179.0	179.0	179.0	179.0	179.0	179.0	179.0
179.0	179.0	179.0	42.0	42.0	42.0	42.0	179.0	179.0	179.0	179.0	179.0	179.0	179.0
179.0	179.0	179.0	42.0	42.0	42.0	42.0	179.0	179.0	179.0	179.0	179.0	179.0	179.0
179.0	179.0	179.0	42.0	42.0	42.0	42.0	179.0	179.0	179.0	179.0	179.0	179.0	179.0
179.0	179.0	179.0	42.0	42.0	42.0	42.0	179.0	179.0	179.0	179.0	179.0	179.0	179.0
117.0	117.0	117.0	179.0	179.0	179.0	179.0	122.0	122.0	127.0	127.0	127.0	117.0	127.0
107.0	107.0	107.0	179.0	179.0	179.0	179.0	122.0	122.0	127.0	127.0	127.0	107.0	127.0
122.0	122.0	122.0	179.0	179.0	179.0	179.0	84.0	84.0	127.0	127.0	127.0	122.0	127.0
122.0	122.0	122.0	179.0	179.0	179.0	179.0	84.0	84.0	127.0	127.0	127.0	122.0	127.0
122.0	122.0	122.0	179.0	179.0	179.0	179.0	84.0	84.0	127.0	127.0	127.0	122.0	127.0
122.0	122.0	122.0	179.0	179.0	179.0	179.0	84.0	84.0	127.0	127.0	127.0	122.0	127.0
117.0	117.0	117.0	179.0	179.0	179.0	179.0	122.0	122.0	127.0	127.0	127.0	117.0	127.0
117.0	117.0	117.0	179.0	179.0	179.0	179.0	122.0	122.0	127.0	127.0	127.0	117.0	127.0
117.0	117.0	117.0	179.0	179.0	179.0	179.0	122.0	122.0	127.0	127.0	127.0	117.0	127.0
107.0	107.0	107.0	179.0	179.0	179.0	179.0	122.0	122.0	127.0	127.0	127.0	107.0	127.0
117.0	117.0	117.0	179.0	179.0	179.0	179.0	122.0	122.0	127.0	127.0	127.0	117.0	127.0
179.0	179.0	179.0	6.7	6.7	6.7	6.7	179.0	179.0	179.0	179.0	179.0	179.0	179.0
127.0	127.0	127.0	179.0	179.0	179.0	179.0	127.0	127.0	98.0	98.0	47.0	127.0	31.3
117.0	117.0	117.0	179.0	179.0	179.0	179.0	122.0	122.0	127.0	127.0	127.0	117.0	127.0
117.0	117.0	117.0	179.0	179.0	179.0	179.0	122.0	122.0	127.0	127.0	127.0	117.0	127.0
117.0	117.0	117.0	179.0	179.0	179.0	179.0	122.0	122.0	127.0	127.0	127.0	117.0	127.0
179.0	179.0	179.0	5.3	5.3	5.3	5.3	179.0	179.0	179.0	179.0	179.0	179.0	179.0
179.0	179.0	179.0	12.0	12.0	12.0	12.0	179.0	179.0	179.0	179.0	179.0	179.0	179.0
117.0	117.0	117.0	179.0	179.0	179.0	179.0	122.0	122.0	127.0	127.0	127.0	117.0	127.0
179.0	179.0	179.0	5.3	5.3	5.3	5.3	179.0	179.0	179.0	179.0	179.0	179.0	179.0
179.0	179.0	179.0	12.0	12.0	12.0	12.0	179.0	179.0	179.0	179.0	179.0	179.0	179.0
179.0	179.0	179.0	12.0	12.0	12.0	12.0	179.0	179.0	179.0	179.0	179.0	179.0	179.0
179.0	179.0	179.0	12.0	12.0	12.0	12.0	179.0	179.0	179.0	179.0	179.0	179.0	179.0
107.0	107.0	107.0	179.0	179.0	179.0	179.0	122.0	122.0	127.0	127.0	127.0	107.0	127.0
179.0	179.0	179.0	8.0	8.0	8.0	8.0	179.0	179.0	179.0	179.0	179.0	179.0	179.0
127.0	127.0	127.0	179.0	179.0	179.0	179.0	127.0	127.0	112.5	112.5	112.5	127.0	112.5
127.0	127.0	127.0	179.0	179.0	179.0	179.0	127.0	127.0	112.5	112.5	112.5	127.0	112.5
127.0	127.0	127.0	179.0	179.0	179.0	179.0	127.0	127.0	112.5	112.5	112.5	127.0	112.5
400.0	400.0	400.0	400.0	400.0	400.0	400.0	400.0	400.0	400.0	400.0	400.0	400.0	400.0
117.0	117.0	117.0	179.0	179.0	179.0	179.0	122.0	122.0	127.0	127.0	127.0	117.0	127.0
127.0	127.0	127.0	179.0	179.0	179.0	179.0	127.0	127.0	112.5	112.5	112.5	127.0	112.5
117.0	117.0	117.0	179.0	179.0	179.0	179.0	122.0	122.0	127.0	127.0	127.0	117.0	127.0
179.0	179.0	179.0	5.3	5.3	5.3	5.3	179.0	179.0	179.0	179.0	179.0	179.0	179.0
179.0	179.0	179.0	5.3	5.3	5.3	5.3	179.0	179.0	179.0	179.0	179.0	179.0	179.0
179.0	179.0	179.0	5.3	5.3	5.3	5.3	179.0	179.0	179.0	179.0	179.0	179.0	179.0
117.0	117.0	117.0	179.0	179.0	179.0	179.0	122.0	122.0	127.0	127.0	127.0	117.0	127.0
117.0	117.0	117.0	179.0	179.0	179.0	179.0	122.0	122.0	127.0	127.0	127.0	117.0	127.0
127.0	127.0	127.0	179.0	179.0	179.0	179.0	127.0	127.0	98.0	98.0	11.8	127.0	47.0
63.0	63.0	63.0	179.0	179.0	179.0	179.0	122.0	122.0	127.0	127.0	127.0	23.0	127.0
127.0	127.0	127.0	179.0	179.0	179.0	179.0	127.0	127.0	98.0	98.0	90.7	127.0	90.7
127.0	127.0	127.0	179.0	179.0	179.0	179.0	127.0	127.0	98.0	98.0	23.5	127.0	47.0
117.0	117.0	117.0	179.0	179.0	179.0	179.0	122.0	122.0	127.0	127.0	127.0	117.0	127.0
117.0	117.0	117.0	179.0	179.0	179.0	179.0	122.0	122.0	127.0	127.0	127.0	117.0	127.0

179.0	179.0	179.0	5.3	5.3	5.3	5.3	179.0	179.0	179.0	179.0	179.0	179.0	179.0
122.0	122.0	122.0	179.0	179.0	179.0	179.0	84.0	84.0	127.0	127.0	127.0	122.0	127.0
117.0	117.0	117.0	179.0	179.0	179.0	179.0	122.0	122.0	127.0	127.0	127.0	117.0	127.0
117.0	117.0	117.0	179.0	179.0	179.0	179.0	122.0	122.0	127.0	127.0	127.0	117.0	127.0
117.0	117.0	117.0	179.0	179.0	179.0	179.0	122.0	122.0	127.0	127.0	127.0	117.0	127.0
117.0	117.0	117.0	179.0	179.0	179.0	179.0	122.0	122.0	127.0	127.0	127.0	117.0	127.0
117.0	117.0	117.0	179.0	179.0	179.0	179.0	122.0	122.0	127.0	127.0	127.0	117.0	127.0
127.0	127.0	127.0	179.0	179.0	179.0	179.0	127.0	127.0	98.0	98.0	76.0	127.0	76.0
179.0	179.0	179.0	5.3	5.3	5.3	5.3	179.0	179.0	179.0	179.0	179.0	179.0	179.0
179.0	179.0	179.0	5.3	5.3	5.3	5.3	179.0	179.0	179.0	179.0	179.0	179.0	179.0
179.0	179.0	179.0	8.0	8.0	8.0	8.0	179.0	179.0	179.0	179.0	179.0	179.0	179.0
179.0	179.0	179.0	8.0	8.0	8.0	8.0	179.0	179.0	179.0	179.0	179.0	179.0	179.0
127.0	127.0	127.0	179.0	179.0	179.0	179.0	127.0	127.0	81.0	81.0	98.0	127.0	98.0
117.0	117.0	117.0	179.0	179.0	179.0	179.0	122.0	122.0	127.0	127.0	127.0	117.0	127.0
117.0	117.0	117.0	179.0	179.0	179.0	179.0	122.0	122.0	127.0	127.0	127.0	117.0	127.0
179.0	179.0	179.0	42.0	42.0	42.0	42.0	179.0	179.0	179.0	179.0	179.0	179.0	179.0
179.0	179.0	179.0	42.0	42.0	42.0	42.0	179.0	179.0	179.0	179.0	179.0	179.0	179.0
117.0	117.0	117.0	179.0	179.0	179.0	179.0	122.0	122.0	127.0	127.0	127.0	117.0	127.0
117.0	117.0	117.0	179.0	179.0	179.0	179.0	122.0	122.0	127.0	127.0	127.0	117.0	127.0
117.0	117.0	117.0	179.0	179.0	179.0	179.0	122.0	122.0	127.0	127.0	127.0	117.0	127.0
127.0	127.0	127.0	179.0	179.0	179.0	179.0	127.0	127.0	112.5	112.5	112.5	127.0	112.5
117.0	117.0	117.0	179.0	179.0	179.0	179.0	122.0	122.0	127.0	127.0	127.0	117.0	127.0
50.4	50.4	50.4	179.0	179.0	179.0	179.0	122.0	122.0	127.0	127.0	127.0	63.0	127.0
179.0	179.0	179.0	8.0	8.0	8.0	8.0	179.0	179.0	179.0	179.0	179.0	179.0	179.0
127.0	127.0	127.0	179.0	179.0	179.0	179.0	127.0	127.0	98.0	98.0	90.7	127.0	90.7
127.0	127.0	127.0	179.0	179.0	179.0	179.0	127.0	127.0	98.0	98.0	90.7	127.0	90.7
127.0	127.0	127.0	179.0	179.0	179.0	179.0	127.0	127.0	98.0	98.0	90.7	127.0	90.7
179.0	179.0	179.0	42.0	42.0	42.0	42.0	179.0	179.0	179.0	179.0	179.0	179.0	179.0
127.0	127.0	127.0	179.0	179.0	179.0	179.0	127.0	127.0	98.0	98.0	47.0	127.0	31.3
117.0	117.0	117.0	179.0	179.0	179.0	179.0	122.0	122.0	127.0	127.0	127.0	117.0	127.0
127.0	127.0	127.0	179.0	179.0	179.0	179.0	127.0	127.0	98.0	98.0	90.7	127.0	90.7
179.0	179.0	179.0	10.7	10.7	10.7	10.7	179.0	179.0	179.0	179.0	179.0	179.0	179.0
122.0	122.0	122.0	179.0	179.0	179.0	179.0	84.0	84.0	127.0	127.0	127.0	122.0	127.0
450.0	450.0	450.0	450.0	450.0	450.0	450.0	450.0	450.0	450.0	450.0	450.0	450.0	450.0
500.0	500.0	500.0	500.0	500.0	500.0	500.0	500.0	500.0	500.0	500.0	500.0	500.0	500.0
107.0	107.0	107.0	179.0	179.0	179.0	179.0	122.0	122.0	127.0	127.0	127.0	107.0	127.0
127.0	127.0	127.0	179.0	179.0	179.0	179.0	127.0	127.0	112.5	112.5	112.5	127.0	112.5
127.0	127.0	127.0	179.0	179.0	179.0	179.0	127.0	127.0	98.0	98.0	90.7	127.0	90.7
147.0	147.0	147.0	179.0	179.0	179.0	179.0	147.0	147.0	147.0	147.0	147.0	147.0	147.0
147.0	147.0	147.0	179.0	179.0	179.0	179.0	147.0	147.0	147.0	147.0	147.0	147.0	147.0
122.0	122.0	122.0	179.0	179.0	179.0	179.0	84.0	84.0	127.0	127.0	127.0	122.0	127.0
179.0	179.0	179.0	6.7	6.7	6.7	6.7	179.0	179.0	179.0	179.0	179.0	179.0	179.0
179.0	179.0	179.0	12.0	12.0	12.0	12.0	179.0	179.0	179.0	179.0	179.0	179.0	179.0
117.0	117.0	117.0	179.0	179.0	179.0	179.0	122.0	122.0	127.0	127.0	127.0	117.0	127.0
325.0	325.0	325.0	325.0	325.0	325.0	325.0	325.0	325.0	325.0	325.0	325.0	325.0	325.0
0.0	25.2	25.2	179.0	179.0	179.0	179.0	122.0	122.0	127.0	127.0	127.0	63.0	127.0
25.2	0.0	12.6	179.0	179.0	179.0	179.0	122.0	122.0	127.0	127.0	127.0	63.0	127.0
25.2	12.6	0.0	179.0	179.0	179.0	179.0	122.0	122.0	127.0	127.0	127.0	63.0	127.0
179.0	179.0	179.0	0.0	1.8	1.8	1.8	179.0	179.0	179.0	179.0	179.0	179.0	179.0
179.0	179.0	179.0	1.8	0.0	1.8	1.8	179.0	179.0	179.0	179.0	179.0	179.0	179.0
179.0	179.0	179.0	1.8	1.8	0.0	1.8	179.0	179.0	179.0	179.0	179.0	179.0	179.0
179.0	179.0	179.0	1.8	1.8	1.8	0.0	179.0	179.0	179.0	179.0	179.0	179.0	179.0
122.0	122.0	122.0	179.0	179.0	179.0	179.0	0.0	14.0	127.0	127.0	127.0	122.0	127.0
122.0	122.0	122.0	179.0	179.0	179.0	179.0	14.0	0.0	127.0	127.0	127.0	122.0	127.0
127.0	127.0	127.0	179.0	179.0	179.0	179.0	127.0	127.0	0.0	10.0	98.0	127.0	98.0
127.0	127.0	127.0	179.0	179.0	179.0	179.0	127.0	127.0	10.0	0.0	98.0	127.0	98.0
127.0	127.0	127.0	179.0	179.0	179.0	179.0	127.0	127.0	98.0	98.0	0.0	127.0	47.0
63.0	63.0	63.0	179.0	179.0	179.0	179.0	122.0	122.0	127.0	127.0	127.0	0.0	127.0
127.0	127.0	127.0	179.0	179.0	179.0	179.0	127.0	127.0	98.0	98.0	47.0	127.0	0.0
127.0	127.0	127.0	179.0	179.0	179.0	179.0	127.0	127.0	98.0	98.0	90.7	127.0	90.7
127.0	127.0	127.0	179.0	179.0	179.0	179.0	127.0	127.0	98.0	98.0	35.3	127.0	47.0
127.0	127.0	127.0	179.0	179.0	179.0	179.0	127.0	127.0	98.0	98.0	35.3	127.0	47.0
122.0	122.0	122.0	179.0	179.0	179.0	179.0	28.0	28.0	127.0	127.0	127.0	122.0	127.0
122.0	122.0	122.0	179.0	179.0	179.0	179.0	28.0	28.0	127.0	127.0	127.0	122.0	127.0
122.0	122.0	122.0	179.0	179.0	179.0	179.0	28.0	28.0	127.0	127.0	127.0	122.0	127.0
122.0	122.0	122.0	179.0	179.0	179.0	179.0	28.0	28.0	127.0	127.0	127.0	122.0	127.0
122.0	122.0	122.0	179.0	179.0	179.0	179.0	84.0	84.0	127.0	127.0	127.0	122.0	127.0
127.0	127.0	127.0	179.0	179.0	179.0	179.0	127.0	127.0	20.0	20.0	98.0	127.0	98.0
127.0	127.0	127.0	179.0	179.0	179.0	179.0	127.0	127.0	20.0	20.0	98.0	127.0	98.0
127.0	127.0	127.0	179.0	179.0	179.0	179.0	127.0	127.0	20.0	20.0	98.0	127.0	98.0
122.0	122.0	122.0	179.0	179.0	179.0	179.0	84.0	84.0	127.0	127.0	127.0	122.0	127.0
122.0	122.0	122.0	179.0	179.0	179.0	179.0	84.0	84.0	127.0	127.0	127.0	122.0	127.0
122.0	122.0	122.0	179.0	179.0	179.0	179.0	84.0	84.0	127.0	127.0	127.0	122.0	127.0
117.0	117.0	117.0	179.0	179.0	179.0	179.0	122.0	122.0	127.0	127.0	127.0	117.0	127.0

117.0	117.0	117.0	179.0	179.0	179.0	179.0	122.0	122.0	127.0	127.0	127.0	117.0	127.0
117.0	117.0	117.0	179.0	179.0	179.0	179.0	122.0	122.0	127.0	127.0	127.0	117.0	127.0
179.0	179.0	179.0	12.0	12.0	12.0	12.0	179.0	179.0	179.0	179.0	179.0	179.0	179.0
179.0	179.0	179.0	12.0	12.0	12.0	12.0	179.0	179.0	179.0	179.0	179.0	179.0	179.0
122.0	122.0	122.0	179.0	179.0	179.0	179.0	84.0	84.0	127.0	127.0	127.0	122.0	127.0
127.0	127.0	127.0	179.0	179.0	179.0	179.0	127.0	127.0	112.5	112.5	112.5	127.0	112.5
127.0	127.0	127.0	179.0	179.0	179.0	179.0	127.0	127.0	112.5	112.5	112.5	127.0	112.5
127.0	127.0	127.0	179.0	179.0	179.0	179.0	127.0	127.0	112.5	112.5	112.5	127.0	112.5
107.0	107.0	107.0	179.0	179.0	179.0	179.0	122.0	122.0	127.0	127.0	127.0	107.0	127.0
117.0	117.0	117.0	179.0	179.0	179.0	179.0	122.0	122.0	127.0	127.0	127.0	117.0	127.0
117.0	117.0	117.0	179.0	179.0	179.0	179.0	122.0	122.0	127.0	127.0	127.0	117.0	127.0
117.0	117.0	117.0	179.0	179.0	179.0	179.0	122.0	122.0	127.0	127.0	127.0	117.0	127.0
122.0	122.0	122.0	179.0	179.0	179.0	179.0	84.0	84.0	127.0	127.0	127.0	122.0	127.0
122.0	122.0	122.0	179.0	179.0	179.0	179.0	84.0	84.0	127.0	127.0	127.0	122.0	127.0
117.0	117.0	117.0	179.0	179.0	179.0	179.0	122.0	122.0	127.0	127.0	127.0	117.0	127.0
117.0	117.0	117.0	179.0	179.0	179.0	179.0	122.0	122.0	127.0	127.0	127.0	117.0	127.0
117.0	117.0	117.0	179.0	179.0	179.0	179.0	122.0	122.0	127.0	127.0	127.0	117.0	127.0
117.0	117.0	117.0	179.0	179.0	179.0	179.0	122.0	122.0	127.0	127.0	127.0	117.0	127.0
127.0	127.0	127.0	179.0	179.0	179.0	179.0	127.0	127.0	98.0	98.0	90.7	127.0	90.7
127.0	127.0	127.0	179.0	179.0	179.0	179.0	127.0	127.0	98.0	98.0	90.7	127.0	90.7
127.0	127.0	127.0	179.0	179.0	179.0	179.0	127.0	127.0	98.0	98.0	90.7	127.0	90.7
127.0	127.0	127.0	179.0	179.0	179.0	179.0	127.0	127.0	98.0	98.0	90.7	127.0	90.7
127.0	127.0	127.0	179.0	179.0	179.0	179.0	127.0	127.0	98.0	98.0	90.7	127.0	90.7
117.0	117.0	117.0	179.0	179.0	179.0	179.0	122.0	122.0	127.0	127.0	127.0	117.0	127.0
117.0	117.0	117.0	179.0	179.0	179.0	179.0	122.0	122.0	127.0	127.0	127.0	117.0	127.0
179.0	179.0	179.0	72.0	72.0	72.0	72.0	179.0	179.0	179.0	179.0	179.0	179.0	179.0
127.0	127.0	127.0	179.0	179.0	179.0	179.0	127.0	127.0	98.0	98.0	76.0	127.0	76.0
63.0	63.0	63.0	179.0	179.0	179.0	179.0	122.0	122.0	127.0	127.0	127.0	43.0	127.0
37.8	37.8	37.8	179.0	179.0	179.0	179.0	122.0	122.0	127.0	127.0	127.0	63.0	127.0
37.8	37.8	37.8	179.0	179.0	179.0	179.0	122.0	122.0	127.0	127.0	127.0	63.0	127.0
127.0	127.0	127.0	179.0	179.0	179.0	179.0	127.0	127.0	98.0	98.0	90.7	127.0	90.7
127.0	127.0	127.0	179.0	179.0	179.0	179.0	127.0	127.0	98.0	98.0	90.7	127.0	90.7
127.0	127.0	127.0	179.0	179.0	179.0	179.0	127.0	127.0	98.0	98.0	90.7	127.0	90.7
127.0	127.0	127.0	179.0	179.0	179.0	179.0	127.0	127.0	98.0	98.0	90.7	127.0	90.7
127.0	127.0	127.0	179.0	179.0	179.0	179.0	127.0	127.0	81.0	81.0	98.0	127.0	98.0
127.0	127.0	127.0	179.0	179.0	179.0	179.0	127.0	127.0	81.0	81.0	98.0	127.0	98.0
127.0	127.0	127.0	179.0	179.0	179.0	179.0	127.0	127.0	81.0	81.0	98.0	127.0	98.0

Robinia_ps	Rubus_frut	Rubus_idae	Rumex_ace	Rumex_ace	Rumex_cris	Rumex_thy	Sagina_pro	Salix_capre	Salix_vimin	Salix_x_rub	Salsola_kal	Scleranthus	Scleranthus
127.0	127.0	127.0	122.0	122.0	122.0	122.0	122.0	127.0	127.0	127.0	122.0	122.0	122.0
179.0	179.0	179.0	179.0	179.0	179.0	179.0	179.0	179.0	179.0	179.0	179.0	179.0	179.0
179.0	179.0	179.0	179.0	179.0	179.0	179.0	179.0	179.0	179.0	179.0	179.0	179.0	179.0
179.0	179.0	179.0	179.0	179.0	179.0	179.0	179.0	179.0	179.0	179.0	179.0	179.0	179.0
127.0	127.0	127.0	122.0	122.0	122.0	122.0	122.0	127.0	127.0	127.0	122.0	122.0	122.0
179.0	179.0	179.0	179.0	179.0	179.0	179.0	179.0	179.0	179.0	179.0	179.0	179.0	179.0
127.0	127.0	127.0	122.0	122.0	122.0	122.0	122.0	127.0	127.0	127.0	122.0	122.0	122.0
127.0	127.0	127.0	84.0	84.0	84.0	84.0	25.8	127.0	127.0	127.0	51.5	25.8	25.8
127.0	127.0	127.0	122.0	122.0	122.0	122.0	122.0	127.0	127.0	127.0	122.0	122.0	122.0
127.0	127.0	127.0	122.0	122.0	122.0	122.0	122.0	127.0	127.0	127.0	122.0	122.0	122.0
112.5	112.5	112.5	127.0	127.0	127.0	127.0	127.0	112.5	112.5	112.5	127.0	127.0	127.0
90.7	83.3	83.3	127.0	127.0	127.0	127.0	127.0	98.0	98.0	98.0	127.0	127.0	127.0
179.0	179.0	179.0	179.0	179.0	179.0	179.0	179.0	179.0	179.0	179.0	179.0	179.0	179.0
179.0	179.0	179.0	179.0	179.0	179.0	179.0	179.0	179.0	179.0	179.0	179.0	179.0	179.0
179.0	179.0	179.0	179.0	179.0	179.0	179.0	179.0	179.0	179.0	179.0	179.0	179.0	179.0
179.0	179.0	179.0	179.0	179.0	179.0	179.0	179.0	179.0	179.0	179.0	179.0	179.0	179.0
179.0	179.0	179.0	179.0	179.0	179.0	179.0	179.0	179.0	179.0	179.0	179.0	179.0	179.0
179.0	179.0	179.0	179.0	179.0	179.0	179.0	179.0	179.0	179.0	179.0	179.0	179.0	179.0
179.0	179.0	179.0	179.0	179.0	179.0	179.0	179.0	179.0	179.0	179.0	179.0	179.0	179.0
179.0	179.0	179.0	179.0	179.0	179.0	179.0	179.0	179.0	179.0	179.0	179.0	179.0	179.0
127.0	127.0	127.0	122.0	122.0	122.0	122.0	122.0	127.0	127.0	127.0	122.0	122.0	122.0
127.0	127.0	127.0	122.0	122.0	122.0	122.0	122.0	127.0	127.0	127.0	122.0	122.0	122.0
127.0	127.0	127.0	84.0	84.0	84.0	84.0	25.8	127.0	127.0	127.0	51.5	25.8	25.8
127.0	127.0	127.0	84.0	84.0	84.0	84.0	25.8	127.0	127.0	127.0	51.5	25.8	25.8
127.0	127.0	127.0	84.0	84.0	84.0	84.0	51.5	127.0	127.0	127.0	19.0	51.5	51.5
127.0	127.0	127.0	84.0	84.0	84.0	84.0	51.5	127.0	127.0	127.0	19.0	51.5	51.5
127.0	127.0	127.0	122.0	122.0	122.0	122.0	122.0	127.0	127.0	127.0	122.0	122.0	122.0
127.0	127.0	127.0	122.0	122.0	122.0	122.0	122.0	127.0	127.0	127.0	122.0	122.0	122.0
127.0	127.0	127.0	122.0	122.0	122.0	122.0	122.0	127.0	127.0	127.0	122.0	122.0	122.0
127.0	127.0	127.0	122.0	122.0	122.0	122.0	122.0	127.0	127.0	127.0	122.0	122.0	122.0
127.0	127.0	127.0	122.0	122.0	122.0	122.0	122.0	127.0	127.0	127.0	122.0	122.0	122.0
179.0	179.0	179.0	179.0	179.0	179.0	179.0	179.0	179.0	179.0	179.0	179.0	179.0	179.0
90.7	47.0	47.0	127.0	127.0	127.0	127.0	127.0	98.0	98.0	98.0	127.0	127.0	127.0
127.0	127.0	127.0	122.0	122.0	122.0	122.0	122.0	127.0	127.0	127.0	122.0	122.0	122.0
127.0	127.0	127.0	122.0	122.0	122.0	122.0	122.0	127.0	127.0	127.0	122.0	122.0	122.0
127.0	127.0	127.0	122.0	122.0	122.0	122.0	122.0	127.0	127.0	127.0	122.0	122.0	122.0
179.0	179.0	179.0	179.0	179.0	179.0	179.0	179.0	179.0	179.0	179.0	179.0	179.0	179.0
179.0	179.0	179.0	179.0	179.0	179.0	179.0	179.0	179.0	179.0	179.0	179.0	179.0	179.0
127.0	127.0	127.0	122.0	122.0	122.0	122.0	122.0	127.0	127.0	127.0	122.0	122.0	122.0
179.0	179.0	179.0	179.0	179.0	179.0	179.0	179.0	179.0	179.0	179.0	179.0	179.0	179.0
179.0	179.0	179.0	179.0	179.0	179.0	179.0	179.0	179.0	179.0	179.0	179.0	179.0	179.0
179.0	179.0	179.0	179.0	179.0	179.0	179.0	179.0	179.0	179.0	179.0	179.0	179.0	179.0
179.0	179.0	179.0	179.0	179.0	179.0	179.0	179.0	179.0	179.0	179.0	179.0	179.0	179.0
127.0	127.0	127.0	122.0	122.0	122.0	122.0	122.0	127.0	127.0	127.0	122.0	122.0	122.0
179.0	179.0	179.0	179.0	179.0	179.0	179.0	179.0	179.0	179.0	179.0	179.0	179.0	179.0
112.5	112.5	112.5	127.0	127.0	127.0	127.0	127.0	112.5	112.5	112.5	127.0	127.0	127.0
112.5	112.5	112.5	127.0	127.0	127.0	127.0	127.0	112.5	112.5	112.5	127.0	127.0	127.0
112.5	112.5	112.5	127.0	127.0	127.0	127.0	127.0	112.5	112.5	112.5	127.0	127.0	127.0
400.0	400.0	400.0	400.0	400.0	400.0	400.0	400.0	400.0	400.0	400.0	400.0	400.0	400.0
127.0	127.0	127.0	122.0	122.0	122.0	122.0	122.0	127.0	127.0	127.0	122.0	122.0	122.0
112.5	112.5	112.5	127.0	127.0	127.0	127.0	127.0	112.5	112.5	112.5	127.0	127.0	127.0
127.0	127.0	127.0	122.0	122.0	122.0	122.0	122.0	127.0	127.0	127.0	122.0	122.0	122.0
179.0	179.0	179.0	179.0	179.0	179.0	179.0	179.0	179.0	179.0	179.0	179.0	179.0	179.0
179.0	179.0	179.0	179.0	179.0	179.0	179.0	179.0	179.0	179.0	179.0	179.0	179.0	179.0
179.0	179.0	179.0	179.0	179.0	179.0	179.0	179.0	179.0	179.0	179.0	179.0	179.0	179.0
127.0	127.0	127.0	122.0	122.0	122.0	122.0	122.0	127.0	127.0	127.0	122.0	122.0	122.0
127.0	127.0	127.0	122.0	122.0	122.0	122.0	122.0	127.0	127.0	127.0	122.0	122.0	122.0
90.7	35.3	35.3	127.0	127.0	127.0	127.0	127.0	98.0	98.0	98.0	127.0	127.0	127.0
127.0	127.0	127.0	122.0	122.0	122.0	122.0	122.0	127.0	127.0	127.0	122.0	122.0	122.0
56.0	90.7	90.7	127.0	127.0	127.0	127.0	127.0	98.0	98.0	98.0	127.0	127.0	127.0
90.7	35.3	35.3	127.0	127.0	127.0	127.0	127.0	98.0	98.0	98.0	127.0	127.0	127.0
127.0	127.0	127.0	122.0	122.0	122.0	122.0	122.0	127.0	127.0	127.0	122.0	122.0	122.0
127.0	127.0	127.0	122.0	122.0	122.0	122.0	122.0	127.0	127.0	127.0	122.0	122.0	122.0

179.0	179.0	179.0	179.0	179.0	179.0	179.0	179.0	179.0	179.0	179.0	179.0	179.0	179.0
127.0	127.0	127.0	84.0	84.0	84.0	84.0	45.1	127.0	127.0	127.0	51.5	45.1	45.1
127.0	127.0	127.0	122.0	122.0	122.0	122.0	122.0	127.0	127.0	127.0	122.0	122.0	122.0
127.0	127.0	127.0	122.0	122.0	122.0	122.0	122.0	127.0	127.0	127.0	122.0	122.0	122.0
127.0	127.0	127.0	122.0	122.0	122.0	122.0	122.0	127.0	127.0	127.0	122.0	122.0	122.0
127.0	127.0	127.0	122.0	122.0	122.0	122.0	122.0	127.0	127.0	127.0	122.0	122.0	122.0
127.0	127.0	127.0	122.0	122.0	122.0	122.0	122.0	127.0	127.0	127.0	122.0	122.0	122.0
90.7	76.0	76.0	127.0	127.0	127.0	127.0	127.0	98.0	98.0	98.0	127.0	127.0	127.0
179.0	179.0	179.0	179.0	179.0	179.0	179.0	179.0	179.0	179.0	179.0	179.0	179.0	179.0
179.0	179.0	179.0	179.0	179.0	179.0	179.0	179.0	179.0	179.0	179.0	179.0	179.0	179.0
179.0	179.0	179.0	179.0	179.0	179.0	179.0	179.0	179.0	179.0	179.0	179.0	179.0	179.0
179.0	179.0	179.0	179.0	179.0	179.0	179.0	179.0	179.0	179.0	179.0	179.0	179.0	179.0
98.0	98.0	98.0	127.0	127.0	127.0	127.0	127.0	81.0	81.0	81.0	127.0	127.0	127.0
127.0	127.0	127.0	122.0	122.0	122.0	122.0	122.0	127.0	127.0	127.0	122.0	122.0	122.0
127.0	127.0	127.0	122.0	122.0	122.0	122.0	122.0	127.0	127.0	127.0	122.0	122.0	122.0
179.0	179.0	179.0	179.0	179.0	179.0	179.0	179.0	179.0	179.0	179.0	179.0	179.0	179.0
179.0	179.0	179.0	179.0	179.0	179.0	179.0	179.0	179.0	179.0	179.0	179.0	179.0	179.0
127.0	127.0	127.0	122.0	122.0	122.0	122.0	122.0	127.0	127.0	127.0	122.0	122.0	122.0
127.0	127.0	127.0	122.0	122.0	122.0	122.0	122.0	127.0	127.0	127.0	122.0	122.0	122.0
127.0	127.0	127.0	122.0	122.0	122.0	122.0	122.0	127.0	127.0	127.0	122.0	122.0	122.0
112.5	112.5	112.5	127.0	127.0	127.0	127.0	127.0	112.5	112.5	112.5	127.0	127.0	127.0
127.0	127.0	127.0	122.0	122.0	122.0	122.0	122.0	127.0	127.0	127.0	122.0	122.0	122.0
127.0	127.0	127.0	122.0	122.0	122.0	122.0	122.0	127.0	127.0	127.0	122.0	122.0	122.0
179.0	179.0	179.0	179.0	179.0	179.0	179.0	179.0	179.0	179.0	179.0	179.0	179.0	179.0
29.9	90.7	90.7	127.0	127.0	127.0	127.0	127.0	98.0	98.0	98.0	127.0	127.0	127.0
56.0	90.7	90.7	127.0	127.0	127.0	127.0	127.0	98.0	98.0	98.0	127.0	127.0	127.0
56.0	90.7	90.7	127.0	127.0	127.0	127.0	127.0	98.0	98.0	98.0	127.0	127.0	127.0
179.0	179.0	179.0	179.0	179.0	179.0	179.0	179.0	179.0	179.0	179.0	179.0	179.0	179.0
90.7	47.0	47.0	127.0	127.0	127.0	127.0	127.0	98.0	98.0	98.0	127.0	127.0	127.0
127.0	127.0	127.0	122.0	122.0	122.0	122.0	122.0	127.0	127.0	127.0	122.0	122.0	122.0
44.8	90.7	90.7	127.0	127.0	127.0	127.0	127.0	98.0	98.0	98.0	127.0	127.0	127.0
179.0	179.0	179.0	179.0	179.0	179.0	179.0	179.0	179.0	179.0	179.0	179.0	179.0	179.0
127.0	127.0	127.0	84.0	84.0	84.0	84.0	25.8	127.0	127.0	127.0	51.5	25.8	25.8
450.0	450.0	450.0	450.0	450.0	450.0	450.0	450.0	450.0	450.0	450.0	450.0	450.0	450.0
500.0	500.0	500.0	500.0	500.0	500.0	500.0	500.0	500.0	500.0	500.0	500.0	500.0	500.0
127.0	127.0	127.0	122.0	122.0	122.0	122.0	122.0	127.0	127.0	127.0	122.0	122.0	122.0
112.5	112.5	112.5	127.0	127.0	127.0	127.0	127.0	112.5	112.5	112.5	127.0	127.0	127.0
29.9	90.7	90.7	127.0	127.0	127.0	127.0	127.0	98.0	98.0	98.0	127.0	127.0	127.0
147.0	147.0	147.0	147.0	147.0	147.0	147.0	147.0	147.0	147.0	147.0	147.0	147.0	147.0
147.0	147.0	147.0	147.0	147.0	147.0	147.0	147.0	147.0	147.0	147.0	147.0	147.0	147.0
127.0	127.0	127.0	84.0	84.0	84.0	84.0	32.2	127.0	127.0	127.0	51.5	32.2	32.2
179.0	179.0	179.0	179.0	179.0	179.0	179.0	179.0	179.0	179.0	179.0	179.0	179.0	179.0
179.0	179.0	179.0	179.0	179.0	179.0	179.0	179.0	179.0	179.0	179.0	179.0	179.0	179.0
127.0	127.0	127.0	122.0	122.0	122.0	122.0	122.0	127.0	127.0	127.0	122.0	122.0	122.0
325.0	325.0	325.0	325.0	325.0	325.0	325.0	325.0	325.0	325.0	325.0	325.0	325.0	325.0
127.0	127.0	127.0	122.0	122.0	122.0	122.0	122.0	127.0	127.0	127.0	122.0	122.0	122.0
127.0	127.0	127.0	122.0	122.0	122.0	122.0	122.0	127.0	127.0	127.0	122.0	122.0	122.0
127.0	127.0	127.0	122.0	122.0	122.0	122.0	122.0	127.0	127.0	127.0	122.0	122.0	122.0
179.0	179.0	179.0	179.0	179.0	179.0	179.0	179.0	179.0	179.0	179.0	179.0	179.0	179.0
179.0	179.0	179.0	179.0	179.0	179.0	179.0	179.0	179.0	179.0	179.0	179.0	179.0	179.0
179.0	179.0	179.0	179.0	179.0	179.0	179.0	179.0	179.0	179.0	179.0	179.0	179.0	179.0
179.0	179.0	179.0	179.0	179.0	179.0	179.0	179.0	179.0	179.0	179.0	179.0	179.0	179.0
127.0	127.0	127.0	28.0	28.0	28.0	28.0	84.0	127.0	127.0	127.0	84.0	84.0	84.0
127.0	127.0	127.0	28.0	28.0	28.0	28.0	84.0	127.0	127.0	127.0	84.0	84.0	84.0
98.0	98.0	98.0	127.0	127.0	127.0	127.0	127.0	20.0	20.0	20.0	127.0	127.0	127.0
98.0	98.0	98.0	127.0	127.0	127.0	127.0	127.0	20.0	20.0	20.0	127.0	127.0	127.0
90.7	35.3	35.3	127.0	127.0	127.0	127.0	127.0	98.0	98.0	98.0	127.0	127.0	127.0
127.0	127.0	127.0	122.0	122.0	122.0	122.0	122.0	127.0	127.0	127.0	122.0	122.0	122.0
90.7	47.0	47.0	127.0	127.0	127.0	127.0	127.0	98.0	98.0	98.0	127.0	127.0	127.0
0.0	90.7	90.7	127.0	127.0	127.0	127.0	127.0	98.0	98.0	98.0	127.0	127.0	127.0
90.7	0.0	17.6	127.0	127.0	127.0	127.0	127.0	98.0	98.0	98.0	127.0	127.0	127.0
90.7	17.6	0.0	127.0	127.0	127.0	127.0	127.0	98.0	98.0	98.0	127.0	127.0	127.0
127.0	127.0	127.0	0.0	14.0	14.0	14.0	84.0	127.0	127.0	127.0	84.0	84.0	84.0
127.0	127.0	127.0	14.0	0.0	14.0	14.0	84.0	127.0	127.0	127.0	84.0	84.0	84.0
127.0	127.0	127.0	14.0	14.0	0.0	14.0	84.0	127.0	127.0	127.0	84.0	84.0	84.0
127.0	127.0	127.0	14.0	14.0	14.0	0.0	84.0	127.0	127.0	127.0	84.0	84.0	84.0
127.0	127.0	127.0	84.0	84.0	84.0	84.0	0.0	127.0	127.0	127.0	51.5	17.2	17.2
98.0	98.0	98.0	127.0	127.0	127.0	127.0	127.0	0.0	10.0	10.0	127.0	127.0	127.0
98.0	98.0	98.0	127.0	127.0	127.0	127.0	127.0	10.0	0.0	10.0	127.0	127.0	127.0
98.0	98.0	98.0	127.0	127.0	127.0	127.0	127.0	10.0	10.0	0.0	127.0	127.0	127.0
127.0	127.0	127.0	84.0	84.0	84.0	84.0	51.5	127.0	127.0	127.0	0.0	51.5	51.5
127.0	127.0	127.0	84.0	84.0	84.0	84.0	17.2	127.0	127.0	127.0	51.5	0.0	8.6
127.0	127.0	127.0	84.0	84.0	84.0	84.0	17.2	127.0	127.0	127.0	51.5	8.6	0.0
127.0	127.0	127.0	122.0	122.0	122.0	122.0	122.0	127.0	127.0	127.0	122.0	122.0	122.0

127.0	127.0	127.0	122.0	122.0	122.0	122.0	122.0	127.0	127.0	127.0	122.0	122.0	122.0
127.0	127.0	127.0	122.0	122.0	122.0	122.0	122.0	127.0	127.0	127.0	122.0	122.0	122.0
179.0	179.0	179.0	179.0	179.0	179.0	179.0	179.0	179.0	179.0	179.0	179.0	179.0	179.0
179.0	179.0	179.0	179.0	179.0	179.0	179.0	179.0	179.0	179.0	179.0	179.0	179.0	179.0
127.0	127.0	127.0	84.0	84.0	84.0	84.0	32.2	127.0	127.0	127.0	51.5	32.2	32.2
112.5	112.5	112.5	127.0	127.0	127.0	127.0	127.0	112.5	112.5	112.5	127.0	127.0	127.0
112.5	112.5	112.5	127.0	127.0	127.0	127.0	127.0	112.5	112.5	112.5	127.0	127.0	127.0
112.5	112.5	112.5	127.0	127.0	127.0	127.0	127.0	112.5	112.5	112.5	127.0	127.0	127.0
127.0	127.0	127.0	122.0	122.0	122.0	122.0	122.0	127.0	127.0	127.0	122.0	122.0	122.0
127.0	127.0	127.0	122.0	122.0	122.0	122.0	122.0	127.0	127.0	127.0	122.0	122.0	122.0
127.0	127.0	127.0	122.0	122.0	122.0	122.0	122.0	127.0	127.0	127.0	122.0	122.0	122.0
127.0	127.0	127.0	122.0	122.0	122.0	122.0	122.0	127.0	127.0	127.0	122.0	122.0	122.0
127.0	127.0	127.0	84.0	84.0	84.0	84.0	38.6	127.0	127.0	127.0	51.5	38.6	38.6
127.0	127.0	127.0	84.0	84.0	84.0	84.0	25.8	127.0	127.0	127.0	51.5	25.8	25.8
127.0	127.0	127.0	122.0	122.0	122.0	122.0	122.0	127.0	127.0	127.0	122.0	122.0	122.0
127.0	127.0	127.0	122.0	122.0	122.0	122.0	122.0	127.0	127.0	127.0	122.0	122.0	122.0
127.0	127.0	127.0	122.0	122.0	122.0	122.0	122.0	127.0	127.0	127.0	122.0	122.0	122.0
127.0	127.0	127.0	122.0	122.0	122.0	122.0	122.0	127.0	127.0	127.0	122.0	122.0	122.0
44.8	90.7	90.7	127.0	127.0	127.0	127.0	127.0	98.0	98.0	98.0	127.0	127.0	127.0
44.8	90.7	90.7	127.0	127.0	127.0	127.0	127.0	98.0	98.0	98.0	127.0	127.0	127.0
44.8	90.7	90.7	127.0	127.0	127.0	127.0	127.0	98.0	98.0	98.0	127.0	127.0	127.0
44.8	90.7	90.7	127.0	127.0	127.0	127.0	127.0	98.0	98.0	98.0	127.0	127.0	127.0
44.8	90.7	90.7	127.0	127.0	127.0	127.0	127.0	98.0	98.0	98.0	127.0	127.0	127.0
127.0	127.0	127.0	122.0	122.0	122.0	122.0	122.0	127.0	127.0	127.0	122.0	122.0	122.0
127.0	127.0	127.0	122.0	122.0	122.0	122.0	122.0	127.0	127.0	127.0	122.0	122.0	122.0
179.0	179.0	179.0	179.0	179.0	179.0	179.0	179.0	179.0	179.0	179.0	179.0	179.0	179.0
90.7	76.0	76.0	127.0	127.0	127.0	127.0	127.0	98.0	98.0	98.0	127.0	127.0	127.0
127.0	127.0	127.0	122.0	122.0	122.0	122.0	122.0	127.0	127.0	127.0	122.0	122.0	122.0
127.0	127.0	127.0	122.0	122.0	122.0	122.0	122.0	127.0	127.0	127.0	122.0	122.0	122.0
127.0	127.0	127.0	122.0	122.0	122.0	122.0	122.0	127.0	127.0	127.0	122.0	122.0	122.0
44.8	90.7	90.7	127.0	127.0	127.0	127.0	127.0	98.0	98.0	98.0	127.0	127.0	127.0
44.8	90.7	90.7	127.0	127.0	127.0	127.0	127.0	98.0	98.0	98.0	127.0	127.0	127.0
44.8	90.7	90.7	127.0	127.0	127.0	127.0	127.0	98.0	98.0	98.0	127.0	127.0	127.0
44.8	90.7	90.7	127.0	127.0	127.0	127.0	127.0	98.0	98.0	98.0	127.0	127.0	127.0
98.0	98.0	98.0	127.0	127.0	127.0	127.0	127.0	81.0	81.0	81.0	127.0	127.0	127.0
98.0	98.0	98.0	127.0	127.0	127.0	127.0	127.0	81.0	81.0	81.0	127.0	127.0	127.0
98.0	98.0	98.0	127.0	127.0	127.0	127.0	127.0	81.0	81.0	81.0	127.0	127.0	127.0

Senecio_ve	Senecio_vi	Senecio_vu	Setaria_pu	Setaria_viri	Silene_alba	Sinapis_arv	Sisymbrium	Sisymbrium	Solanum_n	Solidago_c	Sonchus_ar	Sonchus_as	Spergularia
40.9	40.9	40.9	179.0	179.0	122.0	127.0	127.0	127.0	117.0	40.9	40.9	40.9	122.0
179.0	179.0	179.0	12.0	12.0	179.0	179.0	179.0	179.0	179.0	179.0	179.0	179.0	179.0
179.0	179.0	179.0	12.0	12.0	179.0	179.0	179.0	179.0	179.0	179.0	179.0	179.0	179.0
179.0	179.0	179.0	12.0	12.0	179.0	179.0	179.0	179.0	179.0	179.0	179.0	179.0	179.0
117.0	117.0	117.0	179.0	179.0	122.0	127.0	127.0	127.0	107.0	117.0	117.0	117.0	122.0
179.0	179.0	179.0	12.0	12.0	179.0	179.0	179.0	179.0	179.0	179.0	179.0	179.0	179.0
44.0	44.0	44.0	179.0	179.0	122.0	127.0	127.0	127.0	117.0	44.0	44.0	44.0	122.0
122.0	122.0	122.0	179.0	179.0	32.2	127.0	127.0	127.0	122.0	122.0	122.0	122.0	38.6
40.9	40.9	40.9	179.0	179.0	122.0	127.0	127.0	127.0	117.0	40.9	40.9	40.9	122.0
40.9	40.9	40.9	179.0	179.0	122.0	127.0	127.0	127.0	117.0	40.9	40.9	40.9	122.0
127.0	127.0	127.0	179.0	179.0	127.0	18.0	12.0	12.0	127.0	127.0	127.0	127.0	127.0
127.0	127.0	127.0	179.0	179.0	127.0	112.5	112.5	112.5	127.0	127.0	127.0	127.0	127.0
179.0	179.0	179.0	12.0	12.0	179.0	179.0	179.0	179.0	179.0	179.0	179.0	179.0	179.0
179.0	179.0	179.0	12.0	12.0	179.0	179.0	179.0	179.0	179.0	179.0	179.0	179.0	179.0
179.0	179.0	179.0	12.0	12.0	179.0	179.0	179.0	179.0	179.0	179.0	179.0	179.0	179.0
179.0	179.0	179.0	12.0	12.0	179.0	179.0	179.0	179.0	179.0	179.0	179.0	179.0	179.0
179.0	179.0	179.0	42.0	42.0	179.0	179.0	179.0	179.0	179.0	179.0	179.0	179.0	179.0
179.0	179.0	179.0	42.0	42.0	179.0	179.0	179.0	179.0	179.0	179.0	179.0	179.0	179.0
179.0	179.0	179.0	42.0	42.0	179.0	179.0	179.0	179.0	179.0	179.0	179.0	179.0	179.0
179.0	179.0	179.0	42.0	42.0	179.0	179.0	179.0	179.0	179.0	179.0	179.0	179.0	179.0
44.0	44.0	44.0	179.0	179.0	122.0	127.0	127.0	127.0	117.0	44.0	44.0	44.0	122.0
117.0	117.0	117.0	179.0	179.0	122.0	127.0	127.0	127.0	87.7	117.0	117.0	117.0	122.0
122.0	122.0	122.0	179.0	179.0	32.2	127.0	127.0	127.0	122.0	122.0	122.0	122.0	38.6
122.0	122.0	122.0	179.0	179.0	32.2	127.0	127.0	127.0	122.0	122.0	122.0	122.0	38.6
122.0	122.0	122.0	179.0	179.0	51.5	127.0	127.0	127.0	122.0	122.0	122.0	122.0	51.5
122.0	122.0	122.0	179.0	179.0	51.5	127.0	127.0	127.0	122.0	122.0	122.0	122.0	51.5
37.7	37.7	37.7	179.0	179.0	122.0	127.0	127.0	127.0	117.0	28.3	15.7	15.7	122.0
44.0	44.0	44.0	179.0	179.0	122.0	127.0	127.0	127.0	117.0	44.0	44.0	44.0	122.0
44.0	44.0	44.0	179.0	179.0	122.0	127.0	127.0	127.0	117.0	44.0	44.0	44.0	122.0
117.0	117.0	117.0	179.0	179.0	122.0	127.0	127.0	127.0	78.0	117.0	117.0	117.0	122.0
37.7	37.7	37.7	179.0	179.0	122.0	127.0	127.0	127.0	117.0	18.9	28.3	28.3	122.0
179.0	179.0	179.0	12.0	12.0	179.0	179.0	179.0	179.0	179.0	179.0	179.0	179.0	179.0
127.0	127.0	127.0	179.0	179.0	127.0	112.5	112.5	112.5	127.0	127.0	127.0	127.0	127.0
37.7	37.7	37.7	179.0	179.0	122.0	127.0	127.0	127.0	117.0	28.3	15.7	15.7	122.0
37.7	37.7	37.7	179.0	179.0	122.0	127.0	127.0	127.0	117.0	28.3	15.7	15.7	122.0
37.7	37.7	37.7	179.0	179.0	122.0	127.0	127.0	127.0	117.0	28.3	15.7	15.7	122.0
179.0	179.0	179.0	12.0	12.0	179.0	179.0	179.0	179.0	179.0	179.0	179.0	179.0	179.0
179.0	179.0	179.0	9.6	9.6	179.0	179.0	179.0	179.0	179.0	179.0	179.0	179.0	179.0
107.0	107.0	107.0	179.0	179.0	122.0	127.0	127.0	127.0	117.0	107.0	107.0	107.0	122.0
179.0	179.0	179.0	12.0	12.0	179.0	179.0	179.0	179.0	179.0	179.0	179.0	179.0	179.0
179.0	179.0	179.0	7.2	7.2	179.0	179.0	179.0	179.0	179.0	179.0	179.0	179.0	179.0
179.0	179.0	179.0	7.2	7.2	179.0	179.0	179.0	179.0	179.0	179.0	179.0	179.0	179.0
179.0	179.0	179.0	7.2	7.2	179.0	179.0	179.0	179.0	179.0	179.0	179.0	179.0	179.0
117.0	117.0	117.0	179.0	179.0	122.0	127.0	127.0	127.0	97.3	117.0	117.0	117.0	122.0
179.0	179.0	179.0	12.0	12.0	179.0	179.0	179.0	179.0	179.0	179.0	179.0	179.0	179.0
127.0	127.0	127.0	179.0	179.0	127.0	95.0	95.0	95.0	127.0	127.0	127.0	127.0	127.0
127.0	127.0	127.0	179.0	179.0	127.0	95.0	95.0	95.0	127.0	127.0	127.0	127.0	127.0
127.0	127.0	127.0	179.0	179.0	127.0	95.0	95.0	95.0	127.0	127.0	127.0	127.0	127.0
400.0	400.0	400.0	400.0	400.0	400.0	400.0	400.0	400.0	400.0	400.0	400.0	400.0	400.0
37.7	37.7	37.7	179.0	179.0	122.0	127.0	127.0	127.0	117.0	18.9	28.3	28.3	122.0
127.0	127.0	127.0	179.0	179.0	127.0	95.0	95.0	95.0	127.0	127.0	127.0	127.0	127.0
37.7	37.7	37.7	179.0	179.0	122.0	127.0	127.0	127.0	117.0	34.6	34.6	34.6	122.0
179.0	179.0	179.0	12.0	12.0	179.0	179.0	179.0	179.0	179.0	179.0	179.0	179.0	179.0
179.0	179.0	179.0	12.0	12.0	179.0	179.0	179.0	179.0	179.0	179.0	179.0	179.0	179.0
179.0	179.0	179.0	12.0	12.0	179.0	179.0	179.0	179.0	179.0	179.0	179.0	179.0	179.0
37.7	37.7	37.7	179.0	179.0	122.0	127.0	127.0	127.0	117.0	31.4	31.4	31.4	122.0
37.7	37.7	37.7	179.0	179.0	122.0	127.0	127.0	127.0	117.0	31.4	31.4	31.4	122.0
127.0	127.0	127.0	179.0	179.0	127.0	112.5	112.5	112.5	127.0	127.0	127.0	127.0	127.0
117.0	117.0	117.0	179.0	179.0	122.0	127.0	127.0	127.0	107.0	117.0	117.0	117.0	122.0
127.0	127.0	127.0	179.0	179.0	127.0	112.5	112.5	112.5	127.0	127.0	127.0	127.0	127.0
127.0	127.0	127.0	179.0	179.0	127.0	112.5	112.5	112.5	127.0	127.0	127.0	127.0	127.0
37.7	37.7	37.7	179.0	179.0	122.0	127.0	127.0	127.0	117.0	31.4	31.4	31.4	122.0
37.7	37.7	37.7	179.0	179.0	122.0	127.0	127.0	127.0	117.0	31.4	31.4	31.4	122.0

179.0	179.0	179.0	12.0	12.0	179.0	179.0	179.0	179.0	179.0	179.0	179.0	179.0	179.0
122.0	122.0	122.0	179.0	179.0	45.1	127.0	127.0	127.0	122.0	122.0	122.0	122.0	45.1
37.7	37.7	37.7	179.0	179.0	122.0	127.0	127.0	127.0	117.0	28.3	22.0	22.0	122.0
37.7	37.7	37.7	179.0	179.0	122.0	127.0	127.0	127.0	117.0	28.3	22.0	22.0	122.0
37.7	37.7	37.7	179.0	179.0	122.0	127.0	127.0	127.0	117.0	28.3	22.0	22.0	122.0
37.7	37.7	37.7	179.0	179.0	122.0	127.0	127.0	127.0	117.0	28.3	22.0	22.0	122.0
37.7	37.7	37.7	179.0	179.0	122.0	127.0	127.0	127.0	117.0	28.3	22.0	22.0	122.0
127.0	127.0	127.0	179.0	179.0	127.0	112.5	112.5	112.5	127.0	127.0	127.0	127.0	127.0
179.0	179.0	179.0	12.0	12.0	179.0	179.0	179.0	179.0	179.0	179.0	179.0	179.0	179.0
179.0	179.0	179.0	12.0	12.0	179.0	179.0	179.0	179.0	179.0	179.0	179.0	179.0	179.0
179.0	179.0	179.0	12.0	12.0	179.0	179.0	179.0	179.0	179.0	179.0	179.0	179.0	179.0
179.0	179.0	179.0	12.0	12.0	179.0	179.0	179.0	179.0	179.0	179.0	179.0	179.0	179.0
127.0	127.0	127.0	179.0	179.0	127.0	112.5	112.5	112.5	127.0	127.0	127.0	127.0	127.0
37.7	37.7	37.7	179.0	179.0	122.0	127.0	127.0	127.0	117.0	28.3	15.7	15.7	122.0
90.0	90.0	90.0	179.0	179.0	122.0	127.0	127.0	127.0	117.0	90.0	90.0	90.0	122.0
179.0	179.0	179.0	42.0	42.0	179.0	179.0	179.0	179.0	179.0	179.0	179.0	179.0	179.0
179.0	179.0	179.0	42.0	42.0	179.0	179.0	179.0	179.0	179.0	179.0	179.0	179.0	179.0
37.7	37.7	37.7	179.0	179.0	122.0	127.0	127.0	127.0	117.0	28.3	18.9	18.9	122.0
37.7	37.7	37.7	179.0	179.0	122.0	127.0	127.0	127.0	117.0	28.3	15.7	15.7	122.0
37.7	37.7	37.7	179.0	179.0	122.0	127.0	127.0	127.0	117.0	28.3	15.7	15.7	122.0
127.0	127.0	127.0	179.0	179.0	127.0	24.0	24.0	24.0	127.0	127.0	127.0	127.0	127.0
40.9	40.9	40.9	179.0	179.0	122.0	127.0	127.0	127.0	117.0	40.9	40.9	40.9	122.0
117.0	117.0	117.0	179.0	179.0	122.0	127.0	127.0	127.0	107.0	117.0	117.0	117.0	122.0
179.0	179.0	179.0	12.0	12.0	179.0	179.0	179.0	179.0	179.0	179.0	179.0	179.0	179.0
127.0	127.0	127.0	179.0	179.0	127.0	112.5	112.5	112.5	127.0	127.0	127.0	127.0	127.0
127.0	127.0	127.0	179.0	179.0	127.0	112.5	112.5	112.5	127.0	127.0	127.0	127.0	127.0
127.0	127.0	127.0	179.0	179.0	127.0	112.5	112.5	112.5	127.0	127.0	127.0	127.0	127.0
179.0	179.0	179.0	42.0	42.0	179.0	179.0	179.0	179.0	179.0	179.0	179.0	179.0	179.0
127.0	127.0	127.0	179.0	179.0	127.0	112.5	112.5	112.5	127.0	127.0	127.0	127.0	127.0
40.9	40.9	40.9	179.0	179.0	122.0	127.0	127.0	127.0	117.0	40.9	40.9	40.9	122.0
127.0	127.0	127.0	179.0	179.0	127.0	112.5	112.5	112.5	127.0	127.0	127.0	127.0	127.0
179.0	179.0	179.0	12.0	12.0	179.0	179.0	179.0	179.0	179.0	179.0	179.0	179.0	179.0
122.0	122.0	122.0	179.0	179.0	32.2	127.0	127.0	127.0	122.0	122.0	122.0	122.0	38.6
450.0	450.0	450.0	450.0	450.0	450.0	450.0	450.0	450.0	450.0	450.0	450.0	450.0	450.0
500.0	500.0	500.0	500.0	500.0	500.0	500.0	500.0	500.0	500.0	500.0	500.0	500.0	500.0
117.0	117.0	117.0	179.0	179.0	122.0	127.0	127.0	127.0	97.3	117.0	117.0	117.0	122.0
127.0	127.0	127.0	179.0	179.0	127.0	95.0	95.0	95.0	127.0	127.0	127.0	127.0	127.0
127.0	127.0	127.0	179.0	179.0	127.0	112.5	112.5	112.5	127.0	127.0	127.0	127.0	127.0
147.0	147.0	147.0	179.0	179.0	147.0	147.0	147.0	147.0	147.0	147.0	147.0	147.0	147.0
147.0	147.0	147.0	179.0	179.0	147.0	147.0	147.0	147.0	147.0	147.0	147.0	147.0	147.0
122.0	122.0	122.0	179.0	179.0	16.1	127.0	127.0	127.0	122.0	122.0	122.0	122.0	38.6
179.0	179.0	179.0	12.0	12.0	179.0	179.0	179.0	179.0	179.0	179.0	179.0	179.0	179.0
179.0	179.0	179.0	9.6	9.6	179.0	179.0	179.0	179.0	179.0	179.0	179.0	179.0	179.0
37.7	37.7	37.7	179.0	179.0	122.0	127.0	127.0	127.0	117.0	28.3	15.7	15.7	122.0
325.0	325.0	325.0	325.0	325.0	325.0	325.0	325.0	325.0	325.0	325.0	325.0	325.0	325.0
117.0	117.0	117.0	179.0	179.0	122.0	127.0	127.0	127.0	107.0	117.0	117.0	117.0	122.0
117.0	117.0	117.0	179.0	179.0	122.0	127.0	127.0	127.0	107.0	117.0	117.0	117.0	122.0
117.0	117.0	117.0	179.0	179.0	122.0	127.0	127.0	127.0	107.0	117.0	117.0	117.0	122.0
179.0	179.0	179.0	12.0	12.0	179.0	179.0	179.0	179.0	179.0	179.0	179.0	179.0	179.0
179.0	179.0	179.0	12.0	12.0	179.0	179.0	179.0	179.0	179.0	179.0	179.0	179.0	179.0
179.0	179.0	179.0	12.0	12.0	179.0	179.0	179.0	179.0	179.0	179.0	179.0	179.0	179.0
179.0	179.0	179.0	12.0	12.0	179.0	179.0	179.0	179.0	179.0	179.0	179.0	179.0	179.0
122.0	122.0	122.0	179.0	179.0	84.0	127.0	127.0	127.0	122.0	122.0	122.0	122.0	84.0
122.0	122.0	122.0	179.0	179.0	84.0	127.0	127.0	127.0	122.0	122.0	122.0	122.0	84.0
127.0	127.0	127.0	179.0	179.0	127.0	112.5	112.5	112.5	127.0	127.0	127.0	127.0	127.0
127.0	127.0	127.0	179.0	179.0	127.0	112.5	112.5	112.5	127.0	127.0	127.0	127.0	127.0
127.0	127.0	127.0	179.0	179.0	127.0	112.5	112.5	112.5	127.0	127.0	127.0	127.0	127.0
117.0	117.0	117.0	179.0	179.0	122.0	127.0	127.0	127.0	107.0	117.0	117.0	117.0	122.0
127.0	127.0	127.0	179.0	179.0	127.0	112.5	112.5	112.5	127.0	127.0	127.0	127.0	127.0
127.0	127.0	127.0	179.0	179.0	127.0	112.5	112.5	112.5	127.0	127.0	127.0	127.0	127.0
127.0	127.0	127.0	179.0	179.0	127.0	112.5	112.5	112.5	127.0	127.0	127.0	127.0	127.0
127.0	127.0	127.0	179.0	179.0	127.0	112.5	112.5	112.5	127.0	127.0	127.0	127.0	127.0
122.0	122.0	122.0	179.0	179.0	84.0	127.0	127.0	127.0	122.0	122.0	122.0	122.0	84.0
122.0	122.0	122.0	179.0	179.0	84.0	127.0	127.0	127.0	122.0	122.0	122.0	122.0	84.0
122.0	122.0	122.0	179.0	179.0	84.0	127.0	127.0	127.0	122.0	122.0	122.0	122.0	84.0
122.0	122.0	122.0	179.0	179.0	84.0	127.0	127.0	127.0	122.0	122.0	122.0	122.0	84.0
122.0	122.0	122.0	179.0	179.0	32.2	127.0	127.0	127.0	122.0	122.0	122.0	122.0	38.6
127.0	127.0	127.0	179.0	179.0	127.0	112.5	112.5	112.5	127.0	127.0	127.0	127.0	127.0
127.0	127.0	127.0	179.0	179.0	127.0	112.5	112.5	112.5	127.0	127.0	127.0	127.0	127.0
127.0	127.0	127.0	179.0	179.0	127.0	112.5	112.5	112.5	127.0	127.0	127.0	127.0	127.0
122.0	122.0	122.0	179.0	179.0	51.5	127.0	127.0	127.0	122.0	122.0	122.0	122.0	51.5
122.0	122.0	122.0	179.0	179.0	32.2	127.0	127.0	127.0	122.0	122.0	122.0	122.0	38.6
122.0	122.0	122.0	179.0	179.0	32.2	127.0	127.0	127.0	122.0	122.0	122.0	122.0	38.6
0.0	12.6	12.6	179.0	179.0	122.0	127.0	127.0	127.0	117.0	37.7	37.7	37.7	122.0

12.6	0.0	12.6	179.0	179.0	122.0	127.0	127.0	127.0	117.0	37.7	37.7	37.7	122.0
12.6	12.6	0.0	179.0	179.0	122.0	127.0	127.0	127.0	117.0	37.7	37.7	37.7	122.0
179.0	179.0	179.0	0.0	3.6	179.0	179.0	179.0	179.0	179.0	179.0	179.0	179.0	179.0
179.0	179.0	179.0	3.6	0.0	179.0	179.0	179.0	179.0	179.0	179.0	179.0	179.0	179.0
122.0	122.0	122.0	179.0	179.0	0.0	127.0	127.0	127.0	122.0	122.0	122.0	122.0	38.6
127.0	127.0	127.0	179.0	179.0	127.0	0.0	18.0	18.0	127.0	127.0	127.0	127.0	127.0
127.0	127.0	127.0	179.0	179.0	127.0	18.0	0.0	6.0	127.0	127.0	127.0	127.0	127.0
127.0	127.0	127.0	179.0	179.0	127.0	18.0	6.0	0.0	127.0	127.0	127.0	127.0	127.0
117.0	117.0	117.0	179.0	179.0	122.0	127.0	127.0	127.0	0.0	117.0	117.0	117.0	122.0
37.7	37.7	37.7	179.0	179.0	122.0	127.0	127.0	127.0	117.0	0.0	28.3	28.3	122.0
37.7	37.7	37.7	179.0	179.0	122.0	127.0	127.0	127.0	117.0	28.3	0.0	7.9	122.0
37.7	37.7	37.7	179.0	179.0	122.0	127.0	127.0	127.0	117.0	28.3	7.9	0.0	122.0
122.0	122.0	122.0	179.0	179.0	38.6	127.0	127.0	127.0	122.0	122.0	122.0	122.0	0.0
122.0	122.0	122.0	179.0	179.0	32.2	127.0	127.0	127.0	122.0	122.0	122.0	122.0	38.6
40.9	40.9	40.9	179.0	179.0	122.0	127.0	127.0	127.0	117.0	40.9	40.9	40.9	122.0
37.7	37.7	37.7	179.0	179.0	122.0	127.0	127.0	127.0	117.0	28.3	15.7	15.7	122.0
107.0	107.0	107.0	179.0	179.0	122.0	127.0	127.0	127.0	117.0	107.0	107.0	107.0	122.0
37.7	37.7	37.7	179.0	179.0	122.0	127.0	127.0	127.0	117.0	28.3	25.1	25.1	122.0
127.0	127.0	127.0	179.0	179.0	127.0	112.5	112.5	112.5	127.0	127.0	127.0	127.0	127.0
127.0	127.0	127.0	179.0	179.0	127.0	112.5	112.5	112.5	127.0	127.0	127.0	127.0	127.0
127.0	127.0	127.0	179.0	179.0	127.0	112.5	112.5	112.5	127.0	127.0	127.0	127.0	127.0
127.0	127.0	127.0	179.0	179.0	127.0	112.5	112.5	112.5	127.0	127.0	127.0	127.0	127.0
127.0	127.0	127.0	179.0	179.0	127.0	112.5	112.5	112.5	127.0	127.0	127.0	127.0	127.0
40.9	40.9	40.9	179.0	179.0	122.0	127.0	127.0	127.0	117.0	40.9	40.9	40.9	122.0
25.1	25.1	25.1	179.0	179.0	122.0	127.0	127.0	127.0	117.0	37.7	37.7	37.7	122.0
179.0	179.0	179.0	72.0	72.0	179.0	179.0	179.0	179.0	179.0	179.0	179.0	179.0	179.0
127.0	127.0	127.0	179.0	179.0	127.0	112.5	112.5	112.5	127.0	127.0	127.0	127.0	127.0
117.0	117.0	117.0	179.0	179.0	122.0	127.0	127.0	127.0	107.0	117.0	117.0	117.0	122.0
117.0	117.0	117.0	179.0	179.0	122.0	127.0	127.0	127.0	107.0	117.0	117.0	117.0	122.0
117.0	117.0	117.0	179.0	179.0	122.0	127.0	127.0	127.0	107.0	117.0	117.0	117.0	122.0
127.0	127.0	127.0	179.0	179.0	127.0	112.5	112.5	112.5	127.0	127.0	127.0	127.0	127.0
127.0	127.0	127.0	179.0	179.0	127.0	112.5	112.5	112.5	127.0	127.0	127.0	127.0	127.0
127.0	127.0	127.0	179.0	179.0	127.0	112.5	112.5	112.5	127.0	127.0	127.0	127.0	127.0
127.0	127.0	127.0	179.0	179.0	127.0	112.5	112.5	112.5	127.0	127.0	127.0	127.0	127.0
127.0	127.0	127.0	179.0	179.0	127.0	112.5	112.5	112.5	127.0	127.0	127.0	127.0	127.0
127.0	127.0	127.0	179.0	179.0	127.0	112.5	112.5	112.5	127.0	127.0	127.0	127.0	127.0
127.0	127.0	127.0	179.0	179.0	127.0	112.5	112.5	112.5	127.0	127.0	127.0	127.0	127.0

Stellaria_m	Tanacetum	Taraxacum	Torilis_japc	Tragopogo	Trifolium_a	Trifolium_c	Trifolium_c	Trifolium_p	Trifolium_r	Tripleurosp	Tussilago_f	Typha	Ulmus
122.0	16.3	40.9	107.0	40.9	127.0	127.0	127.0	127.0	127.0	32.7	40.9	179.0	127.0
179.0	179.0	179.0	179.0	179.0	179.0	179.0	179.0	179.0	179.0	179.0	179.0	72.0	179.0
179.0	179.0	179.0	179.0	179.0	179.0	179.0	179.0	179.0	179.0	179.0	179.0	72.0	179.0
179.0	179.0	179.0	179.0	179.0	179.0	179.0	179.0	179.0	179.0	179.0	179.0	72.0	179.0
122.0	117.0	117.0	117.0	117.0	127.0	127.0	127.0	127.0	127.0	117.0	117.0	179.0	127.0
179.0	179.0	179.0	179.0	179.0	179.0	179.0	179.0	179.0	179.0	179.0	179.0	72.0	179.0
122.0	44.0	44.0	107.0	44.0	127.0	127.0	127.0	127.0	127.0	44.0	44.0	179.0	127.0
19.3	122.0	122.0	122.0	122.0	127.0	127.0	127.0	127.0	127.0	122.0	122.0	179.0	127.0
122.0	32.7	40.9	107.0	40.9	127.0	127.0	127.0	127.0	127.0	21.8	40.9	179.0	127.0
122.0	32.7	40.9	107.0	40.9	127.0	127.0	127.0	127.0	127.0	21.8	40.9	179.0	127.0
127.0	127.0	127.0	127.0	127.0	112.5	112.5	112.5	112.5	112.5	127.0	127.0	179.0	112.5
127.0	127.0	127.0	127.0	127.0	90.7	90.7	90.7	90.7	90.7	127.0	127.0	179.0	83.3
179.0	179.0	179.0	179.0	179.0	179.0	179.0	179.0	179.0	179.0	179.0	179.0	72.0	179.0
179.0	179.0	179.0	179.0	179.0	179.0	179.0	179.0	179.0	179.0	179.0	179.0	72.0	179.0
179.0	179.0	179.0	179.0	179.0	179.0	179.0	179.0	179.0	179.0	179.0	179.0	72.0	179.0
179.0	179.0	179.0	179.0	179.0	179.0	179.0	179.0	179.0	179.0	179.0	179.0	72.0	179.0
179.0	179.0	179.0	179.0	179.0	179.0	179.0	179.0	179.0	179.0	179.0	179.0	72.0	179.0
179.0	179.0	179.0	179.0	179.0	179.0	179.0	179.0	179.0	179.0	179.0	179.0	72.0	179.0
179.0	179.0	179.0	179.0	179.0	179.0	179.0	179.0	179.0	179.0	179.0	179.0	72.0	179.0
179.0	179.0	179.0	179.0	179.0	179.0	179.0	179.0	179.0	179.0	179.0	179.0	72.0	179.0
122.0	44.0	44.0	107.0	44.0	127.0	127.0	127.0	127.0	127.0	44.0	44.0	179.0	127.0
122.0	117.0	117.0	117.0	117.0	127.0	127.0	127.0	127.0	127.0	117.0	117.0	179.0	127.0
12.9	122.0	122.0	122.0	122.0	127.0	127.0	127.0	127.0	127.0	122.0	122.0	179.0	127.0
12.9	122.0	122.0	122.0	122.0	127.0	127.0	127.0	127.0	127.0	122.0	122.0	179.0	127.0
51.5	122.0	122.0	122.0	122.0	127.0	127.0	127.0	127.0	127.0	122.0	122.0	179.0	127.0
51.5	122.0	122.0	122.0	122.0	127.0	127.0	127.0	127.0	127.0	122.0	122.0	179.0	127.0
122.0	40.9	9.4	107.0	25.1	127.0	127.0	127.0	127.0	127.0	40.9	37.7	179.0	127.0
122.0	44.0	44.0	107.0	44.0	127.0	127.0	127.0	127.0	127.0	44.0	44.0	179.0	127.0
122.0	44.0	44.0	107.0	44.0	127.0	127.0	127.0	127.0	127.0	44.0	44.0	179.0	127.0
122.0	117.0	117.0	117.0	117.0	127.0	127.0	127.0	127.0	127.0	117.0	117.0	179.0	127.0
122.0	40.9	28.3	107.0	28.3	127.0	127.0	127.0	127.0	127.0	40.9	37.7	179.0	127.0
179.0	179.0	179.0	179.0	179.0	179.0	179.0	179.0	179.0	179.0	179.0	179.0	72.0	179.0
127.0	127.0	127.0	127.0	127.0	90.7	90.7	90.7	90.7	90.7	127.0	127.0	179.0	76.0
122.0	40.9	6.3	107.0	25.1	127.0	127.0	127.0	127.0	127.0	40.9	37.7	179.0	127.0
122.0	40.9	6.3	107.0	25.1	127.0	127.0	127.0	127.0	127.0	40.9	37.7	179.0	127.0
122.0	40.9	6.3	107.0	25.1	127.0	127.0	127.0	127.0	127.0	40.9	37.7	179.0	127.0
179.0	179.0	179.0	179.0	179.0	179.0	179.0	179.0	179.0	179.0	179.0	179.0	72.0	179.0
179.0	179.0	179.0	179.0	179.0	179.0	179.0	179.0	179.0	179.0	179.0	179.0	72.0	179.0
122.0	107.0	107.0	33.0	107.0	127.0	127.0	127.0	127.0	127.0	107.0	107.0	179.0	127.0
179.0	179.0	179.0	179.0	179.0	179.0	179.0	179.0	179.0	179.0	179.0	179.0	72.0	179.0
179.0	179.0	179.0	179.0	179.0	179.0	179.0	179.0	179.0	179.0	179.0	179.0	72.0	179.0
179.0	179.0	179.0	179.0	179.0	179.0	179.0	179.0	179.0	179.0	179.0	179.0	72.0	179.0
179.0	179.0	179.0	179.0	179.0	179.0	179.0	179.0	179.0	179.0	179.0	179.0	72.0	179.0
122.0	117.0	117.0	117.0	117.0	127.0	127.0	127.0	127.0	127.0	117.0	117.0	179.0	127.0
179.0	179.0	179.0	179.0	179.0	179.0	179.0	179.0	179.0	179.0	179.0	179.0	72.0	179.0
127.0	127.0	127.0	127.0	127.0	112.5	112.5	112.5	112.5	112.5	127.0	127.0	179.0	112.5
127.0	127.0	127.0	127.0	127.0	112.5	112.5	112.5	112.5	112.5	127.0	127.0	179.0	112.5
127.0	127.0	127.0	127.0	127.0	112.5	112.5	112.5	112.5	112.5	127.0	127.0	179.0	112.5
400.0	400.0	400.0	400.0	400.0	400.0	400.0	400.0	400.0	400.0	400.0	400.0	400.0	400.0
122.0	40.9	28.3	107.0	28.3	127.0	127.0	127.0	127.0	127.0	40.9	37.7	179.0	127.0
127.0	127.0	127.0	127.0	127.0	112.5	112.5	112.5	112.5	112.5	127.0	127.0	179.0	112.5
122.0	40.9	34.6	107.0	34.6	127.0	127.0	127.0	127.0	127.0	40.9	37.7	179.0	127.0
179.0	179.0	179.0	179.0	179.0	179.0	179.0	179.0	179.0	179.0	179.0	179.0	72.0	179.0
179.0	179.0	179.0	179.0	179.0	179.0	179.0	179.0	179.0	179.0	179.0	179.0	72.0	179.0
179.0	179.0	179.0	179.0	179.0	179.0	179.0	179.0	179.0	179.0	179.0	179.0	72.0	179.0
122.0	40.9	31.4	107.0	31.4	127.0	127.0	127.0	127.0	127.0	40.9	37.7	179.0	127.0
122.0	40.9	31.4	107.0	31.4	127.0	127.0	127.0	127.0	127.0	40.9	37.7	179.0	127.0
127.0	127.0	127.0	127.0	127.0	90.7	90.7	90.7	90.7	90.7	127.0	127.0	179.0	76.0
122.0	117.0	117.0	117.0	117.0	127.0	127.0	127.0	127.0	127.0	117.0	117.0	179.0	127.0
127.0	127.0	127.0	127.0	127.0	56.0	56.0	56.0	56.0	56.0	127.0	127.0	179.0	90.7
127.0	127.0	127.0	127.0	127.0	90.7	90.7	90.7	90.7	90.7	127.0	127.0	179.0	76.0
122.0	40.9	31.4	107.0	31.4	127.0	127.0	127.0	127.0	127.0	40.9	37.7	179.0	127.0
122.0	40.9	31.4	107.0	31.4	127.0	127.0	127.0	127.0	127.0	40.9	37.7	179.0	127.0

179.0	179.0	179.0	179.0	179.0	179.0	179.0	179.0	179.0	179.0	179.0	179.0	72.0	179.0
45.1	122.0	122.0	122.0	122.0	127.0	127.0	127.0	127.0	127.0	122.0	122.0	179.0	127.0
122.0	40.9	22.0	107.0	25.1	127.0	127.0	127.0	127.0	127.0	40.9	37.7	179.0	127.0
122.0	40.9	22.0	107.0	25.1	127.0	127.0	127.0	127.0	127.0	40.9	37.7	179.0	127.0
122.0	40.9	22.0	107.0	25.1	127.0	127.0	127.0	127.0	127.0	40.9	37.7	179.0	127.0
122.0	40.9	22.0	107.0	25.1	127.0	127.0	127.0	127.0	127.0	40.9	37.7	179.0	127.0
122.0	40.9	22.0	107.0	25.1	127.0	127.0	127.0	127.0	127.0	40.9	37.7	179.0	127.0
127.0	127.0	127.0	127.0	127.0	90.7	90.7	90.7	90.7	90.7	127.0	127.0	179.0	38.0
179.0	179.0	179.0	179.0	179.0	179.0	179.0	179.0	179.0	179.0	179.0	179.0	72.0	179.0
179.0	179.0	179.0	179.0	179.0	179.0	179.0	179.0	179.0	179.0	179.0	179.0	72.0	179.0
179.0	179.0	179.0	179.0	179.0	179.0	179.0	179.0	179.0	179.0	179.0	179.0	72.0	179.0
179.0	179.0	179.0	179.0	179.0	179.0	179.0	179.0	179.0	179.0	179.0	179.0	72.0	179.0
127.0	127.0	127.0	127.0	127.0	98.0	98.0	98.0	98.0	98.0	127.0	127.0	179.0	98.0
122.0	40.9	12.6	107.0	25.1	127.0	127.0	127.0	127.0	127.0	40.9	37.7	179.0	127.0
122.0	90.0	90.0	107.0	90.0	127.0	127.0	127.0	127.0	127.0	90.0	90.0	179.0	127.0
179.0	179.0	179.0	179.0	179.0	179.0	179.0	179.0	179.0	179.0	179.0	179.0	72.0	179.0
179.0	179.0	179.0	179.0	179.0	179.0	179.0	179.0	179.0	179.0	179.0	179.0	72.0	179.0
122.0	40.9	18.9	107.0	25.1	127.0	127.0	127.0	127.0	127.0	40.9	37.7	179.0	127.0
122.0	40.9	12.6	107.0	25.1	127.0	127.0	127.0	127.0	127.0	40.9	37.7	179.0	127.0
122.0	40.9	12.6	107.0	25.1	127.0	127.0	127.0	127.0	127.0	40.9	37.7	179.0	127.0
127.0	127.0	127.0	127.0	127.0	112.5	112.5	112.5	112.5	112.5	127.0	127.0	179.0	112.5
122.0	24.5	40.9	107.0	40.9	127.0	127.0	127.0	127.0	127.0	32.7	40.9	179.0	127.0
122.0	117.0	117.0	117.0	117.0	127.0	127.0	127.0	127.0	127.0	117.0	117.0	179.0	127.0
179.0	179.0	179.0	179.0	179.0	179.0	179.0	179.0	179.0	179.0	179.0	179.0	72.0	179.0
127.0	127.0	127.0	127.0	127.0	44.8	44.8	44.8	44.8	44.8	127.0	127.0	179.0	90.7
127.0	127.0	127.0	127.0	127.0	56.0	56.0	56.0	56.0	56.0	127.0	127.0	179.0	90.7
127.0	127.0	127.0	127.0	127.0	56.0	56.0	56.0	56.0	56.0	127.0	127.0	179.0	90.7
179.0	179.0	179.0	179.0	179.0	179.0	179.0	179.0	179.0	179.0	179.0	179.0	72.0	179.0
127.0	127.0	127.0	127.0	127.0	90.7	90.7	90.7	90.7	90.7	127.0	127.0	179.0	76.0
122.0	16.3	40.9	107.0	40.9	127.0	127.0	127.0	127.0	127.0	32.7	40.9	179.0	127.0
127.0	127.0	127.0	127.0	127.0	33.6	33.6	33.6	33.6	33.6	127.0	127.0	179.0	90.7
179.0	179.0	179.0	179.0	179.0	179.0	179.0	179.0	179.0	179.0	179.0	179.0	72.0	179.0
19.3	122.0	122.0	122.0	122.0	127.0	127.0	127.0	127.0	127.0	122.0	122.0	179.0	127.0
450.0	450.0	450.0	450.0	450.0	450.0	450.0	450.0	450.0	450.0	450.0	450.0	450.0	450.0
500.0	500.0	500.0	500.0	500.0	500.0	500.0	500.0	500.0	500.0	500.0	500.0	500.0	500.0
122.0	117.0	117.0	117.0	117.0	127.0	127.0	127.0	127.0	127.0	117.0	117.0	179.0	127.0
127.0	127.0	127.0	127.0	127.0	112.5	112.5	112.5	112.5	112.5	127.0	127.0	179.0	112.5
127.0	127.0	127.0	127.0	127.0	44.8	44.8	44.8	44.8	44.8	127.0	127.0	179.0	90.7
147.0	147.0	147.0	147.0	147.0	147.0	147.0	147.0	147.0	147.0	147.0	147.0	179.0	147.0
147.0	147.0	147.0	147.0	147.0	147.0	147.0	147.0	147.0	147.0	147.0	147.0	179.0	147.0
32.2	122.0	122.0	122.0	122.0	127.0	127.0	127.0	127.0	127.0	122.0	122.0	179.0	127.0
179.0	179.0	179.0	179.0	179.0	179.0	179.0	179.0	179.0	179.0	179.0	179.0	72.0	179.0
179.0	179.0	179.0	179.0	179.0	179.0	179.0	179.0	179.0	179.0	179.0	179.0	72.0	179.0
122.0	40.9	12.6	107.0	25.1	127.0	127.0	127.0	127.0	127.0	40.9	37.7	179.0	127.0
325.0	325.0	325.0	325.0	325.0	325.0	325.0	325.0	325.0	325.0	325.0	325.0	325.0	325.0
122.0	117.0	117.0	117.0	117.0	127.0	127.0	127.0	127.0	127.0	117.0	117.0	179.0	127.0
122.0	117.0	117.0	117.0	117.0	127.0	127.0	127.0	127.0	127.0	117.0	117.0	179.0	127.0
122.0	117.0	117.0	117.0	117.0	127.0	127.0	127.0	127.0	127.0	117.0	117.0	179.0	127.0
179.0	179.0	179.0	179.0	179.0	179.0	179.0	179.0	179.0	179.0	179.0	179.0	72.0	179.0
179.0	179.0	179.0	179.0	179.0	179.0	179.0	179.0	179.0	179.0	179.0	179.0	72.0	179.0
179.0	179.0	179.0	179.0	179.0	179.0	179.0	179.0	179.0	179.0	179.0	179.0	72.0	179.0
179.0	179.0	179.0	179.0	179.0	179.0	179.0	179.0	179.0	179.0	179.0	179.0	72.0	179.0
84.0	122.0	122.0	122.0	122.0	127.0	127.0	127.0	127.0	127.0	122.0	122.0	179.0	127.0
84.0	122.0	122.0	122.0	122.0	127.0	127.0	127.0	127.0	127.0	122.0	122.0	179.0	127.0
127.0	127.0	127.0	127.0	127.0	98.0	98.0	98.0	98.0	98.0	127.0	127.0	179.0	98.0
127.0	127.0	127.0	127.0	127.0	98.0	98.0	98.0	98.0	98.0	127.0	127.0	179.0	98.0
127.0	127.0	127.0	127.0	127.0	90.7	90.7	90.7	90.7	90.7	127.0	127.0	179.0	76.0
122.0	117.0	117.0	117.0	117.0	127.0	127.0	127.0	127.0	127.0	117.0	117.0	179.0	127.0
127.0	127.0	127.0	127.0	127.0	90.7	90.7	90.7	90.7	90.7	127.0	127.0	179.0	76.0
127.0	127.0	127.0	127.0	127.0	44.8	44.8	44.8	44.8	44.8	127.0	127.0	179.0	90.7
127.0	127.0	127.0	127.0	127.0	90.7	90.7	90.7	90.7	90.7	127.0	127.0	179.0	76.0
127.0	127.0	127.0	127.0	127.0	90.7	90.7	90.7	90.7	90.7	127.0	127.0	179.0	76.0
84.0	122.0	122.0	122.0	122.0	127.0	127.0	127.0	127.0	127.0	122.0	122.0	179.0	127.0
84.0	122.0	122.0	122.0	122.0	127.0	127.0	127.0	127.0	127.0	122.0	122.0	179.0	127.0
84.0	122.0	122.0	122.0	122.0	127.0	127.0	127.0	127.0	127.0	122.0	122.0	179.0	127.0
84.0	122.0	122.0	122.0	122.0	127.0	127.0	127.0	127.0	127.0	122.0	122.0	179.0	127.0
25.8	122.0	122.0	122.0	122.0	127.0	127.0	127.0	127.0	127.0	122.0	122.0	179.0	127.0
127.0	127.0	127.0	127.0	127.0	98.0	98.0	98.0	98.0	98.0	127.0	127.0	179.0	98.0
127.0	127.0	127.0	127.0	127.0	98.0	98.0	98.0	98.0	98.0	127.0	127.0	179.0	98.0
127.0	127.0	127.0	127.0	127.0	98.0	98.0	98.0	98.0	98.0	127.0	127.0	179.0	98.0
51.5	122.0	122.0	122.0	122.0	127.0	127.0	127.0	127.0	127.0	122.0	122.0	179.0	127.0
25.8	122.0	122.0	122.0	122.0	127.0	127.0	127.0	127.0	127.0	122.0	122.0	179.0	127.0
25.8	122.0	122.0	122.0	122.0	127.0	127.0	127.0	127.0	127.0	122.0	122.0	179.0	127.0
122.0	40.9	37.7	107.0	37.7	127.0	127.0	127.0	127.0	127.0	40.9	25.1	179.0	127.0

122.0	40.9	37.7	107.0	37.7	127.0	127.0	127.0	127.0	127.0	40.9	25.1	179.0	127.0
122.0	40.9	37.7	107.0	37.7	127.0	127.0	127.0	127.0	127.0	40.9	25.1	179.0	127.0
179.0	179.0	179.0	179.0	179.0	179.0	179.0	179.0	179.0	179.0	179.0	179.0	72.0	179.0
179.0	179.0	179.0	179.0	179.0	179.0	179.0	179.0	179.0	179.0	179.0	179.0	72.0	179.0
32.2	122.0	122.0	122.0	122.0	127.0	127.0	127.0	127.0	127.0	122.0	122.0	179.0	127.0
127.0	127.0	127.0	127.0	127.0	112.5	112.5	112.5	112.5	112.5	127.0	127.0	179.0	112.5
127.0	127.0	127.0	127.0	127.0	112.5	112.5	112.5	112.5	112.5	127.0	127.0	179.0	112.5
127.0	127.0	127.0	127.0	127.0	112.5	112.5	112.5	112.5	112.5	127.0	127.0	179.0	112.5
122.0	117.0	117.0	117.0	117.0	127.0	127.0	127.0	127.0	127.0	117.0	117.0	179.0	127.0
122.0	40.9	28.3	107.0	28.3	127.0	127.0	127.0	127.0	127.0	40.9	37.7	179.0	127.0
122.0	40.9	15.7	107.0	25.1	127.0	127.0	127.0	127.0	127.0	40.9	37.7	179.0	127.0
122.0	40.9	15.7	107.0	25.1	127.0	127.0	127.0	127.0	127.0	40.9	37.7	179.0	127.0
38.6	122.0	122.0	122.0	122.0	127.0	127.0	127.0	127.0	127.0	122.0	122.0	179.0	127.0
0.0	122.0	122.0	122.0	122.0	127.0	127.0	127.0	127.0	127.0	122.0	122.0	179.0	127.0
122.0	0.0	40.9	107.0	40.9	127.0	127.0	127.0	127.0	127.0	32.7	40.9	179.0	127.0
122.0	40.9	0.0	107.0	25.1	127.0	127.0	127.0	127.0	127.0	40.9	37.7	179.0	127.0
122.0	107.0	107.0	0.0	107.0	127.0	127.0	127.0	127.0	127.0	107.0	107.0	179.0	127.0
122.0	40.9	25.1	107.0	0.0	127.0	127.0	127.0	127.0	127.0	40.9	37.7	179.0	127.0
127.0	127.0	127.0	127.0	127.0	0.0	11.2	11.2	11.2	11.2	127.0	127.0	179.0	90.7
127.0	127.0	127.0	127.0	127.0	11.2	0.0	11.2	11.2	11.2	127.0	127.0	179.0	90.7
127.0	127.0	127.0	127.0	127.0	11.2	11.2	0.0	11.2	11.2	127.0	127.0	179.0	90.7
127.0	127.0	127.0	127.0	127.0	11.2	11.2	11.2	0.0	11.2	127.0	127.0	179.0	90.7
127.0	127.0	127.0	127.0	127.0	11.2	11.2	11.2	11.2	0.0	127.0	127.0	179.0	90.7
122.0	32.7	40.9	107.0	40.9	127.0	127.0	127.0	127.0	127.0	0.0	40.9	179.0	127.0
122.0	40.9	37.7	107.0	37.7	127.0	127.0	127.0	127.0	127.0	40.9	0.0	179.0	127.0
179.0	179.0	179.0	179.0	179.0	179.0	179.0	179.0	179.0	179.0	179.0	179.0	0.0	179.0
127.0	127.0	127.0	127.0	127.0	90.7	90.7	90.7	90.7	90.7	127.0	127.0	179.0	0.0
122.0	117.0	117.0	117.0	117.0	127.0	127.0	127.0	127.0	127.0	117.0	117.0	179.0	127.0
122.0	117.0	117.0	117.0	117.0	127.0	127.0	127.0	127.0	127.0	117.0	117.0	179.0	127.0
122.0	117.0	117.0	117.0	117.0	127.0	127.0	127.0	127.0	127.0	117.0	117.0	179.0	127.0
127.0	127.0	127.0	127.0	127.0	22.4	22.4	22.4	22.4	22.4	127.0	127.0	179.0	90.7
127.0	127.0	127.0	127.0	127.0	22.4	22.4	22.4	22.4	22.4	127.0	127.0	179.0	90.7
127.0	127.0	127.0	127.0	127.0	22.4	22.4	22.4	22.4	22.4	127.0	127.0	179.0	90.7
127.0	127.0	127.0	127.0	127.0	22.4	22.4	22.4	22.4	22.4	127.0	127.0	179.0	90.7
127.0	127.0	127.0	127.0	127.0	98.0	98.0	98.0	98.0	98.0	127.0	127.0	179.0	98.0
127.0	127.0	127.0	127.0	127.0	98.0	98.0	98.0	98.0	98.0	127.0	127.0	179.0	98.0
127.0	127.0	127.0	127.0	127.0	98.0	98.0	98.0	98.0	98.0	127.0	127.0	179.0	98.0

Verbascum	Veronica_c	Veronica_c	Vicia_angu	Vicia_hirsu	Vicia_tetra	Vicia_villos	Viola_arver	Viola_canir	Viola_tricolor_ag
117.0	117.0	117.0	127.0	127.0	127.0	127.0	127.0	127.0	127.0
179.0	179.0	179.0	179.0	179.0	179.0	179.0	179.0	179.0	179.0
179.0	179.0	179.0	179.0	179.0	179.0	179.0	179.0	179.0	179.0
179.0	179.0	179.0	179.0	179.0	179.0	179.0	179.0	179.0	179.0
43.0	63.0	63.0	127.0	127.0	127.0	127.0	127.0	127.0	127.0
179.0	179.0	179.0	179.0	179.0	179.0	179.0	179.0	179.0	179.0
117.0	117.0	117.0	127.0	127.0	127.0	127.0	127.0	127.0	127.0
122.0	122.0	122.0	127.0	127.0	127.0	127.0	127.0	127.0	127.0
117.0	117.0	117.0	127.0	127.0	127.0	127.0	127.0	127.0	127.0
117.0	117.0	117.0	127.0	127.0	127.0	127.0	127.0	127.0	127.0
127.0	127.0	127.0	112.5	112.5	112.5	112.5	112.5	112.5	112.5
127.0	127.0	127.0	90.7	90.7	90.7	90.7	98.0	98.0	98.0
179.0	179.0	179.0	179.0	179.0	179.0	179.0	179.0	179.0	179.0
179.0	179.0	179.0	179.0	179.0	179.0	179.0	179.0	179.0	179.0
179.0	179.0	179.0	179.0	179.0	179.0	179.0	179.0	179.0	179.0
179.0	179.0	179.0	179.0	179.0	179.0	179.0	179.0	179.0	179.0
179.0	179.0	179.0	179.0	179.0	179.0	179.0	179.0	179.0	179.0
179.0	179.0	179.0	179.0	179.0	179.0	179.0	179.0	179.0	179.0
179.0	179.0	179.0	179.0	179.0	179.0	179.0	179.0	179.0	179.0
179.0	179.0	179.0	179.0	179.0	179.0	179.0	179.0	179.0	179.0
117.0	117.0	117.0	127.0	127.0	127.0	127.0	127.0	127.0	127.0
107.0	107.0	107.0	127.0	127.0	127.0	127.0	127.0	127.0	127.0
122.0	122.0	122.0	127.0	127.0	127.0	127.0	127.0	127.0	127.0
122.0	122.0	122.0	127.0	127.0	127.0	127.0	127.0	127.0	127.0
122.0	122.0	122.0	127.0	127.0	127.0	127.0	127.0	127.0	127.0
122.0	122.0	122.0	127.0	127.0	127.0	127.0	127.0	127.0	127.0
117.0	117.0	117.0	127.0	127.0	127.0	127.0	127.0	127.0	127.0
117.0	117.0	117.0	127.0	127.0	127.0	127.0	127.0	127.0	127.0
117.0	117.0	117.0	127.0	127.0	127.0	127.0	127.0	127.0	127.0
107.0	107.0	107.0	127.0	127.0	127.0	127.0	127.0	127.0	127.0
117.0	117.0	117.0	127.0	127.0	127.0	127.0	127.0	127.0	127.0
179.0	179.0	179.0	179.0	179.0	179.0	179.0	179.0	179.0	179.0
127.0	127.0	127.0	90.7	90.7	90.7	90.7	98.0	98.0	98.0
117.0	117.0	117.0	127.0	127.0	127.0	127.0	127.0	127.0	127.0
117.0	117.0	117.0	127.0	127.0	127.0	127.0	127.0	127.0	127.0
117.0	117.0	117.0	127.0	127.0	127.0	127.0	127.0	127.0	127.0
179.0	179.0	179.0	179.0	179.0	179.0	179.0	179.0	179.0	179.0
179.0	179.0	179.0	179.0	179.0	179.0	179.0	179.0	179.0	179.0
117.0	117.0	117.0	127.0	127.0	127.0	127.0	127.0	127.0	127.0
179.0	179.0	179.0	179.0	179.0	179.0	179.0	179.0	179.0	179.0
179.0	179.0	179.0	179.0	179.0	179.0	179.0	179.0	179.0	179.0
179.0	179.0	179.0	179.0	179.0	179.0	179.0	179.0	179.0	179.0
179.0	179.0	179.0	179.0	179.0	179.0	179.0	179.0	179.0	179.0
107.0	107.0	107.0	127.0	127.0	127.0	127.0	127.0	127.0	127.0
179.0	179.0	179.0	179.0	179.0	179.0	179.0	179.0	179.0	179.0
127.0	127.0	127.0	112.5	112.5	112.5	112.5	112.5	112.5	112.5
127.0	127.0	127.0	112.5	112.5	112.5	112.5	112.5	112.5	112.5
127.0	127.0	127.0	112.5	112.5	112.5	112.5	112.5	112.5	112.5
400.0	400.0	400.0	400.0	400.0	400.0	400.0	400.0	400.0	400.0
117.0	117.0	117.0	127.0	127.0	127.0	127.0	127.0	127.0	127.0
127.0	127.0	127.0	112.5	112.5	112.5	112.5	112.5	112.5	112.5
117.0	117.0	117.0	127.0	127.0	127.0	127.0	127.0	127.0	127.0
179.0	179.0	179.0	179.0	179.0	179.0	179.0	179.0	179.0	179.0
179.0	179.0	179.0	179.0	179.0	179.0	179.0	179.0	179.0	179.0
179.0	179.0	179.0	179.0	179.0	179.0	179.0	179.0	179.0	179.0
117.0	117.0	117.0	127.0	127.0	127.0	127.0	127.0	127.0	127.0
117.0	117.0	117.0	127.0	127.0	127.0	127.0	127.0	127.0	127.0
127.0	127.0	127.0	90.7	90.7	90.7	90.7	98.0	98.0	98.0
43.0	63.0	63.0	127.0	127.0	127.0	127.0	127.0	127.0	127.0
127.0	127.0	127.0	56.0	56.0	56.0	56.0	98.0	98.0	98.0
127.0	127.0	127.0	90.7	90.7	90.7	90.7	98.0	98.0	98.0
117.0	117.0	117.0	127.0	127.0	127.0	127.0	127.0	127.0	127.0
117.0	117.0	117.0	127.0	127.0	127.0	127.0	127.0	127.0	127.0

179.0	179.0	179.0	179.0	179.0	179.0	179.0	179.0	179.0	179.0
122.0	122.0	122.0	127.0	127.0	127.0	127.0	127.0	127.0	127.0
117.0	117.0	117.0	127.0	127.0	127.0	127.0	127.0	127.0	127.0
117.0	117.0	117.0	127.0	127.0	127.0	127.0	127.0	127.0	127.0
117.0	117.0	117.0	127.0	127.0	127.0	127.0	127.0	127.0	127.0
117.0	117.0	117.0	127.0	127.0	127.0	127.0	127.0	127.0	127.0
117.0	117.0	117.0	127.0	127.0	127.0	127.0	127.0	127.0	127.0
127.0	127.0	127.0	90.7	90.7	90.7	90.7	98.0	98.0	98.0
179.0	179.0	179.0	179.0	179.0	179.0	179.0	179.0	179.0	179.0
179.0	179.0	179.0	179.0	179.0	179.0	179.0	179.0	179.0	179.0
179.0	179.0	179.0	179.0	179.0	179.0	179.0	179.0	179.0	179.0
179.0	179.0	179.0	179.0	179.0	179.0	179.0	179.0	179.0	179.0
127.0	127.0	127.0	98.0	98.0	98.0	98.0	54.0	54.0	54.0
117.0	117.0	117.0	127.0	127.0	127.0	127.0	127.0	127.0	127.0
117.0	117.0	117.0	127.0	127.0	127.0	127.0	127.0	127.0	127.0
179.0	179.0	179.0	179.0	179.0	179.0	179.0	179.0	179.0	179.0
179.0	179.0	179.0	179.0	179.0	179.0	179.0	179.0	179.0	179.0
117.0	117.0	117.0	127.0	127.0	127.0	127.0	127.0	127.0	127.0
117.0	117.0	117.0	127.0	127.0	127.0	127.0	127.0	127.0	127.0
117.0	117.0	117.0	127.0	127.0	127.0	127.0	127.0	127.0	127.0
127.0	127.0	127.0	112.5	112.5	112.5	112.5	112.5	112.5	112.5
117.0	117.0	117.0	127.0	127.0	127.0	127.0	127.0	127.0	127.0
63.0	50.4	50.4	127.0	127.0	127.0	127.0	127.0	127.0	127.0
179.0	179.0	179.0	179.0	179.0	179.0	179.0	179.0	179.0	179.0
127.0	127.0	127.0	44.8	44.8	44.8	44.8	98.0	98.0	98.0
127.0	127.0	127.0	56.0	56.0	56.0	56.0	98.0	98.0	98.0
127.0	127.0	127.0	56.0	56.0	56.0	56.0	98.0	98.0	98.0
179.0	179.0	179.0	179.0	179.0	179.0	179.0	179.0	179.0	179.0
127.0	127.0	127.0	90.7	90.7	90.7	90.7	98.0	98.0	98.0
117.0	117.0	117.0	127.0	127.0	127.0	127.0	127.0	127.0	127.0
127.0	127.0	127.0	33.6	33.6	33.6	33.6	98.0	98.0	98.0
179.0	179.0	179.0	179.0	179.0	179.0	179.0	179.0	179.0	179.0
122.0	122.0	122.0	127.0	127.0	127.0	127.0	127.0	127.0	127.0
450.0	450.0	450.0	450.0	450.0	450.0	450.0	450.0	450.0	450.0
500.0	500.0	500.0	500.0	500.0	500.0	500.0	500.0	500.0	500.0
107.0	107.0	107.0	127.0	127.0	127.0	127.0	127.0	127.0	127.0
127.0	127.0	127.0	112.5	112.5	112.5	112.5	112.5	112.5	112.5
127.0	127.0	127.0	44.8	44.8	44.8	44.8	98.0	98.0	98.0
147.0	147.0	147.0	147.0	147.0	147.0	147.0	147.0	147.0	147.0
147.0	147.0	147.0	147.0	147.0	147.0	147.0	147.0	147.0	147.0
122.0	122.0	122.0	127.0	127.0	127.0	127.0	127.0	127.0	127.0
179.0	179.0	179.0	179.0	179.0	179.0	179.0	179.0	179.0	179.0
179.0	179.0	179.0	179.0	179.0	179.0	179.0	179.0	179.0	179.0
117.0	117.0	117.0	127.0	127.0	127.0	127.0	127.0	127.0	127.0
325.0	325.0	325.0	325.0	325.0	325.0	325.0	325.0	325.0	325.0
63.0	37.8	37.8	127.0	127.0	127.0	127.0	127.0	127.0	127.0
63.0	37.8	37.8	127.0	127.0	127.0	127.0	127.0	127.0	127.0
63.0	37.8	37.8	127.0	127.0	127.0	127.0	127.0	127.0	127.0
179.0	179.0	179.0	179.0	179.0	179.0	179.0	179.0	179.0	179.0
179.0	179.0	179.0	179.0	179.0	179.0	179.0	179.0	179.0	179.0
179.0	179.0	179.0	179.0	179.0	179.0	179.0	179.0	179.0	179.0
179.0	179.0	179.0	179.0	179.0	179.0	179.0	179.0	179.0	179.0
122.0	122.0	122.0	127.0	127.0	127.0	127.0	127.0	127.0	127.0
122.0	122.0	122.0	127.0	127.0	127.0	127.0	127.0	127.0	127.0
127.0	127.0	127.0	98.0	98.0	98.0	98.0	81.0	81.0	81.0
127.0	127.0	127.0	98.0	98.0	98.0	98.0	81.0	81.0	81.0
127.0	127.0	127.0	90.7	90.7	90.7	90.7	98.0	98.0	98.0
43.0	63.0	63.0	127.0	127.0	127.0	127.0	127.0	127.0	127.0
127.0	127.0	127.0	90.7	90.7	90.7	90.7	98.0	98.0	98.0
127.0	127.0	127.0	44.8	44.8	44.8	44.8	98.0	98.0	98.0
127.0	127.0	127.0	90.7	90.7	90.7	90.7	98.0	98.0	98.0
127.0	127.0	127.0	90.7	90.7	90.7	90.7	98.0	98.0	98.0
122.0	122.0	122.0	127.0	127.0	127.0	127.0	127.0	127.0	127.0
122.0	122.0	122.0	127.0	127.0	127.0	127.0	127.0	127.0	127.0
122.0	122.0	122.0	127.0	127.0	127.0	127.0	127.0	127.0	127.0
122.0	122.0	122.0	127.0	127.0	127.0	127.0	127.0	127.0	127.0
122.0	122.0	122.0	127.0	127.0	127.0	127.0	127.0	127.0	127.0
127.0	127.0	127.0	98.0	98.0	98.0	98.0	81.0	81.0	81.0
127.0	127.0	127.0	98.0	98.0	98.0	98.0	81.0	81.0	81.0
127.0	127.0	127.0	98.0	98.0	98.0	98.0	81.0	81.0	81.0
122.0	122.0	122.0	127.0	127.0	127.0	127.0	127.0	127.0	127.0
122.0	122.0	122.0	127.0	127.0	127.0	127.0	127.0	127.0	127.0
122.0	122.0	122.0	127.0	127.0	127.0	127.0	127.0	127.0	127.0
117.0	117.0	117.0	127.0	127.0	127.0	127.0	127.0	127.0	127.0

117.0	117.0	117.0	127.0	127.0	127.0	127.0	127.0	127.0	127.0
117.0	117.0	117.0	127.0	127.0	127.0	127.0	127.0	127.0	127.0
179.0	179.0	179.0	179.0	179.0	179.0	179.0	179.0	179.0	179.0
179.0	179.0	179.0	179.0	179.0	179.0	179.0	179.0	179.0	179.0
122.0	122.0	122.0	127.0	127.0	127.0	127.0	127.0	127.0	127.0
127.0	127.0	127.0	112.5	112.5	112.5	112.5	112.5	112.5	112.5
127.0	127.0	127.0	112.5	112.5	112.5	112.5	112.5	112.5	112.5
127.0	127.0	127.0	112.5	112.5	112.5	112.5	112.5	112.5	112.5
107.0	107.0	107.0	127.0	127.0	127.0	127.0	127.0	127.0	127.0
117.0	117.0	117.0	127.0	127.0	127.0	127.0	127.0	127.0	127.0
117.0	117.0	117.0	127.0	127.0	127.0	127.0	127.0	127.0	127.0
117.0	117.0	117.0	127.0	127.0	127.0	127.0	127.0	127.0	127.0
122.0	122.0	122.0	127.0	127.0	127.0	127.0	127.0	127.0	127.0
122.0	122.0	122.0	127.0	127.0	127.0	127.0	127.0	127.0	127.0
117.0	117.0	117.0	127.0	127.0	127.0	127.0	127.0	127.0	127.0
117.0	117.0	117.0	127.0	127.0	127.0	127.0	127.0	127.0	127.0
117.0	117.0	117.0	127.0	127.0	127.0	127.0	127.0	127.0	127.0
117.0	117.0	117.0	127.0	127.0	127.0	127.0	127.0	127.0	127.0
127.0	127.0	127.0	22.4	22.4	22.4	22.4	98.0	98.0	98.0
127.0	127.0	127.0	22.4	22.4	22.4	22.4	98.0	98.0	98.0
127.0	127.0	127.0	22.4	22.4	22.4	22.4	98.0	98.0	98.0
127.0	127.0	127.0	22.4	22.4	22.4	22.4	98.0	98.0	98.0
127.0	127.0	127.0	22.4	22.4	22.4	22.4	98.0	98.0	98.0
117.0	117.0	117.0	127.0	127.0	127.0	127.0	127.0	127.0	127.0
117.0	117.0	117.0	127.0	127.0	127.0	127.0	127.0	127.0	127.0
179.0	179.0	179.0	179.0	179.0	179.0	179.0	179.0	179.0	179.0
127.0	127.0	127.0	90.7	90.7	90.7	90.7	98.0	98.0	98.0
0.0	63.0	63.0	127.0	127.0	127.0	127.0	127.0	127.0	127.0
63.0	0.0	18.9	127.0	127.0	127.0	127.0	127.0	127.0	127.0
63.0	18.9	0.0	127.0	127.0	127.0	127.0	127.0	127.0	127.0
127.0	127.0	127.0	0.0	11.2	11.2	11.2	98.0	98.0	98.0
127.0	127.0	127.0	11.2	0.0	11.2	11.2	98.0	98.0	98.0
127.0	127.0	127.0	11.2	11.2	0.0	11.2	98.0	98.0	98.0
127.0	127.0	127.0	11.2	11.2	11.2	0.0	98.0	98.0	98.0
127.0	127.0	127.0	98.0	98.0	98.0	98.0	0.0	27.0	27.0
127.0	127.0	127.0	98.0	98.0	98.0	98.0	27.0	0.0	27.0
127.0	127.0	127.0	98.0	98.0	98.0	98.0	27.0	27.0	0.0
